# Structure Revision of Pyranoxanthones via DFT-Assisted ^13^C NMR Analysis and NAPROC-13 Platform: Diagnostic Markers and Discovery of Unreported Natural Products

**DOI:** 10.1021/acs.joc.5c01371

**Published:** 2025-11-03

**Authors:** Hugo A Sánchez-Martínez, Juan A. Morán-Pinzón, Esther del Olmo, José F. Adserias Vistué, Estela Guerrero De León, José L. López-Pérez

**Affiliations:** † CIPFAR. Departamento de Farmacología. Facultad de Medicina. 54717 Universidad de Panamá, Ave. Octavio Mendez Pereira, 0824 Panama City, Panama; ‡ Departamento de Ciencias Farmacéuticas, Área de Química Farmacéutica, Facultad de Farmacia, CIETUS, IBSAL. Campus Miguel de Unamuno, 16779 University of Salamanca, 37007 Salamanca, Spain; § Departamento de Sistemas, Fundación General, 16779 University of Salamanca, Fonseca 2, 37002 Salamanca, Spain

## Abstract

The structures of 47 natural and synthetic pyranoxanthones, previously misassigned in the literature, were revised using the NAPROC-13 platform in combination with DFT-based ^13^C NMR calculations. Structural misassignments primarily involved incorrect prenyl group localization or misplacement of the oxygen atom involved in the pyran ring. NAPROC-13 searches frequently revealed alternative, structurally distinct compounds sharing identical ^13^C NMR data, or instances where the same compound had been reported with divergent data sets. DFT-calculated chemical shifts confirmed the revised structures, which included six previously unreported natural products. Systematic deviations between calculated and experimental values were observed for carbons adjacent to the carbonyl and for the olefinic carbons of the pyran ring. Statistical analysis of a 76-compound modeling set yielded empirical correction factors that markedly improved data concordance. This combined database–computational approach demonstrates high reliability in structural validation and is applicable to other families of natural products. Additionally, diagnostic ^13^C NMR chemical shifts were identified for carbons adjacent to the pyran ring, offering a rapid tool for determining the site of fusion on the xanthone scaffold.

## Graphical abstract

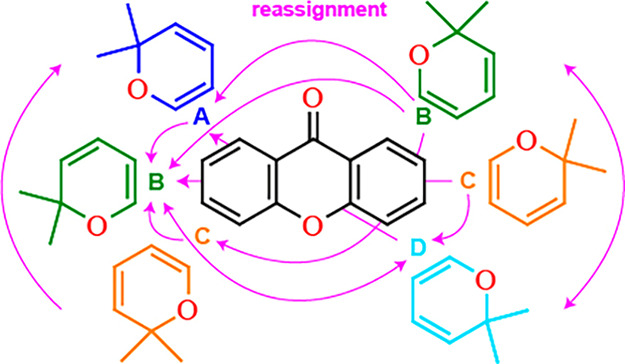

## Introduction

Xanthones are secondary metabolites widely distributed in nature. Their basic structure consists of two benzene rings linked by a carbonyl group and an oxygen atom, forming a dibenzo-γ-pyrone system known as 9*H*-xanthen-9-one according to IUPAC nomenclature. Carbon atoms are numbered following the IUPAC 2004 recommendations (Figure 1a). Despite the apparent symmetry of the central scaffold, xanthones show remarkable structural diversity. This diversity arises primarily from the frequent attachment of prenyl groups to any carbon atoms of the xanthone core and, in some cases, their cyclization via adjacent oxygen atoms, leading to fused pyran rings (Figure 1b). The anchoring of these pyran groups at different positions on the xanthone skeleton generates isomeric pyranoxanthones, which often display similar ^13^C NMR data, complicating their accurate structural elucidation. Additionally, oxygenated functional groups, such as hydroxyl or methoxy groups, may be present elsewhere in the xanthone nucleus (Figures 2 and 3). A significant number of pyranoxanthones, both naturally occurring and synthetically derived, have been described. These compounds are often synthesized either to confirm the structure of a natural product^1^ or to expand structural diversity.^2^

**Figure 1:**
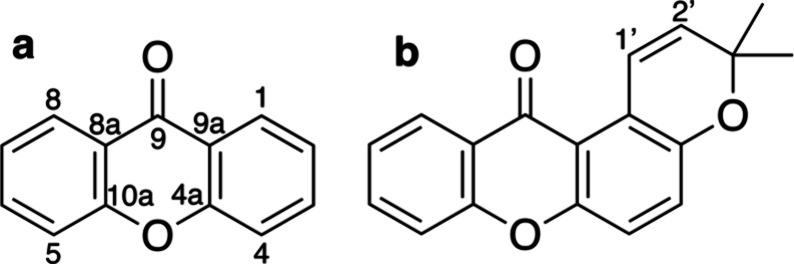
Structures of as 9*H*-xanthen-9-one (a) and pyranoxanthone (b).

**Figure 2:**
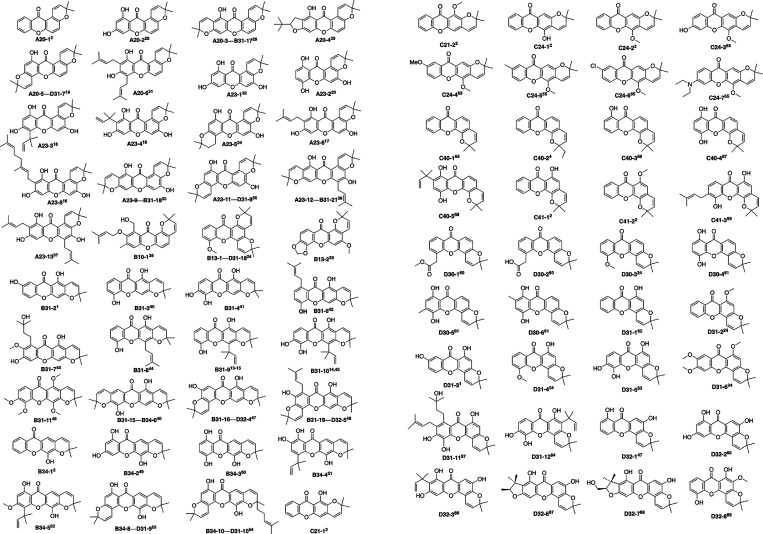
Modeling pyranoxanthones with subtype codes (Figure 3) and sequential numbers; dual codes denote two pyran rings. References in superscript.^1,2,4,13–19,24,28–69^

**Figure 3:**
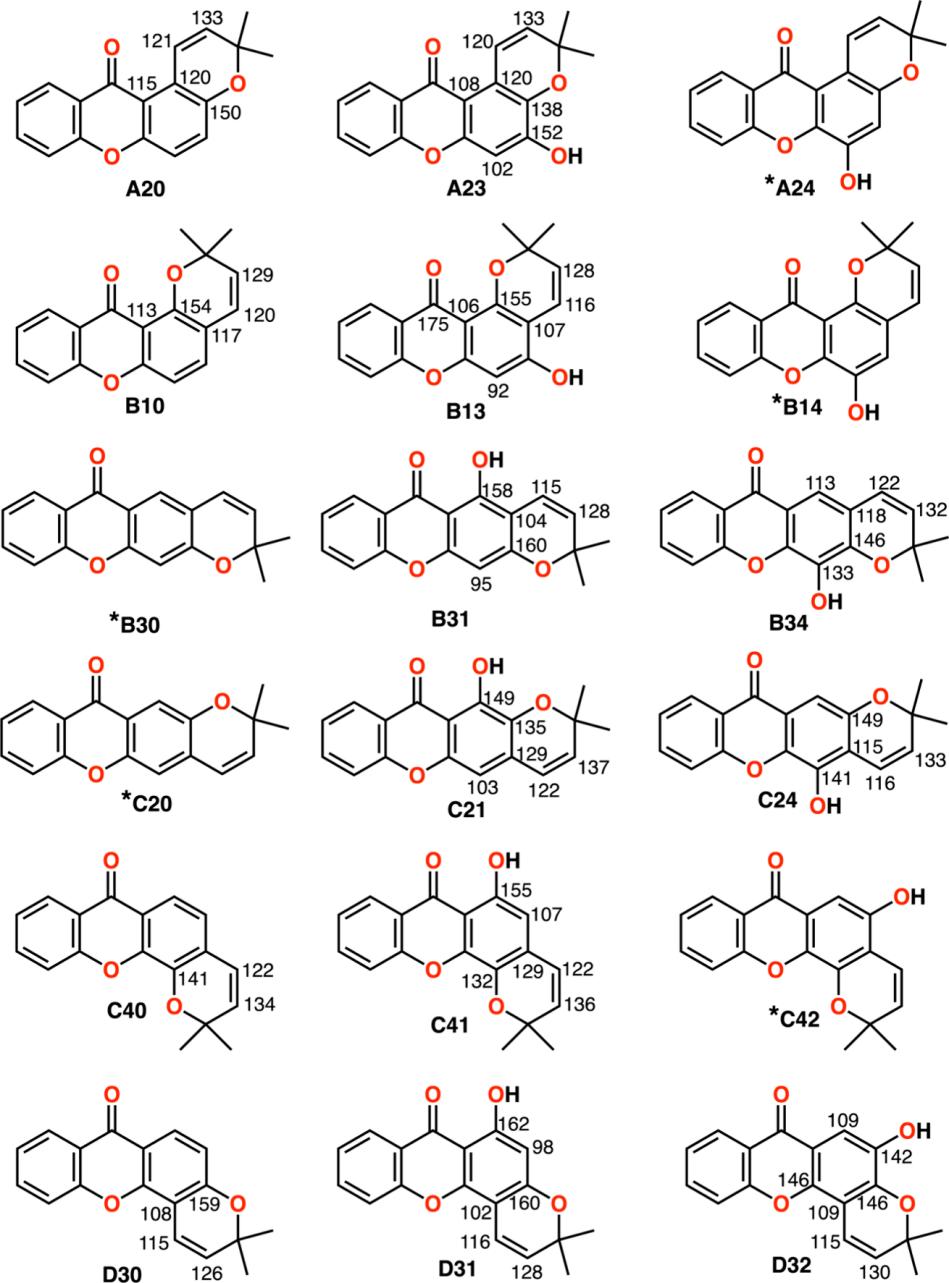
Classification of pyranoxanthones by type (A-D) and subtype (two-digit numerals), with designations shown below each structure. Diagnostic ^13^C NMR shifts are provided for each subtype. Subtypes lacking ^13^C NMR data are marked with a superscript asterisk.

Xanthones have shown a wide range of biological activities, including antitumor effects.^3,4^ Numerous additional activities have been documented by Gul et al.,^5^ along with insights into the molecular targets and cellular mechanisms associated with these compounds. The pharmacological relevance of xanthones has spurred research into their structure–activity relationships. Different substitution patterns in these natural products can result in distinct biological activities.^6,7^ Of particular interest is the positioning of oxygenated functional groups, such as hydroxyl and methoxy groups, within the xanthone skeleton, as these play a critical role in determining the compound’s activity. Additionally, the recurrent presence of prenyl groups, which give rise to condensed pyrans, introduces steric factors that influence interactions at receptor sites. Precise localization of the attachment points of these prenyl groups to the xanthone core, along with accurate structural elucidation, is essential for establishing robust structure–activity relationships.

NAPROC-13 ^8^ is a freely accessible and searchable database available at c13.usal.es. It compiles structural and ^13^C NMR spectroscopic data for over 28,000 natural products (NPs), including approximately 1,700 xanthones. This comprehensive representation facilitates successful dereplication and the elucidation of new structurally related xanthones through comparative analysis of ^13^C NMR data. Two recent studies highlight the utility of NAPROC-13 as a novel strategy for identifying erroneously assigned NPs.^9,10^

## Results and Discussion

Although the structural simplicity and symmetry of the xanthone core might suggest straightforward elucidation, this is not always the case. Despite their nominal C_2_v symmetry, the benzene rings are not ideal hexagons, and the central pyran ring—only partially aromatic—adds complexity. Substituents frequently distort the scaffold, inducing a propeller-like twist along the longitudinal axis due to steric effects. The C = O bond length, typically 1.22 Å in xanthen-9-one, may elongate to 1.24–1.28 Å when the carbonyl group participates in intramolecular hydrogen bonding.^11^ These perturbations substantially affect the ^13^C NMR chemical shifts, complicating straightforward structural elucidation based solely on 2D representations. For example, the carbonyl carbon resonates at ∼ 176 ppm in the absence of hydrogen bonding but shifts up to ∼ 186 ppm when simultaneously engaged in two intramolecular H-bonds with hydroxyl groups at C-1 or C-8. Methoxy groups on the aromatic rings substantially influences the chemical shifts of carbons in *ortho* and *para* positions, depending on coplanarity or orthogonality due to steric hindrance. This effect is especially pronounced at C-1 or C-8, where steric repulsion with the C-9 carbonyl may alter the local environment.^12^ Additional challenges arise from low-intensity signals of quaternary carbons within a narrow δ range and the subtle NMR differences among regioisomeric pyranoxanthones. Beyond the geometric deformations induced by substituents on the xanthone core, the isoprenyl side chains often adopt conformations that deviate from the molecular plane, further increasing structural variability.^12^ Collectively, these factors contribute to frequent misassignments and also to the largest deviations between calculated and experimental chemical shifts near the carbonyl region, including the double bonds of pyran ring.

NAPROC-13 enables direct comparison of closely related xanthone structures and their ^13^C NMR shifts (See SI: I. Figures SI-2, SI-3, SI-4 and SI-5), facilitating the identification of inconsistencies. For example, three database entries corresponding to the same compound—inoxanthone^13^ also known as blancoxanthone^14^ and caloxanthone C^15^ (**B31–9**, Figure 2)—exhibit notable spectral differences despite being acquired in the same solvent (CDCl_3_). A striking discrepancy is observed at C-4a, resonating at 157 ppm in inoxanthone and 144 ppm in blancoxanthone, likely reflecting spectral misinterpretation or sample contamination. Additionally, the assignments for C-8a and C-9a are reversed between data sets. Searches by chemical shift or substructure can also reveal instances where identical ^13^C NMR data have been assigned to distinct structures or, conversely, where divergent data are reported for the same compound—both cases warranting reassessment. DFT-based ^13^C NMR calculations help verify structural correctness, as misassigned compounds typically show several large deviations between experimental and computed shifts. In some cases, however, only one signal showed a large deviation, suggesting a typographical error, misinterpreted spectrum, or contamination. Representative examples are shown in SI (Section I, Figures SI-4 and SI-5). For allanxanthone B^16^ (**A23–8**, Figure 2, SI: V.13), the C-7 signal at 144.1 ppm deviated by 8.2 ppm, while the remaining values, after empirical correction, matched well. The weak intensity of this signal—likely due to poor relaxation from a lack of neighboring protonated carbons—may have led to its misreporting. In the related garcinone B^17^ (**A23–6**, Figure 2, SI: V.12), C-7 resonates at 138.1 ppm, closely matching the calculated value of 135.9 ppm for both compounds. A more complex issue involves the misassignment of olefinic methine signals from the pyran ring, swapped with aromatic CH signals. This is illustrated by cudraxanthone A (**A20–5–D31–7**, Figure 2; SI: V.5), as reported by Hano et al.^18^ and Chang et al.^19^ Hano et al. correctly assigned the 120.8 ppm signal to the pyran C-1’’, classifying it as A20. Chang et al., however, attributed this value to CH-5 and reassigned the pyran methine to 117.6 ppm. As a result, the reported chemical shifts are inconsistent with those in Figure 3 or may match an alternative structure. In such cases, further comparisons with structurally related xanthones are required to confirm signal identity.

GIAO (gauge-independent atomic orbital) ^13^C NMR chemical shift calculations are now widely recognized as a reliable tool for the structural elucidation of complex NPs.^20,21^ Given the sensitivity of chemical shifts to molecular conformation, we applied the protocol implemented in Spartan’24 (using Spartan’20 conditions^22^) for the calculation of ^13^C NMR chemical shifts in pyranoxanthones. The most accurate predictions are obtained by Boltzmann-weighted averaging across all conformers, which requires precise evaluation of their relative energies. Accordingly, we employed the ωB97X-V functional with the extended 6–311+G­(2df,2p)­[6–311G*] basis set, well-suited for long-range interactions. Additionally, recurrent misassignments of quaternary carbon shifts observed —particularly for C-8a and C-9a—underscore the robustness and reliability of the computational NMR approach employed herein.

This methodology, previously applied with success,^9,10^ involved building up the structures in Spartan’24 applying the full computational protocol for ^13^C NMR calculations implemented in Spartan’20 (see Experimental Section). Agreement between calculated and experimental chemical shifts was assessed using rmsd and max dev statistical parameters (SI: V and VIII). When multiple alternatives are possible, the most probable structure was identified based on these metrics and the DP4 probability score (Goodman’s method).^23^

## Modeling Set of Pyranoxanthones: Empirical Correction Factors

To validate the previously mentioned approach, we selected a modeling set comprising seventy-six pyranoxanthones from the literature and the NAPROC-13 database (Figure 2) prioritizing structures confirmed by X-ray crystallography,^12^ total synthesis,^2,24^ or high-quality ^13^C NMR data. These compounds were systematically categorized into four main types (A–D) based on the prenylation site (C-1 to C-4) and further divided into subtypes according to the position of the pyran oxygen and any additional oxygenated substituents (Figure 3). Type A includes compounds with the prenyl group linked to C-1 of the xanthone core; in type B, the prenyl group is linked to C-2; in type C, it is linked to the C-3 and finally, in type D, it is linked to the C-4. Each subtype is designated by two digits following the type letter. The first digit corresponds to the xanthone carbon bonded to the pyran oxygen, while the second digit is zero when no additional oxygenated functionality is present on the aromatic ring fused to the pyran, or ranges from 1 to 4 depending on the position of such a substituent (Figure 3). Not all subtypes represented exist, and some lack ^13^C NMR data. Specifically, no representatives of subtypes B14, C20, or C42 have been reported, while compounds to B30-subtype have been synthesized but lack ^13^C NMR data.^25^ In contrast, synthetic compounds from subtypes C21 and C24 have been described^2^ despite the absence of corresponding NPs. All of NPs previously assigned to A24 were reclassified to A23 ^26,27^ based on ^13^C NMR analysis in this investigation. Subtypes for which no ^13^C NMR data are currently available are denoted by a superscript asterisk in Figure 3.

Figure 3 summarizes the key experimental ^13^C NMR chemical shifts of carbons adjacent to the pyran ring, as reported in the literature for selected subtypes. These chemical shifts are consistent within subtypes but vary by pyran ring attachment, presence or absence of oxygenated functions, and their positions. As subtype-specific markers, they aid in the rapid assignment of new pyranoxanthones and help minimize misassignments during structural elucidation. This approach offers a practical tool for the expedited identification of pyranoxanthones not yet reported in the literature.

The seventy-six xanthones selected as the modeling set (Figure 2) were subjected to DFT-calculated ^13^C NMR chemical shifts, following the protocol described in the Experimental Section. Overall, good agreement was observed between calculated and experimental chemical shifts; however, systematic deviations were noted for C-1 and C-8 of the xanthone core, as well as for C-1′ and C-2′ olefinic carbons of the pyran moiety. Specifically, calculated values for C-1, C-8, and C-1′ were consistently higher than the experimental values, while those for C-2′ were lower (SI: III, Table S-1). To correct these systematic discrepancies, empirical correction factors were derived through statistical analysis of the modeling set (for details of the statistical analysis, see Supporting Information, Section IV). The statistical analysis was performed using the SIMFIT program, both for each individual type (A, B, C, and D) and for the combined data set (″gathered″). Given the symmetrical positions of C-1 and C-8 and their similar deviations, they were evaluated together. For simplicity and broader applicability, correction factors were ultimately derived from the gathered data set, which provided the most representative mean values: – 2.9 ppm for C-1, and C-8, – 3.7 ppm for C-1′, and +2.8 ppm for C-2′ (SI: IV. Tables SI-2.1, SI-2.2 and SI-2.3). Application of these four correction factors significantly improved the agreement between calculated and experimental ^13^C NMR chemical shifts, as shown in SI (Sections V and VIII).

## Misassigned Structures

Combined substructure–chemical shift searches in NAPROC-13 were conducted for each pyranoxanthone subtype shown in Figure 3 (SI: I, Figures SI-3 to SI-5). This systematic approach enabled rapid identification of compounds whose ^13^C NMR chemical shifts deviate from the subtype reference markers. As a result, forty-seven literature-reported pyranoxanthones showing inconsistencies between their proposed structures and ^13^C NMR data were recognized as misassigned. All compounds requiring revision are listed systematically as **R1**—**R31** in Table 1 (“Original Assignment”) and discussed in the manuscript. Their structures are organized by the originally reported subtype. Entry **R22** includes 17 synthetic derivatives (**R22a** —**R22q**) also requiring reassignment. Six compounds were revised as previously undescribed natural products, while the rest show ^13^C NMR data matching those of known corrected structures (SI: VII). Five additional compounds (Figure 4) could not be reassigned, as their ^13^C NMR shifts were incompatible with any subtype model in Figure 3 and yielded no matches in NAPROC-13. The calculated ^13^C NMR shifts for each misassigned compound, together with the corresponding experimental data and statistical parameters derived after applying empirical correction factors from the modeling set, are provided in the Supporting Information (Section VIII). The alphanumeric code following the dash serves as a structural identifier consistent with the coding scheme in Figure 3, facilitating correlation with the training set (Figure 2). Reassigned structures are shown in the second column (“Revised Assignment”). For consistency, consecutive numbers appear after the hyphen following each subtype code (Figure 3). For example, **R19–C40** indicates that compound **R19** was originally misassigned to subtype **C40**, whereas its revised structure is **A20–2**, the analog of **R19** within subtype **A20** (Figure 2). For compounds bearing two pyran rings, the code is duplicated and separated by a double hyphen, each segment identifying the pyran position. The misassigned structures span all four types (A–D; Figure 3), though types B and D predominate. Common names are included when available. Below each structure, the product code appears along with the RMSD and maximum deviation (in parentheses) between calculated and experimental ^13^C NMR shifts. In all cases, these values improve substantially for the revised structures and generally fall within the expected range. Larger deviations in a few entries (noted in the third column) result from a single outlier—typically a weak quaternary carbon signal. Comments and justifications for each reassignment are summarized in the third column (“Key Evidence and Justification”).

**Table 1:**
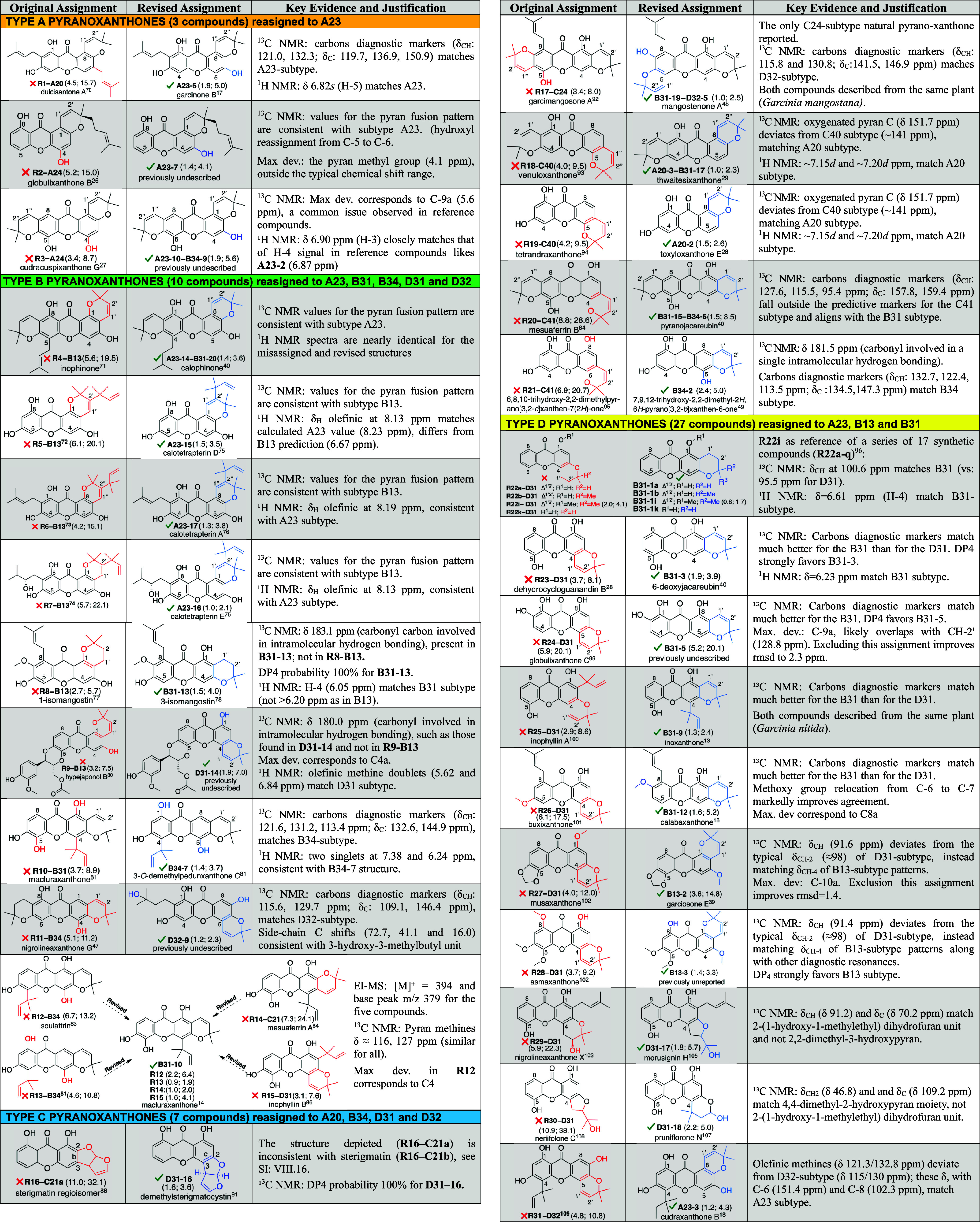
Structures of Pyranoxanthones Revised in This Work

**Figure 4:**
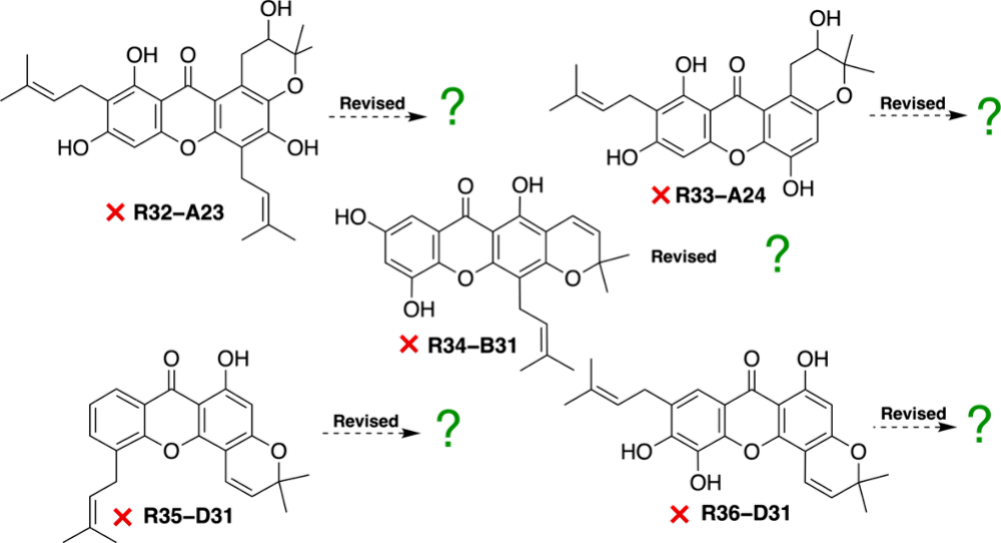
Pyranoxanthone structures in need of revision. A consistent solution has not yet been found.

### Type A Pyranoxanthones Reassigned (R1–A20 to R3–A24)

Three compounds originally assigned to subtypes A20 or A24 were reassigned to A23 based on diagnostic ^13^C NMR markers (Figure 3). A NAPROC-13 search for dulcisantone A^70^ (**R1–A20**) retrieved several A23-subtype pyranoxanthones, including garcinone B^17^ (**A23–6**), differing only by a C-6 hydroxyl group instead of the reported prenyl substituent. Excluding the signals from this group, the ^13^C NMR shifts match closely, with a maximum deviation of 2.6 ppm in only one carbon. DFT calculations showed markedly better agreement for **A23–6** than for **R1–A20**, with the largest deviation at CH-5 (15.7 ppm) in the misassigned structure. The ^1^H NMR singlet at δ 6.82 ppm—present in both dulcisantone A and garcinone B— agrees with the predicted value for **A23–6** (δ_H‑5_ = 6.7 ppm), whereas a C-6 prenyl group would shift H-5 downfield to ∼ 7.1 ppm. Additional ^13^C signals attributed to prenylation in **R1–A20** most likely arise from minor impurities. These findings establish that dulcisantone A (**R1–A20**) is identical to garcinone B (**A23–6)**. Similarly, globulixanthone B^26^ (**R2–A24**) and cudracuspixanthone G^27^ (**R3–A24**) were reassigned to **A23–7** and **A23–10–B34–9**, respectively, with improved DFT agreement after hydroxyl repositioning from C-5 to C-6. The ^1^H NMR singlet at 6.90 ppm in **R3–A24** matches H-4 signals in A23 reference compounds^33^ (6.87 ppm). Structures **A23–7** and **A23–10–B34–9** represent previously unreported natural products.

### Type B Pyranoxanthones Reassigned (R4–B13 to R13–B34)

The first four compounds (**R4–B13** to **R7–B13**) were reassigned to A23 based on pyran carbon shifts deviating at C-3 by up to 22 ppm from B13 patterns while matching A23 diagnostic values (Figure 3). Inophinone^71^ (**R4–B13**) showed ^13^C NMR data nearly identical to calophinone^40^ (**A23–14–B31–20**), which structure is confirmed by DFT calculations. NAPROC-13 searches using their ^13^C shifts of three additional compounds—**R5–B13**,^72^ **R6–B13**,^73^ and **R7–B13** ^74^—identified these structurally distinct but spectroscopically equivalent pyranoxanthones, all isolated from two *Calophyllum* species and reported as calotetrapterins D^75^ (**A23–15**), A^76^ (**A23–17**), E^75^ (**A23–16**). DFT calculations confirmed the A23 assignments with significantly improved statistical parameters.

**R8–B13**, a semisynthetic pyranoxanthone reported by Chi et al.,^77^ was initially misnamed as ″1-isomangostin hydrate″. The authors’ textual discussion and spectroscopic data support the corrected designation of 1-isomangostin; however, the ^13^C carbonyl shift observed for **R8–B13** (δ_C_ 183.1 ppm) is characteristic of hydrogen-bonded carbonyls in B31-subtype compounds, such as 3-isomangostin^78^ (**B31–13**), and inconsistent with the value (δ_C_ 176.1 ppm) reported by Morelli et al.^79^ for 1-isomangostin. DFT calculations including DP4 analysis (100% probability) confirmed the reassignment to 3-isomangostin (**B31–13**).

Hypejaponol B^80^ (**R9–B13)** displayed key pyran carbon signals deviating markedly from the typical B13 pattern but aligning with those of the D31 subtype (Figure 3). Furthermore, the carbonyl carbon at δ 180.0 ppm suggests the presence of an intramolecular hydrogen bond in agreement with the D31-subtype. DFT calculations favored **D31–14** over **R9–B13**, identifying this as a previously unreported natural product. The only noteworthy deviation in the **D31–14** assignment (7.0 ppm) involves the signal attributed to C-4a with the same shift value (160.5 ppm) assigned to both C-4a and C-3 (SI: VIII.9b). This suggests that a single, double-intensity signal was misinterpreted.

The ^13^C NMR data provided by Goh et al.^81^ for **R10–B31** differ significantly from those previously published for macluraxanthone^45,53^ (**B31–10**; Figure 2) but are nearly identical to those of pedunxanthone C^52^ (**B34–5**) (Figure 2). DFT calculations on the demethylated derivative matched well with the experimental data reported for demethylpedunxanthone C^81^ **(B34–7)**, supporting the proposed reassignment. Although the authors did not provide the ^1^H NMR in their publication, they referred to those reported by Monache et al.,^82^ which include two singlets at 7.38 and 6.24 ppm—values consistent with **B34–7.**

Nigrolineaxanthone G^47^ (**R11–B34**) exhibits ^13^C NMR spectral profile typical of a D32-subtype pyranoxanthone (Figure 3), further supported by a singlet at 7.63 ppm in its ^1^H NMR spectrum—identical to the single aromatic proton observed in nigrolineaxanthone H (**D32–1**)^47^ (Figure 2; SI: V-71). Additional resonances (δ_C_ 72.7, 41.1, 16.0 ppm) indicate the presence a 3-hydroxy-3-methylbutyl side chain rather than a dihydropyran moiety.^77^ The calculated spectra and experimental data consistently favor **D32–9** (SI: VIII.11b), representing a newly characterized natural product.

Four structurally diverse compounds—soulattrin^83^ (**R12–B34**), compound **R13–B34**,^81^ mesuaferrin A^84,85^ (**R14–C21**), and inophyllin B^86,87^ (**R15–D31**)—originally assigned to subtypes B34, C21, or D31, all exhibited pyran methine carbons at approximately 116 and 127 ppm with deviations below 1 ppm. These signal together with other ^13^C NMR chemical shifts closely matched the diagnostic marker values characteristic of the B31 subtype. NAPROC-13 substructure searches retrieved macluraxanthone (**B31–10**),^14,45,53^ whose ^13^C data matched closely with those of the four compounds. Supporting evidence included identical EI-MS fragmentation patterns ([M^+^] = 394, base peak *m*/*z* 379) and ^1^H NMR doublets at 6.9 and 7.5 ppm matching the **B31–10** aromatic pattern. For **R12–B34**, persistent deviations at C-4 (6.4 ppm) and the prenyl olefinic CH (5.2 ppm) suggest possible signal misassignment. The substantial ^13^C discrepancies between inophyllin B (**R15–D31**) and cudratricusxanthone H^63^ (**D31–12**; Figure 2)—to which it was claimed structurally identical—further support the need for structural revision of **R15–D31.**

### Type C Pyranoxanthones Reassigned (R14–C21, R16–C21 to R21–C41)

In addition to the compound **R14–C21** discussed in the preceding section, six other compounds originally assigned to subtype C pyranoxanthones (**R16–C21** through **R21–C41**) required structural revision. Sala et al. reported the structure depicted as **R16–C21a** for sterigmatin from *Aspergillus nidulans*;^88^ however, this structure is inconsistent with the X-ray confirmed [3,2-*b*] furan fusion of authentic sterigmatin^89,90^ (**R16–C21b**, SI: VIII.16b) rather than the [2,3-*b*] fusion depicted by Sala, T. et al. NAPROC-13 searches identified demethylsterigmatocystin^91^ (**D31–16**) as a potential match. DFT calculations yielded rmsd values of 11.0 ppm (**R16–C21a**), 4.2 ppm (**R16–C21b**), and 3.6 ppm (**D31–16**), with a DP4 probability of 100% for **D31–16**. The compound is accordingly reassigned to demethylsterigmatocystin.

Garcimangosone A^92^ (**R17–C24**), the only natural pyranoxanthone reported under the C24 subtype, exhibited ^13^C NMR pyran ring signals matching D32-subtype patterns. NAPROC-13 retrieved mangostenone A^48^ (**B31–19–D32–5**), a compound previously isolated from the same plant source (*Garcinia mangostana*), showing closely matching ^13^C NMR data. DFT calculations confirmed superior agreement for **B31–19–D32–5**, leading to the reassignment of garcimangosone A to this structure. Venuloxanthone^93^ (**R18–C40**) and tetrandraxanthone^94^ (**R19–C40**) both displayed an oxygenated pyran carbon signal at 151.7 ppm, deviating from the expected ∼ 141 ppm for C40-subtype but matching A20-subtype patterns. Combined spectroscopic analysis and computational analyses supported their reassignment as thwaitesixanthone^29^ (**A20–3–B31–17**) and toxyloxanthone E^28^ (**A20–2**), respectively. The dipyranoxanthone mesuaferrin B^84^ (**R20–C41**) and the compound reported by Han et al.^95^ **R21–C41** both exhibit ^13^C NMR chemical shifts incompatible with C41-subtype patterns. Detailed analysis revealed that **R20–C41** corresponds to pyranojacareubin^40^ (**B31–15–B34–6**), while **R21–C41** matches the spectroscopic profile of the known compound **B34–2**.^49^ Both reassigned structures show markedly improved statistical agreement after correction.

### Type D Pyranoxanthones (R22–D31 to R31–D32)

The largest group of pyranoxanthones revised in this study corresponds to subtype D31, comprising twenty-six compounds (**R22a–q–D31** to **R31–D32**), in addition to **R15–D31**, discussed above. Most of these compounds are reassigned to the B31-subtype. This high rate of misassignments arises from to the striking similarity in the ^13^C NMR chemical shifts of carbons adjacent to the pyran ring in both angular (D31) and linear (B31) pyranoxanthones. Detailed analysis of the modeling data set revealed subtle yet consistent differences in the ^13^C NMR shifts between the two subtypes (Figure 3), enabling more reliable structural reassignments. A particularly significant case involves 17 synthetic pyranoxanthones (**R22a–q–D31**) reported by Yong Rok and Gala Sri^96^ who claimed a regioselective synthesis yielding exclusively angular D31-subtype pyranoxanthones. However, detailed spectroscopic analysis revealed systematic discrepancies aligning with diagnostic markers of B31-subtype rather than those of the angular D31 form. The methylated derivative **R22i–D31** was selected for detailed comparison as both B31- and D31-regioisomers have reported ^13^C NMR data in NAPROC-13. The experimental data show a methine carbon at 100.6 ppm and quaternary carbons at 159.2, 156.3, and 112.2 ppm—values nearly identical to those of the linear B31 regioisomer^97^ and distinctly different from the angular D31 isomer.^24^ Moreover, the ^1^H NMR singlet at 6.61 ppm matches the linear isomer (contrasting with 6.34 ppm for the angular form). DFT calculations for three representative compounds yielded DP4 probabilities of 100%, unequivocally supporting the linear B31-subtype structures. Consequently, the entire series **R22a–q–D31** is reassigned to the corresponding **B31–1a** to **B31–1q** structures. Notably, this work inadvertently established a regioselective synthetic route to linear B31-subtype pyranoxanthones, complementing the tosyl-directed strategy developed by Ming Dai et al.^98^ for accessing angular D31-subtype compounds. Their approach involves introducing a bulky *p*-toluenesulfonyl (*p*-tosyl) group at the C-1 hydroxyl, which hinders formation of the linear B31-subtype—presumably through steric effects—thereby directing the Claisen-type cyclization toward C-4 and yielding angular D31 products.

The following four compounds (**R23–D31** to **R26–D31**) were likewise reassigned to B31-subtype structures. Dehydrocycloguanandin B^28^ (**R23–D31**) was reassigned as 6-deoxyjacareubin^40^ (**B31–3**) with DP4 probability of 100%. Globulixanthone C^99^ (**R24–D31**) was reassigned to **B31–5**, representing a previously unreported natural pyranoxanthone. The initially problematic C-9a signal at 128.8 ppm likely resulted from overlap with the CH-2’ resonance; excluding this value substantially improved the statistical agreement (rmsd = 2.3 ppm; Table 1). Structurally inophyllin A^100^ (**R25–D31**) has been described as the C-6 dehydroxy derivative of inophyllin B^86,87^ (**R15–D31**), both isolated from *Garcinia nitida*.^92^ Inophyllins A and B exhibit nearly identical spectroscopic profiles, differing only in the presence or absence of the C-6 hydroxyl group. Considering this close similarity, and given the previous reassignment of **R15–D31** to the linear analogue macluraxanthone (**B31–10**), **R25–D31** should likewise be reassigned to its corresponding linear pyranoxanthone of the B31 subtype—inoxanthone^13^ (**B31–9**; Figure 2)—with which it shares identical spectroscopic data. DFT-based ^13^C NMR calculations for **R25–D31** in both angular and linear forms further support its reassignment to **B31–9**. In light of the reassignments of **R24–D31** and **R25–D31**, a lineal B31-subtype is also proposed for buxixanthone^101^ (**R26–D31**) first reported as an angular pyranoxanthone. Computational predictions align slightly better with the experimental ^13^C NMR data for **B31–12a** (SI: VIII.26). However, the largest deviation—exceeding 17 ppm—persists at C-5 in both models. Relocation of the methoxy group from C-6 to C-7 markedly improved the agreement, yielding **B31–12**. This revised structure matches calabaxanthone,^18^ whose ^13^C NMR data correspond closely to those of **R26–D31.**

Musaxanthone (**R27–D31**) and asmaxanthone (**R28–D31**), both angular pyranoxanthones isolated from the *Garcinia rigida* ^102^ exhibited CH-2 signals at 91.6 and 91.4 ppm, respectively, characteristic of B13-subtype rather than D31-subtype patterns (∼98 ppm; Figure 3). NAPROC-13 searches and computational analysis supported the reassignment of **R27–D31** as garciosone E^39^ (**B13–2**) and **R28–D31** as the previously unreported natural product **B13–3**. Calculated ^13^C NMR data for **B13–2** matched **R27–D31** except for C-10a (a remote carbon unaffected by reassignment), which deviated by 14.8 ppm—mirroring the original structure. This discrepancy likely arose from the weak C-10a intensity, a frequent pitfall in pyranoxanthones. The ^13^C NMR spectrum of nigrolineaxanthone X^103^ (**R29–D31**) displays a methine carbon signal at 91.2 ppm and a quaternary carbon signal at 70.2 ppm, which are inconsistent with the proposed 2,2-dimethyl-3-hydroxypyrano ring for this xanthone but instead characteristic of a 2-(1-hydroxy-1-methylethyl)­dihydrofuran moiety.^104^ A NAPROC-13 search identified morusignin H^105^ (**D31–17**) as the correct structure, corroborated by computational analysis and diagnostic ^1^H NMR signals —3.34 ppm (CH_2_) and 4.90 ppm (CH–O)—matching those observed in morusignin H and related xanthones. Although the same 2-(1-hydroxy-1-methylethyl)­dihydrofuran motif was proposed for neriifolone C^106^ (**R30–D31**), the reported ^13^C NMR chemical shifts do not support this structural feature. A NAPROC-13 search, identified pruniflorone N^107,108^ (**D31–18**) as a better match, a conclusion reinforced by DFT-based ^13^C NMR calculations, which showed excellent agreement with the experimental data. Finally, the D32-subtype assignment for **R31–D32** ^109^ was invalidated by pyran ring olefinic methines at 121.3 and 132.8 ppm— values inconsistent with D32 expectations but matching A23-subtype (Figure 3). Spectroscopic comparison and computational data support reassignment of **R31–D32** to cudraxanthone B^18^ (**A23–3**).

### Unresolved Structures

Five pyranoxanthones—butyraxanthone C^110^ (*R* **32–A23**), garcimangosone C^92^ (**R33–A24**), baphikixanthone B^111^ (**R34–B31**), laurentixanthone A^112^ (**R35–D31**), and paucinervins I^113^ (**R36–D31**)—could not be satisfactorily reassigned (Figure 4). Their reported ^13^C NMR data showed no correspondence with any reference compounds in NAPROC-13 and were incompatible with all subtype models presented in Figure 3. These discrepancies likely indicate that the reported spectroscopic data include contributions from sample impurities rather than representing single pure compounds.

## Conclusions

In this study, the structures of forty-seven natural and synthetic pyranoxanthones were reassigned, addressing persistent misassignments in the literature through the integrated application of the NAPROC-13 database and DFT-based ^13^C NMR chemical shift calculations. The classification proposed herein, based on the position of the pyran ring within the tricyclic core, establishes four structural types and their diagnostic subtypes, thereby improving the recognition of misassigned structures. Empirical correction factors derived from a comprehensive statistical analysis markedly increased the accuracy of DFT-based predictions, resolving frequent misplacements of quaternary carbons such as C-8a and C-9a. Six previously unreported natural products were identified in the course of this work. Beyond pyranoxanthones, this combined database–computational workflow offers a broadly applicable strategy for structural validation across other natural product classes, particularly when experimental NMR data are compromised by signal overlap or sample impurities.

## Experimental Section

Computational calculations were performed using Spartan’24 (Wave function Inc., Irvine, CA, USA) following the default Spartan’20 recipe available in Spartan’24.^22^ This procedure comprised six steps: (1) a systematic conformational search using MMFF molecular mechanics, removing duplicate conformers and those with energy 40 kJ/mol above the global minimum; (2) geometry optimization using HF/3–21G, again discarding duplicates and conformers exceeding the 40 kJ/mol threshold; (3) single-point energy calculations at the ωB97X-D/6–31G* level and removing conformers 15 kJ/mol above the global minimum; (4) further geometry optimization with ωB97X-D/6–31G* model, removing conformers with energies >10 kJ/mol above the global minimum. Optimized geometries were verified as true minima on the potential energy surface by harmonic vibrational frequency calculations, which showed no imaginary frequencies; (5) single-point energy calculations with ωB97X-V/6–311+G­(2df,2p)­[6–311G*]; and (6) calculation of NMR chemical shifts using the GIAO method at the ωB97X-D/6–31G* level. The latter were averaged according to a Boltzmann distribution based on conformer energies and corrected empirically using a data set of ∼ 2000 rigid molecules,^22^ These corrections are in the order of 1–3 ppm.

The chemical shifts of olefinic carbons C-1 and C-8 of the xanthone core, and C-1′ and C-2′ of the pyran moiety, were further adjusted using empirical correction factors derived from statistical comparison between calculated and experimental values for a seventy-six-member training set of pyranoxanthones selected for high-quality ^13^C NMR data. Statistical analyses were performed using the SIMFIT program (for details see Supporting Information, Section IV).

## Supplementary Material



## Data Availability

The data underlying this study are available in the published article and its Supporting Information

## Notes

Examples of chemical shift search in NAPROC-13. Cartesian coordinates for optimized structures, computed chemical shifts, and deviations between calculated and experimental values (max absolute and rms terms) for compounds used as a modeling set. Statistical analysis to obtain empirical correction factors. Tables of experimental chemical shifts of the revised structures. ^13^C NMR data experimental and calculated and the Cartesian coordinates of the revised pyranoxantones (PDF)

## Abbreviations

rmsd: root mean square deviation
max dev: maximum absolute deviation
