# Supplementary material for: Structure Revision of Pyranoxanthones via DFT-Assisted 13C NMR Analysis and NAPROC-13 Platform: Diagnostic Markers and Discovery of Unreported Natural Products
Source: J Org Chem. 2025 Nov 3;90(49):17240–51. doi: 10.1021/acs.joc.5c01371 (PMC12706791; doi:10.1021/acs.joc.5c01371)
Supplement: Supplementary file 1 [file jo5c01371_si_001.pdf]

Supporting Information

Structure Revision of Pyranoxanthones via DFT-Assisted ^{13}C NMR Analysis and NAPROC-13 platform: Diagnostic Markers and Discovery of Unreported Natural Products

Hugo A. Sánchez-Martínez,[†] Juan A. Morán-Pinzón,[†] Esther del Olmo,[‡] José F. Adserias Vistué,[§] Estela Guerrero De León[†,] and José L. López-Pérez[†,‡,*]*

[†] CIPFAR. Departamento de Farmacología. Facultad de Medicina. Universidad de Panamá. Ave. Octavio Mendez Pereira, 0824-Panama City, Panama

[‡] Departamento de Ciencias Farmacéuticas, Área de Química Farmacéutica, Facultad de Farmacia, CIETUS, IBSAL. Campus Miguel de Unamuno, University of Salamanca, 37007-Salamanca, Spain.

[§] Departamento de Sistemas, Fundación General, University of Salamanca, Fonseca 2, 37002-Salamanca, Spain.

INDEX

Supporting Information S1

I. Searches in NAPROC-13 by name, chemical shifts, and substructure. S8

II. Pyranoxanthones selected as a modeling group; structures have been validated by DFT ^{13}C NMR calculation. S10

II.1 Figure SI-6.1. Pyranoxanthones selected as a modeling group. A S10

II.2. Figure SI-6.2. Structures of Xanthones selected as selected as a modeling group (continue). S11

III. Table S-1. Differences between Calculated and Experimental ^{13}C NMR Chemical Shifts of C-1 and C-8 Xanthone Core and Olefinic Carbons in the Pyran Moiety of 76 Pyranoxanthones selected as a Modeling Set. The structures of the Modeling Set are depicted in SI-2.1 and SI-2.II, Figures SI-6.2 and SI-6.3 ....S12

IV. Statistical analysis to obtain empirical correction factors for C-1, C-8, C-1' and C-2' carbons. S22

Figure SI-7. Box plots depicting the differences between calculated and experimental ^{13}C NMR chemical shifts for the C-1 and C-8 together, C-1' and C-2' in pyranoxanthones. S22

Table SI-2.1. Differences between calculated and experimental ^{13}C NMR chemical shifts for the C-1 & C-8. ....S23

Table SI-2.2. Differences between calculated and experimental ^{13}C NMR chemical shifts for the C-1'. S23

Table SI-2.3. Differences between calculated and experimental ^{13}C NMR chemical shifts for the C-2'. S23

V. Experimental and Calculated ^{13}C NMR Data and Cartesian Coordinates of the Global Minimum Conformers of the Compounds Shown in Figures SI-4.1 and SI-4.2 Used as the Modeling Set S24

V.1. 3,3-Dimethylpyrano[3,2-a]xanthen-12(3H)-one (A20-1) S24

V.2. Toxyloxanthone E (A20-2). S25

V.3. Thwaitesixanthone (A20-3–B31-17). S26

V.4. Caledonixanthone M (A20-4). S27

V.5. Cudraxanthone A (A20-5–D31-7). S29

V.6. Batukinaxanthone (A20-6). S30

V.7. Toxyloxanthone B (A23-1). S32

V.8. Elliptoxanthone B (A23-2). S33

V.9. Cudraxanthone B (A23-3). S34

V.10. Cudraxanthone K (A23-4). S35

V.11. Cudracuspixanthone P (A23-5). S37

V.12. Garcinone B (A23-6). S38

V.13. Allanxanthone B (A23-8). S39

V.14. Brasilixanthone B (A23-9–B31-18). S41

V.15. Brasilixanthone A (A23-11–D31-8). S43

V.16. Nigrolineaxanthone W (A23-12–B31-21). Also call Dulcisxanthone D and Tovophyllin B S44
V.17. Tovophyllin A (A23-13). S46
V.18. Cycloisoemericellin (B10-1). S47
V.19. 10-Methoxy-2,2,6,6-tetramethyl-2H,6H,14H-dipyrano[2,3-a:2',3'-c]xanthen-14-one (B13-1–D31-18). S49
V.20. Garciosone E (B13-2). S50
V.21. Osajaxanthone (B31-2). S51
V.22. 6-Deoxyjacareubin (B31-3). S52
V.23. Jacareubin (B31-4). S54
V.24. Nigrolineaxanthone K (B31-6). S55
V.25. Garcimangosxanthone E (B31-7). S56
V.26. Trapezifolixanthone (B31-8). Also call Toxyloxanthone A. S58
V.27. Inoxanthone (B31-9). Also call blancoxanthone[2] and caloxanthone C S59
V.28. 3-Hydroxyblancoxanthone, also call macluraxanthone[9] (B31-10) S60
V.30. Pyranojacareubin (B31-15–B34-6). S63
V.31. Nigrolineaxanthone I (B31-16–D32-4). S64
V.32. Mangostenone A (B31-19–D32-5). S66
V.33. 12-Hydroxy-2,2-dimethylpyrano[3,2-b]xanthen-6(2H)-one (B34-1). S67
V.34. 7,9,12-Trihydroxy-2,2-dimethyl-2H,6H-pyrano[3,2-b]xanthen-6-one (B34-2). S68
V.35. Garcinenone A (B34-3). S70
V.36. Subelliptenone H (B34-4). S71
V.37. Pedunxanthone C (B34-5). S72
V.38. Rheediaxanthone A (B34-8–D31-9). S73
V.39. Mesuarianone (B34-10–D31-10). S75
V.40. 12-Hydroxy-2,2-dimethylpyrano[2,3-b]xanthen-11(2H)-one (C21-1). S77
V.41. 12-Methoxy-2,2-dimethylpyrano[2,3-b]xanthen-11(2H)-one (C21-2). S78
V.42. 5-Hydroxy-2,2-dimethylpyrano[2,3-b]xanthen-11(2H)-one (C24-1). S79
V.43. 5-Methoxy-2,2-dimethylpyrano[2,3-b]xanthen-11(2H)-one (C24-2). S80
V.44. 8-Hydroxy-5-methoxy-2,2-dimethylpyrano[2,3-b]xanthen-11(2H)-one (C24-3). S81
V.45. 5,9-Dimethoxy-2,2-dimethylpyrano[2,3-b]xanthen-11(2H)-one (C24-4). S82
V.46. 5-Methoxy-2,2,9-trimethylpyrano[2,3-b]xanthen-11(2H)-one (C24-5). S83
V.47. 9-Chloro-5-methoxy-2,2-dimethylpyrano[2,3-b]xanthen-11(2H)-one (C24-6). S85
V.48. 8-(Diethylamino)-5-methoxy-2,2-dimethylpyrano[2,3-b]xanthen-11(2H)-one (C24-7). S86
V.49. Caledonixanthone B (C40-1). S87
V.50. 5-((3-Methylpent-1-yn-3-yl)oxy)-4a,9a-dihydro-9H-xanthen-9-one (C40-2). S88
V.51. Dehydrocycloguanandin (C40-3). S89
V.52. 8,11-Dihydroxy-2,2-dimethylpyrano[3,2-c]xanthen-7(2H)-one (C40-4). S90
V.53. Garciniaxanthone B (C40-5). S91

V.54. 6-Hydroxy-2,2-dimethylpyrano[3,2-c]xanthen-7(2H)-one (C41-1).....S93
V.55. 6-Methoxy-2,2-dimethylpyrano[3,2-c]xanthen-7(2H)-one (C41-2). S94
V.56. Dulcisxanthone H (C41-3). S95
V.57. Methyl 3,3-dimethyl-7-oxo-3H,7H-pyrano[2,3-c]xanthene-11-acetate (D30-1). S96
V.58. 3,3-Dimethyl-7-oxo-3H,7H-pyrano[2,3-c]xanthene-11-acetic acid (D30-2). S98
V.59. 11-Methoxy-3,3-dimethyl-3H,7H-pyrano[2,3-c]xanthen-7-one (D30-3). S99
V.60. 8,11-Dihydroxy-3,3-dimethyl-3H,7H-pyrano[2,3-c]xanthen-7-one (D30-4).....S100
V.61. 8,11-Dihydroxy-3,3,10-trimethyl-3H,7H-pyrano[2,3-c]xanthen-7-one (D30-5).....S101
V.62. 8,11-Dihydroxy-3,3,9-trimethyl-3H,7H-pyrano[2,3-c]xanthen-7-one (D30-6).....S102
V.63. 6-Hydroxy-3,3-dimethyl-3H,7H-pyrano[2,3-c]xanthen-7-one (D31-1). S103
V.64. 6-Methoxy-3,3-dimethyl-3H,7H-pyrano[2,3-c]xanthen-7-one (D31-2).....S104
V.65. Nigrolineaxanthone F (D31-3). S106
V.66. 6-Deoxyi-5-O-methylsojacareubin (D31-4).....S107
V.67. Isojacareubin (D31-5). S108
V.68. di-O-Methyllorostemin (D31-6). S109
V.69. 6,10,11-Trihydroxy-8-(3-hydroxy-3-methylbutyl)-3,3-dimethyl-9-(3-methyl-2-buten-1-yl)-3H,7H-pyrano[2,3-c] xanthen-7-one (D31-11). S110
V.70. Cudratricusxanthone H (D31-12). S112
V.71. Nigrolineaxanthone H (D32-1). S113
V.72. Oblongixanthone A (D32-2).....S114
V.73. Isocudraxanthone K (D32-3).....S115
V.74. Mangostenone A (D32-6). S117
V.75. Cudratrixanthone J (D32-7). S118
V.76. Caledonixanthone E (D32-8).....S120

VI-1. Pyranoxanthones whose structures have been revised in this study and validated by DFT ^{13}C NMR calculations. S122

VI-2. Pyranoxanthones whose structures have been revised in this study and validated by DFT ^{13}C NMR calculations. S123

VII. 13C NMR chemical shifts (δ, ppm) of revised pyranoxanthones compared with reference compounds (shaded columns). Chemical shifts are listed in decreasing order. Multiplicities (mult.) are indicated.. .....S124

VII.1. Table SI-2. The ^{13}C NMR chemical shifts of the revised pyranoxanthone structures, along with the corresponding data for the reference compounds. S124
VII.2. Table SI-2. The ^{13}C NMR chemical shifts of the revised pyranoxanthone structures, along with the corresponding data for the reference compounds (continue).....S125
VII.3. Table SI-2. The ^{13}C NMR chemical shifts of the revised pyranoxanthone structures, along with the corresponding data for the reference compounds (continue).....S126
VII.4. Table SI-2. The ^{13}C NMR chemical shifts of the revised pyranoxanthone structures, along with the corresponding data for the reference compounds (continue).....S127

VII.5. Table SI-2. The 13C NMR chemical shifts of the revised pyranoxanthone structures, along with the corresponding data for the reference compounds (continue). S128

VII.6. Table SI-2. The ^{13}C NMR chemical shifts of the revised pyranoxanthone structures, along with the corresponding data for the reference compounds (continue). S129

VIII. ^{13}C NMR data experimental and calculated and the cartesian coordinates of the global minimum conformer of the revised pyranoxantones. S130

VIII.1. Dulcisxanthone A (R1–A20) and its revised structure garcinone B (A23-6) S130

VIII.1a. Dulcisxanthone A (R1–A20) S130

VIII.1b. Garcinone B (A23-6). S131

VIII.2. Globulixanthone B (R2–A24) and its revised structure (A23-7). S132

VIII.2a. Globulixanthone B (R2–A24) S132

VIII.2b. A23-7 (Unreported natural product) S134

VIII.3. Cudracuspixanthone G (R3–A24) and its revised structure (A23-10–B34-9) S135

VIII.3a. Cudracuspixanthone G (R3–A24) S135

VIII.3b. A23-10–B34-9 (Unreported natural product) S136

VIII.4. Inophinone (R4–B13) and its revised structure calophinone (A23-14–B31-20) S138

VIII.4a. Inophinone (R4–B13). S138

VIII.4b. Calophinone (A23-14–B31-20) S140

VIII.5. 3-(1,1-Dimethyl-2-propen-1-yl)-5,9,11-trihydroxy-2,2-dimethyl-2H,12H-pyrano[2,3-a]xanthen -12-one (R5–B13), and its revised structure calotetrapterin D (A23-15). S141

VIII.5a. 3-(1,1-Dimethyl-2-propen-1-yl)-5,9,11-trihydroxy-2,2-dimethyl-2H,12H-pyrano[2,3-a]xanthen-12-one (R5–B13) S141

VIII.5b. Calotetrapterin D (A23-15). S143

VIII.6. 3-(1,1-Dimethyl-2-propen-1-yl)-5,9,11-trihydroxy-2,2-dimethyl-10-(3-methyl-2-buten-1-yl)-2H,12H-pyrano[2,3-a]xanthen-12-one (R6–B13) and its revised structure calotetrapterin A (A23-17). S144

VIII.6a. 3-(1,1-Dimethyl-2-propen-1-yl)-5,9,11-trihydroxy-2,2-dimethyl-10-(3-methyl-2-buten-1-yl)-2H,12H-pyrano[2,3-a]xanthen-12-one (R6–B13) S144

VIII.6b. Calotetrapterin A (A23-17) S146

VIII.7. 5,9,11-trihydroxy-10-(2-hydroxy-3-methylbut-3-en-1-yl)-3,3-dimethyl-2-(2-methylbut-3-en-2-yl)pyrano[3,2-a]xanthen-12(3H)-one (R7–B13) and its revised structure calotetrapterin E (A23-16). S148

VIII.7a. 5,9,11-trihydroxy-10-(2-hydroxy-3-methylbut-3-en-1-yl)-3,3-dimethyl-2-(2-methylbut-3-en-2-yl)pyrano[3,2-*a*]xanthen-12(*3H*)-one (R7–B13) S148

VIII.7b. Calotetrapterin E (A23-16) S150

VIII.8. 1-isomangostin (R8–B13) and its revised structure 3-isomangostin (B31-13). S151

VIII.8a. 1-isomangostin (R8–B13) S152

VIII.8b. 3-Isomangostin (B31-13). S153

VIII.9. Hypejaponol B (R9–B13) and its revised structure (D31-14) (Unreported natura product). S155

VIII.9a. Hypejaponol B (R9–B13) S155

VIII.9b. D31-14 (Unreported natural product) S157

VIII.10. 2H,6H-Pyrano[3,2-b]xanthen-6-one,12-(1,1-dimethyl-2-propen-1-yl)-5,9,10-trihydroxy-2,2-dimethyl (R10–B31)[5] and its revised structure pedunxanthone C demethoxylated (B34-7). S158

VIII.10a. 2H,6H-Pyrano[3,2-b]xanthen-6-one,12-(1,1-dimethyl-2-propen-1-yl)-5,9,10-trihydroxy-2,2-dimethyl (R10–B31) S159

VIII.10b. Dedunxanthone C demethoxylated (B34-7). S160
VIII.11. Nigrolineaxanthone G (R11–B34) and its revised structure (D32-9). S161
VIII.11a. Nigrolineaxanthone G (R11–B34)....S161
VIII.11b. D32-9 (Unreported natural product) S163
VIII.12. Soulattrin (R12–B34) and its revised structure macluraxanthone (B31-10). S164
VIII.12a. Soulattrin (R12–B34) S164
VIII.12b. Macluraxanthone (B31-10). S166
VIII.13. 1,3,3-Trihydroxy-6',6'-dimethylpyrano(2',3':6,7)-4-(1,1-dimethylprop-2-enyl)-xanthone (R13–B34), and its revised structure macluraxanthone (B31-10). S166
VIII.13a. 1,3,3-Trihydroxy-6',6'-dimethylpyrano(2',3':6,7)-4-(1,1-dimethylprop-2-enyl)-xanthone (R13–B34)....S166
VIII.13b. Macluraxanthone (B31-10). S168
VIII.14. Mesuaferrin A (R14–C21) and its revised structure macluraxanthone (B31-10) S169
VIII.14a. Mesuaferrin A (R14–C21) S169
VIII.14b. Macluraxanthone (B31-10). S170
VIII.15. Inophyllin B (R15–D31) and its revised structure macluraxanthone (B31-10). S171
VIII.15a. Inophyllin B (R15–D31) S171
VIII.15b. Macluraxanthone (B31-10). S172
VIII.16. Sterigmatin (analogo) (R16–C21a) and its revised structure demethylsterigmatocystin (D31-16). S173
VIII.16b. Sterigmatin (R16–C21b) S174
VIII.16c. Demethylsterigmatocystin (D31-16). S175
VIII.17. Garcimangosone A (R17–C24) and its revised structure mangostenone A (B31-19–D32-5). S176
VIII.17a. Garcimangosone A (R17–C24) S176
VIII.17b. Mangostenone A (B31-19–D32-5) S178
VIII.18. Venuloxanthone (R18–C40) and its revised structure Thwaitesixanthone (A20-3–B31-17). S178
VIII.18a. Venuloxanthone (R18–C40) S178
VIII.18b. Thwaitesixanthone (A20-3–B31-17) S180
VIII.19. Tetrandraxanthone (R19–C40) and its revised structure toxyloxanthone E (A20-2). S180
VIII.19a. Tetrandraxanthone (R19–C40)....S180
VIII.19b. Toxyloxanthone E (A20-2)....S181
VIII.20. Mesuaferrin B (R20–C41) and its revised structure pyranojacareubin (B31-15–B34-6). S182
VIII.20a. Mesuaferrin B (R20–C41) S182
VIII.20b. Pyranojacareubin (B31-15–B34-6). S183
VIII.20c. Rheediaxanthone A (B34-8–D31-9). S184
VIII.21. 6,8,10-Trihydroxy-2,2-dimethylpyrano[3,2-*c*]xanthen-7(2*H*)-one (R21–C41) and its revised structure 7,9,12-trihydroxy-2,2-dimethyl-2*H*,6*H*-pyrano[3,2-*b*]xanthen-6-one (B34-2). S185
VIII.21a. 6,8,10-Trihydroxy-2,2-dimethylpyrano[3,2-c]xanthen-7(2H)-one (R21–C41) S185
VIII.21b. 7,9,12-Trihydroxy-2,2-dimethyl-2H,6H-pyrano[3,2-b]xanthen-6-one (B34-2) S186
VIII.22-1. 6-Hydroxy-3,3-dimethyl-3H,7H-pyrano[2,3-c]xanthen-7-one (R22b–D31) and its revised structure 5-Hydroxy-2,2-dimethyl-2H,6H-pyrano[3,2-b]xanthen-6-one (B31-1b). S187
VIII.22-1a. 6-Hydroxy-3,3-dimethyl-3H,7H-pyrano[2,3-c]xanthen-7-one (R22b–D31) S187
VIII.22-1b. 5-Hydroxy-2,2-dimethyl-2H,6H-pyrano[3,2-b]xanthen-6-one (B31-1b) S188

VIII.22-2. 6-Hydroxy-3-methyl-3*H*,7*H*-pyrano[2,3-*c*]xanthen-7-one (R22a–D31) and its revised structure (B31-1a) S189
VIII.22-2a. 6-Hydroxy-3-methyl-3*H*,7*H*-pyrano[2,3-*c*]xanthen-7-one (R22a–D31) S189
VIII.22-2b. B-31-1a (Previously unreported product) S190
VIII.22-3. 6-Methoxy-3,3-dimethyl-3*H*,7*H*-pyrano[2,3-*c*]xanthen-7-one (R21i–D31) and its revised structure 5-Methoxy-2,2-dimethyl-2*H*,6*H*-pyrano[3,2-*b*]xanthen-6-one (B31-1i). S191
VIII.22-3a. 6-Methoxy-3,3-dimethyl-3*H*,7*H*-pyrano[2,3-*c*]xanthen-7-one (R22i–D31) S191
VIII.22-3b. 5-Methoxy-2,2-dimethyl-2*H*,6*H*-pyrano[3,2-*b*]xanthen-6-one (B31-1i). S192
VIII.23. Dehydrocycloguanandin B (R23–D31) and its revised structure 6-deoxyjacareubin (B31-3). S193
VIII.23a. Dehydrocycloguanandin B (R23–D31) S193
VIII.23b. 6-Deoxyjacareubin (B31-3) S194
VIII.24. Globulixanthone C (R24–D31) and its revised structure (B31-5) S195
VIII.24a. Globulixanthone C (R24–D31) S195
VIII.24b. B31-5 (Unreported natural product) S196
VIII.25. inophyllin A (R25–D31) and its revised structure inoxanthone also call blancoxanthone (B31-9). S197
VIII.25a. Inophyllin A (R25–D31) S197
VIII.25b. Inoxanthone also call blancoxanthone (B31-9). S199
VIII.26. Buxixanthone (R26–D31) and its revised structure calabaxanthone (B31-12). S199
VIII.26a. Buxixanthone (R26–D31) S200
VIII.26b. B31-12a S201
VIII.26c. Calabaxanthone (B31-12). S203
VIII.27. Musaxanthone (R27–D31) and its revised structure garciosone E (B13-2). S204
VIII.27a. Musaxanthone (R27–D31) S204
VIII.27b. Garciosone E (B13-2). S205
VIII.28. Asmaxanthone (R28–D31) and its revised structure (B13-3) (unreported natural product) S206
VIII.28a. Asmaxanthone (R28–D31). S206
VIII.28b. B13-3 (Unreported natural product) S207
VIII.29. Nigrolineaxanthone X (R29–D31) and its revised structure morusignin H (D31-17). S209
VIII. 29a. Nigrolineaxanthone X (R29–D31) S209
VIII.29b. Morusignin H (D31-17). S210
VIII.30. Neriifolone C (R30–D31) and its revised structure pruniflorone N (D31-18). S212
VIII.30a. Neriifolone C (R30–D31) S212
VIII.30b. Pruniflorone N (D31-18) S213
VIII.31. 1,3,7-Trihydroxy-4-(1,1-dimethyl-2-propenyl)-5,6-(2,2-dimethylchromeno)-xanthone (R31–D32) and its revised structure cudraxanthone B (A23-3). S214
VIII.31a. 1,3,7-Trihydroxy-4-(1,1-dimethyl-2-propenyl)-5,6-(2,2-dimethylchromeno)-xanthone (R31–D32) S214
VIII.31b. Cudraxanthone B (A23-3). S216
Bibliography .... S**Error! Bookmark not defined.**

I. Searches in NAPROC-13 by name, chemical shifts, and substructure.

NAPROC-13 is a freely accessible and searchable database at https://c13.usal.es, that collects structural and ^{13}C NMR spectral information. NAPROC-13 contains information regarding 1,500 xanthones.

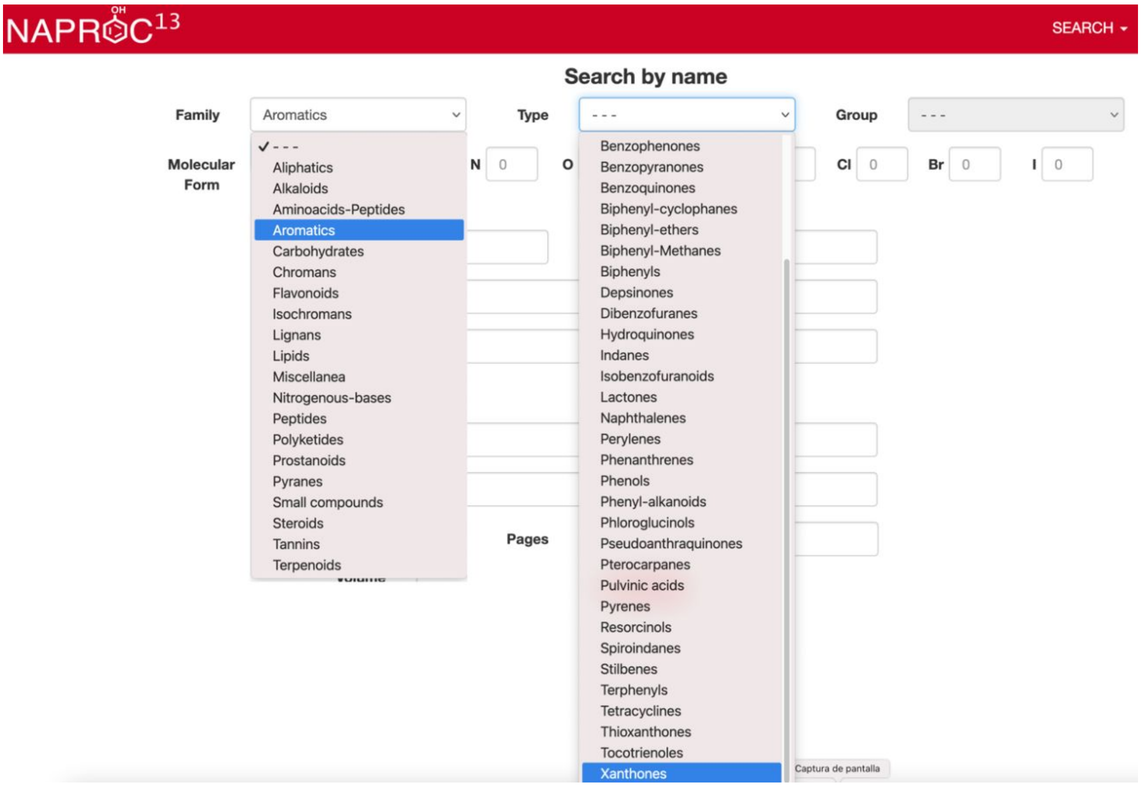

Figure SI-1. Search by family and type of compounds in NAPROC-13.

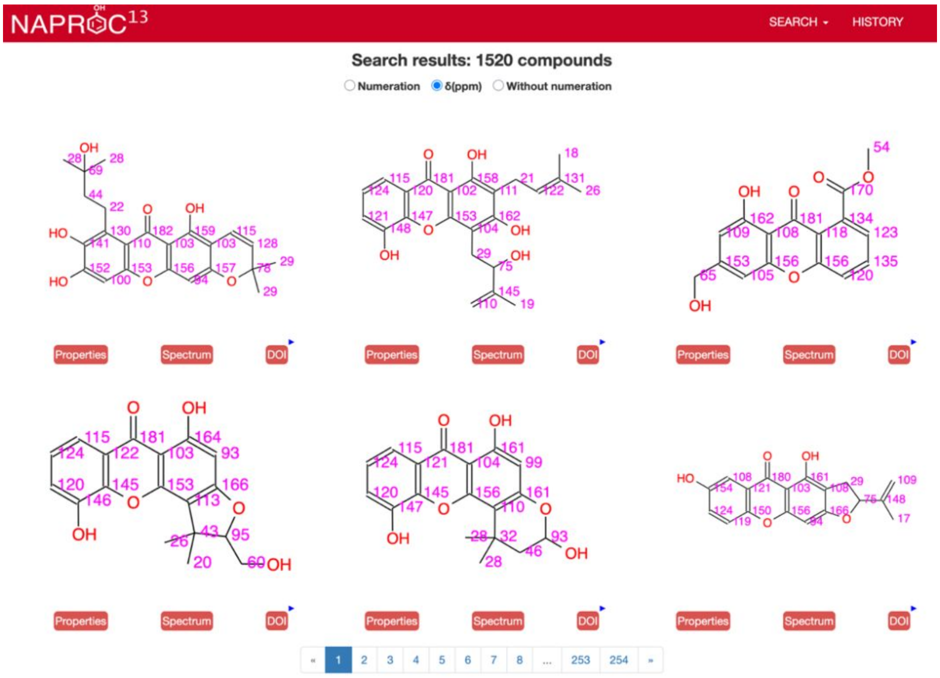

Figure SI-2. Xanthone structures retrieved from the NAPROC-13 database using a search filtered by family ("Aromatics") and type ("Xanthones"). ^{13}C NMR chemical shift values (δ, ppm) for each carbon atom are shown in magenta. Additional information, including properties, spectra, and DOIs, is available through the database interface.

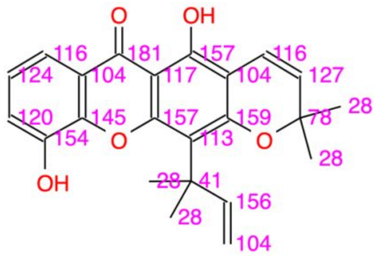 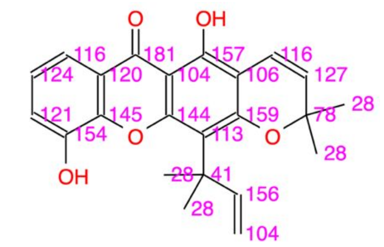 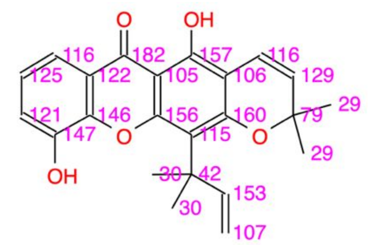

Inoxanthone[1] (**B31-9**) ($CDCl_3$) Blancoxanthone[2] (**B31-9**) ($CDCl_3$) Caloxanthone C[3] (**B31-9**) (Acetone d_6)

Figure SI-3. ^{13}C RMN data of inoxanthone[1] (also known as blancoxanthone[2] or caloxanthone C[3]) recorded in $CDCl_3$ and acetone d_6. Differences in chemical shifts are observed between the spectra. Bibliographic references are indicated in superscript after the name of the xanthone.

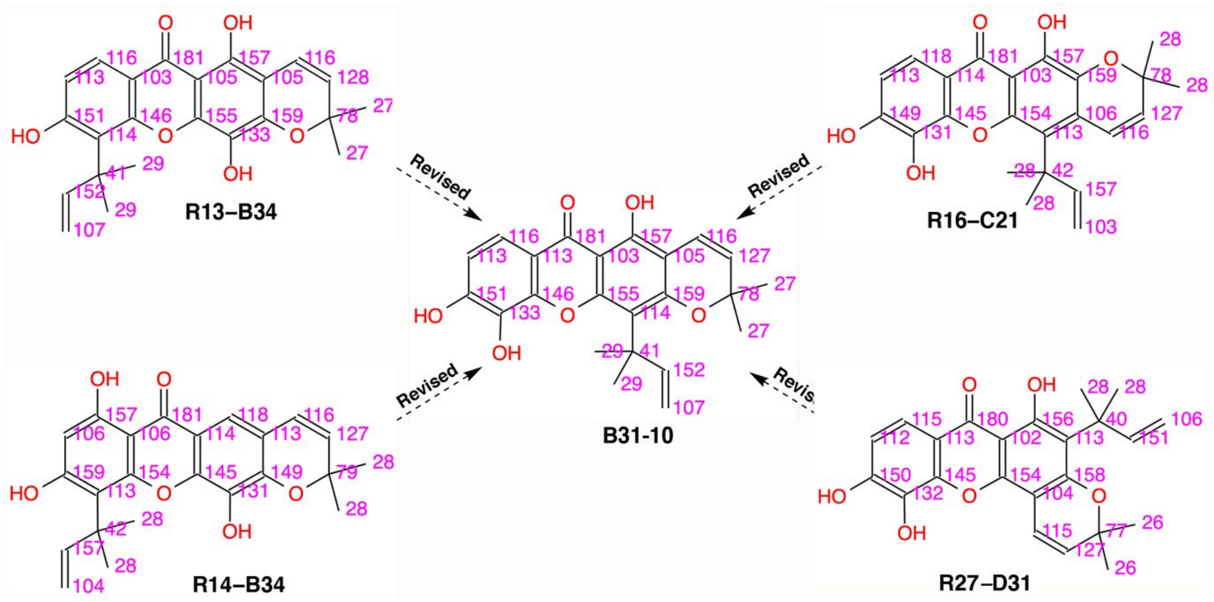

Figure SI-4. Utility of NAPROC-13 to identify misassigned compounds through ^{13}C NMR chemical shift searches. A chemical shift search identified five different pyranoxanthones—soulattrin[4] (**R13–B34**), 1,3,5-Trihydroxy-6',6'-dimethylpyrano(2',3':6,7)-4-(1,1-dimethylprop-2-enyl)-xanthone[5] (**R14–B34**), mesuaferrin A[6] (**R16–C21**), inophyllin B B[7, 8] (**R27–D31**), and 3-hydroxyblancoxanthone[2] (**B31-10**)—sharing the same ^{13}C NMR data set. The first four structures are misassignments, whereas *3-hydroxyblancoxanthone* also known as macluraxanthone (**B31-10**) represents the correct structure.

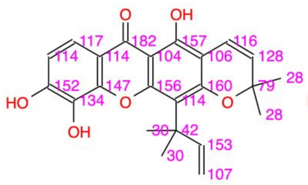 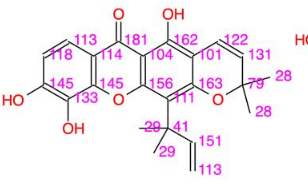 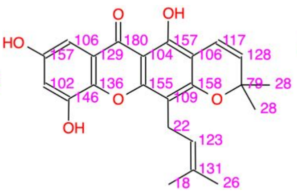 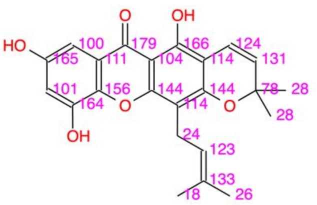

Macluraxanthone[9] (A) Macluraxanthone[5] (B) Staudtiixanthone B[10] (C) Baphikixanthone B[11] (D)

Figure SI-5. Two pairs of pyranoxanthones (A, B) and (C, D) resulting from two independent substructure searches in NAPROC-13. The first pair corresponds to macluraxanthone (**B31-10**), but with significantly different reported ^{13}C NMR data. The second pair involves staudtiixanthone B and baphikixanthone B (**R36–B31**), which also share identical structures but display divergent ^{13}C NMR datasets. In both instances, one of the proposed structures is clearly misassigned.

II. Pyranoxanthones selected as a modeling group; structures have been validated by DFT ^{13}C NMR calculation.

II.1 Figure SI-6.1. Pyranoxanthones selected as a modeling group. A *code is shown below each structure, indicating the key used throughout the entire publication.*

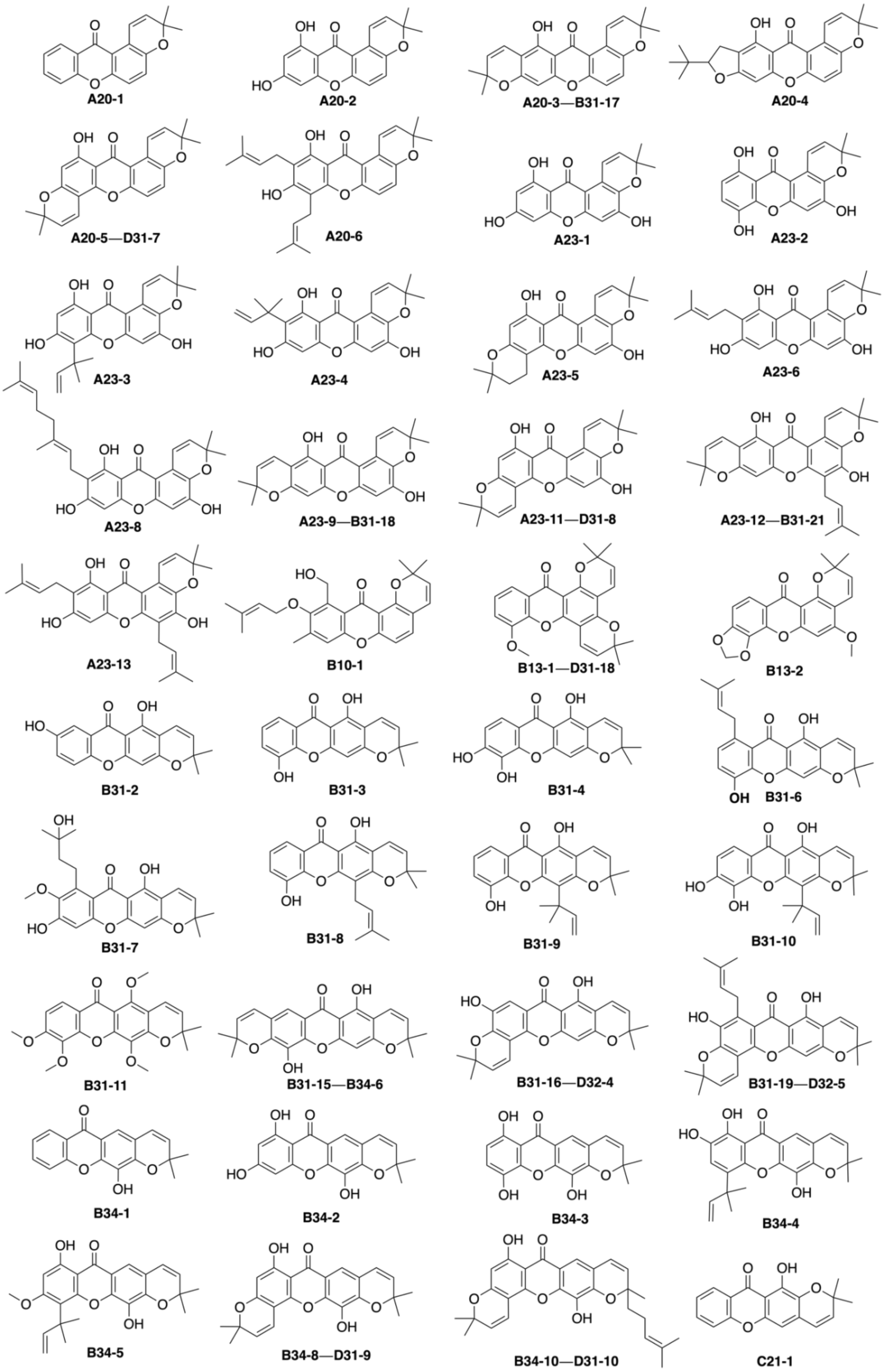

II.2. Figure SI-6.2. Structures of Xanthones selected as selected as a modeling group (continue). *A code is shown below each structure, indicating the key used throughout the entire publication.*

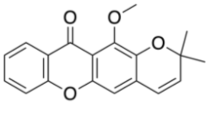
C21-2

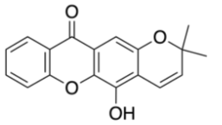
C24-1

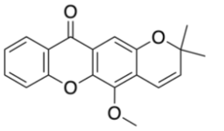
C24-2

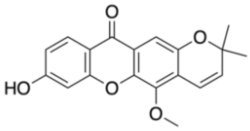
C24-3

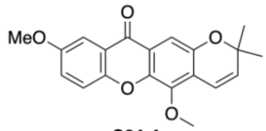
C24-4

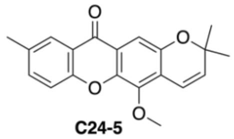
C24-5

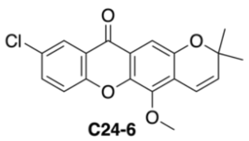
C24-6

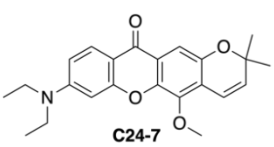
C24-7

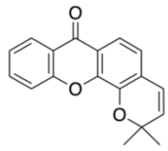
C40-1

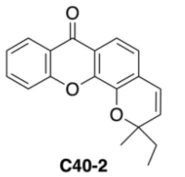
C40-2

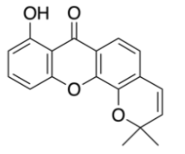
C40-3

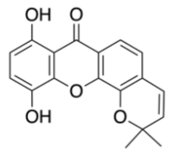
C40-4

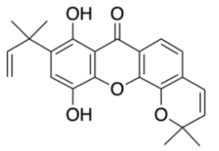
C40-5

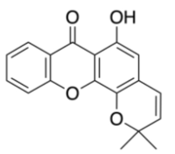
C41-1

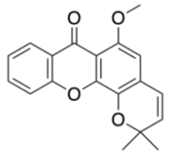
C41-2

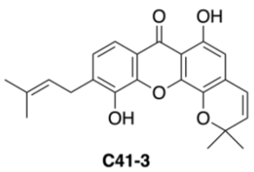
C41-3

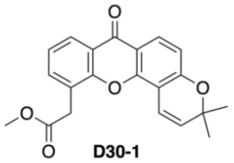
D30-1

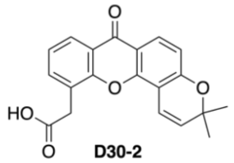
D30-2

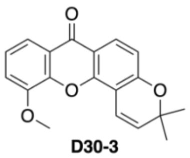
D30-3

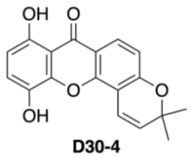
D30-4

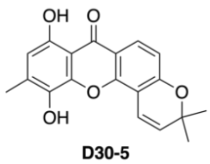
D30-5

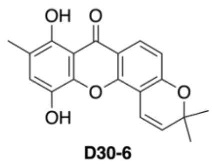
D30-6

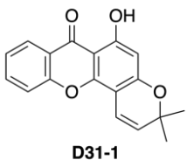
D31-1

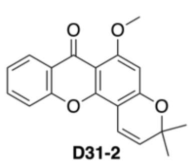
D31-2

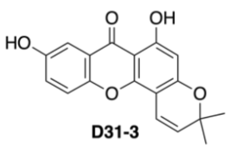
D31-3

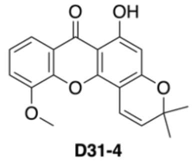
D31-4

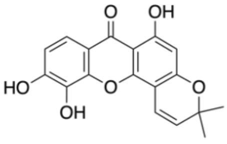
D31-5

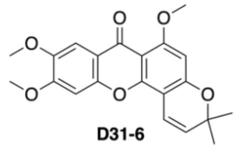
D31-6

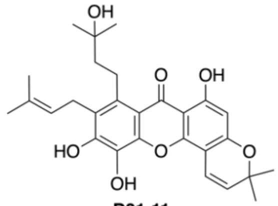
D31-11

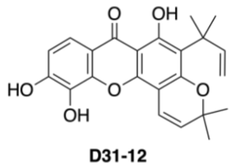
D31-12

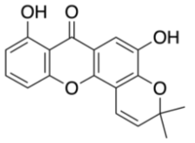
D32-1

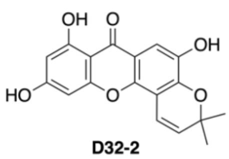
D32-2

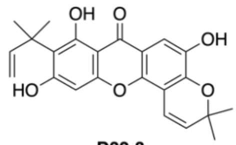
D32-3

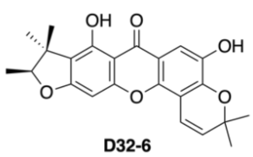
D32-6

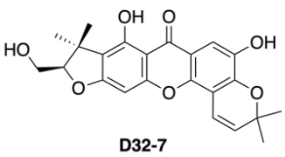
D32-7

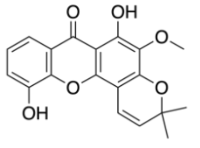
D32-8

III. Table S-1. Differences between Calculated and Experimental ^{13}C NMR Chemical Shifts of C-1 and C-8 Xanthone Core and Olefinic Carbons in the Pyran Moiety of 76 Pyranoxanthones selected as a Modeling Set. The structures of the Modeling Set are depicted in SI-2.1 and SI-2.II, Figures SI-6.2 and SI-6.3

SI number	Code	Category	Subcategory	C-1 and C8	δ_{calc}	δ_{exp}	δ_{Calc}-δ_{exp}	C pyran	δ_{calc}	δ_{exp}	δ_{Calc}-δ_{exp}
1	A20-1[12]	A	A20	C-1	124.1	124.1	0.0	C-1'	124.5	120.9	3.6
1	A20-1[12]	A	A20	C-8	130.0	126.6	3.4	C-2'	131.9	132.5	-0.6
2	A20-2[13]	A	A20	C-1	124.1	120.2	3.9	C-1'	124.3	120.9	3.4
2	A20-2[13]	A	A20	C-8	166.0	164.1	1.9	C-2'	132.3	133.0	-0.7
3	**A20-3**–B31-17[14]	A	A20	C-1	160.9	158.0	2.9	C-1''	124.3	120.8	3.5
3	**A20-3**–B31-17[14]	A	A20	C-8	123.8	118.1	5.7	C-2''	132.0	132.6	-0.6
4	A20-4[15]	A	A20	C-1	160.4	157.9	2.5	C-1'	124.3	119.8	4.5
4	A20-4[15]	A	A20	C-8	123.7	120.8	2.9	C-2'	131.8	132.7	-0.8
5	**A20-5**–D31-7[16]	A	A20	C-1	165.7	163.2	2.5	C-1''	124.2	120.7	3.5
5	**A20-5**–D31-7[16]	A	A20	C-8	124.0	119.9	4.1	C-2''	132.1	132.7	-0.6
5	**A20-5**–D31-7[17]	A	A20	C-1	165.7	163.4	2.3	C-1''	124.2	120.8	3.4
5	**A20-5**–D31-7[17]	A	A20	C-8	124.0	119.0	5.0	C-2''	132.1	132.8	-0.7
6	A20-6[18]	A	A20	C-1	160.5	158.4	2.1	C-1'	124.6	121.8	2.8
6	A20-6[18]	A	A20	C-8	123.7	120.9	2.8	C-2'	131.6	132.4	-0.8
7	A23-1[19]	A	A23	C-1	165.9	165.1	0.8	C-1'	124.9	121.1	3.8
7	A23-1[19]	A	A23	C-8	124.0	120.5	3.5	C-2'	131.4	133.2	-1.8
7	A23-1[20]	A	A23	C-1	165.9	164.4	1.5	C-1'	124.9	121.5	3.4
7	A23-1[20]	A	A23	C-8	124.0	120.5	3.5	C-2'	131.4	133.6	-2.2
8	A23-2[21]	A	A23	C-1	155.5	153.9	1.6	C-1'	124.8	120.6	4.2
8	A23-2[21]	A	A23	C-8	125.1	120.1	5.0	C-2'	132.4	133.0	-0.6

SI number	Code	Category	Subcategory	C-1 and C8	δ_{calc}	δ_{exp}	δ_{Calc}-δ_{exp}	C pyran	δ_{calc}	δ_{exp}	δ_{Calc}-δ_{exp}
9	A23-3[17]	A	A23	C-1	164.6	162.0	2.6	164.6	162.0	2.6	4.2
9	A23-3[17]	A	A23	C-8	124.0	119.6	4.4	124.0	119.6	4.4	-1.1
10	A23-4[17]	A	A23	C-1	164.8	163.3	1.5	164.8	163.3	1.5	4.2
10	A23-4[17]	A	A23	C-8	124.1	119.8	4.3	124.1	119.8	4.3	-1.1
11	A23-5[22]	A	A23	C-1	163.5	161.0	2.5	163.5	161.0	2.5	4.6
11	A23-5[22]	A	A23	C-8	124.1	119.9	4.2	124.1	119.9	4.2	-1.6
12	A23-6[23]	A	A23	C-1	162.3	159.7	2.6	162.3	159.7	2.6	4.9
12	A23-6[23]	A	A23	C-8	124.0	119.7	4.3	124.0	119.7	4.3	-1.5
13	A23-8[24]	A	A23	C-1	162.2	161.5	0.7	162.2	161.5	0.7	3.2
13	A23-8[24]	A	A23	C-8	124.1	119.2	4.9	124.1	119.2	4.9	-0.5
14	**A23-9**–B31-18[25]	A	A23	C-1	160.7	159.6	1.1	160.7	159.6	1.1	4.0
14	**A23-9**–B31-18[25]	A	A23	C-8	124.0	121.5	2.5	124.0	121.5	2.5	-1.0
14	**A23-9**–B31-18[26]	A	A23	C-1	160.7	157.8	2.9	160.7	157.8	2.9	4.1
14	**A23-9**–B31-18[26]	A	A23	C-8	124.0	119.7	4.3	124.0	119.7	4.3	-1.0
15	**A23-11**–D31-8[25]	A	A23	C-1	165.5	163.3	2.2	165.5	163.3	2.2	3.8
15	**A23-11**–D31-8[25]	A	A23	C-8	124.3	120.0	4.3	124.3	120.0	4.3	-0.9
16	**A23-12**–B31-21[27]	A	A23	C-1	160.7	157.8	2.9	160.7	157.8	2.9	4.3
16	**A23-12**–B31-21[27]	A	A23	C-8	121.5	117.1	4.4	121.5	117.1	4.4	-1.0
16	**A23-12**–B31-21[28]	A	A23	C-1	160.7	157.7	3.0	160.7	157.7	3.0	4.4
16	**A23-12**–B31-21[28]	A	A23	C-8	121.5	117.1	4.4	121.5	117.1	4.4	-0.9
16	**A23-12**–B31-21[29]	A	A23	C-1	160.7	157.7	3.0	160.7	157.7	3.0	4.4
16	**A23-12**–B31-21[29]	A	A23	C-8	121.5	117.1	4.4	121.5	117.1	4.4	-0.9

SI number	Code	Category	Subcategory	C-1 and C8	δ_{calc}	δ_{exp}	δ_{Calc}-δ_{exp}	C pyran	δ_{calc}	δ_{exp}	δ_{Calc}-δ_{exp}
17	A23-13[30]	A	A23	C-1	162.2	160.1	2.1	C-1'	125.4	121.0	4.4
17	A23-13[30]	A	A23	C-8	121.5	117.1	4.4	C-2'	130.2	131.3	-1.1
18	B10-1[31]	B	B10	C-1	156.7	154.4	2.3	C-1'	124.0	120.0	4.0
18	B10-1[31]	B	B10	C-8	139.4	134.6	4.8	C-2'	128.4	129.4	-1.0
19	**B13-1**–D31-18[32]	B	B13	C-1	158.4	155.3	3.1	C-1'	119.9	115.7	4.2
19	**B13-1**–D31-18[32]	B	B13	C-8	120.2	117.6	2.6	C-2'	125.4	127.7	-2.3
20	B13-2[33]	B	B13	C-1	158.2	155.3	2.9	C-1'	119.8	115.8	4.0
20	B13-2[33]	B	B13	C-8	124.3	121.3	3.0	C-2'	125.4	127.6	-2.2
21	B31-2[34]	B	B31	C-1	160.4	157.0	3.4	C-1'	118.4	114.7	3.7
21	B31-2[34]	B	B31	C-8	110.4	108.2	2.2	C-2'	122.2	128.8	-6.6
21	B31-2[16]	B	B31	C-1	160.4	158.2	2.2	C-1'	118.4	115.6	3.6
21	B31-2[16]	B	B31	C-8	110.4	109.1	1.3	C-2'	122.2	129.1	-6.9
22	B31-3[26]	B	B31	C-1	160.9	157.7	3.2	C-1'	118.3	114.8	3.5
22	B31-3[26]	B	B31	C-8	118.2	115.4	2.8	C-2'	122.4	128.1	-5.7
22	B31-3[35]	B	B31	C-1	160.9	156.7	4.2	C-1'	118.3	114.4	3.9
22	B31-3[35]	B	B31	C-8	118.2	114.3	3.9	C-2'	122.4	128.4	-6.0
23	B31-4[36]	B	B31	C-1	161.0	156.7	4.3	C-1'	118.4	114.5	3.9
23	B31-4[36]	B	B31	C-8	121.4	116.0	5.4	C-2'	122.3	128.2	-5.9
24	B31-6[37]	B	B31	C-1	161.2	158.2	3.0	C-1'	120.0	115.5	4.5
24	B31-6[37]	B	B31	C-8	136.0	135.5	0.5	C-2'	123.5	127.5	-4.0
25	B31-7[38]	B	B31	C-1	160.7	157.8	2.9	C-1'	120.1	115.6	4.5
25	B31-7[38]	B	B31	C-8	143.4	142.6	0.8	C-2'	123.3	127.3	-4.0

SI number	Code	Category	Subcategory	C-1 and C8	δ_{calc}	δ_{exp}	δ_{Calc}-δ_{exp}	C pyran	δ_{calc}	δ_{exp}	δ_{Calc}-δ_{exp}
26	B31-8[39]	B	B31	C-1	159.4	156.1	3.3	C-1'	121.0	115.8	5.2
26	B31-8[39]	B	B31	C-8	118.2	116.9	1.3	C-2'	124.5	127.6	-3.1
27	B31-9[1]	B	B31	C-1	159.7	156.7	3.0	C-1'	121.1	116.0	5.1
27	B31-9[1]	B	B31	C-8	117.3	116.0	1.3	C-2'	123.9	127.3	-3.4
27	B31-9[2]	B	B31	C-1	159.7	156.7	3.0	C-1'	121.2	116.1	5.1
27	B31-9[2]	B	B31	C-8	117.3	116.0	1.3	C-2'	123.9	127.3	-3.4
27	B31-9[3]	B	B31	C-1	159.7	157.2	2.5	C-1'	121.2	116.3	4.9
27	B31-9[3]	B	B31	C-8	117.3	116.0	1.3	C-2'	123.9	128.6	-4.7
28	B31-10[2]	B	B31	C-1	159.9	156.5	3.4	C-1'	121.3	115.5	5.8
28	B31-10[2]	B	B31	C-8	120.8	116.2	4.6	C-2'	123.8	127.2	-3.4
28	B31-10[9]	B	B31	C-1	159.9	157.3	2.6	C-1'	121.3	116.4	4.9
28	B31-10[9]	B	B31	C-8	120.8	117.2	3.6	C-2'	123.8	128.2	-4.4
28	B31-10[40]	B	B31	C-1	159.9	157.7	2.2	C-1'	121.3	115.3	6.0
28	B31-10[40]	B	B31	C-8	120.8	114.9	5.9	C-2'	123.8	127.1	-3.3
29	B31-11[41]	B	B31	C-1	154.4	151.5	2.9	C-1'	121.1	116.2	4.9
29	B31-11[41]	B	B31	C-8	125.4	121.1	4.3	C-2'	127.3	130.1	-2.8
30	**B31-15**–B34-6[26]	B	B31	C-1	160.7	157.8	2.9	C-1'	120.7	115.5	5.2
30	B31-15–B34-6[26]	B	B31	C-8	116.2	113.6	2.6	C-2'	123.7	127.6	-3.9
31	**B31-16**–D32-4[42]	B	B31	C-1	160.6	157.6	3.0	C-1'	118.9	115.6	3.3
31	**B31-16**–D32-4[42]	B	B31	C-8	113.0	108.7	4.3	C-2'	122.1	127.4	-5.3
3	A20-3–**B31-17**[14]	B	B31					C-1'	120.6	115.5	5.1
3	A20-3–**B31-17**[14]	B	B31					C-2'	123.5	127.2	-3.7

SI number	Code	Category	Subcategory	C-1 and C8	δ_{calc}	δ_{exp}	δ_{Calc}-δ_{exp}	C pyran	δ_{calc}	δ_{exp}	δ_{Calc}-δ_{exp}

14	A23-9–**B31-18**[25]	B	B31					C-1'	120.8	115.7	5.1
14	A23-9–**B31-18**[25]	B	B31					C-2'	123.5	127.2	-3.7
14	A23-9–**B31-18**[26]	B	B31					C-1'	120.8	115.6	5.2
14	A23-9–**B31-18**[26]	B	B31					C-2'	123.5	127.2	-3.7
32	**B31-19**–D32-5[28]	B	B31	C-1	161.1	157.9	3.2	C-1'	121.0	115.8	5.2
32	**B31-19**–D32-5[28]	B	B31	C-8	132.7	127.9	4.8	C-2'	123.5	127.0	-3.5
16	A23-12–**B31-21**[27]	B	B31					C-1'	120.9	115.7	5.2
16	A23-12–**B31-21**[27]	B	B31					C-2'	123.6	127.1	-3.5
16	A23-12–**B31-21**[28]	B	B31					C-1'	120.9	115.6	5.3
16	A23-12–**B31-21**[28]	B	B31					C-2'	123.6	127.0	-3.4
16	A23-12–**B31-21**[29]	B	B31					C-1'	120.9	115.7	5.3
16	A23-12–**B31-21**[29]	B	B31					C-2'	123.6	127.0	-3.4
33	B34-1[12]	B	B34	C-1	117.7	114.4	3.3	C-1'	123.5	121.5	2.0
33	B34-1[12]	B	B34	C-8	129.5	126.7	2.8	C-2'	126.5	131.0	-4.5
34	B34-2[43]	B	B34	C-1	165.7	162.8	2.9	C-1'	123.3	121.1	2.2
34	B34-2[43]	B	B34	C-8	116.8	112.0	4.8	C-2'	126.8	131.6	-4.8
35	B34-3[44]	B	B34	C-1	155.3	154.6	0.7	C-1'	123.2	121.9	1.3
35	B34-3[44]	B	B34	C-8	117.5	113.4	4.1	C-2'	127.0	132.5	-5.5
36	B34-4[45]	B	B34	C-1	147.0	147.0	0.0	C-1'	124.9	122.1	2.8
36	B34-4[45]	B	B34	C-8	116.3	113.2	3.1	C-2'	130.0	132.7	-2.7
37	B34-5[46]	B	B34	C-1	164.4	163.3	1.1	C-1'	125.0	122.1	2.9
37	B34-5[46]	B	B34	C-8	115.9	113.0	2.9	C-2'	130.0	132.5	-2.5

SI number	Code	Category	Subcategory	C-1 and C8	δ_{calc}	δ_{exp}	δ_{Calc}-δ_{exp}	C pyran	δ_{calc}	δ_{exp}	δ_{Calc}-δ_{exp}

30	B31-15–**B34-6**[26]	B	B34					C-1''	125.2	121.5	3.7
30	B31-15–**B34-6**[26]	B	B34					C-2''	129.7	131.1	-1.4
38	**B34-8**–D31-9[40]	B	B34	C-1	166.4	162.1	4.3	C-1''	123.3	120.9	2.4
38	**B34-8**–D31-9[40]	B	B34	C-8	116.9	114.8	2.1	C-2''	126.6	131.0	-4.4
38	**B34-8**–D31-9[47]	B	B34	C-1	165.4	164.5	0.9	C-1''	123.3	122.4	0.9
38	**B34-8**–D31-9[47]	B	B34	C-8	116.9	116.0	0.9	C-2''	126.6	133.0	-6.4
39	**B34-10**–D31-10[48]	B	B34	C-1	165.3	163.3	2.0	C-1''	125.6	122.2	3.4
39	**B34-10**–D31-10[48]	B	B34	C-8	117.1	113.7	3.4	C-2''	126.2	130.0	-3.8
40	C21-1[12]	C	C21	C-1	152.0	148.7	3.3	C-1'	122.5	120.1	2.4
40	C21-1[12]	C	C21	C-8	129.2	125.8	3.4	C-2'	133.0	135.2	-2.2
41	C21-2[12]	C	C21	C-1	151.2	147.1	4.1	C-1'	123.9	121.5	2.4
41	C21-2[12]	C	C21	C-8	129.8	126.7	3.1	C-2'	135.8	136.5	-0.7
42	C24-1[12]	C	C24	C-1	106.4	100.7	5.7	C-1'	119.8	116.3	3.5
42	C24-1[12]	C	C24	C-8	130.4	125.8	4.6	C-2'	131.2	132.9	-1.7
43	C24-2[12]	C	C24	C-1	111.9	106.7	5.2	C-1'	120.3	116.4	3.9
43	C24-2[12]	C	C24	C-8	129.8	126.7	3.1	C-2'	133.7	135.6	-1.9
44	C24-3[49]	C	C24	C-1	112.0	106.9	5.1	C-1'	120.4	116.4	4.0
44	C24-3[49]	C	C24	C-8	132.5	128.7	3.8	C-2'	133.5	135.5	-2.0
45	C24-4[49]	C	C24	C-1	111.5	106.5	5.0	C-1'	120.4	116.4	4.0
45	C24-4[49]	C	C24	C-8	108.6	105.6	3.0	C-2'	133.4	135.6	-2.2
46	C24-5[49]	C	C24	C-1	111.8	106.7	5.1	C-1'	120.4	116.4	4.0
46	C24-5[49]	C	C24	C-8	129.7	125.9	3.8	C-2'	133.6	135.5	-1.9

SI number	Code	Category	Subcategory	C-1 and C8	δ_{calc}	δ_{exp}	δ_{Calc}-δ_{exp}	C pyran	δ_{calc}	δ_{exp}	δ_{Calc}-δ_{exp}

47	C24-6[49]	C	C24	C-1	111.9	106.7	5.2	C-1'	120.0	116.3	3.7
47	C24-6[49]	C	C24	C-8	129.7	129.6	0.1	C-2'	134.3	135.8	-1.5
48	C24-7[49]	C	C24	C-1	112.0	109.5	2.5	C-1'	120.5	116.6	3.9
48	C24-7[49]	C	C24	C-8	132.1	128.1	4.0	C-2'	132.8	134.7	-1.9
49	C40-1[50]	C	C40	C-1	119.2	117.6	1.7	C-1'	123.3	121.3	1.3
49	C40-1[50]	C	C40	C-8	129.7	126.6	3.1	C-2'	129.4	133.5	-4.1
49	C40-1[12]	C	C40	C-1	119.2	117.6	1.6	C-1'	123.3	121.3	1.3
49	C40-1[12]	C	C40	C-8	129.7	126.0	3.7	C-2'	129.4	133.5	-4.1
50	C40-2[51]	C	C40	C-1	120.7	118.4	2.3	C-1'	125.3	123.9	1.4
59	C40-2[51]	C	C40	C-8	129.7	126.6	3.1	C-2'	132.5	134.6	-2.1
51	C40-3[50]	C	C40	C-1	164.0	161.8	2.2	C-1'	123.0	121.9	1.1
51	C40-3[50]	C	C40	C-8	118.5	116.8	1.7	C-2'	130.0	134.0	-4.0
52	C40-4[52]	C	C40	C-1	155.4	154.2	1.2	C-1'	122.9	122.1	0.8
52	C40-4[52]	C	C40	C-8	119.1	117.3	1.8	C-2'	130.1	134.9	-4.8
53	C40-5[53]	C	C40	C-1	153.9	153.0	0.9	C-1'	124.6	121.8	2.8
53	C40-5[53]	C	C40	C-8	120.5	117.6	2.9	C-2'	132.8	133.8	-1.0
53	C40-5[54]	C	C40	C-1	153.9	153.0	0.9	C-1'	124.6	121.8	2.8
53	C40-5[54]	C	C40	C-8	120.5	117.6	2.9	C-2'	132.8	133.8	-1.0
54	C41-1[12]	C	C41	C-1	155.9	154.7	1.2	C-1'	123.2	122.2	1.0
54	C41-1[12]	C	C41	C-8	129.1	125.9	3.2	C-2'	132.3	136.2	-3.9
55	C41-2[12]	C	C41	C-1	152.9	153.7	-0.8	C-1'	122.9	121.9	1.0
55	C41-2[12]	C	C41	C-8	129.7	126.7	3.0	C-2'	131.0	133.9	-2.9

SI number	Code	Category	Subcategory	C-1 and C8	δ_{calc}	δ_{exp}	δ_{Calc}-δ_{exp}	C pyran	δ_{calc}	δ_{exp}	δ_{Calc}-δ_{exp}

56	C41-3[55]	C	C41	C-1	157.6	155.2	2.4	C-1'	125.0	122.2	2.8
56	C41-3[55]	C	C41	C-8	118.3	115.8	2.5	C-2'	135.3	135.9	-0.6
57	D30-1[56]	D	D30	C-1	130.9	127.3	3.6	C-1'	120.2	115.1	5.1
57	D30-1[56]	D	D30	C-8	128.7	126.1	2.6	C-2'	128.3	130.1	-1.8
58	D30-2[56]	D	D30	C-1	130.9	130.7	4.3	C-1'	120.1	114.8	5.3
58	D30-2[56]	D	D30	C-8	129.1	125.0	4.1	C-2'	127.9	130.7	-2.8
59	D30-3[32]	D	D30	C-1	131.3	129.7	1.6	C-1'	118.1	115.4	2.7
59	D30-3[32]	D	D30	C-8	120.6	117.4	3.2	C-2'	125.1	127.1	-2.0
60	D30-4[57]	D	D30	C-1	155.7	152.6	3.1	C-1'	116.5	114.1	2.4
60	D30-4[57]	D	D30	C-8	131.3	126.1	5.2	C-2'	126.6	130.6	-4.0
61	D30-5[57]	D	D30	C-1	155.5	152.4	3.1	C-1'	116.6	114.0	2.6
61	D30-5[57]	D	D30	C-8	131.3	126.1	5.2	C-2'	126.3	130.4	-4.1
62	D30-6[57]	D	D30	C-1	153.8	151.7	2.1	C-1'	116.6	113.9	2.7
62	D30-6[57]	D	D30	C-8	131.3	126.2	5.1	C-2'	126.3	130.5	-4.2
63	D31-1[58]	D	D31	C-1	165.6	163.2	2.4	C-1'	117.4	115.0	2.4
63	D31-1[58]	D	D31	C-8	129.0	125.9	3.1	C-2'	122.8	127.2	-4.4
64	D31-2[32]	D	D31	C-1	162.7	161.7	1.0	C-1'	118.3	115.4	2.9
64	D31-2[32]	D	D31	C-8	129.8	127.0	2.8	C-2'	122.6	126.7	-4.1
65	D31-3[34]	D	D31	C-1	165.5	162.4	3.1	C-1'	117.4	114.5	2.9
65	D31-3[34]	D	D31	C-8	110.6	108.1	2.5	C-2'	122.5	127.8	-5.3
65	D31-3[42]	D	D31	C-1	165.5	163.0	2.5	C-1'	117.4	114.5	2.9
65	D31-3[42]	D	D31	C-8	110.6	109.3	1.3	C-2'	122.5	127.0	-4.5

SI number	Code	Category	Subcategory	C-1 and C8	δ_{calc}	δ_{exp}	δ_{Calc}-δ_{exp}	C pyran	δ_{calc}	δ_{exp}	δ_{Calc}-δ_{exp}

66	D31-4[32]	D	D31	C-1	165.4	162.9	2.5	C-1'	117.8	115.6	2.2
66	D31-4[32]	D	D31	C-8	119.8	116.5	3.3	C-2'	122.3	127.0	-4.7
67	D31-5[59]	D	D31	C-1	165.9	164.2	1.7	C-1'	118.1	115.9	2.2
67	D31-5[59]	D	D31	C-8	121.6	117.5	4.1	C-2'	125.2	128.0	-2.8
68	D31-6[32]	D	D31	C-1	163.0	161.5	1.5	C-1'	119.5	115.8	3.7
68	D31-6[32]	D	D31	C-8	111.2	105.7	5.5	C-2'	123.7	126.9	-3.2
5	A20-5–**D31-7**[16]	D	D31					C-1'	119.4	114.9	4.5
5	A20-5–**D31-7**[16]	D	D31					C-2'	123.9	126.8	-2.9
5	A20-5–**D31-7**[17]	D	D31					C-1'	119.4	115.1	4.3
5	A20-5–**D31-7**[17]	D	D31					C-2'	123.9	126.8	-2.9
15	A23-11–**D31-8**[25]	D	D31					C-1'	119.5	115.3	4.2
15	A23-11–**D31-8**[25]	D	D31					C-2'	123.9	127.0	-3.1
38	B34-8–**D31-9**[40]	D	D31					C-1'	117.8	111.8	6.0
38	B34-8–**D31-9**[40]	D	D31					C-2'	122.2	126.5	-4.3
38	B34-8–**D31-9**[47]	D	D31					C-1'	117.8	113.7	4.1
38	B34-8–**D31-9**[47]	D	D31					C-2'	122.2	128.6	-6.4
39	B34-10–**D31-10**[48]	D	D31					C-1'	120.1	115.4	4.7
39	B34-10–**D31-10**[48]	D	D31					C-2'	124.1	127.3	-3.2
69	D31-11[52]	D	D31	C-1	166.2	163.8	2.4	C-1'	118.4	115.9	2.5
69	D31-11[52]	D	D31	C-8	143.0	136.7	6.3	C-2'	125.2	127.2	-2.0

SI number	Code	Category	Subcategory	C-1 and C8	δ_{calc}	δ_{exp}	δ_{Calc}-δ_{exp}	C pyran	δ_{calc}	δ_{exp}	δ_{Calc}-δ_{exp}

70	D31-12[60]	D	D31	C-1	165.3	163.8	1.5	C-1'	118.3	116.8	1.5
70	D31-12[60]	D	D31	C-8	121.7	118.2	3.5	C-2'	125.6	127.8	-2.2
19	B13-1–**D31-18**[32]	D	D31					C-1''	120.9	115.8	5.1
19	B13-1–**D31-18**[32]	D	D31					C-2''	123.7	126.9	-3.2
71	D32-1[42]	D	D32	C-1	113.4	108.7	4.7	C-1'	117.9	115.4	2.5
71	D32-1[42]	D	D32	C-8	164.1	161.8	2.3	C-2'	125.5	129.9	-4.4
72	D32-2[61]	D	D32	C-1	165.7	162.5	3.2	C-1'	118.1	114.7	3.4
72	D32-2[61]	D	D32	C-8	113.4	108.7	4.7	C-2'	125.5	130.8	-5.3
73	D32-3[62]	D	D32	C-1	164.7	162.8	1.9	C-1'	118.0	115.0	3.0
73	D32-3[62]	D	D32	C-8	113.4	108.7	4.7	C-2'	125.3	130.6	-5.3
31	B31-16–**D32-4**[42]	D	D32					C-1''	118.2	115.4	2.8
31	B31-16–**D32-4**[42]	D	D32					C-2''	125.9	129.9	-4.0
32	B31-19–**D32-5**[28]	D	D32					C-1''	120.6	115.5	5.1
32	B31-19–**D32-5**[28]	D	D32					C-2''	127.0	130.8	-3.8
74	D32-6[63]	D	D32	C-1	160.6	157.8	2.8	C-1'	118.1	114.7	3.4
74	D32-6[63]	D	D32	C-8	113.3	108.1	5.2	C-2'	125.3	130.3	-5.0
75	D32-7[64]	D	D32	C-1	160.7	158.7	2.0	C-1'	119.2	115.8	3.4
75	D32-7[64]	D	D32	C-8	113.1	109.3	3.8	C-2'	126.9	131.5	-4.6
76	D32-8[65]	D	D32	C-1	160.7	155.3	5.4	C-1'	118.3	114.7	3.6
76	D32-8[65]	D	D32	C-8	118.4	117.0	1.4	C-2'	126.1	127.8	-1.7

IV. Statistical analysis to obtain empirical correction factors for C-1, C-8, C-1′ and C-2′ carbons.

To address systematic discrepancies between calculated and experimental chemical shifts, empirical correction factors were determined from statistical analysis of a modeling set comprising seventy-six pyranoxanthones (SI: II.2. Figures SI-6.1 and 6.2). Consistent deviations were observed for C-1 and C-8 of the xanthone core, and for the C-1′ and C-2′ olefinic carbons of the pyran moiety. Specifically, calculated shifts for C-1, C-8, and C-1′ were consistently higher, showing positive differences versus the corresponding experimental values, whereas C-2′ shifts showed lower values with negative differences (SI: III, Table S-1). Eleven compounds in the modeling set contained two pyran moieties, assigned a dual subtype code separated by a long dash (SI: II.2 Figures SI-6.1 and 6.2). Both pyran moieties were included in the analysis for C-1′ and C-2′. When multiple ^{13}C NMR datasets were available for a compound—sometimes recorded in different deuterated solvents—all were incorporated to enhance the robustness of the analysis (SI: III, Table S-1). In total, 107 pairs of calculated and experimental values for C-1, C-8, C-1′, and C-2′ were analyzed. Given the symmetrical positions of C-1 and C-8 and their similar deviations, they were evaluated together (C1+C8) while C-1′ and C-2′ were analyzed separately. Statistical analyses were performed using the SIMFIT[66] program, first for each group separately (A, B, C, and D) and then for their combined dataset ("gathered"); see Figure SI-7, Tables SI-2.1–2.3).

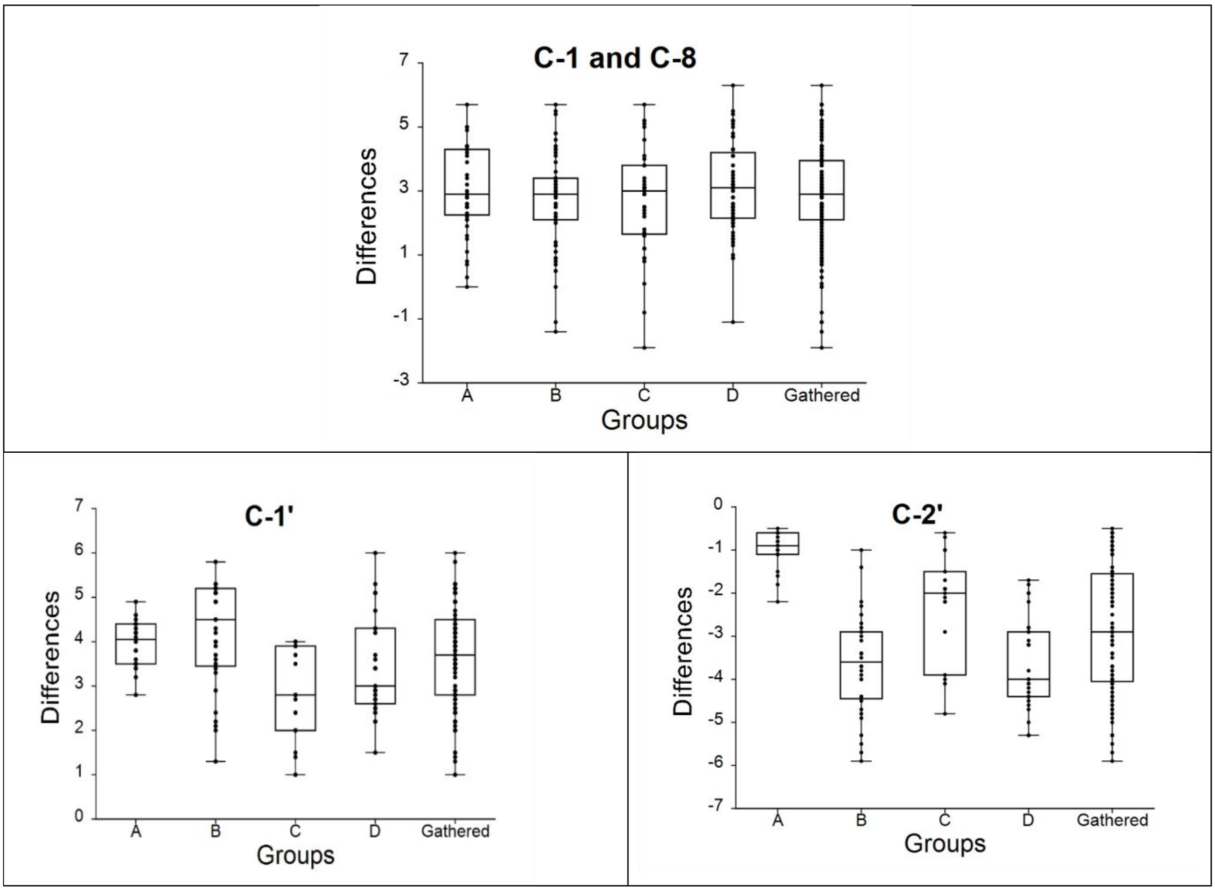

Figure SI-7. Box plots depicting the differences between calculated and experimental ^{13}C NMR chemical shifts for the C-1 and C-8 together, C-1' and C-2' in pyranoxanthones.

The Kolmogorov–Smirnov test confirmed normal distribution for all five sets (A, B, C, D, and gathered), with p-values for the gathered datasets of 0.16 (C-1+C-8), 0.65 (C-1′), and 0.25 (C-2′)**.** Accordingly, mean values of the gathered sets could be used to represent the data of individual groups and their mean values used as correction factors. Mean differences in the case of groups A, B, C and D for C-1+C-8 were similar (A: +3.0 ppm; B: +2.7 ppm; C: +2.7; D: +3.1 ppm), regarding C-1′ groups values were also similar (A: +3.9 ppm; B: +4.2 ppm; D: +3.4 ppm), with slightly lower shifts for type C (+2.7 ppm), for C-2′ carbon, mean differences for groups B and D were identical (-3.6 ppm), somewhat lower for type C (−2.3 ppm), and much lower for type A (−1.0 ppm). The gathered dataset yielded mean differences values of +2.9 ppm (C-1+C-8), +3.7 ppm (C-1′), and −2.8 ppm (C-2′), closely matching those of their individual groups. To check whether the mean differences are equal for all sets studied (A, B, C, D and gathered) the ANOVA procedure was used. In the case of C1+C8 carbons the p-value was 0.53 meaning equality of all means and suggesting that the mean of the gathered set can be taken as average for a correction factor between calculated and experimental values. For C1' the ANOVA showed $p<0.05$ but the Tukey analysis comparing all means

in pairs showed that the gathered mean was significantly equal to means of groups A, B, C and D and therefore may be also appropriate for a correctio factor. For C-2' the ANOVA had $p<0.05$ but the Tuckey tests showed equality of means of the gathered set with group C ($p=0.5$) and roughly with D ($p=0.03$) but not with groups A and B; in contrast with the better results found with C1+C8 and C-1′ probably due to a smaller sample size in C-2´. In summary, the gathered sets may be considered a good representation for the isolated groups A, B, C and D. Thus, the following correction factors were applied to calculated ^{13}C NMR shifts for pyranoxanthones: subtract 2.9 ppm for both C-1 and C-8 carbons, subtract 3.7 ppm for C-1′, and add 2.8 ppm for C-2′. Applying these corrections markedly improved agreement between calculated and experimental shifts (SI: V and VIII).

Table SI-2.1. Differences between calculated and experimental ^{13}C NMR chemical shifts for the C-1 & C-8.

Set	N	Mean	95% CL
Group A	44	3.0	2.6-3.4
Group B	72	2.7	2.4-3.0
Group C	38	2.7	2.2-3.3
Group D	60	3.1	2.7-3.5
**Gathered**	214	2.9	2.7-3.1

N: number of cases; 95% CL: confidence limits based on the Student's t-test.

Table SI-2.2. Differences between calculated and experimental ^{13}C NMR chemical shifts for the C-1'.

Set	N	Mean	95% CL
Group A	22	3.9	3.7 to 4.2
Group B	34	4.2	3.8 to 4.6
Group C	19	2.7	2.2 to 2.4
Group D	29	3.4	3.0 to 3.9
Gathered	104	3.7	3.4 to 3.9

N: number of cases; 95% CL: confidence limits based on the Student's t-test.

Table SI-2.3. Differences between calculated and experimental ^{13}C NMR chemical shifts for the C-2'.

Set	N	Mean	95% CL
Group A	22	-1.0	-1.2 to -0.8
Group B	34	-3.6	-4.0 to -3.3
Group C	19	-2.3	-2.9 to -1.7
Group D	29	-3.6	-4.1 to -3.2
Gathered	104	-2.8	-3.1 to -2.6

N: number of cases; 95% CL: confidence limits based on the Student's t-test.

V. Experimental and Calculated ¹³C NMR Data and Cartesian Coordinates of the Global Minimum Conformers of the Compounds Shown in Figures SI-4.1 and SI-4.2 Used as the Modeling Set

The calculated and experimental ^{13}C NMR chemical shifts (δ, ppm) are reported for each carbon atom of the pyranoxanthones selected as the modeling set. Carbon numbering follows the conventional system used for xanthones, and the corresponding carbon types are also indicated. The difference between calculated and experimental values ($\Delta\delta = \delta_{cal} - \delta_{exp}$) is also provided. For each compound, the root-mean-square deviation (RMSD) and the maximum absolute deviation (Max Abs) are included without applying empirical correction factors. These same parameters are recalculated after applying the correction factors (−2.9 ppm for C1 and C8; −3.7 ppm for C1′; and +2.8 ppm for C2′), which were derived from a previous statistical analysis. The corrected values are reported as RMSD+CFx and Max Abs+CFx. In the table, the correction factors applied to each carbon appear in parentheses after the calculated chemical shift value. These values were not considered in the difference column but were included in the calculation of the RMSD+CFx and Max Abs+CFx parameters. An asterisk next to an experimental chemical shift indicates that the value has been reassigned based on the calculated data.
Additionally, the cartesian coordinates of the lowest-energy conformers are provided in MOL2 format. The names listed in the headings correspond to those used in the original publication or the names of the revised structures, along with the reference codes used throughout this work. *The number of conformers considered for the calculation of the ^{13}C NMR data is reported. The average energy is expressed in Hartrees at the ωB97X-V/6-311+G(2df,2p)[6-311G*] level

V.1. 3,3-Dimethylpyrano[3,2-a]xanthen-12(3H)-one (A20-1).[12]

No	type	δ_{cal}	δ_{exp}	diff
C1	C	124.1 (-2.9)	124.1	0.0 (-2.9)
C2	C	149.1	149.1	0.0
C3	CH	124.5	123.4	1.1
C4	CH	118.2	118	0.2
C4a	C	151.4	151.9	-0.5
C5	CH	116.7	116.3	0.4
C6	CH	133.5	134.3	-0.8
C7	CH	121.9	120	1.9
C8	CH	130 (-2.9)	126.6	3.4 (-2.9)
C8a	C	123.7	122.2	1.5
C9	C	181	179.1	1.9
C9a	C	118.7	117.4	1.3
C10a	C	155.1	155.3	-0.2
C1′	CH	124.5 (-3.7)	120.9	3.6 (-3.7)
C2′	CH	131.9 (+2.8)	132.5	-0.6 (+2.8)
C3′	C	75.6	75.4	0.2
C4′[2]	CH3	27.1	27.3	-0.2
RMSD		1.45		
Max Abs		3.57		
RMSD+CFx		1.22		
Max abs+CFx		2.95		

mol2 coordinates for lowest energy conformer

1	C1	-0.890385643	-4.097496445	-2.721756813
2	C2	1.301798923	-2.859885539	-2.537048001
3	O3	-0.754110700	2.215364347	-1.048368902
4	C4	-0.909418979	-0.536134743	-1.761884428
5	C5	-0.857540136	-1.594987964	-2.573214044
6	C6	0.370028120	-2.154449826	1.355198628
7	C7	0.563061060	-1.093105340	2.213140112
8	C8	0.558635093	3.414708743	3.388856491
9	C9	-0.345204042	4.230585998	0.875163670
10	O10	-0.286152895	-3.025846944	-0.718595829
11	O11	0.546510289	1.178058686	2.692591768
12	C12	-0.113810658	5.169678003	1.860809454
13	C13	-0.077118498	-1.928669753	0.050764105
14	C14	0.339957247	4.754033264	3.120697540
15	C15	-0.355001860	-0.637766732	-0.408502310
16	C16	-0.381624891	1.867713702	0.064396652
17	C17	0.312530892	0.204359310	1.765791254

18	C18	0.318188535	2.475885728	2.382796799
19	C19	-0.133314281	2.866520894	1.123484769
20	C20	-0.143042583	0.461501177	0.464969205
21	C21	-0.181219417	-2.875258422	-2.149543528
22	H22	-0.827891007	-4.093846767	-3.814789244
23	H23	-1.944139014	-4.097131633	-2.428816773
24	H24	-0.422388980	-5.013107736	-2.348385651
25	H25	1.810612929	-2.013279624	-2.065069829
26	H26	1.787106033	-3.788296160	-2.218458763
27	H27	1.409018746	-2.759392973	-3.621944532
28	H28	-1.354044977	0.398904281	-2.070175930
29	H29	-1.271956701	-1.552693620	-3.576858353
30	H30	0.559492685	-3.174825690	1.670172972
31	H31	0.914539417	-1.238334318	3.228322522
32	H32	0.909758760	3.072631293	4.356234387
33	H33	-0.694485105	4.512306368	-0.112745936
34	H34	-0.281242855	6.223346621	1.663849207
35	H35	0.522854493	5.488911814	3.898919329

2 lowest-energy conformers used for Boltzmann-averaged ^{13}C NMR data.
Avg. Energy: -920.052279 au.

V.2. Toxyloxanthone E (A20-2).[13]

No	type	δcal	δexp	diff
C1	C	124.1 (-2.9)	120.2	3.9 (-2.9)
C2	C	149.4	149.6	-0.2
C3	CH	124.9	124.6	0.3
C4	CH	117.8	117.9	-0.1
C4a	C	151.2	151.9	-0.7
C5	CH	91.6	93.7	-2.1
C6	C	162.4	163	-0.6
C7	CH	96.3	98.4	-2.1
C8	C	166.0 (-2.9)	164.1	1.9 (-2.9)
C8a	C	105.4	104.7	0.7
C9	C	185.4	183.6	1.8
C9a	C	117.3	115.3	2.0
C10a	C	157.9	157.6	0.3
C1'	CH	124.3 (-3.7)	120.9	3.4 (-3.7)
C2'	CH	132.3 (+2.8)	133	-0.7 (+2.8)
C3'	C	75.6	75.8	-0.2
C4'[2]	CH3	26.8	27.5	-0.7
RMSD		1.66		
Max Abs		3.93		
RMSD+CFx		1.18		
Max abs+CFx		2.14		

mol2 coordinates for lowest energy conformer

1	C1	0.855390809	4.330491192	-2.910356861
2	C2	-1.316918631	3.078173967	-2.612435125
3	O3	0.734506750	-1.938339984	-1.105170204
4	C4	0.919112022	0.787784294	-1.867707000
5	C5	0.852270498	1.830698505	-2.698623158
6	C6	-0.295973364	2.475496657	1.239108034
7	C7	-0.494426412	1.432677026	2.118955483
8	C8	-0.586034744	-3.036066171	3.368941074
9	C9	0.277854861	-3.940562281	0.845189148
10	O10	0.343071795	3.297921716	-0.861065720
11	O11	-0.665886507	-5.244537871	4.126848824
12	O12	-0.512833237	-0.831908413	2.629311251
13	C13	0.022593721	-4.852170335	1.862796800
14	C14	0.128666916	2.219544379	-0.066958845

15	C15	-0.405528103	-4.394072443	3.110500681
16	C16	0.383388151	0.916921965	-0.508630761
17	C17	0.366497896	-1.576654365	0.025937159
18	C18	-0.272597218	0.124731868	1.689052225
19	C19	-0.325866975	-2.140345612	2.346048230
20	C20	0.107749978	-2.545551617	1.072502173
21	C21	0.162885438	-0.161570782	0.388589646
22	C22	0.180119101	3.116012391	-2.282450019
23	O23	0.683014323	-4.401940695	-0.336058876
24	H24	1.920705448	4.343637747	-2.661974363
25	H25	0.746780414	4.303593827	-3.999709613
26	H26	0.396127685	5.250286237	-2.536729938
27	H27	-1.798319801	4.010506111	-2.299525500
28	H28	-1.464207420	2.948741107	-3.689584851
29	H29	-1.799532852	2.239344838	-2.099868703
30	H30	1.363577070	-0.150578873	-2.164685281
31	H31	1.248802220	1.770020048	-3.708544418
32	H32	-0.464911066	3.504203011	1.537771670
33	H33	-0.829787656	1.600290670	3.135947245
34	H34	-0.919380090	-2.692603362	4.339522547
35	H35	-0.506564223	-6.150625137	3.834799543
36	H36	0.162197288	-5.909760731	1.661469140
37	H37	0.803455916	-3.613788884	-0.923211637

2 lowest-energy conformers used for Boltzmann-averaged ^{13}C NMR data.
Avg. Energy: -1070.523469 au.

V.3. Thwaitesixanthone (A20-3–B31-17).[14]

No	type	δcal	δexp	diff
C1	C	160.9 (-2.9)	158	2.9 (-2.9)
C2	C	104.9	104.3	0.6
C3	C	161.5	160.5	1.0
C4	CH	94.2	94.2	0.0
C4a	C	157.2	157	0.2
C5	CH	117.9	117.7	0.2
C6	CH	124.8	124.2	0.6
C7	C	149.3	149.5	-0.2
C8	C	123.8 (-2.9)	118.1	5.7 (-2.9)
C8a	C	117.2	114.5	2.7
C9	C	185.7	183.9	1.8
C9a	C	105.5	104	1.5
C10a	C	151.1	152	-0.9
C1'	CH	120.6 (-3.7)	115.5	5.1 (-3.7)
C2'	CH	123.5 (+2.8)	127.2	-3.7 (+2.8)
C3'	C	78.2	78.2	0.0
C4'^{2}	CH3	28.1	28.3	-0.2
C1''	CH	124.3 (-3.7)	120.8	3.5 (-3.7)
C2''	CH	132.0 (+2.8)	132.6	-0.6 (+2.8)
C3''	C	75.5	75.4	0.1
C4''^{2}	CH3	26.9	27.3	-0.4
RMSD		2.17		
Max Abs		5.65		
RMSD+CFx		1.16		
Max abs+CFx		2.75		

mol2 coordinates for lowest energy conformer

1	C1	0.873005141	6.215317119	-3.452861820
2	C2	-1.222284569	4.810255386	-3.518005248
3	C3	-1.200651499	-6.029666245	3.553022312
4	C4	1.083108730	-4.959351425	3.676332650
5	O5	0.986812208	-0.130136917	-1.959544633

6	C6	0.287438054	-4.677263635	0.477262733
7	C7	1.061767977	2.636914702	-2.569917909
8	C8	0.096435617	-5.559169557	1.459042407
9	C9	1.022039736	3.714191508	-3.357438202
10	C10	-0.648095750	4.070679100	0.428421741
11	C11	-0.868091625	2.973161788	1.232779023
12	C12	-0.801297955	-1.554537180	2.251957818
13	C13	0.428786423	-2.262251890	-0.181396059
14	O14	0.158664020	5.043607186	-1.544106291
15	O15	-0.866458293	-3.805793248	2.858186906
16	O16	-0.781921240	0.689276751	1.635841142
17	C17	0.109718518	-3.256093980	0.743977162
18	C18	-0.055195229	3.912831256	-0.827070702
19	C19	-0.499527998	-2.880291204	1.955052473
20	C20	0.353425282	2.657123325	-1.286597401
21	C21	0.458857390	0.143556289	-0.866895675
22	C22	-0.499410586	1.707991912	0.776990620
23	C23	-0.476929735	-0.587806074	1.315211979
24	C24	0.145100095	-0.895157527	0.094008749
25	C25	0.109671919	1.520645110	-0.470391674
26	C26	0.208228279	4.927698850	-2.980926173
27	C27	-0.208393487	-5.099444628	2.864963477
28	O28	0.996609152	-2.626427496	-1.330808060
29	H29	1.885919217	6.290933439	-3.048098991
30	H30	0.927676146	6.233404323	-4.545800101
31	H31	0.295752190	7.082082210	-3.118194180
32	H32	-1.708402606	3.914683501	-3.119157032
33	H33	-1.211926822	4.733535952	-4.609934464
34	H34	-1.808411543	5.689616007	-3.231523369
35	H35	-0.770497399	-7.031153895	3.652452748
36	H36	-2.122951932	-6.099054895	2.969835781
37	H37	-1.443070760	-5.653637965	4.551171546
38	H38	0.859010548	-4.614391902	4.690940790
39	H39	1.599184825	-5.922950863	3.736604572
40	H40	1.755912806	-4.241411224	3.197274962
41	H41	0.544778368	-4.984023527	-0.530575232
42	H42	1.612156615	1.749503562	-2.845742806
43	H43	0.191506802	-6.628521417	1.294675017
44	H44	1.549335391	3.733846490	-4.307309427
45	H45	-0.930614486	5.068082565	0.747133989
46	H46	-1.333104309	3.064286274	2.207587215
47	H47	-1.275015222	-1.287978328	3.187718658
48	H48	1.131351598	-1.796709584	-1.856151020

1 lowest-energy conformers used for Boltzmann-averaged ^{13}C NMR data.
Avg. Energy: -1264.676515 au.

V.4. Caledonixanthone M (A20-4).[15]

No	type	δcal	δexp	diff
C1	C	160.4 (-2.9)	157.9	2.5 (-2.9)
C2	C	107.2	107.6	-0.4
C3	C	166.8	166.9	-0.1
C4	CH	88.2	88.4	-0.2
C4a	C	158.0	157.7	0.3
C5	CH	117.9	117.6	0.3
C6	CH	124.8	124.2	0.6
C7	C	149.4	149.3	0.1
C8	C	123.7 (-2.9)	120.8	2.9 (-2.9)
C8a	C	117.3	115	2.3
C9	C	185.7	183.4	2.3
C9a	C	106.3	104.7	1.6

C10a	C	151.1	157.6	-6.5
C1'	CH2	28.3	26.8	1.5
C2'	CH	94.1	91.9	2.2
C3'	C	71.5	71.9	-0.4
C4'	CH3	23.0	25.9	-2.9
C5'	CH3	25.3	29.3	-4.0
C1''	CH	124.3 (-3.7)	119.8	4.5 (-3.7)
C2''	CH	131.8 (+2.8)	132.6	-0.8 (+2.8)
C3''	C	75.7	75.5	0.2
C4''	CH3	27.0	27.3	-0.3
C5''	CH3	27.0	27.9	-0.9
RMSD		2.33		
Max Abs		6.52		
RMSD+CFx		2.02		
Max abs+CFx		6.52		

mol2 coordinates for lowest energy conformer

1	O1	0.928532002	-1.012727955	-1.870841996
2	C2	-2.668564081	-0.042917676	1.430693029
3	C3	4.368344114	0.832614617	1.408495461
4	C4	-2.727223109	-0.616211829	0.163773412
5	C5	4.533474900	0.274248078	0.137647589
6	C6	-1.497840425	0.389966823	2.032858632
7	C7	3.110629610	0.931502179	1.963823091
8	C8	-1.568623103	-0.774679147	-0.570787065
9	C9	3.443514450	-0.214474381	-0.589305840
10	O10	0.810041317	0.631018178	1.869290449
11	C11	0.917049857	-0.529545825	-0.725058132
12	C12	-0.337072265	0.213730780	1.287351851
13	C13	2.008058541	0.476512622	1.240782596
14	C14	-0.325362487	-0.362873729	0.004370550
15	C15	2.143041744	-0.093039866	-0.031257148
16	O16	-3.882659051	0.052902050	2.012385539
17	C17	-4.851175675	-0.653046429	1.182786969
18	C18	-4.162662938	-0.884779470	-0.178802084
19	C19	-6.127946387	0.200888876	1.143793270
20	C20	-7.172514402	-0.458214904	0.249376337
21	C21	-6.678134265	0.398928687	2.559829181
22	O22	-5.828472137	1.455460450	0.553322485
23	C23	3.758276774	-0.882738898	-1.855518333
24	C24	4.974161970	-0.740012023	-2.387965853
25	C25	6.008137298	0.153327002	-1.748691584
26	O26	5.805427624	0.174085014	-0.319859075
27	C27	5.896940689	1.584458949	-2.283660174
28	C28	7.415582066	-0.401449753	-1.932456831
29	O29	-1.635037476	-1.305433953	-1.791200766
30	H30	5.247262177	1.180997864	1.939964350
31	H31	-1.470542369	0.829586877	3.021036562
32	H32	2.952664051	1.363676245	2.945321942
33	H33	-5.055603349	-1.604969718	1.688049004
34	H34	-4.336324856	-1.895786275	-0.555906984
35	H35	-4.527346659	-0.170591896	-0.925832915
36	H36	-8.072582590	0.162900222	0.224624870
37	H37	-6.805646592	-0.562282374	-0.775582268
38	H38	-7.443369461	-1.448314760	0.630458621
39	H39	-7.566255395	1.035627654	2.518936270
40	H40	-5.934019711	0.876931278	3.203611775
41	H41	-6.955067087	-0.558077823	3.015678404
42	H42	-5.124453599	1.849852546	1.086525256
43	H43	2.998602633	-1.489665168	-2.325940500

44	H44	5.242248540	-1.240260835	-3.314680987
45	H45	6.643017848	2.226813780	-1.804487755
46	H46	4.899833032	1.989445374	-2.086176392
47	H47	6.061526980	1.598539576	-3.365966496
48	H48	7.479258018	-1.417631183	-1.533757209
49	H49	7.676995456	-0.422223215	-2.995031447
50	H50	8.140010884	0.227626940	-1.407214766
51	H51	-0.708133105	-1.329693577	-2.138804892

9 lowest-energy conformers used for Boltzmann-averaged ^{13}C NMR data.
Avg. Energy: -1341.127644 au.

V.5. Cudraxanthone A (A20-5–D31-7).[16]

No	type	δcal	δexp	diff
C1	C	165.7(-2.9)	163.2*	2.5 (-2.9)
C2	CH	99.3	99	0.3
C3	C	161.5	160.5*	1
C4	C	100.5	100.3	0.2
C4a	C	152.8	151.5	1.3
C5	CH	117.6	117.6*	0
C6	CH	124.7	124.2	0.5
C7	C	149.5	149.4*	0.1
C8	C	124 (-2.9)	119.9*	4.1 (-2.9)
C8a	C	117.3	115.0*	2.3
C9	C	185.5	183.3	2.2
C9a	C	105.6	104.2	1.4
C10a	C	150.8	151.3*	-0.5
C1'	CH	119.4 (-3.7)	114.9	4.5 (-3.7)
C2'	CH	123.9 (+2.8)	126.8	-2.9 (+2.8)
C3'	C	78.1	75.6	2.5
C4'[2]	CH3	27.9	28.3	-0.4
C1''	CH	124.2 (-3.7)	120.7*	3.5 (-3.7)
C2''	CH	132.1 (+2.8)	132.7	-0.6 (+2.8)
C3''	C	75.7	78.1	-2.4
C4''[2]	CH3	26.9	27.3	-0.4
RMSD		2.00		
Max Abs		4.48		
RMSD+CFx		1.23		
Max abs+CFx		2.47		

mol2 coordinates for lowest energy conformer

1	O1	-0.134478268	-0.305268292	-2.776961925
2	C2	-3.620851233	0.389950411	0.748199365
3	C3	3.469021680	-0.330771639	0.833990022
4	C4	-3.723882180	0.292908372	-0.635850726
5	C5	3.574499105	-0.445552225	-0.554460915
6	C6	-2.387933714	0.298455917	1.418336104
7	C7	2.231427647	-0.183139268	1.423435544
8	C8	-2.578641613	0.091789477	-1.395527393
9	C9	2.440214067	-0.444437985	-1.373547038
10	O10	-0.077142859	0.028140062	1.295091576
11	C11	-0.094904721	-0.210754008	-1.538362660
12	C12	-1.254926218	0.098623094	0.634811519
13	C13	1.093142902	-0.143907963	0.619489184
14	C14	-1.304513474	-0.016498397	-0.762429728
15	C15	1.165318352	-0.273022911	-0.772783689
16	O16	-2.698942839	-0.007564130	-2.717460556
17	O17	4.821737601	-0.620274245	-1.056287199
18	C18	2.675310199	-0.678122867	-2.801761106
19	C19	3.915143521	-0.581431848	-3.286975934
20	C20	5.071636629	-0.176695869	-2.407220198

21	C21	5.246944162	1.345378269	-2.402729508
22	C22	6.358174651	-0.884571555	-2.814762177
23	C23	-2.367070739	0.512323534	2.859276405
24	C24	-3.517922595	0.546607495	3.532172345
25	C25	-4.830094125	0.277029168	2.834465738
26	O26	-4.753415949	0.634044060	1.430958994
27	C27	-5.947385621	1.152578493	3.389926370
28	C28	-5.187419394	-1.210188759	2.918205639
29	H29	4.376205991	-0.356541052	1.427766515
30	H30	-4.689709292	0.371371592	-1.118825078
31	H31	2.119575947	-0.083399225	2.497031951
32	H32	-1.787479826	-0.146679915	-3.078110220
33	H33	1.835570549	-0.933855247	-3.430882140
34	H34	4.120867776	-0.764943757	-4.338090779
35	H35	6.082877302	1.629326394	-1.755125663
36	H36	5.447264063	1.706556598	-3.416630147
37	H37	4.336040929	1.833367880	-2.041968931
38	H38	7.166153344	-0.623750607	-2.124978803
39	H39	6.651059559	-0.582252826	-3.825157936
40	H40	6.217824928	-1.968797609	-2.797931044
41	H41	-1.412765285	0.668483864	3.350426942
42	H42	-3.550474322	0.727276930	4.602638724
43	H43	-6.119290350	0.917263951	4.445009913
44	H44	-6.875155384	0.976929115	2.837653619
45	H45	-5.681030111	2.209622726	3.303171686
46	H46	-6.134288492	-1.402151906	2.403079293
47	H47	-4.401744016	-1.815353628	2.455265793
48	H48	-5.284548282	-1.518099670	3.964418251

1 lowest-energy conformers used for Boltzmann-averaged ^{13}C NMR data.
Avg. Energy: -1264.675434 au,

V.6. Batukinaxanthone (A20-6).[18]

C	Type	Theory	Expt.	diff.
C1	C	160.5 (-2.9)	158.4	2.1 (-2.9)
C2	C	106.6	108.5	-1.9
C3	C	162.1	160.5	1.6
C4	C	104.9	104.5	0.4
C4a	C	154.0	152.4	1.6
C5	CH	118.0	117.6	0.4
C6	CH	124.6	124.0	0.6
C7	C	149.1	149.0	0.1
C8	C	123.7 (-2.9)	120.9	2.8 (-2.9)
C8a	C	117.2	114.8	2.4
C9	C	185.7	183.0	2.7
C9a	C	105.2	104.0	1.2
C10a	C	151.3	151.8	-0.5
C1'	CH2	22.9	21.6	1.3
C2'	CH	125.4	121.8	3.6
C3'	C	136.6	135.3	1.3
C4'	CH3	25.6	25.9	-0.3
C5'	CH3	17.3	17.9	-0.6
C1''	CH2	23.2	21.7	1.5
C2''	CH	125.0	121.7	3.3
C3''	C	130.6	133.6	-3.0
C4''	CH3	18.2	17.9	0.3
C5''	CH3	25.5	25.8	-0.3
C1'''	CH	124.6 (-3.7)	121.8	2.8 (-3.7)
C2'''	CH	131.6 (+2.8)	132.4	-0.8 (+2.8)
C3'''	C	75.5	75.4	0.1
C4'''[2]	CH3	27.0	29.6	-2.6

RMSD	1.87
Max Abs	3.60
RMSD+CFx	1.72
Max abs+CFx	3.60

mol2 coordinates for lowest energy conformer

1	C1	2.164852754	-5.428041429	-5.317704760
2	C2	3.606871727	-3.659044387	-4.239833693
3	C3	-2.578067320	6.238471050	2.170338998
4	C4	-4.954348837	5.364689028	2.200171630
5	O5	-0.337027335	0.144604977	-3.043129065
6	C6	-1.960592928	3.996639483	0.055145276
7	C7	0.610050975	-2.325081291	-4.055692957
8	C8	-3.278110849	4.275356476	0.736110581
9	C9	1.255006231	-3.126396923	-4.906902725
10	C10	1.378012547	-4.140812879	-0.881300053
11	C11	0.939384511	-3.278985767	0.100326767
12	C12	-0.830409849	0.700274798	1.702440212
13	C13	-1.131740380	1.805308054	-0.887246221
14	O14	1.716419690	-4.688631955	-3.133269305
15	O15	-1.639584754	2.692359300	2.612625043
16	O16	0.039932965	-1.249995427	0.777649883
17	C17	-1.485973871	2.567475985	0.223862299
18	C18	1.297419036	-3.771475375	-2.226482886
19	C19	-1.327815045	1.993244168	1.498957837
20	C20	0.751064953	-2.543573369	-2.612625212
21	C21	-0.221889326	-0.299759097	-1.886476279
22	C22	0.424935009	-2.034213722	-0.263465713
23	C23	-0.465424343	-0.010768109	0.567987761
24	C24	-0.611916510	0.489832458	-0.735005223
25	C25	0.321018960	-1.641623399	-1.603096568
26	C26	2.185093677	-4.204996461	-4.409021984
27	C27	-3.562507193	5.227167927	1.638615610
28	O28	-1.274394311	2.341049389	-2.104130571
29	H29	1.146159835	-5.814899344	-5.411407718
30	H30	2.535393004	-5.165217246	-6.313780276
31	H31	2.802438775	-6.214712862	-4.904072053
32	H32	3.613487990	-2.820471973	-3.536700602
33	H33	3.991481636	-3.300136467	-5.200192024
34	H34	4.271981088	-4.443366226	-3.863680813
35	H35	-2.564514571	6.217100851	3.267584997
36	H36	-2.882979278	7.251223297	1.878692198
37	H37	-1.556871186	6.083183762	1.817008221
38	H38	-4.947907564	5.260793666	3.292385201
39	H39	-5.361426130	6.359877322	1.980566445
40	H40	-5.637720310	4.616739863	1.788248873
41	H41	-0.027609302	-1.523842515	-4.399322514
42	H42	-4.083463650	3.603393717	0.439144971
43	H43	1.152931905	-3.001875810	-5.981610869
44	H44	1.791914036	-5.112217196	-0.633704178
45	H45	0.996194714	-3.536808505	1.151634710
46	H46	-0.964574928	1.657638615	-2.752172970
47	H47	-2.222290123	3.432560809	2.369701198
48	H48	-2.072166973	4.187291942	-1.016283134
49	H49	-1.181843224	4.682553701	0.408021202
50	C50	-0.645614891	0.126999123	3.088888351
51	H51	-1.372976592	0.600919496	3.755491415
52	H52	-0.871491916	-0.941651512	3.072375738
53	C53	0.745145842	0.389924155	3.608684453
54	H54	0.967341685	1.446830482	3.758665246

55	C55	1.712512253	-0.495760779	3.863942505
56	C56	1.586522897	-1.987173868	3.695734437
57	H57	2.353587737	-2.353467386	3.000609536
58	H58	0.612954898	-2.296388057	3.311949341
59	H59	1.756399652	-2.499123957	4.651366491
60	C60	3.062058232	-0.041450864	4.360384469
61	H61	3.858143388	-0.363857230	3.676033738
62	H62	3.289324027	-0.484489739	5.339134334
63	H63	3.117216859	1.046807232	4.457830398

26 lowest-energy conformers used for Boltzmann-averaged ^{13}C NMR data.
Avg. Energy: -1461.229201 au.

V.7. Toxyloxanthone B (A23-1).[19]

C	Type	Theory	Expt.	diff.
C1	C	165.9 (-2.9)	165.1	0.8 (-2.9)
C2	CH	96.6	98.3	-1.7
C3	C	162	164.4	-2.4
C4	CH	91.4	93.5	-2.1
C4a	C	157.7	157.8	-0.1
C5	CH	102.4	103.1	-0.7
C6	C	152.2	153.7	-1.5
C7	C	136	138.4	-2.4
C8	C	124 (-2.9)	120.5	3.5 (-2.9)
C8a	C	109.8	108	1.8
C9	C	184.4	182.7	1.7
C9a	C	105	103.5	1.5
C10a	C	153.7	153.3	0.4
C1'	CH	124.9 (-3.7)	121.1	3.8 (-3.7)
C2'	CH	131.4 (+2.8)	133.2	-1.8 (+2.8)
C3'	C	76.8	76.4	0.4
C4'[2]	CH3	26.5	26.7	-0.2
RMSD		1.85		
Max Abs		3.81		
RMSD+CFx		1.43		
Max abs+CFx		2.43		

mol2 coordinates for lowest energy conformer

1	O1	-0.591750093	1.734345074	-1.680192923
2	C2	0.112009342	-1.962448763	1.774335413
3	C3	0.292387675	5.147887969	1.883413707
4	C4	-0.208490933	-2.023698849	0.405670841
5	C5	-0.020033107	5.287101234	0.529876448
6	C6	0.280859903	-0.734448336	2.381575185
7	C7	0.409396346	3.893948904	2.481757104
8	C8	-0.373164002	-0.884102320	-0.362847512
9	C9	-0.222172412	4.154537465	-0.250746605
10	O10	0.323843988	1.575773903	2.303959022
11	C11	-0.321130613	1.660895349	-0.467144557
12	C12	0.129696380	0.419672660	1.614438067
13	C13	0.202896070	2.777064194	1.690803758
14	C14	-0.191251963	0.388146360	0.249327630
15	C15	-0.113758968	2.858907591	0.325709545
16	O16	-0.398759313	-3.284053328	-0.081098899
17	C17	-0.101373225	-3.496449599	-1.481156214
18	C18	-0.655720089	-2.338757359	-2.278832862
19	C19	-0.759417036	-1.112662644	-1.759973764
20	C20	-0.799423738	-4.804407927	-1.833165645
21	C21	1.416574497	-3.602427921	-1.655202806
22	O22	0.249732671	-3.101764227	2.474698111
23	O23	-0.517289888	4.312306945	-1.539627616

24	O24	0.496936870	6.221121885	2.678324024
25	H25	-0.110985064	6.263518399	0.063970680
26	H26	0.535137499	-0.662956730	3.431737912
27	H27	0.652094539	3.797165540	3.531908219
28	H28	-0.938546916	-2.534376301	-3.309595547
29	H29	-1.121736642	-0.275763329	-2.339077925
30	H30	-1.878792658	-4.710998403	-1.686342207
31	H31	-0.425878808	-5.615825310	-1.200852845
32	H32	-0.606993659	-5.064430988	-2.878522184
33	H33	1.665079569	-3.765185124	-2.708518024
34	H34	1.810901019	-4.439298897	-1.068873158
35	H35	1.904229568	-2.678068778	-1.329942980
36	H36	0.102516657	-3.831175083	1.851099027
37	H37	-0.617537827	3.402978766	-1.920319767
38	H38	0.389914363	7.027927980	2.159429349

2 lowest-energy conformers used for Boltzmann-averaged ^{13}C NMR data.
Avg. Energy: -1145.756536 au.

V.8. Elliptoxanthone B (A23-2).[21]

No	type	δcal	δexp	diff
C1	C	155.5 (-2.9)	153.9	1.6 (-2.9)
C2	CH	110.9	108.7	2.2
C3	CH	123.0	122.3	0.7
C4	C	134.7	136.7	-2.0
C4a	C	142.2	143.5	-1.3
C5	CH	101.6	102.7	-1.1
C6	C	152.7	153.5	-0.8
C7	C	136.1	138.3	-2.2
C8	C	125.1 (-2.9)	120.1	5.0 (-2.9)
C8a	C	109.9	107.9	2.0
C9	C	185.6	183.5	2.1
C9a	C	110.0	109.2	0.8
C10a	C	153.3	153.5	-0.2
C1'	CH	124.8 (-3.7)	120.6	4.2 (-3.7)
C2'	CH	132.4 (+2.8)	133	-0.6 (+2.8)
C3'	C	76.8	76.1	0.7
C4'[2]	CH3	26.2	26.3	-0.1
RMSD		2.01		
Max Abs		4.97		
RMSD+CFx		1.47		
Max abs+CFx		2.21		

mol2 coordinates for lowest energy conformer

1	O1	-0.244441545	-1.888276821	-1.767742896
2	C2	-0.358841676	-5.184678044	2.014099193
3	C3	0.740025895	1.832762597	1.587405435
4	C4	-0.531815338	-5.351241572	0.643872305
5	C5	0.589106487	1.860249262	0.188847363
6	C6	-0.099288392	-3.935288437	2.563565558
7	C7	0.620230951	0.635208464	2.263756995
8	C8	-0.448447476	-4.261926814	-0.219725032
9	C9	0.335337757	0.715024177	-0.546985323
10	O10	0.239843144	-1.648052821	2.287950966
11	C11	-0.087555951	-1.796315119	-0.540582060
12	C12	-0.015819779	-2.848925171	1.697688669
13	C13	0.349892042	-0.520797735	1.534152974
14	C14	-0.182539388	-2.976174342	0.314543489
15	C15	0.199355317	-0.526144913	0.138621794
16	C16	0.262065943	0.902359639	-2.001321567
17	C17	0.144706717	2.136243967	-2.498436518

18	C18	0.034842731	3.345645986	-1.599451228
19	O19	0.773953491	3.085094081	-0.382758890
20	O20	0.998788856	2.973403779	2.248260046
21	C21	0.712249319	4.563314168	-2.214235949
22	C22	-1.426627327	3.632130676	-1.244905977
23	O23	-0.622222518	-4.460317071	-1.532469934
24	O24	0.065057589	-3.794956489	3.906781355
25	H25	-0.424509667	-6.034968855	2.684817546
26	H26	-0.734264094	-6.329551757	0.223766741
27	H27	0.724302758	0.594209502	3.340989260
28	H28	0.304511380	0.031696476	-2.639252056
29	H29	0.091432775	2.303165548	-3.570878383
30	H30	1.027438731	3.680465929	1.582949680
31	H31	0.672022261	5.410330080	-1.522386198
32	H32	1.759390903	4.342278807	-2.441589536
33	H33	0.203024363	4.849147650	-3.140418253
34	H34	-1.875925984	2.771474190	-0.740428169
35	H35	-2.002483679	3.830985228	-2.154600979
36	H36	-1.496248243	4.506490901	-0.588974607
37	H37	-0.535043054	-3.581709531	-1.966439942
38	H38	0.238494700	-2.862355612	4.091514131

1 lowest-energy conformers used for Boltzmann-averaged ^{13}C NMR data.
Avg. Energy: -1145.748028 au.

V.9. Cudraxanthone B (A23-3).[17]

No	type	δcal	δexp	diff
C1	C	164.6 (-2.9)	162	2.6 (-2.9)
C2	CH	101.0	100.2	0.8
C3	C	163.8	161.8	2.0
C4	C	105.1	108.2	-3.1
C4a	C	155.8	155.2	0.6
C5	CH	102.0	101.9	0.1
C6	C	152.1	152.6	-0.5
C7	C	136.0	136.9	-0.9
C8	C	124.0 (-2.9)	119.6	4.4 (-2.9)
C8a	C	109.5	109	0.5
C9	C	184.8	182.8	2.0
C9a	C	105.5	104.6	0.9
C10a	C	153.1	151	2.1
C1'	C	42.0	40.9	1.1
C2'[2]	CH3	29.1	28	1.1
C4'	CH	149.5	149.3	0.2
C5'	CH2	114.3	113.3	1.0
C1''	CH	125.1 (-3.7)	120.9	4.2 (-3.7)
C2''	CH	131.3 (+2.8)	132.4	-1.1 (+2.8)
C3''	C	76.9	76.9	0.0
C4''[2]	CH3	26.5	27.3	-0.8
RMSD		1.82		
Max Abs		4.36		
RMSD+CFx		1.27		
Max abs+CFx		3.10		

mol2 coordinates for lowest energy conformer

1	C1	1.666962977	-4.827297586	3.937529604
2	C2	1.117379649	-1.546731082	3.285698919
3	C3	-0.744230941	-3.195313571	3.430224964
4	O4	-0.574475223	-0.642126782	-3.014025856
5	O5	0.083517969	-5.311082506	1.158575409
6	O6	0.194216259	4.336089947	-1.480433909
7	O7	-0.710215845	-3.203112123	-2.890990954

8	C8	1.679396618	-3.872217642	3.006974353
9	C9	-0.288105940	-4.213934894	-0.826666293
10	C10	-0.060951315	1.947541441	-1.737838530
11	O11	0.732994610	4.112593319	1.098892233
12	O12	0.358706944	-0.549518648	0.940574663
13	C13	0.046339288	-4.121551222	0.522837932
14	C14	0.180729545	3.071831492	-0.967132847
15	C15	-0.394020085	-3.076223178	-1.603482075
16	C16	0.550770467	1.751604237	1.014862634
17	C17	0.493318756	2.987246160	0.402422998
18	C18	0.300378338	-2.887758509	1.176149285
19	C19	-0.272368427	-0.593267709	-1.807336947
20	C20	-0.014317501	0.669587995	-1.113567496
21	C21	0.292529721	0.614255861	0.249197453
22	C22	0.168075471	-1.759259759	0.357097043
23	C23	-0.159052430	-1.805205829	-1.012113005
24	C24	0.572059515	-2.871460824	2.699182516
25	H25	2.537237989	-5.453232184	4.105838560
26	H26	0.801444550	-5.017505125	4.565769995
27	H27	2.009247966	-1.198499353	2.759052523
28	H28	1.386052226	-1.730505546	4.331101321
29	H29	0.369413089	-0.754523299	3.257225025
30	H30	-0.604474675	-3.194906112	4.517545245
31	H31	-1.486917611	-2.429518136	3.186175278
32	H32	-1.154617619	-4.165022116	3.129996848
33	H33	0.558178441	-5.233746687	2.002889929
34	H34	-0.741727840	-2.287808822	-3.265953705
35	H35	2.580680739	-3.731958010	2.410105175
36	H36	-0.459963069	-5.189647806	-1.263509548
37	C37	-0.313272484	2.196293069	-3.161707480
38	H38	-0.276348629	1.360098212	-3.844926424
39	C39	-0.578538307	3.438171901	-3.574951595
40	H40	-0.763162682	3.650732068	-4.624392501
41	C41	-0.681600020	4.592222667	-2.603967960
42	C42	-2.115700770	4.746861226	-2.089223270
43	H43	-2.179279410	5.580837183	-1.382199887
44	H44	-2.444887704	3.830179553	-1.589873791
45	H45	-2.796528318	4.942167791	-2.923937717
46	C46	-0.162709363	5.887540094	-3.214515746
47	H47	0.867756167	5.760830558	-3.558360189
48	H48	-0.192633151	6.693925511	-2.475289352
49	H49	-0.785178445	6.176759929	-4.067493674
50	H50	0.783125816	1.663784550	2.069043740
51	H51	0.624764692	4.851780294	0.478927104

4 lowest-energy conformers used for Boltzmann-averaged ^{13}C NMR data.
Avg. Energy: -1341.093935 au.

V.10. Cudraxanthone K (A23-4).[17]

No	type	δcal	δexp	diff
C1	C	164.8 (-2.9)	163.3	1.5 (-2.9)
C2	C	110.1	113	-2.9
C3	C	164.3	162.4	1.9
C4	CH	95.0	94.6	0.4
C4a	C	156.4	155.4	1.0
C5	CH	102.4	102.3	0.1
C6	C	152.2	152.9	-0.7
C7	C	136.0	136.8	-0.8
C8	C	124.1 (-2.9)	119.8	4.3 (-2.9)
C8a	C	109.7	108.5	1.2
C9	C	184.9	182.8	2.1

C9a	C	104.6	103.8	0.8
C10a	C	153.6	151	2.6
C1'	C	42.1	41.1	1.0
C2'²	CH3	28.2	27.4	0.8
C4'	CH	150.6	149.9	0.7
C5'	CH2	113.6	113.3	0.3
C1''	CH	125.3 (-3.7)	121.1	4.2 (-3.7)
C2''	CH	131.1 (+2.8)	132.2	-1.1 (+2.8)
C3''	C	76.7	76.9	-0.2
C4''²	CH3	26.4	27.4	-1.0
RMSD		1.77		
Max Abs		4.29		
RMSD+CFx		1.31		
Max abs+CFx		2.93		

mol2 coordinates for lowest energy conformer

1	O1	0.624541790	0.276472439	-1.612586158
2	C2	-0.402473808	-3.217652753	1.833053916
3	C3	-0.223936977	3.896984527	1.873240510
4	C4	0.008677240	-3.380372703	0.484998662
5	C5	0.157756602	3.979728375	0.521377968
6	C6	-0.543380203	-1.969949040	2.440936777
7	C7	-0.412030209	2.659647307	2.454615095
8	C8	0.212030281	-2.197208325	-0.244216979
9	C9	0.368980909	2.852203114	-0.254115430
10	O10	-0.445374754	0.350535434	2.333885533
11	C11	0.316244516	0.309864277	-0.404528967
12	C12	-0.295650168	-0.836784711	1.699716116
13	C13	-0.221343835	1.518036274	1.677217302
14	C14	0.085388994	-0.906325503	0.354274319
15	C15	0.161946138	1.569968707	0.330121563
16	O16	-0.716338312	-4.250375767	2.640297097
17	C17	0.823375234	-5.723637448	0.816929695
18	C18	0.072600696	-4.796944112	-0.131798994
19	C19	0.434934992	-6.926659788	1.243840562
20	C20	-1.365780435	-5.269462828	-0.409047058
21	C21	0.879961321	-4.913377960	-1.447938122
22	O22	0.519075190	-2.259030568	-1.540694169
23	O23	0.359990586	5.248518331	0.061556011
24	O24	-0.400488434	5.025697207	2.582532881
25	C25	0.836696158	3.105627375	-1.622077285
26	C26	0.751383978	4.338369377	-2.128698795
27	C27	0.128578050	5.473136720	-1.349293098
28	C28	-1.380194817	5.546714267	-1.599596542
29	C29	0.811739741	6.801237046	-1.648431368
30	H30	-0.847217969	-1.907661685	3.477752348
31	H31	-0.714227855	2.570662237	3.490710105
32	H32	-0.408901558	-5.091344887	2.260990579
33	H33	1.802361111	-5.349966158	1.119882468
34	H34	-0.526365412	-7.356903759	0.975839666
35	H35	1.079017332	-7.529776611	1.876627933
36	H36	-1.843012647	-4.572712225	-1.103909595
37	H37	-1.970317884	-5.301153681	0.503610242
38	H38	-1.375085090	-6.263903185	-0.869084979
39	H39	1.883428043	-4.491142461	-1.347964217
40	H40	0.974289438	-5.977090428	-1.691415984
41	H41	0.382715584	-4.404363435	-2.271847955
42	H42	0.626069765	-1.321467026	-1.857080951
43	H43	-0.230110724	5.764896673	1.976676044
44	H44	1.246860288	2.283210509	-2.189633868

45	H45	1.099450143	4.554476603	-3.135090167
46	H46	-1.862356700	4.606964386	-1.313869967
47	H47	-1.577423154	5.720606899	-2.662087047
48	H48	-1.823861428	6.364901969	-1.022216060
49	H49	0.384616574	7.595653815	-1.028870649
50	H50	1.883805133	6.732744675	-1.444548548
51	H51	0.669356543	7.068408506	-2.700040442

2 lowest-energy conformers used for Boltzmann-averaged ^{13}C NMR data.
Avg. Energy: -1341.095707 au.

V.11. Cudracuspixanthone P (A23-5).[22]

C	Type	Theory	Expt.	diff.
C1	C	163.5 (-2.9)	161.0	2.5 (-2.9)
C2	CH	99.5	98.5	1.0
C3	C	161.2	160.7	0.5
C4	C	97.1	99.0	-1.9
C4a	C	155.0	154.1	0.9
C5	CH	102.3	102.7	-0.4
C6	C	151.9	153.2	-1.3
C7	C	136.0	138.1	-2.1
C8	C	124.1 (-2.9)	119.9	4.2 (-2.9)
C8a	C	110.0	107.6	2.4
C9	C	184.5	182.4	2.1
C9a	C	105.4	103.3	2.1
C10a	C	153.6	152.8	0.8
C1'	CH2	17.3	15.8	1.5
C2'	CH2	31.3	31.4	-0.1
C3'	C	74.8	75.9	-1.1
C4'[2]	CH3	26.4	26.0	0.4
C1''	CH	125.2 (-3.7)	120.6	4.6 (-3.7)
C2''	CH	131.1 (+2.8)	132.7	-1.6 (+2.8)
C3''	C	76.9	76.0	0.9
C4''[2]	CH3	26.5	26.2	0.3
RMSD		1.86		
Max Abs		4.58		
RMSD+CFx		1.26		
Max abs+CFx		2.44		

mol2 coordinates for lowest energy conformer

1	C1	4.239583366	0.911345709	-4.009768337
2	C2	5.141827508	-1.351032387	-4.674342316
3	O3	-1.059555652	-0.596971443	-2.731594782
4	C4	1.620562519	-1.045495394	-3.589131754
5	C5	2.669336888	-0.998868427	-4.414729341
6	C6	3.464564492	-0.603457284	-0.368036052
7	C7	2.467093879	-0.395755537	0.562763308
8	C8	-1.917442898	0.137387823	1.943913637
9	C9	-2.970302859	-0.154980112	-0.674616644
10	O10	4.186736367	-1.021124460	-2.547618809
11	O11	-4.201768810	0.424859472	2.659882047
12	O12	0.236130038	-0.139897374	1.117214321
13	C13	-3.816847938	0.072820000	0.397346045
14	C14	3.127069017	-0.781559026	-1.722302333
15	C15	-3.292515719	0.219208887	1.684419024
16	C16	1.813272398	-0.770044182	-2.160345235
17	C17	-0.646177237	-0.468637276	-1.565175037
18	C18	1.142176078	-0.363536360	0.128998058
19	C19	-1.092400644	-0.079863853	0.845453154
20	C20	-1.563314615	-0.233146827	-0.465199899
21	C21	0.776851968	-0.540933884	-1.212050272

22	C22	4.044260873	-0.605098404	-3.927344626
23	O23	-3.494298239	-0.289254077	-1.894509110
24	C24	-1.370639174	0.246981504	3.343174842
25	C25	-2.500798006	0.062364380	4.353588891
26	C26	-3.745314645	0.862428885	3.958859223
27	C27	-4.909441292	0.559047714	4.891816232
28	C28	-3.475818669	2.366985644	3.890293066
29	O29	4.756688451	-0.638537036	0.003872288
30	H30	3.480922004	1.428758425	-3.415013306
31	H31	4.145181993	1.246874790	-5.047606613
32	H32	5.232941684	1.188148022	-3.640489378
33	H33	6.123342626	-1.088442232	-4.268211373
34	H34	4.999782478	-2.431587829	-4.583763901
35	H35	5.122780930	-1.080903532	-5.734711331
36	H36	0.627174766	-1.285760135	-3.939800300
37	H37	2.556598352	-1.212534898	-5.474151429
38	H38	2.704151358	-0.246856634	1.609011442
39	H39	-4.886119778	0.142854721	0.240393778
40	H40	-2.737909821	-0.432333018	-2.513903926
41	H41	-0.596600263	-0.510366186	3.503086463
42	H42	-0.878243326	1.217297750	3.489456512
43	H43	-2.178541457	0.363955882	5.355785287
44	H44	-2.781196960	-0.996605036	4.403882438
45	H45	-5.812295318	1.076821187	4.554417876
46	H46	-5.113546128	-0.515367398	4.911457351
47	H47	-4.675641046	0.890890334	5.908368341
48	H48	-3.114188937	2.734170989	4.856744668
49	H49	-4.396448048	2.900409745	3.635943321
50	H50	-2.726907873	2.605140071	3.128793909
51	H51	5.275245317	-0.779801691	-0.804519418

2 lowest-energy conformers used for Boltzmann-averaged ^{13}C NMR data.
Avg. Energy: -1341.140631au.

V.12. Garcinone B (A23-6).[23]

No	type	δcal	δexp	diff
C1	C	162.3 (-2.9)	159.7	2.6 (-2.9)
C2	C	105.5	109.7	-4.2
C3	C	164.3	162.4	1.9
C4	CH	93.9	92.4	1.5
C4a	C	156.5	154.3	2.2
C5	CH	102.6	102.7	-0.1
C6	C	152.1	153.2	-1.1
C7	C	135.9	138.1	-2.2
C8	C	124.0 (-2.9)	119.7	4.3 (-2.9)
C8a	C	109.8	106.8	3.0
C9	C	184.6	181.5	3.1
C9a	C	104.4	102.1	2.3
C10a	C	153.9	152.3	1.6
C1'	CH2	22.7	21	1.7
C2'	CH	126.6	122.5	4.1
C3'	C	136.3	130.4	5.9
C4'	CH3	16.8	17.8	-1.0
C5'	CH3	25.5	25.6	-0.1
C1''	CH	125.3 (-3.7)	120.4	4.9 (-3.7)
C2''	CH	131.1 (+2.8)	132.6	-1.5 (+2.8)
C3''	C	76.7	75.1	1.6
C4''[2]	CH3	26.5	26.8	-0.3
RMSD		2.72		
Max Abs		5.94		
RMSD+CFx		2.32		

Max abs+CFx 5.94

mol2 coordinates for lowest energy conformer

1	C1	-5.999850109	-0.831100263	-1.854806790
2	C2	-6.935486430	1.500050067	-2.115019162
3	O3	-0.489707071	0.419541813	-1.907498681
4	C4	4.335128470	-0.739425095	-0.312234049
5	C5	-3.272353895	1.013228939	-2.003482547
6	C6	5.400261016	0.104104689	0.346164500
7	C7	-4.499174323	1.059038236	-2.530147510
8	C8	-4.246568939	0.368318080	1.547017828
9	C9	-3.055650063	0.055526662	2.170052282
10	C10	1.513606589	-0.706706694	2.294006255
11	C11	1.860109209	-0.241384144	-0.452697952
12	O12	-5.481284318	0.984979140	-0.336282998
13	O13	3.797163935	-1.115415540	2.513996040
14	O14	-0.773446434	-0.309947379	2.109115245
15	C15	2.971104204	-0.574051824	0.323414628
16	C16	-4.259025286	0.646069016	0.167499422
17	C17	2.772379057	-0.792899139	1.699208436
18	C18	-3.104451795	0.631027945	-0.596585455
19	C19	-0.594009115	0.211557823	-0.683348070
20	C20	-1.888988919	0.019346950	1.406710682
21	C21	0.433337601	-0.383152802	1.496063020
22	C22	0.562281588	-0.133691435	0.121745315
23	C23	-1.871773969	0.294898543	0.031463762
24	C24	-5.719440603	0.668738825	-1.728232695
25	C25	7.562818872	0.723847057	1.384977579
26	C26	6.613676931	-0.275889058	0.773041927
27	C27	7.171292466	-1.672048927	0.659820427
28	O28	2.034664305	-0.032861291	-1.761162505
29	H29	-6.190016666	-1.091283592	-2.900763975
30	H30	-6.876939374	-1.107369792	-1.259436640
31	H31	-5.138548322	-1.411757348	-1.511470618
32	H32	-7.791268969	1.237462257	-1.485621352
33	H33	-7.202393762	1.308972338	-3.158480290
34	H34	-6.723092589	2.565573191	-1.994937500
35	H35	4.252429424	-0.450601199	-1.364326819
36	H36	4.614378676	-1.799664780	-0.305673941
37	H37	-2.392445394	1.254242522	-2.582527797
38	H38	5.123499991	1.152425337	0.473551173
39	H39	-4.653013227	1.350273726	-3.566011168
40	H40	-3.023101509	-0.170556987	3.228658673
41	H41	1.393316191	-0.880449384	3.355660332
42	H42	4.637390236	-0.912118752	2.068293230
43	H43	8.473749691	0.809324947	0.779380242
44	H44	7.114731484	1.717400121	1.468279377
45	H45	7.878229226	0.402674877	2.385343216
46	H46	6.439459325	-2.411025701	0.326799905
47	H47	8.004170016	-1.682618989	-0.054113651
48	H48	7.575488839	-2.003900050	1.622382827
49	H49	1.141515434	0.184794384	-2.134673835
50	O50	-5.397588107	0.411500896	2.241283338
51	H51	-6.096563584	0.635001782	1.605606338

2 lowest-energy conformers used for Boltzmann-averaged ^{13}C NMR data.
Avg. Energy: -1341.111126 au.

V.13. Allanxanthone B (A23-8).[24]

No	type	δcal	δexp	diff
C1	C	162.2 (-2.9)	161.5	0.7 (-2.9)

C2	C	107.6	111.0	-3.4
C3	C	163.4	163	0.4
C4	CH	94.8	93	1.8
C4a	C	156.1	158.2*	-2.1
C5	CH	102.5	102.1	0.4
C6	C	152.2	153.5*	-1.3
C7	C	135.9	144.1	-8.2
C8	C	124.1 (-2.9)	119.2	4.9 (-2.9)
C8a	C	109.8	109.1	0.7
C9	C	184.7	181.5	3.2
C9a	C	105.2	103	2.2
C10a	C	153.8	156.2*	-2.4
C1'	CH2	22.7	22	0.7
C2'	CH	126.6	123.1	3.5
C3'	C	136.3	135.2	1.1
C4'	CH3	14.9	16.4	-1.5
C5'	CH2	39.6	39.7	-0.1
C6'	CH2	26.1	26.5	-0.4
C7'	CH	124.3	124.1	0.2
C8'	C	135.1	131.3	3.8
C9'	CH3	17.4	17.7	0.3
C10'	CH3	25.9	25.6	0.3
C1''	CH	125.2 (-3.7)	122	3.2 (-3.7)
C2''	CH	131.3 (+2.8)	131.8	-0.5 (+2.8)
C3''	C	76.7	79.5	-2.8
C4''[2]	CH3	26.4	30.1	-3.7
RMSD		2.74		
Max Abs		8.17		
RMSD+CFx		2.60		
Max abs+CFx		8.17		

mol2 coordinates for lowest energy conformer

1	C1	3.818810705	-1.261462081	4.875608100
2	C2	1.559980491	-0.136719055	4.720199706
3	C3	5.923668602	-0.099080100	1.578805476
4	C4	-5.560789649	-1.308459627	-5.152867648
5	C5	-7.221011564	0.563333311	-4.811495101
6	O6	-0.997274472	1.129326888	-2.885130900
7	O7	2.635970980	-1.193640941	1.768145425
8	O8	-6.252717134	-2.022800580	-0.949486024
9	O9	1.442360473	1.283465991	-2.099130498
10	C10	-3.729885954	0.996440279	-3.674517004
11	C11	-4.762620857	0.980691151	-4.521355983
12	C12	3.607960295	1.193706076	4.319917125
13	C13	4.018496136	1.362653992	0.790327577
14	C14	0.441762702	-1.312596485	0.973657176
15	C15	-3.976123208	-1.721434781	-0.367787848
16	C16	5.059287228	1.539866382	4.150483275
17	C17	3.531834478	0.523253098	-0.364325414
18	C18	5.350599425	2.096441497	2.740158690
19	O19	-6.049312961	-0.344289205	-2.982349849
20	O20	-1.751534400	-1.448716344	0.200937012
21	C21	3.056361447	0.012846409	4.621756966
22	C22	5.036938194	1.120632342	1.628848732
23	C23	1.729562528	-0.795691800	0.847100070
24	C24	-5.057347877	-1.452250830	-1.182019335
25	C25	1.110186771	0.453003369	-1.106415049
26	C26	-3.705702449	0.055463530	-2.548175057
27	C27	2.096834126	0.069940294	-0.197398508
28	C28	-4.908255167	-0.571158255	-2.268892165

29	C29	-1.248575827	0.349474813	-1.946722186
30	C30	-0.508924870	-0.929421078	0.046253536
31	C31	-2.754892661	-1.110566698	-0.650424958
32	C32	-0.221024002	-0.039511336	-0.998335338
33	C33	-2.578698963	-0.226244332	-1.724634632
34	C34	-5.882634034	-0.024250995	-4.383406345
35	H35	3.541972663	-2.014203817	4.127519317
36	H36	4.903791997	-1.134028927	4.849608577
37	H37	3.555117941	-1.674573369	5.857212792
38	H38	1.041061319	0.805436108	4.521069234
39	H39	1.265828479	-0.487493900	5.717869763
40	H40	1.207901790	-0.880947487	3.996457005
41	H41	5.798831189	-0.709584693	2.482088999
42	H42	5.727712913	-0.741785546	0.718801409
43	H43	6.978003043	0.200084023	1.543348166
44	H44	-4.629770142	-1.751446792	-4.786422767
45	H45	-6.371311400	-2.036214545	-5.038057799
46	H46	-5.435971455	-1.087936771	-6.217651181
47	H47	-8.023152313	-0.163876914	-4.652431186
48	H48	-7.443498322	1.465471547	-4.234976862
49	H49	-7.194818441	0.822079858	-5.874492568
50	H50	3.288084843	-0.483119110	1.900269646
51	H51	-6.852577221	-1.706673687	-1.644117858
52	H52	0.622275335	1.422759647	-2.639262955
53	H53	-2.906401475	1.686170958	-3.789922978
54	H54	-4.809558907	1.671359320	-5.358913461
55	H55	2.928887577	2.031078692	4.146976987
56	H56	3.441516231	2.273929857	0.948422335
57	H57	0.191552989	-1.983597406	1.785636076
58	H58	-4.064905120	-2.402989728	0.469067939
59	H59	5.337256979	2.312820724	4.879402244
60	H60	5.707562473	0.682396323	4.355379316
61	H61	3.594500833	1.113558313	-1.282849379
62	H62	4.167860054	-0.356420135	-0.510249998
63	H63	4.768555810	3.014277493	2.596022002
64	H64	6.410401806	2.381225066	2.686868163

6 lowest-energy conformers used for Boltzmann-averaged ^{13}C NMR data.
Avg. Energy: -1536.461983 au.

V.14. Brasilixanthone B (A23-9–B31-18).[25]

No	type	δcal	δexp	diff
C1	C	160.7 (-2.9)	159.6	1.1 (-2.9)
C2	C	105.2	104.5	0.7
C3	C	161.1	158.6	2.5
C4	CH	94.4	94.3	0.1
C4a	C	157.1	155.1	2.0
C5	CH	102.5	102.4	0.1
C6	C	152.2	150.9	1.3
C7	C	135.9	135.8	0.1
C8	C	124.0 (-2.9)	121.5	2.5 (-2.9)
C8a	C	109.8	110.1	-0.3
C9	C	184.6	182.1	2.5
C9a	C	105.1	103.8	1.3
C10a	C	153.7	152.5	1.2
C1'	CH	120.8 (-3.7)	115.7	5.1 (-3.7)
C2'	CH	123.5 (+2.8)	127.2	-3.7 (+2.8)
C3'	C	78.3	77.4	0.9
C4'[2]	CH3	28.1	28.4	-0.3
C1''	CH	125.0 (-3.7)	121.0	4.0 (-3.7)
C2''	CH	131.3 (+2.8)	132.3	-1.0 (+2.8)

C3''	C	76.9	77.4	-0.5
C4''2	CH3	26.5	27.4	-0.9
RMSD		1.97		
Max Abs		5.10		
RMSD+CFx		1.23		
Max abs+CFx		2.55		

mol2 coordinates for lowest energy conformer

1	C1	2.365406914	-0.077765810	-0.641327621
2	C2	3.458430421	-0.290256217	0.199517032
3	C3	3.228329021	-0.446256896	1.578455535
4	C4	1.948203036	-0.414855230	2.124689083
5	C5	-2.650936294	0.030777923	1.792613843
6	C6	-3.836312211	0.221781966	1.111759600
7	C7	-3.816397704	0.412085787	-0.282357126
8	C8	-2.636655343	0.428316443	-1.005965786
9	C9	-0.100631500	0.188457680	-0.980329657
10	C10	1.045510375	-0.024524578	-0.115229306
11	C11	-1.457619739	0.029469170	1.071929076
12	O12	0.040341022	0.330549815	-2.209342011
13	C13	-5.409666240	-1.289323493	-2.267011559
14	O14	4.246033788	-0.690015495	2.423333558
15	O15	-5.038837808	0.638196504	-0.844797031
16	O16	-0.348788203	-0.178174504	1.831006961
17	O17	-5.012783323	0.227920927	1.763142708
18	C18	5.742920629	1.197432540	2.418015527
19	C19	-6.451140270	0.972625050	-2.696733065
20	C20	6.500175691	-1.162853770	2.903433648
21	C21	4.821206577	-0.450002946	-0.290976473
22	O22	2.589793001	0.065305304	-1.947826622
23	C23	-2.777209640	0.699493723	-2.441437318
24	C24	-3.984531101	0.632163777	-3.008298363
25	C25	0.881809880	-0.207258614	1.266635864
26	C26	5.831467432	-0.494198155	0.578538476
27	C27	-1.407869107	0.220112050	-0.317039348
28	C28	5.590273432	-0.283117412	2.054447948
29	C29	-5.209965544	0.228114939	-2.222044175
30	H30	5.038491841	1.804283231	1.840597474
31	H31	5.550855124	1.348333953	3.485431209
32	H32	6.757171600	1.540476366	2.188724142
33	H33	7.548288145	-0.898653096	2.728500874
34	H34	6.277512485	-1.023549440	3.965362240
35	H35	6.356075626	-2.216881676	2.648503014
36	H36	-6.292388986	2.053168576	-2.637538004
37	H37	-6.677377685	0.705708626	-3.734305660
38	H38	-7.312528843	0.707760616	-2.076062172
39	H39	-6.296465856	-1.574652829	-1.691173210
40	H40	-4.536426393	-1.803077493	-1.853653012
41	H41	-5.541494196	-1.622198859	-3.301859201
42	H42	-5.700690546	0.362174847	1.091264657
43	H43	1.704288352	0.190898000	-2.373772092
44	H44	4.973960754	-0.559088399	-1.359070941
45	H45	-1.892700175	0.950420084	-3.008863448
46	H46	6.858471610	-0.641308929	0.257261512
47	H47	-4.116289403	0.832847488	-4.068079062
48	H48	-2.642503705	-0.126299866	2.864049642
49	H49	1.793193062	-0.544561677	3.187878639

1 lowest-energy conformers used for Boltzmann-averaged ^{13}C NMR data.
Avg. Energy: -1339.909613 au.

V.15. Brasilixanthone A (A23-11–D31-8).[25]

No	type	δcal	δexp	diff
C1	C	165.5 (-2.9)	163.3	2.2 (-2.9)
C2	CH	99.4	99.3	0.1
C3	C	161.1	160.2	0.9
C4	C	100.5	100.6	-0.1
C4a	C	152.8	151.4	1.4
C5	CH	102.3	102.5	-0.2
C6	C	152.2	151.2	1.0
C7	C	136.0	137.1	-1.1
C8	C	124.3 (-2.9)	120	4.3 (-2.9)
C8a	C	109.8	108.7	1.1
C9	C	184.6	182.6	2.0
C9a	C	105.2	104	1.2
C10a	C	153.5	153.1	0.4
C1'	CH	119.5 (-3.7)	115.3	4.2 (-3.7)
C2'	CH	123.9 (+2.8)	127	-3.1 (+2.8)
C3'	C	78.0	78.2	-0.2
C4'[2]	CH3	27.9	27.5	0.4
C1''	CH	124.9 (-3.7)	121.1	3.8 (-3.7)
C2''	CH	131.7 (+2.8)	132.6	-0.9 (+2.8)
C3''	C	76.9	77.2	-0.3
C4''[2]	CH3	26.4	27.5	-1.1
RMSD		1.88		
Max Abs		4.30		
RMSD+CFx		0.95		
Max abs+CFx		1.97		

mol2 coordinates for lowest energy conformer

1	C1	-4.752636013	-1.282553184	3.675061160
2	C2	-5.025794318	0.946282981	4.830377831
3	C3	4.789865601	1.260969294	-3.100832378
4	C4	5.337446468	-0.892212059	-4.304265142
5	O5	-0.840182569	0.504141467	-2.671935621
6	O6	4.534187596	-1.304942418	0.355958762
7	O7	-3.257671092	1.070029099	-1.957410327
8	C8	-1.765152834	0.092848933	3.361095587
9	C9	1.825873863	-0.168317668	-3.422333095
10	C10	-2.740482497	0.077454819	4.270701424
11	C11	2.960122972	-0.110519711	-4.124894244
12	C12	-3.796807891	0.927506578	0.315517613
13	C13	2.268988499	-0.767385675	0.800223479
14	O14	-4.337173224	0.824815935	2.584582383
15	O15	4.209913156	-0.928755348	-2.238775456
16	O16	0.062521795	-0.235337954	1.244729823
17	C17	3.319367706	-0.928956163	-0.080933303
18	C18	-2.868958507	0.807243129	-0.711126449
19	C19	-3.412568133	0.649097054	1.623468783
20	C20	-2.102954399	0.256659842	1.953798332
21	C21	1.886913484	-0.321613044	-1.963964326
22	C22	3.117984233	-0.697433713	-1.453715341
23	C23	-0.545430092	0.269264921	-1.485785964
24	C24	1.029171415	-0.375230095	0.298459555
25	C25	-1.194330595	0.140330266	0.906057202
26	C26	-1.533191250	0.397683964	-0.429234612
27	C27	0.798039389	-0.143585971	-1.064641291
28	C28	-4.190112358	0.131402190	3.850182848
29	C29	4.313586373	-0.150716858	-3.454293132
30	H30	-5.800091862	-1.237966306	3.359616328
31	H31	-4.177871507	-1.828517080	2.920548829

32	H32	-4.690061390	-1.834746303	4.618430410
33	H33	-6.059592191	1.013795786	4.479751075
34	H34	-4.620472277	1.957331598	4.927070305
35	H35	-5.021521027	0.469230761	5.815499941
36	H36	5.768792335	1.222249924	-2.611222266
37	H37	4.075455949	1.749583866	-2.431027196
38	H38	4.875372057	1.868470327	-4.007264304
39	H39	6.292823962	-0.959815576	-3.774776999
40	H40	5.501087631	-0.359469183	-5.245987689
41	H41	4.985827314	-1.902918552	-4.528020633
42	H42	5.110524096	-1.340641485	-0.424745219
43	H43	-2.468741071	0.928795218	-2.538062356
44	H44	-0.720327521	0.019295023	3.642621507
45	H45	0.856447552	-0.102573500	-3.894477255
46	H46	-2.531228773	-0.013094578	5.332580094
47	H47	2.942695992	0.002810631	-5.205451111
48	H48	-4.812679275	1.231629467	0.096579215
49	H49	2.403023226	-0.931620651	1.862263224

1 lowest-energy conformers used for Boltzmann-averaged ^{13}C NMR data.
Avg. Energy: -1339.908620 au.

V.16. Nigrolineaxanthone W (A23-12–B31-21).[27] Also call Dulcisxanthone D[29] and Tovophyllin B[28]

C	Type	Theory	Expt.	diff.
C1	C	160.7 (-2.9)	157.8*	2.9 (-2.9)
C2	C	105.2	103.8	1.4
C3	C	161.0	159.8*	1.2
C4	CH	94.6	94.2	0.4
C4a	C	157.2	156.5	0.7
C5	C	115.9	115.3	0.6
C6	C	149.5	148.6	0.9
C7	C	135.9	136.6	-0.7
C8	C	121.5 (-2.9)	117.1	4.4 (-2.9)
C8a	C	110.1	108.4	1.7
C9	C	185.0	182.8	2.2
C9a	C	105.2	104.3	0.9
C10a	C	152.0	150.9	1.1
C1'	CH	120.9 (-3.7)	115.7	5.2 (-3.7)
C2'	CH	123.6 (+2.8)	127.1	-3.5 (+2.8)
C3'	C	78.0	77.9	0.1
C4'[2]	CH3	27.9	28.0	-0.1
C1''	CH2	24.1	22.6	1.5
C2''	CH	123.2	120.9	2.3
C3''	C	131.9	132.7	-0.8
C4''	CH3	25.5	25.8	-0.3
C5''	CH3	18.4	18.0	0.4
C1'''	CH	125.3 (-3.7)	121.0	4.3 (-3.7)
C2'''	CH	130.4 (+2.8)	131.4	-1.0 (+2.8)
C3'''	C	76.8	76.9	-0.1
C4'''[2]	CH3	26.5	27.4	-0.9

RMSD	2.00
Max Abs	5.22
RMSD+CFx	1.10
Max abs+CFx	2.28

mol2 coordinates for lowest energy conformer

1	C1	1.007682473	-0.517612637	-1.333460353
2	C2	0.974606947	-0.735859119	0.052111263
3	O3	-0.140961222	-0.515687533	0.799677097
4	C4	-1.275933326	-0.037952999	0.242037342
5	C5	-1.342729090	0.233015253	-1.132168086

6	C6	-0.202092783	-0.016754049	-1.993697200
7	O7	-0.269047786	0.191446365	-3.219932740
8	C8	-2.345189940	0.170007287	1.097776954
9	C9	-3.524217696	0.672669715	0.557226969
10	C10	-3.658862071	0.956953935	-0.813408264
11	C11	-2.567141284	0.732366936	-1.653518052
12	C12	2.221336150	-0.782607816	-2.026048526
13	C13	3.298304425	-1.257217834	-1.299743293
14	C14	3.237066435	-1.455489821	0.089730650
15	C15	2.069039114	-1.206413236	0.790915157
16	O16	-4.562820354	0.828419666	1.397165009
17	C17	-5.614157806	1.769433875	1.057545606
18	C18	-5.907989737	1.736204032	-0.423922579
19	C19	-4.962425560	1.390802048	-1.298678756
20	C20	2.467205764	-0.561856577	-3.455542667
21	C21	3.560519690	-1.080319134	-4.021360039
22	O22	4.519407278	-1.506363137	-1.856729219
23	O23	-2.703425586	0.990911528	-2.954458190
24	C24	-5.143789244	3.164085421	1.483449462
25	C25	-6.815911336	1.313295270	1.876770919
26	C26	4.526444348	-1.938333878	-3.237790620
27	C27	4.120293145	-3.413669944	-3.296421236
28	C28	5.962221589	-1.734690818	-3.702987177
29	H29	-2.267085008	-0.045337397	2.155270966
30	H30	-6.900410517	2.041969497	-0.743293298
31	H31	-5.138227272	1.394919916	-2.368874627
32	H32	1.754310749	0.022165880	-4.019299501
33	H33	3.771828716	-0.925124378	-5.075904117
34	H34	-1.839708610	0.753760368	-3.380158760
35	H35	-5.922731455	3.905985773	1.279393537
36	H36	-4.915060059	3.176984243	2.554229654
37	H37	-4.246692670	3.448492074	0.924488740
38	H38	-7.655571651	1.999074150	1.725343685
39	H39	-6.564602163	1.298970759	2.941510073
40	H40	-7.123412610	0.308319276	1.573881335
41	H41	4.123393967	-3.764693609	-4.333271279
42	H42	3.111436495	-3.549141002	-2.894880532
43	H43	4.819661112	-4.025358997	-2.716817028
44	H44	6.648306996	-2.320040877	-3.083574955
45	H45	6.070413663	-2.061045631	-4.742055730
46	H46	6.238503878	-0.679163120	-3.635078043
47	C47	2.001943870	-1.375863088	2.290894845
48	H48	1.026705037	-1.777942742	2.570862570
49	H49	2.755951642	-2.111373987	2.588237982
50	C50	2.282347653	-0.064897650	2.982570204
51	H51	3.286717616	0.322206728	2.805260836
52	C52	1.452569771	0.665482819	3.733135296
53	C53	0.033153459	0.293101757	4.070057213
54	H54	-0.297030605	-0.629918782	3.591636555
55	H55	-0.649477230	1.093894721	3.758751253
56	H56	-0.087627688	0.178571862	5.154419431
57	C57	1.906020432	1.977019361	4.324186271
58	H58	1.839660483	1.957427482	5.420200928
59	H59	1.262006858	2.798365030	3.983551967
60	H60	2.937442821	2.216045390	4.049385018
61	O61	4.338049413	-1.890736230	0.735270862
62	H62	5.019780370	-2.020902397	0.056129220

5 lowest-energy conformers used for Boltzmann-averaged ^{13}C *NMR data.*
Avg. Energy: -1535.262604 au.

V.17. Tovophyllin A (A23-13).[30]

C	Type	Theory	Expt.	diff.
C1	C	162.2 (-2.9)	160.1	2.1 (-2.9)
C2	C	105.3	110	-4.7
C3	C	164.3	162.3	2
C4	CH	94.1	92.6	1.5
C4a	C	156.5	154.8	1.7
C5	C	115.8	115.3	0.5
C6	C	149.5	149.6	-0.1
C7	C	135.8	137.1	-1.3
C8	C	121.5 (-2.9)	117.1	4.4 (-2.9)
C8a	C	110.1	110	0.1
C9	C	184.9	183.3	1.6
C9a	C	104.5	102.7	1.8
C10a	C	152.2	150.7	1.5
C1'	CH2	22.7	21.2	1.5
C2'	CH	126.8	122.7	4.1
C3'	C	135.9	131.4	4.5
C4'	CH3	25.4	25.7	-0.3
C5'	CH3	16.8	17.8	-1
C1''	CH	125.4 (-3.7)	121	4.4 (-3.7)
C2''	CH	130.2 (+2.8)	131.3	-1.1 (+2.8)
C3''	C	76.7	75.7	1
C4''²	CH3	26.5	27	-0.5
C1'''	CH2	24.2	22.5	1.7
C2'''	CH	123.2	121.6	1.6
C3'''	C	131.8	130.7	1.1
C4'''	CH3	25.5	25.7	-0.2
C5'''	CH3	18.6	17.9	0.7
RMSD		2.19		
Max Abs		4.72		
RMSD+CFx		1.85		
Max abs+CFx		4.72		

mol2 coordinates for lowest energy conformer

1	C1	-4.764900735	-1.032532765	-5.594545812
2	C2	-4.589801442	1.099859623	-4.252585767
3	O3	0.861597061	0.653170182	-2.734964239
4	C4	4.614906352	1.570106981	0.762280367
5	C5	-1.543300057	-0.149559035	-4.017873002
6	C6	4.860427175	2.788398687	1.619607180
7	C7	-2.517354002	-0.135878044	-4.931150629
8	C8	-3.605748776	-1.129907020	-1.061668885
9	C9	-2.781815856	-0.942960203	0.035661385
10	C10	1.215489501	0.310316269	2.025164345
11	C11	2.451885305	0.975450859	-0.405332982
12	O12	-4.060710793	-1.085810780	-3.348621602
13	O13	3.151476332	0.913497806	3.174393080
14	O14	-0.734361539	-0.294159754	0.903792715
15	C15	3.170362555	1.117140270	0.781764704
16	C16	-3.158847723	-0.823023595	-2.357451656
17	C17	2.523585033	0.790227984	1.987217381
18	C18	-1.881315586	-0.355226926	-2.605101656
19	C19	0.357913818	0.354279200	-1.634255297
20	C20	-1.490395592	-0.457436785	-0.215124985
21	C21	0.535744320	0.167756860	0.830487964
22	C22	1.112167098	0.496408045	-0.403462601
23	C23	-1.011703937	-0.152436955	-1.497670777
24	C24	-3.971039627	-0.270919822	-4.540783256
25	O25	3.051183469	1.290181222	-1.557044743

26	O26	-4.855117908	-1.613355590	-0.899685188
27	C27	-2.830038162	-2.714133451	1.776203811
28	C28	-3.240140170	-1.304201967	1.429751610
29	C29	-1.936902114	-3.111641177	2.686859771
30	C30	-1.152600166	-2.197741820	3.591052323
31	C31	-1.632141908	-4.577039590	2.873631775
32	C32	5.786791536	2.961519978	2.574522175
33	C33	6.810135041	1.931530727	2.983318994
34	C34	5.891996515	4.271488274	3.314954882
35	H35	-5.799756162	-1.167974152	-5.266142755
36	H36	-4.771021292	-0.474485530	-6.535698822
37	H37	-4.318932379	-2.015202905	-5.770415677
38	H38	-4.538000725	1.731823981	-5.144692157
39	H39	-4.046657588	1.603493170	-3.447287587
40	H40	-5.639814421	0.991328667	-3.960530804
41	H41	4.878793230	1.805635790	-0.273107685
42	H42	5.262842377	0.738017156	1.062767064
43	H43	-0.505823474	-0.017552597	-4.287474163
44	H44	4.185105956	3.618432885	1.413707177
45	H45	-2.294172031	0.006176379	-5.984892902
46	H46	0.745547915	0.072591700	2.969989372
47	H47	3.925557893	1.492098384	3.066065631
48	H48	2.384732355	1.130708263	-2.275536408
49	H49	-5.256150200	-1.637185473	-1.783971892
50	H50	-3.320343413	-3.477619190	1.171439995
51	H51	-4.331673033	-1.228803183	1.462368796
52	H52	-2.842562619	-0.580816911	2.144001338
53	H53	-1.398293131	-1.143254810	3.457247478
54	H54	-0.078014716	-2.313866988	3.404300773
55	H55	-1.322568418	-2.458677360	4.642628015
56	H56	-0.567168662	-4.777473559	2.696219320
57	H57	-2.215388848	-5.207158291	2.195349713
58	H58	-1.845615697	-4.893164466	3.903002940
59	H59	7.814802037	2.270277475	2.703656357
60	H60	6.812004108	1.804206380	4.072035302
61	H61	6.651507665	0.951801775	2.527771868
62	H62	5.782467368	4.121107857	4.395807725
63	H63	5.132733624	4.987568014	2.989632563
64	H64	6.878437264	4.724599851	3.158418038

8 lowest-energy conformers used for Boltzmann-averaged ^{13}C NMR data.
Avg. Energy: -1536.464136 au.

V.18. Cycloisoemericellin (B10-1).[31]

No	type	δcal	δexp	diff
C1	C	156.7 (-2.9)	154.4	2.3 (-2.9)
C2	C	117.1	116.9	0.2
C3	CH	132.3	131.8	0.5
C4	CH	108.0	108.3	-0.3
C4a	C	157.0	156.4	0.6
C5	CH	118.0	118.8	-0.8
C6	C	140.8	140.5	0.3
C7	C	153.9	152.4	1.5
C8	C	139.4 (-2.9)	134.6	4.8 (-2.9)
C8a	C	123.8	120.7	3.1
C9	C	182.0	179.1	2.9
C9a	C	114.8	112.9	1.9
C10a	C	151.8	152.8	-1.0
C1'	CH	124.0 (-3.7)	120.0*	4.0 (-3.7)
C2'	CH	128.4 (+2.8)	129.4	-1.0 (+2.8)
C3'	C	78.2	77.9	0.3

C4'2	CH3	27.6	28.0	-0.4
C1''	CH2	73.3	72.1	1.2
C2''	CH	122.1	121.6*	0.5
C3''	C	139.3	138.5	0.8
C4''	CH3	26.3	25.8	0.5
C5''	CH3	17.1	18.1	-1.0
C1'''	CH3	18.7	17.5	1.2
C1''''	CH2	58.7	57.2	1.5
RMSD		1.78		
Max Abs		4.76		
RMSD+CFx		1.28		
Max abs+CFx		3.10		

mol2 coordinates for lowest energy conformer

1	C1	0.358433131	-0.393898411	1.009141732
2	C2	0.444776770	-1.044364733	2.242129314
3	O3	0.554868915	-2.397268058	2.355073822
4	C4	0.743670294	-3.160558006	1.252290015
5	C5	0.672962168	-2.626399637	-0.043341661
6	C6	0.294532880	-1.211271370	-0.221616704
7	O7	-0.080440038	-0.755006611	-1.297852487
8	C8	0.986193264	-4.512471047	1.490004740
9	C9	1.192966867	-5.347758191	0.408959449
10	C10	1.160889852	-4.868878675	-0.903611462
11	C11	0.900039638	-3.509742787	-1.123253127
12	C12	0.285075847	1.021029397	0.993819253
13	C13	0.228710547	1.700054662	2.206244323
14	C14	0.291629600	1.030928528	3.444599359
15	C15	0.413107002	-0.346787184	3.447394327
16	C16	1.455754507	-5.695917398	-2.070751744
17	C17	1.239243031	-5.218796488	-3.297091670
18	C18	0.628141776	-3.850132583	-3.487269026
19	O19	0.942225767	-2.998798512	-2.361251626
20	C20	1.229351777	-3.126462560	-4.685465524
21	C21	-0.896601015	-3.941495559	-3.593940172
22	C22	0.302049774	1.822334668	-0.296886071
23	O23	-0.943216591	1.830555479	-0.959510200
24	C24	0.250279500	1.825758178	4.720155162
25	O25	0.150382605	3.067553423	2.228485113
26	C26	-1.172877800	3.573243932	1.961893061
27	C27	-1.075963104	5.052026947	1.786002643
28	C28	-1.380737692	5.746769100	0.684356654
29	C29	-1.260859966	7.249421658	0.674881899
30	C30	-1.882170886	5.154443854	-0.609033017
31	H31	1.019039182	-4.871517741	2.511833507
32	H32	1.400769261	-6.401120076	0.577993597
33	H33	0.480783604	-0.905612046	4.375384115
34	H34	1.868876075	-6.688330988	-1.911542443
35	H35	1.460181484	-5.801466608	-4.187020844
36	H36	0.817081388	-2.116560707	-4.753567311
37	H37	2.315774876	-3.055830855	-4.582283220
38	H38	0.996047303	-3.666734780	-5.608314816
39	H39	-1.322484052	-2.937073162	-3.682007029
40	H40	-1.311025165	-4.424106227	-2.703262856
41	H41	-1.183539451	-4.533198354	-4.469917728
42	H42	0.539158903	2.859318764	-0.055119374
43	H43	1.092198161	1.435888648	-0.951959023
44	H44	-1.030305126	0.928603996	-1.309371515
45	H45	-0.718034220	2.324346676	4.839162142
46	H46	1.012227075	2.610640776	4.710505356

47	H47	0.412893605	1.183710163	5.590031078
48	H48	-1.587445032	3.071178206	1.082960230
49	H49	-1.815018111	3.327411906	2.821899032
50	H50	-0.716297874	5.592005268	2.661402529
51	H51	-0.565551919	7.576475694	-0.108844163
52	H52	-2.230202695	7.713212911	0.450135553
53	H53	-0.907807061	7.641305088	1.633406970
54	H54	-1.751588235	4.072718299	-0.690982289
55	H55	-1.359898841	5.616278003	-1.455344147
56	H56	-2.948251554	5.380345128	-0.739733725

2 lowest-energy conformers used for Boltzmann-averaged ^{13}C NMR data.
Avg. Energy: -1344.471072 au.

V.19. 10-Methoxy-2,2,6,6-tetramethyl-2H,6H,14H-dipyrano[2,3-a:2′,3′-c]xanthen-14-one (B13-1–D31-18).[32]

No	type	δcal	δexp	diff
C1	C	158.4 (-2.9)	155.3	3.1 (-2.9)
C2	C	107.5	106.7	0.8
C3	C	154.4	153.4	1.0
C4	C	103.6	102.2	1.4
C4a	C	154.5	152.4	2.1
C5	C	147.4	148.2	-0.8
C6	CH	113.0	114.6	-1.6
C7	CH	122.4	123.1	-0.7
C8	CH	120.2 (-2.9)	117.6	2.6 (-2.9)
C8a	C	125.8	123.7	2.1
C9	C	175.1	175.2	-0.1
C9a	C	109.8	106.4	3.4
C10a	C	145.4	145.2	0.2
C1'	CH	119.9 (-3.7)	115.7	4.2 (-3.7)
C2'	CH	125.4 (+2.8)	127.7	-2.3 (+2.8)
C3'	C	78.0	77.8	0.2
C4'[2]	CH3	27.5	28	-0.5
C1''	CH	120.9 (-3.7)	115.8	5.1 (-3.7)
C2''	CH	123.7 (+2.8)	126.9	-3.2 (+2.8)
C3''	C	78.1	78.1	0.0
C4''[2]	CH3	27.7	28.2	-0.5
C1'''	CH3	54.5	56.4	-1.9
RMSD		2.12		
Max Abs		5.08		
RMSD+CFx		1.20		
Max abs+CFx		3.36		

mol2 coordinates for lowest energy conformer

1	C1	0.622486019	-0.321355352	-0.917866786
2	C2	0.645167252	0.052116049	0.436549898
3	C3	-0.508565649	0.342807290	1.169963001
4	C4	-1.736919722	0.241581003	0.513287487
5	C5	-1.829381091	-0.119208135	-0.835705097
6	C6	-0.649545102	-0.393520584	-1.537616281
7	C7	1.888082111	-0.566236296	-1.633889326
8	C8	3.105328781	-0.404118844	-0.795081060
9	C9	2.992748595	-0.044604993	0.539089717
10	O10	1.796825545	0.177031549	1.139608802
11	C11	4.377618010	-0.613027201	-1.346743152
12	C12	5.498610777	-0.461613703	-0.559231254
13	C13	5.380852186	-0.099125349	0.791575923
14	C14	4.131447383	0.113521517	1.355347168
15	O15	1.980601102	-0.868019352	-2.812145744
16	O16	-0.707595160	-0.800592445	-2.810498152
17	C17	-0.499929792	0.819938726	2.547800970

18	C18	-1.650905708	0.898363093	3.217657910
19	C19	-2.937796717	0.407346818	2.596352706
20	O20	-2.886150450	0.537004663	1.151544573
21	C21	-3.168419487	-1.070523437	2.926016526
22	C22	-4.126861472	1.264221587	3.011844888
23	O23	3.887431843	0.464622499	2.639856591
24	C24	4.998551264	0.627182012	3.492637467
25	C25	-3.101350632	-0.321825842	-1.520019907
26	C26	-3.115359782	-0.495282864	-2.842055766
27	C27	-1.831146468	-0.397591575	-3.630542469
28	C28	-1.808548476	-1.371071950	-4.802160688
29	C29	-1.579134705	1.040283770	-4.093702558
30	H30	4.436706532	-0.892658579	-2.392545674
31	H31	6.487356553	-0.621808974	-0.977123121
32	H32	6.277073952	0.012532742	1.391020759
33	H33	0.444366389	1.125997847	2.984669404
34	H34	-1.697378638	1.276709363	4.234601305
35	H35	-2.331851987	-1.674874449	2.562271094
36	H36	-4.093427816	-1.425249221	2.459053960
37	H37	-3.244280041	-1.208491144	4.009332142
38	H38	-5.035844492	0.918491275	2.510841375
39	H39	-4.278381772	1.196970434	4.093857992
40	H40	-3.953311320	2.310499483	2.745021101
41	H41	5.663405411	1.425637844	3.139855581
42	H42	4.590377741	0.903251663	4.465145987
43	H43	5.570801967	-0.305192799	3.588404969
44	H44	-4.005654749	-0.352675439	-0.922421763
45	H45	-4.035240821	-0.681572383	-3.388707226
46	H46	-0.838590314	-1.325631681	-5.305089467
47	H47	-2.594054798	-1.113139231	-5.519814408
48	H48	-1.973274614	-2.393262628	-4.450071172
49	H49	-0.625766885	1.099288852	-4.627928330
50	H50	-2.384091444	1.370202327	-4.758459215
51	H51	-1.547079312	1.716672047	-3.233790678

3 lowest-energy conformers used for Boltzmann-averaged ^{13}C NMR data.
Avg. Energy: -1303.957518 au.

V.20. Garciosone E (B13-2).[33]

No	type	δcal	δexp	diff
C1	C	158.2 (-2.9)	155.3	2.9 (-2.9)
C2	C	107.7	107.2	0.5
C3	C	158.2	159.4	-1.2
C4	CH	91.4	91.4	0.0
C4a	C	159.2	158	1.2
C5	C	134.1	133.5	0.6
C6	C	151.3	152.2	-0.9
C7	CH	104.8	105.5	-0.7
C8	CH	124.3 (-2.9)	121.3	3.0 (-2.9)
C8a	C	120.8	119.2	1.6
C9	C	174.1	174	0.1
C9a	C	109.7	106.8	2.9
C10a	C	140.6	140	0.6
C1'	CH	119.8 (-3.7)	115.8	4.0 (-3.7)
C2'	CH	125.4 (+2.8)	127.6	-2.2 (+2.8)
C3'	C	77.6	77.6	0.0
C4'[2]	CH3	27.3	27.8	-0.5
C1''	CH3	54.6	55.9	-1.3
C1'''	CH2	105.3	102.9	2.4
RMSD		1.76		
Max Abs		3.96		

RMSD+CFx	1.11
Max abs+CFx	2.92

mol2 coordinates for lowest energy conformer

1	O1	-1.622221101	-0.291855160	-2.084176966
2	C2	2.316651297	-0.156904060	1.187379880
3	C3	-4.779552221	0.296774794	1.697398190
4	C4	2.308720377	-0.344564724	-0.207177180
5	C5	-5.029702999	0.161049368	0.341371599
6	C6	1.131743573	-0.001778999	1.893326340
7	C7	-3.504807561	0.280903413	2.229934515
8	C8	1.083911449	-0.363963184	-0.878854078
9	C9	-3.917376524	0.004584086	-0.479505288
10	O10	-1.170312871	0.125078973	1.979300993
11	C11	-1.460329174	-0.187160629	-0.879395219
12	C12	-0.071553668	-0.037108321	1.193123926
13	C13	-2.401270606	0.126716638	1.412440453
14	C14	-0.149919133	-0.210795110	-0.196381873
15	C15	-2.616717354	-0.015184083	0.033546308
16	C16	3.518257562	-0.616312577	-0.977094177
17	C17	3.466238330	-0.604897555	-2.309571681
18	C18	2.183665804	-0.231781302	-3.012777929
19	O19	1.044741767	-0.592231444	-2.197393206
20	C20	2.119006248	1.276474252	-3.270942623
21	C21	1.989891486	-1.024772807	-4.299175285
22	O22	-5.683716300	0.463895022	2.702441284
23	C23	-4.932238775	0.538631605	3.911410429
24	O24	-3.549543480	0.436894349	3.585187458
25	O25	3.537418393	-0.151594631	1.763423097
26	C26	3.616708236	-0.004087282	3.165884784
27	H27	-6.040028526	0.176905697	-0.049122506
28	H28	1.096067872	0.144383151	2.964476207
29	H29	-4.025625522	-0.108857434	-1.552533033
30	H30	4.430244531	-0.851034682	-0.439736111
31	H31	4.335487806	-0.830782695	-2.920617840
32	H32	1.169354668	1.533844219	-3.750148384
33	H33	2.201792796	1.827339883	-2.328731656
34	H34	2.944568535	1.584622335	-3.920954481
35	H35	2.028530232	-2.098103417	-4.093376432
36	H36	2.776179561	-0.774025618	-5.018433240
37	H37	1.017231606	-0.787625016	-4.738463732
38	H38	-5.214990911	-0.293161251	4.565615490
39	H39	-5.121992090	1.501541897	4.396917380
40	H40	4.678449295	-0.042858166	3.410282182
41	H41	3.092950490	-0.817478765	3.682439555
42	H42	3.204086900	0.959279229	3.488662850

1 lowest-energy conformers used for Boltzmann-averaged ^{13}C NMR data.
Avg. Energy: -1222.685511 au.

V.21. Osajaxanthone (B31-2)[34]

No	type	δcal	δexp	diff
C1	C	160.4 (-2.9)	157	3.4 (-2.9)
C2	C	102.1	104	-1.9
C3	C	161.0	160.5	0.5
C4	CH	93.6	94.9	-1.3
C4a	C	157.9	157	0.9
C5	CH	119.4	119.4	0.0
C6	CH	121.9	125.2	-3.3
C7	C	151.1	154.4	-3.3
C8	CH	110.4 (-2.9)	108.2	2.2 (-2.9)

C8a	C	123.1	120.6	2.5
C9	C	182.0	180.5	1.5
C9a	C	104.6	103.1	1.5
C10a	C	149.4	149.4	0.0
C1'	CH	118.4 (-3.7)	114.8	3.6 (-3.7)
C2'	CH	122.2 (+2.8)	128.8	-6.6 (+2.8)
C3'	C	80.1	78.8	1.3
C4'[2]	CH3	30.9	28.3	2.6
RMSD		2.66		
Max Abs		6.59		
RMSD+CFx		1.97		
Max abs+CFx		3.79		

mol2 coordinates for lowest energy conformer

1	C1	0.841620871	-1.262950717	-4.786979855
2	C2	0.842565409	1.262638894	-4.787364701
3	O3	-2.540678792	0.000859628	2.450902907
4	O4	-0.246954287	-0.000720124	7.049862795
5	O5	-2.861618270	0.001223600	-0.133695338
6	C6	-1.735744492	0.000994879	-2.726694206
7	C7	-1.123707476	0.000748232	-3.909578154
8	C8	-0.803117035	-0.000123248	4.700027002
9	C9	1.272092125	-0.000187445	-0.475276124
10	O10	1.073307072	0.000099729	-2.785726465
11	O11	1.515143055	-0.000507816	1.840380462
12	C12	0.110377964	-0.000626913	5.738369453
13	C13	-0.953752577	0.000657925	-1.498528325
14	C14	0.448184465	0.000183823	-1.599803332
15	C15	-1.530948986	0.000766912	-0.230046226
16	C16	1.935683470	-0.001026818	4.151300226
17	C17	1.486962668	-0.001079173	5.456392748
18	C18	-1.314071989	0.000441427	2.256646489
19	C19	0.677079545	-0.000096557	0.774404028
20	C20	-0.357529808	-0.000066244	3.372068391
21	C21	1.009817929	-0.000520767	3.105424888
22	C22	-0.721199349	0.000387013	0.938371176
23	C23	0.375264431	0.000133058	-4.060574846
24	H24	0.405057733	-1.308726598	-5.790076668
25	H25	1.932232180	-1.264694227	-4.879055270
26	H26	0.532332738	-2.152799784	-4.231404914
27	H27	0.406076065	1.308430409	-5.790494754
28	H28	1.933178652	1.263529377	-4.879410889
29	H29	0.533936763	2.152888523	-4.232079597
30	H30	-1.209469861	-0.000384989	7.116398075
31	H31	-3.082683681	0.001201987	0.831296857
32	H32	-2.816959579	0.001517130	-2.646271252
33	H33	-1.688022563	0.000982590	-4.837986661
34	H34	-1.875691573	0.000236795	4.874665671
35	H35	2.349284099	-0.000562839	-0.579766558
36	H36	2.994863979	-0.001373985	3.919441441
37	H37	2.187089103	-0.001473686	6.284861524

2 lowest-energy conformers used for Boltzmann-averaged ^{13}C NMR data.
Avg. Energy: -1070.525696 au.

V.22. 6-Deoxyjacareubin (B31-3).[26]

C	Type	Theory	Expt.	diff.
C1	C	160.9 (-2.9)	157.7	3.2 (-2.9)
C2	C	102.6	104.5	-1.9
C3	C	160.9	160.8	0.1
C4	CH	93	94.9	-1.9

C4a	C	156.7	156.9	-0.2
C5	C	144.9	145.2	-0.3
C6	CH	119.1	120.7	-1.6
C7	CH	124.2	124.2	0
C8	CH	118.2 (-2.9)	115.4	2.8 (-2.9)
C8a	C	122.7	121.3	1.4
C9	C	182.6	181	1.6
C9a	C	104.5	103.3	1.2
C10a	C	143.4	146.1	-2.7
C1'	CH	118.3 (-3.7)	114.8	3.5 (-3.7)
C2'	CH	122.4 (+2.8)	128.1	-5.7 (+2.8)
C3'	C	80.4	78.4	2
C4'[2]	CH3	30.9	27.7	3.2
RMSD		2.47		
Max Abs		5.67		
RMSD+CFx		1.76		
Max abs+CFx		3.20		

mol2 coordinates for lowest energy conformer

1	C1	1.068924162	1.262876607	-4.595452390
2	C2	1.067508708	-1.263408338	-4.594333954
3	O3	-2.871783489	0.000555959	2.349787268
4	C4	-1.662108826	0.001464816	-2.740867856
5	C5	-0.960420085	0.001236870	-3.872697010
6	C6	0.930057022	-0.001143273	5.664165039
7	C7	-0.976814857	0.000882914	-1.456146607
8	C8	1.473482838	-0.001057506	4.389649436
9	C9	-1.651702423	0.000790981	-0.235482112
10	O10	1.143055310	0.000689553	-2.581985596
11	O11	1.218496890	-0.000520839	2.069192882
12	C12	0.426941508	0.000487530	-1.449585768
13	C13	-0.458271200	-0.000768823	5.841468397
14	C14	1.161758027	0.000016982	-0.262831067
15	C15	-1.309681946	-0.000312042	4.751690971
16	C16	-1.634851422	0.000244790	2.262674725
17	C17	0.604641036	-0.000584837	3.289016532
18	C18	-0.936396658	0.000320018	0.992410875
19	C19	0.468902488	-0.000060299	0.933008930
20	C20	-0.776465832	-0.000211722	3.456574773
21	C21	0.545690538	0.000341782	-3.908404564
22	O22	2.815498316	-0.001420091	4.216828502
23	O23	-2.984680115	0.001180433	-0.246214118
24	H24	2.163339896	1.263958297	-4.600589228
25	H25	0.713712242	1.308077949	-5.630173495
26	H26	0.716511364	2.153172680	-4.067125251
27	H27	2.161923148	-1.265717377	-4.599553232
28	H28	0.712144467	-1.309158437	-5.628976060
29	H29	0.714139039	-2.152843031	-4.065162885
30	H30	-2.746268489	0.002183242	-2.742883781
31	H31	-1.451629995	0.001650983	-4.841692097
32	H32	1.605814756	-0.001503651	6.512718506
33	H33	-0.863186764	-0.000848051	6.848000090
34	H34	-2.387768741	-0.000015834	4.863953779
35	H35	2.997744201	-0.001319609	3.266547508
36	H36	-3.282131044	0.001035748	0.698521643
37	H37	2.243875931	-0.000274371	-0.286052782

1 lowest-energy conformers used for Boltzmann-averaged ^{13}*C NMR data.*
Avg. Energy: -1070.140445 au.

V.23. Jacareubin (B31-4).[36]

No	type	δcal	δexp	diff
C1	C	161.0 (-2.9)	156.7*	4.3 (-2.9)
C2	C	102.7	103.8	-1.1
C3	C	160.4	159.6*	0.8
C4	CH	92.9	94.7	-1.8
C4a	C	156.8	156.5	0.3
C5	C	130.0	132.6	-2.6
C6	C	149.5	152.1	-2.6
C7	CH	112.1	113.2	-1.1
C8	CH	121.4 (-2.9)	116	5.4 (-2.9)
C8a	C	115.1	113	2.1
C9	C	181.9	180	1.9
C9a	C	104.0	102.3	1.7
C10a	C	144.0	146.1	-2.1
C1'	CH	118.4 (-3.7)	114.5	3.9 (-3.7)
C2'	CH	122.3 (+2.8)	128.2	-5.9 (+2.8)
C3'	C	80.3	78.4	1.9
C4'[2]	CH3	30.9	27.9	3.0
RMSD		2.93		
Max Abs		5.94		
RMSD+CFx		2.05		
Max abs+CFx		3.14		

mol2 coordinates for lowest energy conformer

1	C1	1.704763937	0.001851720	0.499557120
2	C2	0.967950840	0.001748194	1.685159820
3	C3	-0.432995503	0.000205417	1.607554311
4	C4	-1.105584650	-0.000959041	0.384527463
5	C5	-1.172271062	-0.000864466	-4.265896719
6	C6	-0.572526508	0.000372180	-5.523772800
7	C7	0.822855083	0.002048803	-5.637682611
8	C8	1.607229009	0.002272001	-4.500389457
9	C9	1.818051043	0.000948155	-1.998678409
10	C10	-0.370314508	-0.000299719	-3.130683980
11	C11	-0.351830639	-0.000450953	-0.773500940
12	C12	1.022711552	0.001111580	-3.227312088
13	C13	1.053695680	0.000780392	-0.762465265
14	O14	3.035429605	0.003142479	0.578120324
15	O15	-1.206791510	0.000143424	2.702061323
16	O16	3.058830785	0.001372844	-2.017440001
17	O17	-1.046995412	-0.001410403	-1.946934630
18	H18	2.689389966	0.003364524	-4.564627947
19	H19	1.259334320	0.003099783	-6.629911821
20	H20	3.379385013	0.001347479	-0.351545296
21	O21	-2.535291570	-0.002476057	-4.214876488
22	H22	-2.808035043	-0.003249621	-3.287402144
23	O23	-1.336759917	-0.000015142	-6.635648888
24	H24	-2.266107984	-0.001246537	-6.362923766
25	C25	1.586573428	0.003699044	3.003490663
26	H26	2.669075950	0.005980027	3.061870426
27	C27	0.828148096	0.002817178	4.098262661
28	H28	1.269281748	0.004307754	5.091049260
29	C29	-0.677782068	-0.000676141	4.057038007
30	H30	-2.187444286	-0.002035415	0.351863867
31	C31	-1.237875982	1.260140183	4.717842499
32	H32	-2.331104288	1.260081497	4.665678078
33	H33	-0.859178435	2.151683662	4.210254425
34	H34	-0.937357199	1.303900438	5.769814643
35	C35	-1.231763568	-1.266032297	4.714457034

36	H36	-2.324964659	-1.271157409	4.662320364
37	H37	-0.930976380	-1.311133055	5.766280946
38	H38	-0.848754885	-2.154412505	4.204490016

1 lowest-energy conformers used for Boltzmann-averaged ^{13}C NMR data.
Avg. Energy: -1145.756555 au.

V.24. Nigrolineaxanthone K (B31-6).[37]

C	Type	Theory	Expt.	diff.
C1	C	161.2 (-2.9)	158.2	3.0 (-2.9)
C2	C	104.8	104.9	-0.1
C3	C	161.2	160.4	0.8
C4	CH	93.3	94.2	-0.9
C4a	C	156.0	155.5	0.5
C5	C	143.5	142.5	1.0
C6	CH	119.1	119.4	-0.3
C7	CH	126.5	125.2	1.3
C8	C	136.0 (-2.9)	135.5	0.5 (-2.9)
C8a	C	120.6	118.4	2.2
C9	C	184.9	182.9	2.0
C9a	C	105.2	104.2	1.0
C10a	C	145.0	145.1	-0.1
C1'	CH	120.0 (-3.7)	115.5	4.5 (-3.7)
C2'	CH	123.5 (+2.8)	127.5	-4.0 (+2.8)
C3'	C	79.0	78.3	0.7
C4'[2]	CH3	28.7	28.4	0.3
C1''	CH2	35.5	33.0	2.5
C2''	CH	125.4	122.9	2.5
C3''	C	132.3	132.7	-0.4
C4''	CH3	18.7	18.0	0.7
C5''	CH3	25.5	25.9	-0.4
RMSD		1.78		
Max Abs		4.53		
RMSD+CFx		1.24		
Max abs+CFx		2.48		

mol2 coordinates for lowest energy conformer

1	C1	-2.345144672	1.359735365	-4.045507544
2	C2	-0.117398207	1.226634001	-5.236457206
3	C3	0.560010897	-4.966529200	4.340435744
4	C4	1.691003820	-4.728471384	2.095301885
5	O5	-1.655319813	1.038974336	-1.050753219
6	O6	2.676930673	3.717217045	2.106917879
7	O7	-1.966489084	-1.385596617	-0.259437452
8	C8	-1.299424600	-3.456322255	1.545909751
9	C9	-0.785817823	-4.437688387	2.288728392
10	C10	-0.395260888	2.809081812	-3.359020886
11	C11	1.571793090	4.761908686	0.280151603
12	C12	0.639258012	4.691312006	-0.755541668
13	C13	1.035666697	-0.682707425	2.517613958
14	C14	-1.039669861	3.551313799	-2.212557481
15	O15	0.770645876	-2.861591856	3.308315997
16	O16	1.281118780	1.505422799	1.746004557
17	C17	-0.933169190	1.871055365	-4.144158443
18	C18	1.768520685	3.671271271	1.104706144
19	C19	-0.127687598	3.559508687	-1.000172796
20	C20	-0.991616487	-1.145205523	0.616210610
21	C21	0.401503191	-1.919990398	2.423803744
22	C22	-0.618977748	-2.168544354	1.489953256
23	C23	-0.723192831	1.188893276	-0.246308920
24	C24	0.647263988	0.310339811	1.637044323

25	C25	1.001960148	2.520985591	0.875828742
26	C26	0.051888952	2.440446526	-0.147505681
27	C27	-0.347620075	0.121647814	0.666079154
28	C28	0.539760559	-4.260142192	2.990551874
29	H29	-2.855698301	1.474046268	-5.010895109
30	H30	-2.937092303	1.865767777	-3.282514120
31	H31	-2.343951011	0.288962947	-3.808518686
32	H32	-0.056435375	0.141313164	-5.083617390
33	H33	-0.583171829	1.379677986	-6.218584060
34	H34	0.901516115	1.622284467	-5.276114442
35	H35	0.438907433	-6.045440122	4.201287862
36	H36	1.511675871	-4.785450636	4.848508685
37	H37	-0.252739172	-4.600741921	4.973986983
38	H38	1.575761752	-5.790626401	1.856056545
39	H39	2.650744073	-4.581903092	2.601753324
40	H40	1.694284649	-4.165863431	1.156353790
41	H41	2.663344483	2.863607748	2.562573732
42	H42	-2.106130935	-0.543723628	-0.765052542
43	H43	-2.229684400	-3.571040072	1.000463015
44	H44	-1.275082847	-5.402968887	2.381119726
45	H45	0.641540030	3.093495151	-3.544948196
46	H46	2.157376368	5.658958828	0.450495758
47	H47	0.513627487	5.555849477	-1.400875704
48	H48	1.812250828	-0.513444380	3.252421805
49	H49	-1.200722970	4.596803881	-2.508598805
50	H50	-2.010856440	3.127476276	-1.961438489

3 lowest-energy conformers used for Boltzmann-averaged ^{13}C NMR data.
Avg. Energy: -1265.874789 au.

V.25. Garcimangosxanthone E (B31-7).[38]

C	Type	Theory	Expt.	diff.
C1	C	160.7 (-2.9)	157.8	2.9 (-2.9)
C2	C	104.6	104.5	0.1
C3	C	161	160.1	0.9
C4	CH	94.1	94.2	-0.1
C4a	C	157	156.3	0.7
C5	CH	101.1	101.7	-0.6
C6	C	155.7	154.7	1.0
C7	C	140.7	138.5*	2.2
C8	C	143.4 (-2.9)	142.6*	0.8 (-2.9)
C8a	C	113.1	112	1.1
C9	C	184.2	182	2.2
C9a	C	104.8	103.7	1.1
C10a	C	156.7	156	0.7
C1'	CH	120.1 (-3.7)	115.6	4.5 (-3.7)
C2'	CH	123.3 (+2.8)	127.3	-4.0 (+2.8)
C3'	C	78.7	78	0.7
C4'[2]	CH3	28.8	28.3	0.5
C1''	CH2	23.6	22.2	1.4
C2''	CH2	43.1	44.4	-1.3
C3''	C	69.6	70.7	-1.1
C4''[2]	CH3	29.7	29.3	0.4
C1'''	CH3	61.5	62.4	-0.9
RMSD		1.67		
Max Abs		4.51		
RMSD+CFx		1.09		
Max abs+CFx		2.21		

mol2 coordinates for lowest energy conformer

1	C1	-1.329601054	-0.256420808	0.678580352

2	C2	-1.158946912	-0.144718566	2.070592712
3	O3	0.058730736	-0.161255472	2.673861487
4	C4	1.193152505	-0.304809732	1.948976153
5	C5	1.138592620	-0.470581954	0.557518610
6	C6	-0.132078467	-0.458135510	-0.146119132
7	O7	-0.170914855	-0.612192367	-1.382425739
8	C8	2.382450284	-0.293716487	2.657478050
9	C9	3.566581374	-0.451700685	1.943623040
10	C10	3.582381306	-0.611850202	0.546549807
11	C11	2.367551718	-0.624700373	-0.140473913
12	C12	-2.654289110	-0.207502963	0.151908088
13	C13	-3.706778626	-0.062594130	1.039207074
14	C14	-3.501915876	0.013889875	2.433753183
15	C15	-2.224332905	-0.014761433	2.954527097
16	O16	4.713725477	-0.390877272	2.643028169
17	C17	5.912577936	-1.001524031	2.099320071
18	C18	5.985705040	-0.809920302	0.603267051
19	C19	4.875533669	-0.670617328	-0.122514299
20	C20	-2.964629384	-0.266931964	-1.322944969
21	C21	-2.651020255	1.065730170	-2.022005618
22	O22	-5.034246311	-0.046705148	0.662192195
23	O23	2.390732220	-0.773188683	-1.465559119
24	O24	-4.567148856	0.114453502	3.245128032
25	C25	5.876107443	-2.492089847	2.450201584
26	C26	7.058120716	-0.289695486	2.809916295
27	C27	-3.276947850	1.206340123	-3.411978585
28	C28	-2.854933237	2.541023639	-4.032146857
29	C29	-2.892963380	0.042659355	-4.329416459
30	O30	-4.692157421	1.209480749	-3.194711294
31	H31	2.391908184	-0.171074725	3.732775622
32	H32	-2.049065714	0.050811446	4.021042704
33	H33	6.970525832	-0.832458335	0.146004441
34	H34	4.901441847	-0.570402596	-1.201993981
35	H35	-4.024177521	-0.504069299	-1.444145885
36	H36	-2.380329323	-1.062433059	-1.785689054
37	H37	-1.566578001	1.175274239	-2.107854348
38	H38	-3.014046121	1.901323949	-1.410543511
39	H39	1.444872998	-0.753795737	-1.764277029
40	H40	6.781991000	-2.988694185	2.087286834
41	H41	5.809566115	-2.625746090	3.534904917
42	H42	5.012338945	-2.970885264	1.978923884
43	H43	8.017082307	-0.715592586	2.497402206
44	H44	6.961215343	-0.404948915	3.893494748
45	H45	7.051085046	0.776884238	2.567314975
46	H46	-1.770941262	2.580169876	-4.183620123
47	H47	-3.148682601	3.371017134	-3.382234660
48	H48	-3.334319992	2.683339379	-5.009005367
49	H49	-3.288333013	0.202266407	-5.340738020
50	H50	-1.805035883	-0.053863282	-4.402674499
51	H51	-3.298852944	-0.899497317	-3.951031454
52	H52	-5.123124487	1.251184765	-4.057030695
53	C53	-5.513829777	1.208669388	0.168701243
54	H54	-5.213907897	1.365323800	-0.871074274
55	H55	-5.151781834	2.032864134	0.794965805
56	H56	-6.603268746	1.159261172	0.221625747
57	H57	-5.344791047	0.017984792	2.668136710

12 lowest-energy conformers used for Boltzmann-averaged ^{13}C NMR data.
Avg. Energy: -1456.866705 au.

V.26. Trapezifolixanthone (B31-8).[39] **Also call Toxyloxanthone A.**[67]

C	Type	Theory	Expt.	diff.
C1	C	159.4 (-2.9)	156.1	3.3 (-2.9)
C2	C	106.1	104.9	1.2
C3	C	159.6	158.4	1.2
C4	C	106.1	107.1	-1
C4a	C	154.3	153.7	0.6
C5	C	145.2	144.5	0.7
C6	CH	119.1	119.8	-0.7
C7	CH	124	124.1	-0.1
C8	CH	118.2 (-2.9)	116.9	1.3 (-2.9)
C8a	C	122.5	120.9	1.6
C9	C	183.3	181.1	2.2
C9a	C	105.2	103.4	1.8
C10a	C	143.6	144.3	-0.7
C1'	CH	121.0 (-3.7)	115.8	5.2 (-3.7)
C2'	CH	124.5 (+2.8)	127.6	-3.1 (+2.8)
C3'	C	78.3	78.4	-0.1
C4'2	CH3	27.7	28.4	-0.7
C1''	CH2	23.2	21.8	1.4
C2''	CH	125.5	122.8	2.7
C3''	C	129.9	131.7	-1.8
C4''	CH3	18.3	18	0.3
C5''	CH3	25.4	25.7	-0.3
RMSD		1.86		
Max Abs		5.23		
RMSD+CFx		1.24		
Max abs+CFx		2.71		

mol2 coordinates for lowest energy conformer

1	O1	0.931786088	2.426943296	-3.705153950
2	C2	-0.548118790	-1.538424002	-1.000663394
3	C3	0.085455004	5.438875783	0.250623864
4	C4	-0.081860801	-1.456594203	-2.323578038
5	C5	0.565650617	5.708138519	-1.036277036
6	C6	-0.715196053	-0.419802415	-0.176137578
7	C7	-0.253106594	4.146036186	0.615052003
8	C8	0.261526473	-0.206438039	-2.836362555
9	C9	0.712047462	4.692580310	-1.963151615
10	O10	-0.468927249	1.881809552	0.103043863
11	C11	0.514923688	2.258949895	-2.549839530
12	C12	-0.343960714	0.803701607	-0.718934554
13	C13	-0.107586609	3.121403698	-0.331447268
14	C14	0.142783095	0.955214588	-2.031200780
15	C15	0.372974142	3.379825198	-1.610398687
16	O16	-0.909792538	-2.728619590	-0.483815541
17	C17	-0.314099656	-3.940563031	-1.012949171
18	C18	-0.196368999	-3.858864907	-2.516183512
19	C19	-0.046558513	-2.678304342	-3.118165898
20	O20	0.699860841	-0.130241438	-4.093637682
21	O21	-0.718483330	3.881146320	1.857201657
22	C22	-1.186939697	-0.551284132	1.253514229
23	C23	-0.017351005	-0.600263365	2.204853969
24	C24	0.191664804	0.129765080	3.304489451
25	C25	1.446062203	-0.046156913	4.121827227
26	C26	-0.758838456	1.163222232	3.851186341
27	C27	1.065851180	-4.101367843	-0.367839362
28	C28	-1.263460062	-5.050822909	-0.581418142
29	H29	-0.030357807	6.228707898	0.985281495
30	H30	0.825511139	6.727713691	-1.301275621

31	H31	1.085196597	4.875399241	-2.964346323
32	H32	-0.200126123	-4.791411994	-3.072782380
33	H33	0.080154857	-2.588895343	-4.191434198
34	H34	0.889686471	0.824268987	-4.276063105
35	H35	-0.867684925	2.927061051	1.928353493
36	H36	-1.764729968	-1.476736560	1.338774567
37	H37	-1.866835491	0.270844639	1.491316355
38	H38	0.745284194	-1.327712265	1.924845760
39	H39	2.104273124	-0.810748236	3.699492002
40	H40	2.009163860	0.894615836	4.178448765
41	H41	1.205881604	-0.332404834	5.153948443
42	H42	-0.263696874	2.139169929	3.942980601
43	H43	-1.665137081	1.283233951	3.250895776
44	H44	-1.081082329	0.887355955	4.863168421
45	H45	1.542210023	-5.022583280	-0.717740377
46	H46	0.972536528	-4.143817447	0.722660453
47	H47	1.711214070	-3.259947834	-0.638529381
48	H48	-1.368377179	-5.057321156	0.507459874
49	H49	-0.873270619	-6.022556293	-0.899753069
50	H50	-2.249750601	-4.904101073	-1.030339860

2 lowest-energy conformers used for Boltzmann-averaged ^{13}C NMR data.
Avg. Energy: -1265.881421 au.

V.27. Inoxanthone (B31-9).[1] **Also call blancoxanthone**[2] **and caloxanthone C**[3]

No	type	δcal	δexp	diff
C1	C	159.7 (-2.9)	156.7	3.0 (-2.9)
C2	C	106.7	105.5	1.2
C3	C	159.7	159.4	0.3
C4	C	111.6	113.1	-1.5
C4a	C	155.9	153.9	2.0
C5	C	146.4	145.3	1.1
C6	CH	119.7	120.5	-0.8
C7	CH	124.3	124.2	0.1
C8	CH	117.3 (-2.9)	116	1.3 (-2.9)
C8a	C	122.2	119.6	2.6
C9	C	183.7	181.3	2.4
C9a	C	105.7	103.6	2.1
C10a	C	143.8	144.1	-0.3
C1'	CH	121.2 (-3.7)	116.0	5.2 (-3.7)
C2'	CH	123.9 (+2.8)	127.3	-3.4 (+2.8)
C3'	C	77.9	78.4	-0.5
C4'[2]	CH3	27.6	27.9	-0.3
C1''	C	42.1	41.3	0.8
C2''	CH	157.1	155.8	1.3
C3''	CH2	106.0	104	2.0
C4''[2]	CH3	29.7	28.2	1.5
RMSD		1.95		
Max Abs		5.18		
RMSD+CFx		1.36		
Max abs+CFx		2.58		

mol2 coordinates for lowest energy conformer

1	C1	-2.162626544	-2.968978287	-0.997621736
2	C2	3.403940712	0.312510113	-2.585082780
3	C3	2.277456864	2.090983880	-3.979178005
4	C4	-2.248709220	-0.061821828	-1.630392681
5	C5	-0.250263530	-1.019699944	-2.793827139
6	O6	0.662316640	1.293079925	4.199427372
7	O7	-3.375158660	-2.442992404	1.859180065
8	O8	2.159355077	2.302150772	2.349314652

9	C9	-1.086884200	-2.193603298	-0.860955320
10	C10	2.713022355	2.648166758	-0.377635286
11	C11	3.076447903	2.614704259	-1.659125832
12	C12	-2.562121567	-1.277181242	5.226144230
13	C13	-1.505222774	-0.420201478	4.980879466
14	O14	1.193028675	1.163183771	-2.115369454
15	O15	-1.365181070	-0.756828567	1.321132686
16	C16	-3.191910578	-1.959267031	4.177723332
17	C17	-2.770646602	-1.784276197	2.868372721
18	C18	1.441733621	1.588490231	1.482545765
19	C19	-0.050085893	0.056890905	-0.434070545
20	C20	0.969866272	0.955161816	-0.802904530
21	C21	1.723031506	1.702283404	0.124697961
22	C22	0.050274412	0.658812506	3.327106625
23	C23	-1.068920779	-0.227162156	3.663585720
24	C24	-1.708259668	-0.899082337	2.629166490
25	C25	-0.326233415	0.028400047	0.940066831
26	C26	0.391089555	0.744827548	1.915417371
27	C27	2.510531044	1.556408689	-2.571525660
28	C28	-0.906344779	-0.782004918	-1.409303055
29	H29	-2.153762859	-4.000248990	-0.657108889
30	H30	-3.079432942	-2.620603218	-1.466971527
31	H31	3.490049023	-0.105109401	-1.577043896
32	H32	4.408984215	0.574387660	-2.931124666
33	H33	2.989646412	-0.449271821	-3.253666401
34	H34	1.823518390	1.319411266	-4.608419416
35	H35	1.613679618	2.959582999	-3.951015739
36	H36	3.229525300	2.390388982	-4.428470243
37	H37	-2.824352951	0.005786459	-0.702369841
38	H38	-2.856962760	-0.580358200	-2.379311114
39	H39	-2.062770095	0.951806463	-1.997921982
40	H40	0.758536634	-1.432916118	-2.696941959
41	H41	-0.190337967	-0.101535425	-3.376076037
42	H42	-0.863677030	-1.744323465	-3.337295491
43	H43	-2.875614660	-2.306923367	1.032967237
44	H44	1.794392999	2.111508886	3.250789943
45	H45	-0.194096151	-2.615656643	-0.397381033
46	H46	3.119873106	3.373833547	0.317834937
47	H47	3.800648850	3.310441562	-2.072979723
48	H48	-2.909880175	-1.433494803	6.242070658
49	H49	-0.996687633	0.111000711	5.777111047
50	H50	-4.014804685	-2.640662018	4.365554873

4 lowest-energy conformers used for Boltzmann-averaged ^{13}C NMR data.
Avg. Energy: -1265.866326 au.

V.28. 3-Hydroxyblancoxanthone,[2] also call macluraxanthone[9] (B31-10).

No	type	δcal	δexp	diff
C1	C	159.9 (-2.9)	156.5*	3.4 (-2.9)
C2	C	106.8	104.9	1.9
C3	C	159.2	158.8	0.4
C4	C	111.3	113.6	-2.2
C4a	C	155.9	155.2*	0.7
C5	C	131.2	132.8	-1.6
C6	C	150.3	151.0	-0.7
C7	CH	112.1	112.8	-0.7
C8	CH	120.8 (-2.9)	116.2	4.6 (-2.9)
C8a	C	114.5	113.4*	1.1
C9	C	182.9	181.0	1.9
C9a	C	105.2	102.9*	2.3
C10a	C	144.3	146.0	-1.7

C1'	CH	121.3 (-3.7)	115.5	5.8 (-3.7)
C2'	CH	123.8 (+2.8)	127.2	-3.4 (+2.8)
C3'	C	77.8	78.2	-0.4
C4'^{2}	CH3	27.5	27.2	0.3
C1''	C	42.3	41	1.3
C2''	CH	157.0	152.0	5.0
C3''	CH2	104.2	106.5	-2.3
C4''^{2}	CH3	29.4	29.1	0.3
RMSD		2.44		
Max Abs		5.83		
RMSD+CFx		1.71		
Max abs+CFx		5.05		

mol2 coordinates for lowest energy conformer

1	C1	-4.116490909	-1.515537512	-0.154804915
2	C2	-5.106829504	0.778248461	0.200885974
3	O3	2.145374946	-0.364777696	-3.837985625
4	O4	-0.430700537	-0.238570665	-3.954890362
5	C5	-2.885141951	0.197473261	-2.666921730
6	C6	-4.032771752	0.185277163	-1.989616474
7	C7	5.657422582	-0.343831182	-1.425967865
8	C8	5.471842590	-0.183453328	-0.046609923
9	C9	4.565900942	-0.359310999	-2.272548692
10	O10	-2.781856068	0.485755829	0.060567079
11	O11	1.895295548	0.127530218	0.204003485
12	C12	4.189642000	-0.032423475	0.480670016
13	C13	-0.411809492	-0.098732694	-2.630026388
14	C14	-1.618005450	0.072662459	-1.955505692
15	C15	-1.612044241	0.221779314	-0.555095408
16	C16	-0.441337502	0.180539205	0.226629291
17	C17	2.077871206	-0.230510126	-2.605900492
18	C18	3.269198481	-0.210136131	-1.763215855
19	C19	3.105470906	-0.039106732	-0.388826424
20	C20	0.742883147	0.068903777	-0.513664665
21	C21	0.804227545	-0.094727722	-1.908361825
22	C22	-4.016900586	-0.025874838	-0.496738628
23	C23	0.618890045	-0.644538792	2.354082195
24	C24	-0.378565907	0.345622427	1.761514316
25	C25	1.408417908	-0.446193696	3.410814317
26	C26	-0.020117563	1.806637429	2.085332679
27	C27	-1.698612036	-0.006551002	2.496041395
28	O28	6.537875457	-0.180166690	0.779423230
29	H29	-5.047032712	-1.932320656	-0.553306697
30	H30	-3.280536402	-2.064575329	-0.599177210
31	H31	-4.103117685	-1.660303502	0.930536520
32	H32	-4.998493745	1.842432360	-0.026315628
33	H33	-5.046363033	0.639995561	1.284654048
34	H34	-6.092790742	0.444831333	-0.137847350
35	H35	0.513876605	-0.316541495	-4.247642454
36	H36	-2.852431745	0.314171283	-3.744452968
37	H37	-4.995887377	0.289364356	-2.480643848
38	H38	6.668401187	-0.460571489	-1.799839135
39	H39	4.684796478	-0.489285007	-3.342234021
40	H40	0.586071512	-1.638896938	1.907135053
41	H41	1.473006989	0.511628955	3.921067879
42	H42	1.997901071	-1.258939706	3.826029454
43	H43	-0.753175137	2.472745848	1.620780532
44	H44	0.973223643	2.066984557	1.707948401
45	H45	-0.041626131	1.990304945	3.165020400
46	H46	-2.063812349	-0.999271215	2.214893036

47	H47	-2.482965926	0.718910245	2.286524850
48	H48	-1.497134685	-0.009262137	3.571384284
49	H49	6.208026356	-0.076438391	1.684949459
50	O50	4.072820329	0.126062400	1.825798968
51	H51	3.138113695	0.032987760	2.091453413

4 lowest-energy conformers used for Boltzmann-averaged ^{13}C NMR data.
Avg. Energy: -1341.097063 au.

V.29. Dulxanthone E (**B31-11**).[41]

No	type	δcal	δexp	diff
C1	C	154.4 (-2.9)	151.5	2.9 (-2.9)
C2	C	114.1	112.4	1.7
C3	C	154.0	151.3	2.7
C4	C	133.9	132.8	1.1
C4a	C	154.5	156.7	-2.2
C5	C	136.9	136.2	0.7
C6	C	157.0	156.7	0.3
C7	CH	107.4	108.4*	-1.0
C8	CH	125.4 (-2.9)	121.1*	4.3 (-2.9)
C8a	C	118.6	117.3	1.3
C9	C	176.4	174.7	1.7
C9a	C	114.1	110	4.1
C10a	C	151.6	151.2	0.4
C1'	CH	121.1 (-3.7)	116.2*	4.9 (-3.7)
C2'	CH	127.3 (+2.8)	130.1	-2.8 (+2.8)
C3'	C	77.5	77.8	-0.3
C4'[2]	CH3	27.8	28.2	-0.4
C1''	CH3	60.0	62.7	-2.7
C1'''	CH3	59.6	61.3	-1.7
C1''''	CH3	55.0	56.3	-1.3
C1'''''	CH3	61.9	62.7	-0.8
RMSD		2.26		
Max Abs		4.90		
RMSD+CFx		1.61		
Max abs+CFx		4.12		

mol2 coordinates for lowest energy conformer

1	O1	0.169397252	1.408922285	-3.293007198
2	C2	0.399625200	-2.340230673	0.188238702
3	C3	-0.158984555	4.757274764	0.445696324
4	C4	0.570484700	-2.391122135	-1.208408823
5	C5	-0.056990107	4.906481654	-0.947066918
6	C6	0.149343308	-1.133489030	0.833722242
7	C7	-0.149553285	3.477949025	1.015520600
8	C8	0.485980341	-1.219997481	-1.956174681
9	C9	0.045167206	3.786729771	-1.753479388
10	O10	-0.105840163	1.160722629	0.784412331
11	C11	0.152901361	1.307198104	-2.074458649
12	C12	0.098496956	0.031526931	0.060276539
13	C13	-0.067874271	2.368676932	0.173472575
14	C14	0.234296472	0.027412343	-1.338984214
15	C15	0.045959064	2.500534158	-1.210675910
16	O16	0.530322409	-3.438413436	0.952139719
17	C17	0.287901515	-4.736267556	0.358667270
18	C18	0.819452759	-4.788556502	-1.054542462
19	C19	0.916206638	-3.682879166	-1.793469012
20	C20	1.025997037	-5.713086648	1.267102057
21	C21	-1.222984586	-4.986786038	0.375990171
22	C22	-0.446988074	-1.388559608	-4.099474933
23	O23	0.720402109	-1.317378534	-3.293407903

24	O24	-0.071920973	-1.105787780	2.176607030
25	C25	1.009820919	-0.602247538	2.948624780
26	O26	-0.269401354	5.776847033	1.323320631
27	C27	-1.482830392	3.256441590	2.917525590
28	O28	-0.174183499	3.305466833	2.366838101
29	C29	-0.236596386	7.096123426	0.820915295
30	H30	-0.056758000	5.891563246	-1.397990492
31	H31	0.127623694	3.879992468	-2.831137454
32	H32	1.084951446	-5.764448931	-1.450940554
33	H33	1.271686702	-3.699402062	-2.817690542
34	H34	0.658103576	-5.623988839	2.293366696
35	H35	2.099024691	-5.501933634	1.258570616
36	H36	0.866362526	-6.740583900	0.925329944
37	H37	-1.449403559	-5.974691972	-0.038403598
38	H38	-1.602392626	-4.935524832	1.401397417
39	H39	-1.739056102	-4.235500238	-0.230026113
40	H40	-0.099387684	-1.491192350	-5.128728335
41	H41	-1.048562800	-2.265334105	-3.823133566
42	H42	-1.039025694	-0.474904715	-4.007115682
43	H43	1.136574733	0.475005882	2.798198942
44	H44	0.750732645	-0.795720930	3.992534113
45	H45	1.938978813	-1.129246254	2.703681241
46	H46	-1.357941728	3.102486749	3.991336782
47	H47	-2.015232349	4.198765855	2.743154038
48	H48	-2.055508428	2.421526979	2.496064001
49	H49	-1.077471320	7.290053789	0.143478809
50	H50	0.706233771	7.302618594	0.299949152
51	H51	-0.317139909	7.746953847	1.692184719

9 lowest-energy conformers used for Boltzmann-averaged ^{13}C NMR data.
Avg. Energy: -1378.144132 au.

V.30. Pyranojacareubin (B31-15–B34-6).[26]

C	Type	Theory	Expt.	diff.
C1	C	160.7 (-2.9)	157.8	2.9 (-2.9)
C2	C	105.2	104.8	0.4
C3	C	161.3	159.4	1.9
C4	CH	95.3	95.4	-0.1
C4a	C	157.9	156.9	1.0
C5	C	135.2	132.1	3.1
C6	C	144.3	144.8	-0.5
C7	C	118.4	117.9	0.5
C8	CH	116.2 (-2.9)	113.6	2.6 (-2.9)
C8a	C	117.6	114.7	2.9
C9	C	182.3	178.8	3.5
C9a	C	104.9	103.8	1.1
C10a	C	145.5	145.0	0.5
C1'	CH	120.7 (-3.7)	115.5	5.2 (-3.7)
C2'	CH	123.7 (+2.8)	127.6	-3.9 (+2.8)
C3'	C	78.1	78.2	-0.1
C4'[2]	CH3	28.0	28.4	-0.4
C1''	CH	125.2 (-3.7)	121.5	3.7 (-3.7)
C2''	CH	129.7 (+2.8)	131.1	-1.4 (+2.8)
C3''	C	78.1	79.0	-0.9
C4''[2]	CH3	27.1	28.5	-1.4
RMSD		2.25		
Max Abs		5.19		
RMSD+CFx		1.45		
Max abs+CFx		3.54		

1	C1	-1.157112115	0.694990033	-1.019316919

2	C2	-1.177083098	-0.155567976	0.091695136
3	O3	-0.055657853	-0.639105592	0.678411901
4	C4	1.164968411	-0.291694415	0.196859891
5	C5	1.301161487	0.547996554	-0.923896358
6	C6	0.134574113	1.091365504	-1.589180395
7	O7	0.211804435	1.846039948	-2.572043248
8	C8	2.257012721	-0.818930008	0.863573743
9	C9	3.528284131	-0.489800549	0.400237364
10	C10	3.730334839	0.357451824	-0.704520146
11	C11	2.612580175	0.870810268	-1.363198453
12	C12	-2.360223329	1.159525742	-1.562379895
13	C13	-3.572119358	0.793182922	-1.002794611
14	C14	-3.566209561	-0.055547815	0.117281091
15	C15	-2.389755451	-0.543485361	0.671254826
16	O16	4.572307547	-0.974634873	1.094800168
17	C17	5.866433028	-1.089634702	0.448579229
18	C18	6.115594859	0.080077294	-0.473454791
19	C19	5.098585617	0.726129153	-1.045367352
20	C20	-4.882178401	1.210833229	-1.497521930
21	O21	2.800779870	1.679128701	-2.407278006
22	O22	-4.716512373	-0.494677092	0.694552928
23	C23	-5.872046634	0.378422957	0.622147781
24	C24	6.864519642	-1.118382057	1.599860034
25	C25	5.879619609	-2.404473796	-0.337323085
26	O26	-2.400761187	-1.365760496	1.743595246
27	C27	-5.970893671	0.995513114	-0.756759643
28	C28	-7.060375515	-0.528705195	0.914847029
29	C29	-5.718509532	1.467546605	1.687668107
30	H30	2.127366864	-1.468320554	1.719584224
31	H31	-2.314383659	1.812930545	-2.427474481
32	H32	7.149537666	0.340332224	-0.681027527
33	H33	5.253136796	1.542938300	-1.741782869
34	H34	-4.941803233	1.686073062	-2.472287333
35	H35	1.902704469	1.947534261	-2.729157946
36	H36	6.823976363	-0.181126647	2.161158650
37	H37	6.634962344	-1.945393188	2.278048064
38	H38	7.880079304	-1.254042856	1.214400136
39	H39	5.114511893	-2.384765976	-1.119307684
40	H40	5.683593106	-3.249397768	0.331058583
41	H41	6.853650146	-2.549688133	-0.815696268
42	H42	-3.331268235	-1.484316466	1.987376757
43	H43	-6.958672159	1.295054752	-1.095464529
44	H44	-6.943526834	-1.001618710	1.894680477
45	H45	-7.986017792	0.055231172	0.920160359
46	H46	-7.141186223	-1.309803165	0.153657395
47	H47	-5.641789563	1.017546532	2.683048294
48	H48	-6.583113334	2.138666814	1.671986658
49	H49	-4.820880322	2.063551880	1.496709404

2 lowest-energy conformers used for Boltzmann-averaged ^{13}C NMR data.
Avg. Energy: -1339.906692 au.

V.31. Nigrolineaxanthone I (B31-16–D32-4).[42]

No	type	δcal	δexp	diff
C1	C	160.6 (-2.9)	157.6	3.0 (-2.9)
C2	C	102.5	104.5	-2.0
C3	C	160.3	160.1	0.2
C4	CH	93.3	94.8	-1.5
C4a	C	157.5	156.9	0.6
C5	C	107.9	109.2	-1.3
C6	C	145.2	145.7	-0.5

C7	C	141.3	141.9	-0.6
C8	CH	113.0 (-2.9)	108.7	4.3 (-2.9)
C8a	C	115.9	114	1.9
C9	C	181.8	180	1.8
C9a	C	104.4	103.2	1.2
C10a	C	145.9	146.3	-0.4
C1'	CH	118.9 (-3.7)	115.6	3.3 (-3.7)
C2'	CH	122.1 (+2.8)	127.4	-5.3 (+2.8)
C3'	C	79.6	78.1	1.5
C4'^{2}	CH3	30.6	28.3	2.3
C1''	CH	118.2 (-3.7)	115.4	2.8 (-3.7)
C2''	CH	125.9 (+2.8)	129.9	-4.0 (+2.8)
C3''	C	80.3	79.3	1.0
C4''^{2}	CH3	30.3	28.2	2.1
RMSD		2.38		
Max Abs		5.33		
RMSD+CFx		1.48		
Max abs+CFx		2.53		

mol2 coordinates for lowest energy conformer

1	C1	-2.002935247	0.000000000	-2.489359072
2	C2	-1.091060086	0.000000000	-3.544490106
3	C3	0.282065685	0.000000000	-3.247820134
4	C4	0.755941934	0.000000000	-1.936850755
5	C5	0.105938993	0.000000000	2.685570692
6	C6	-0.714491922	0.000000000	3.804547164
7	C7	-2.120263229	0.000000000	3.699323343
8	C8	-2.696839427	0.000000000	2.449126163
9	C9	-2.495446991	0.000000000	-0.038649490
10	O10	-2.879292958	0.000000000	4.821824429
11	C11	-1.496494725	0.000000000	-4.942493635
12	C12	-0.576958340	0.000000000	-5.905901243
13	C13	0.904133768	0.000000000	-5.629755884
14	C14	1.555413822	1.263009057	-6.196817514
15	C15	1.555413822	-1.263009057	-6.196817514
16	O16	-3.306599376	0.000000000	-2.771239964
17	O17	1.216605783	0.000000000	-4.210965545
18	C18	-0.169481869	0.000000000	-0.907778248
19	O19	-0.242554871	0.000000000	5.071616751
20	O20	-3.726007674	0.000000000	-0.206834652
21	C21	1.548699246	0.000000000	2.900489213
22	C22	2.037491947	0.000000000	4.139568746
23	C23	1.178851961	0.000000000	5.380243138
24	C24	1.430672099	1.264002284	6.203345797
25	C25	1.430672101	-1.264002285	6.203345795
26	C26	-0.506346000	0.000000000	1.425235242
27	C27	-1.554925953	0.000000000	-1.141756717
28	C28	-1.894279724	0.000000000	1.296443244
29	O29	0.332980613	0.000000000	0.353520118
30	H30	1.418703314	-1.307417956	-7.282345515
31	H31	2.627916075	-1.265615353	-5.978206406
32	H32	1.101444875	-2.152932912	-5.751843275
33	H33	1.418703314	1.307417956	-7.282345514
34	H34	2.627916076	1.265615353	-5.978206406
35	H35	1.101444875	2.152932911	-5.751843275
36	H36	2.478050135	1.310493890	6.518771217
37	H37	0.798142659	1.266405723	7.096851692
38	H38	1.205618614	2.153373891	5.608113464
39	H39	2.478050137	-1.310493893	6.518771215
40	H40	0.798142662	-1.266405727	7.096851690

41	H41	1.205618616	-2.153373890	5.608113459
42	H42	-2.273147654	0.000000000	5.577005761
43	H43	-3.787506684	0.000000000	-1.904354412
44	H44	-2.557129154	0.000000000	-5.167652771
45	H45	2.202042509	0.000000000	2.035564460
46	H46	-0.857647379	0.000000000	-6.955505672
47	H47	3.109142495	0.000000000	4.318798265
48	H48	-3.775381446	0.000000000	2.340084092
49	H49	1.818972578	0.000000000	-1.733291433

2 lowest-energy conformers used for Boltzmann-averaged ^{13}C NMR data.
Avg. Energy: -1339.906559 au.

V.32. Mangostenone A (B31-19–D32-5).[28]

C	Type	Theory	Expt.	diff.
C1	C	161.1 (-2.9)	157.9	3.2 (-2.9)
C2	C	105.3	104.3	1
C3	C	160.9	159.6	1.3
C4	CH	93.9	93.9	0
C4a	C	156.6	156	0.6
C5	C	108.8	106.9	1.9
C6	C	144.4	144.7	-0.3
C7	C	140	139.4	0.6
C8	C	132.7 (-2.9)	127.9	4.8 (-2.9)
C8a	C	115.2	111.9	3.3
C9	C	184.7	182.4	2.3
C9a	C	105.2	103.8	1.4
C10a	C	147.3	147.3	0
C1'	CH	121.0 (-3.7)	115.8	5.2(-3.7)
C2'	CH	123.5 (+2.8)	127.0	-3.5 (+2.8)
C3'	C	78	77.8	0.2
C4'[2]	CH3	27.9	28.2	-0.3
C1''	CH	120.6 (-3.7)	115.5	5.1 (-3.7)
C2''	CH	127 (+2.8)	129.0	-2 (+2.8)
C3''	C	78.8	79.2	-0.4
C4''[2]	CH3	27.2	28.4	-1.2
C1'''	CH2	27	25.7	1.3
C2'''	CH	124.7	122.4	2.3
C3'''	C	131.6	132.4	-0.8
C4'''	CH3	25.7	26	-0.3
C5'''	CH3	18.6	18.1	0.5
RMSD		2.23		
Max Abs		5.19		
RMSD+CFx		1.27		
Max abs+CFx		3.29		

mol2 coordinates for lowest energy conformer

1	C1	0.620930686	-0.729077295	-0.541306685
2	C2	0.823763394	-0.055782101	0.673220620
3	O3	-0.188086454	0.341027198	1.491902350
4	C4	-1.477953831	0.084922928	1.177210060
5	C5	-1.802822026	-0.597760949	-0.003301177
6	C6	-0.766986372	-1.040500000	-0.920672854
7	O7	-1.065406687	-1.649614243	-1.963564556
8	C8	-2.433649409	0.535989518	2.071884711
9	C9	-3.770598842	0.284730449	1.776497153
10	C10	-4.163225822	-0.411793135	0.619657643
11	C11	-3.177934823	-0.846634269	-0.268595389
12	C12	1.748201359	-1.099950734	-1.328318958
13	C13	3.014150172	-0.780762508	-0.857239170
14	C14	3.179471116	-0.117091094	0.365085075

15	C15	2.100086204	0.265168065	1.145275395
16	O16	-4.681407455	0.688807290	2.679266199
17	C17	-6.053396275	0.899036265	2.256092047
18	C18	-6.474740539	-0.150489628	1.255307519
19	C19	-5.576111233	-0.726749019	0.455810474
20	C20	1.658403770	-1.799320585	-2.671714165
21	C21	1.290300251	-0.836613541	-3.775920765
22	O22	4.124859260	-1.117462376	-1.565295708
23	O23	-3.557802755	-1.507295000	-1.361340676
24	C24	-6.142001839	2.297090148	1.636637590
25	C25	-6.867132196	0.808139331	3.541537989
26	C26	2.088416099	-0.332337459	-4.721549496
27	C27	1.535194681	0.609207493	-5.762396977
28	C28	3.558593799	-0.624424206	-4.869731427
29	H29	-2.152857321	1.067637965	2.971902790
30	H30	-7.534259516	-0.381526894	1.189266143
31	H31	-5.856410405	-1.453865154	-0.298454690
32	H32	0.896932687	-2.578774537	-2.623385815
33	H33	2.619336810	-2.276296433	-2.869748019
34	H34	0.242807583	-0.542297055	-3.772880771
35	H35	-2.727374616	-1.732853061	-1.855342512
36	H36	-7.172439198	2.512345866	1.336032820
37	H37	-5.818509755	3.053980265	2.358708015
38	H38	-5.507203519	2.358987676	0.747205650
39	H39	-7.922127551	1.009394970	3.333559735
40	H40	-6.509568798	1.541636068	4.269227966
41	H41	-6.778609390	-0.190280348	3.976592544
42	H42	2.025083116	1.590200549	-5.703855932
43	H43	0.457876987	0.759625333	-5.645188989
44	H44	1.717972895	0.224591012	-6.774224258
45	H45	4.123502243	0.314776579	-4.933121614
46	H46	3.970722280	-1.200036229	-4.040177145
47	H47	3.749687931	-1.166502950	-5.805941332
48	C48	2.380885297	1.006835944	2.370257472
49	C49	3.636045533	1.071803967	2.818562729
50	H50	4.887141214	-0.819016371	-1.047361647
51	H51	1.564164302	1.506451539	2.879002074
52	H52	3.890193970	1.626722070	3.717164778
53	C53	4.758221369	0.330175047	2.125381013
54	O54	4.456564960	0.178004704	0.712853289
55	C55	4.934239502	-1.066036363	2.727155996
56	H56	5.744310453	-1.602209805	2.219783169
57	H57	4.008966605	-1.642160639	2.627463516
58	H58	5.176014813	-0.990082163	3.791834814
59	C59	6.056080033	1.127214380	2.157859734
60	H60	6.841775732	0.597064994	1.610992483
61	H61	6.386935236	1.263401006	3.192068149
62	H62	5.910784280	2.110627523	1.702371021

5 lowest-energy conformers used for Boltzmann-averaged ^{13}C NMR data.
Avg. Energy: -1535.258263 au.

V.33. 12-Hydroxy-2,2-dimethylpyrano[3,2-b]xanthen-6(2H)-one (B34-1).[12]

No	type	δcal	δexp	diff
C1	CH	117.7 (-2.9)	114.4	3.3 (-2.9)
C2	C	114.5	117.8	-3.3
C3	C	143.6	145.5	-1.9
C4	C	133.4	132.2	1.2
C4a	C	146.1	144.5	1.6
C5	CH	117.4	117.9	-0.5
C6	CH	133.1	134.4	-1.3

C7	CH	122.1	124	-1.9
C8	CH	129.5 (-2.9)	126.7	2.8 (-2.9)
C8a	C	123.3	121.6	1.7
C9	C	177.5	176.4	1.1
C9a	C	118.4	116.1	2.3
C10a	C	156.1	155.9	0.2
C1'	CH	123.5 (-3.7)	121.5	2.0 (-3.7)
C2'	CH	126.5 (+2.8)	131	-4.5(+2.8)
C3'	C	79.7	78.7	1.0
C4'[2]	CH3	30.8	28.5	2.3
RMSD		2.21		
Max Abs		4.46		
RMSD+CFx		1.68		
Max abs+CFx		3.34		

mol2 coordinates for lowest energy conformer

1	O1	2.762889973	0.000000000	2.724410794
2	C2	-0.210411114	0.000000000	-1.325923273
3	C3	-1.288172331	0.000000000	5.720651360
4	C4	1.188012374	0.000000000	-1.212274910
5	C5	0.082223256	0.000000000	6.013092788
6	C6	-1.036679774	0.000000000	-0.208130882
7	C7	-1.733223910	0.000000000	4.410386755
8	C8	1.752737853	0.000000000	0.051540245
9	C9	1.000798587	0.000000000	4.980525613
10	O10	-1.296797164	0.000000000	2.112563658
11	C11	1.554497861	0.000000000	2.538902316
12	C12	-0.442972472	0.000000000	1.058620221
13	C13	-0.795306694	0.000000000	3.376182582
14	C14	0.949965250	0.000000000	1.196567467
15	C15	0.575674620	0.000000000	3.645307235
16	O16	-0.868178850	0.000000000	-2.511609981
17	C17	-0.174770897	0.000000000	-3.786842992
18	C18	1.326146099	0.000000000	-3.633779878
19	C19	1.949890119	0.000000000	-2.456593568
20	O20	-2.384766269	0.000000000	-0.327550482
21	C21	-0.635252934	-1.263639077	-4.515813466
22	C22	-0.635252934	1.263639077	-4.515813466
23	H23	-2.013616505	0.000000000	6.528543788
24	H24	0.417887354	0.000000000	7.044926620
25	H25	-2.788817520	0.000000000	4.161730223
26	H26	2.830005059	0.000000000	0.185022363
27	H27	2.070060889	0.000000000	5.166180653
28	H28	1.884815883	0.000000000	-4.565875395
29	H29	3.034382786	0.000000000	-2.396029290
30	H30	-2.575923280	0.000000000	-1.277367485
31	H31	-0.192329557	-1.311568224	-5.515825140
32	H32	-0.332518838	-2.153331576	-3.956809422
33	H33	-1.725074262	-1.263840203	-4.618140243
34	H34	-0.332518838	2.153331576	-3.956809422
35	H35	-1.725074261	1.263840203	-4.618140243
36	H36	-0.192329557	1.311568224	-5.515825140

2 lowest-energy conformers used for Boltzmann-averaged ^{13}C NMR data.
Avg. Energy: -995.280417 au.

V.34. 7,9,12-Trihydroxy-2,2-dimethyl-2H,6H-pyrano[3,2-b]xanthen-6-one (B34-2).[43]

No	type	δcal	δexp	diff.
C1	C	165.7 (-2.9)	162.8	2.9 (-2.9)
C2	CH	96.4	97.9	-1.5
C3	C	162.2	165.2	-3.0

C4	CH	92.5	93.9	-1.4
C4a	C	158.5	157.2	1.3
C5	C	133.2	133.0	0.2
C6	C	144.0	146.0	-2.0
C7	C	114.7	113.7*	1.0
C8	CH	116.8 (-2.9)	112.0	4.8 (-2.9)
C8a	C	116.7	118.2*	-1.5
C9	C	181.9	179.4	2.5
C9a	C	104.7	101.6	3.1
C10a	C	145.8	146.0	-0.2
C1'	CH	123.3 (-3.7)	121.1	2.2 (-3.7)
C2'	CH	126.8 (+2.8)	131.6	-4.8 (+2.8)
C3'	C	80.0	77.6	2.4
C4'^{2}	CH3	30.8	27.9	2.9
RMSD		2.57		
Max Abs		4.83		
RMSD+CFx		2.06		
Max abs+CFx		3.07		

mol2 coordinates for lowest energy conformer

1	C1	-0.719988694	-1.263670955	4.828123247
2	C2	-0.719988695	1.263670955	4.828123246
3	O3	2.762823844	0.000000000	-2.362885933
4	O4	-2.173027914	0.000000000	-6.387650357
5	O5	2.341505757	0.000000000	-4.935666807
6	O6	-2.420318063	0.000000000	0.620757798
7	C7	1.890102503	0.000000000	2.800433844
8	C8	1.252161072	0.000000000	3.969990130
9	C9	0.122560943	0.000000000	-5.691875969
10	C10	-1.721036003	0.000000000	-4.095121785
11	C11	1.723126034	0.000000000	0.290829239
12	O12	-0.928032128	0.000000000	2.821275114
13	O13	-1.301132845	0.000000000	-1.807490069
14	C14	-1.244410912	0.000000000	-5.406136781
15	C15	1.142777738	0.000000000	1.547449276
16	C16	1.040292056	0.000000000	-4.648226676
17	C17	-1.071864228	0.000000000	0.516786264
18	C18	-0.257075624	0.000000000	1.644329121
19	C19	1.532667556	0.000000000	-2.196792340
20	C20	-0.795155273	0.000000000	-3.067206214
21	C21	0.930674750	0.000000000	-0.861292382
22	C22	-0.463775278	0.000000000	-0.741822243
23	C23	0.592575148	0.000000000	-3.300570935
24	C24	-0.250294827	0.000000000	4.105609109
25	H25	-1.810971750	-1.263971286	4.916569516
26	H26	-0.409900596	-2.153329791	4.273112827
27	H27	-0.289975577	-1.311735370	5.833765402
28	H28	-0.409900596	2.153329791	4.273112827
29	H29	-0.289975577	1.311735370	5.833765403
30	H30	-1.810971751	1.263971286	4.916569515
31	H31	-1.730964935	0.000000000	-7.245528042
32	H32	2.825950389	0.000000000	-4.072854221
33	H33	-2.623936245	0.000000000	1.567925340
34	H34	2.975265295	0.000000000	2.753050806
35	H35	1.799903363	0.000000000	4.908521601
36	H36	0.491748365	0.000000000	-6.712978756
37	H37	-2.783278531	0.000000000	-3.887530133
38	H38	2.801841231	0.000000000	0.171530017

4 lowest-energy conformers used for Boltzmann-averaged ^{13}C NMR data.
Avg. Energy: -1145.751907 au.

V.35. Garcinenone A (B34-3).[44]

No	type	δcal	δexp	diff
C1	C	155.3 (-2.9)	154.6	0.7 (-2.9)
C2	CH	110.8	110	0.8
C3	CH	123.3	123.8	-0.5
C4	C	135.7	137.8	-2.1
C4a	C	143.0	144.1	-1.1
C5	C	132.8	134	-1.2
C6	C	144.1	147.1	-3.0
C7	C	114.7	115	-0.3
C8	CH	117.5 (-2.9)	113.4	4.1 (-2.9)
C8a	C	116.8	119.7	-2.9
C9	C	183.1	182.1	1.0
C9a	C	109.7	108.9	0.8
C10a	C	145.3	146.5	-1.2
C1'	CH	123.2 (-3.7)	121.9	1.3 (-3.7)
C2'	CH	127.0 (+2.8)	132.5	-5.5 (+2.8)
C3'	C	80.3	78.6	1.7
C4'[2]	CH3	30.7	28.2	2.5
RMSD		2.28		
Max Abs		5.51		
RMSD+CFx		1.88		
Max abs+CFx		2.97		

mol2 coordinates for lowest energy conformer

1	C1	1.035055596	0.000000000	-4.791475456
2	C2	0.083081401	0.000000000	-5.808601860
3	C3	-1.274985673	0.000000000	-5.506252738
4	C4	-1.724852406	0.000000000	-4.191007939
5	C5	-1.027103059	0.000000000	0.410015865
6	C6	-0.202187195	0.000000000	1.529160608
7	C7	1.197060802	0.000000000	1.414982627
8	C8	1.763813852	0.000000000	0.152141876
9	C9	1.554266800	0.000000000	-2.335821739
10	C10	1.958371278	0.000000000	2.659809691
11	C11	1.333408363	0.000000000	3.836293984
12	C12	-0.167216096	0.000000000	3.989790344
13	C13	-0.628609210	1.263807765	4.717169946
14	C14	-0.628609210	-1.263807765	4.717169946
15	O15	2.779237511	0.000000000	-2.510171293
16	O16	-1.277990392	0.000000000	-1.909924106
17	C17	-0.771583456	0.000000000	-3.177160525
18	C18	-0.431362010	0.000000000	-0.852876878
19	C19	0.604754444	0.000000000	-3.443343893
20	C20	0.961049927	0.000000000	-0.993638149
21	O21	-0.859871836	0.000000000	2.712102997
22	O22	2.336575380	0.000000000	-5.110986459
23	O23	-3.056319721	0.000000000	-3.917245261
24	O24	-2.376626307	0.000000000	0.519966023
25	H25	-1.718531412	-1.264321642	4.818250836
26	H26	-0.324468980	-2.153592378	4.158998192
27	H27	-0.187142556	-1.311397448	5.717795119
28	H28	-0.324468980	2.153592378	4.158998192
29	H29	-1.718531412	1.264321642	4.818250836
30	H30	-0.187142556	1.311397448	5.717795119
31	H31	-2.015070123	0.000000000	-6.299720802
32	H32	0.423294829	0.000000000	-6.837840893
33	H33	2.841339005	0.000000000	0.022745936
34	H34	3.042806933	0.000000000	2.600276668

35	H35	1.891692884	0.000000000	4.768532235
36	H36	2.840930529	0.000000000	-4.266107027
37	H37	-3.166654903	0.000000000	-2.955891808
38	H38	-2.577412037	0.000000000	1.467819783

1 lowest-energy conformers used for Boltzmann-averaged ^{13}C NMR data.
Avg. Energy: -1145.745045 au.

V.36. Subelliptenone H (B34-4).[45]

No	type	δcal	δexp	diff
C1	C	147.0 (-2.9)	147	0.0 (-2.9)
C2	C	140.0	140.1	-0.1
C3	CH	120.6	122.8	-1.5
C4	C	123.6	126.7	-3.1
C4a	C	146.3	147.9	-1.6
C5	C	135.1	134.5	0.6
C6	C	145.8	146.8	-1.0
C7	C	118.9	119.4	-0.5
C8	CH	116.3 (-2.9)	113.2	3.1 (-2.9)
C8a	C	115.8	114.9	0.9
C9	C	184.4	183.3	1.1
C9a	C	110.6	109.8	0.8
C10a	C	146.1	147.4	-1.3
C1'	C	40.5	41	-0.5
C2'	CH	148.6	148.1	0.5
C3'	CH2	110.7	111.4	-0.7
C4'[2]	CH3	27.0	27.7	-0.7
C1''	CH	124.9 (-3.7)	122.1	2.8 (-3.7)
C2''	CH	130.0 (+2.8)	132.7	-2.7 (+2.8)
C3''	C	78.0	79.2	-1.2
C4''[2]	CH3	27.5	29.3	-1.8
RMSD		1.58		
Max Abs		3.12		
RMSD+CFx		1.35		
Max abs+CFx		3.08		

mol2 coordinates for lowest energy conformer

1	C1	1.608465458	5.875130434	0.749334611
2	C2	-0.823081255	5.192009065	0.772612238
3	O3	-1.144062748	-0.675930749	-3.781450739
4	C4	-0.653289074	-4.402988060	-0.468399728
5	C5	-1.041479956	-4.401666155	-1.801309412
6	C6	-0.299391458	-3.237170118	0.218093959
7	C7	-1.092814128	-3.200152791	-2.508344988
8	C8	-0.314744532	1.720184027	-2.408723649
9	C9	0.482426219	1.350621121	0.267012573
10	C10	0.229534416	4.157521865	-2.193077460
11	C11	0.463716185	5.180877405	-1.371594542
12	O12	0.984136229	3.631576241	0.432231864
13	O13	-0.003217592	-0.904666808	0.137224255
14	C14	0.109735390	2.802912428	-1.658098781
15	C15	0.506603757	2.616174035	-0.313690343
16	C16	-0.783891042	-0.735189198	-2.596999991
17	C17	-0.350186910	-2.050259784	-0.516087795
18	C18	0.027322746	0.283562245	-0.513030849
19	C19	-0.739506762	-2.002662615	-1.865276449
20	C20	-0.366621159	0.441480235	-1.842935544
21	C21	0.548462612	4.976056452	0.123850505
22	O22	-1.372689956	-5.566754291	-2.412977712
23	C23	0.072572572	-3.231123099	1.709253463
24	O24	0.883017687	1.174443029	1.544699984

25	O25	-1.482104982	-3.261293423	-3.793968433
26	C26	0.289639142	-4.663921862	2.238488879
27	C27	1.397002701	-2.510110173	1.914564158
28	C28	1.710901672	-1.686172488	2.913964400
29	C29	-1.075618289	-2.590535263	2.510576732
30	H30	1.690414469	5.666344319	1.819551175
31	H31	1.337705311	6.927431333	0.616736356
32	H32	2.581615317	5.699633864	0.282175019
33	H33	-0.756631306	5.034000544	1.853721015
34	H34	-1.177828797	6.210464344	0.583386304
35	H35	-1.554555680	4.491885222	0.357170584
36	H36	-0.633161853	-5.365398689	0.028457635
37	H37	-0.607241013	1.840025138	-3.446731971
38	H38	0.134837367	4.297630566	-3.265975549
39	H39	0.561711373	6.197631441	-1.741383686
40	H40	-1.612263331	-5.352378445	-3.326900715
41	H41	0.926962453	0.220028332	1.726793023
42	H42	-1.467484554	-2.333970626	-4.137674771
43	H43	1.067976605	-5.191451253	1.677303551
44	H44	0.602144858	-4.614339893	3.285265526
45	H45	-0.633278101	-5.251220880	2.191205005
46	H46	2.165888871	-2.766732979	1.184631750
47	H47	2.712426540	-1.276975179	3.006520228
48	H48	0.997467710	-1.399140651	3.682553550
49	H49	-0.886236772	-2.655160139	3.587236492
50	H50	-2.009042022	-3.121544796	2.303532661
51	H51	-1.212264385	-1.538713278	2.246485615

6 lowest-energy conformers used for Boltzmann-averaged ^{13}C NMR data.
Avg. Energy: -1341.093130 au.

V.37. Pedunxanthone C (B34-5).[46]

C	Type	Theory	Expt.	diff.
C1	C	164.4 (-2.9)	163.3	1.1 (-2.9)
C2	CH	94.4	96.3	-1.9
C3	C	163.3	166.4	-3.1
C4	C	111.2	114.8	-3.6
C4a	C	156.1	155.9	0.2
C5	C	135.5	134.4	1.1
C6	C	146	146.6	-0.6
C7	C	119.5	119.3	0.2
C8	CH	115.9 (-2.9)	113	2.9 (-2.9)
C8a	C	115.9	114.6	1.3
C9	C	182.9	181.8	1.1
C9a	C	105	103.9	1.1
C10a	C	145.4	146.6	-1.2
C1'	C	42.0	42.0	0
C2'	CH	155.4	151.9	3.5
C3'	CH2	106.8	107.5	-0.7
C4'[2]	CH3	29.6	29.6	0
C1''	CH	125 (-3.7)122.1	2.9 (-3.7)	
C2''	CH	130 (+2.8)	132.5	-2.5 (+2.8)
C3''	C	77.5	79	-1.5
C4''[2]	CH3	27.4	28.3	-0.9
C1'''	CH3	54	56.2	-2.2
RMSD		1.82		
Max Abs		3.64		
RMSD+CFx		1.57		
Max abs+CFx		3.64		

mol2 coordinates for lowest energy conformer

1	O1	0.638772160	0.510493807	-3.907293721
2	O2	-1.775485559	-0.034408821	1.547273445
3	O3	3.224994680	0.613379474	-3.749424291
4	C4	-3.088634497	0.211028110	-1.873124350
5	C5	-3.032734133	0.080861483	-0.467660599
6	C6	-1.915454798	0.314801667	-2.602740693
7	O7	5.179847386	0.184239910	0.528925291
8	O8	0.505669050	0.114929281	0.149973356
9	C9	-1.810567348	0.072363890	0.203151167
10	C10	3.068572224	0.480521105	-2.433556141
11	C11	4.197550615	0.428322868	-1.629058395
12	C12	4.079053490	0.285039195	-0.244688183
13	C13	2.825556167	0.229165166	0.413490203
14	C14	0.589923657	0.398954972	-2.672005183
15	C15	-0.677712392	0.286790672	-1.955463606
16	C16	-0.648217564	0.164440336	-0.567168012
17	C17	1.721162732	0.246242976	-0.444818228
18	C18	1.788601505	0.379105022	-1.845213875
19	C19	2.603285856	0.064187264	1.935890302
20	O20	-4.151567205	0.032268809	0.283356583
21	C21	0.639500562	0.827564977	3.383677786
22	C22	1.520753145	1.031734543	2.404705051
23	C23	3.823805401	0.435455101	2.816885952
24	C24	2.241107905	-1.404639464	2.225698750
25	C25	-4.420521999	0.281972644	-2.470998107
26	C26	-5.494788570	-0.022284719	-1.742210170
27	C27	-5.350262668	-0.507858916	-0.317618194
28	C28	-5.257713359	-2.037037371	-0.279368602
29	C29	-6.483260130	0.004077277	0.563759121
30	H30	-0.863879536	0.126619250	1.853583043
31	H31	2.317048836	0.612721145	-4.146035625
32	H32	-1.934312236	0.426106677	-3.682022688
33	H33	5.160707739	0.487094530	-2.116969520
34	H34	-0.030330836	1.619433667	3.705791224
35	H35	0.560334654	-0.119067165	3.912178870
36	H36	1.557159822	2.013383917	1.931913973
37	H37	3.498013698	0.420137285	3.861582807
38	H38	4.201547380	1.436839732	2.591284605
39	H39	4.641531372	-0.274543620	2.695001204
40	H40	1.300389690	-1.688552021	1.746225690
41	H41	3.033841767	-2.057226156	1.848642979
42	H42	2.148563483	-1.582863570	3.302380114
43	H43	-4.503915599	0.598829191	-3.506774062
44	H44	-6.498381621	0.026810646	-2.155722584
45	H45	-5.139180222	-2.380224229	0.753405905
46	H46	-6.163473088	-2.484971581	-0.701458622
47	H47	-4.400558863	-2.380137818	-0.866996390
48	H48	-6.328539566	-0.319141213	1.596579315
49	H49	-7.442327649	-0.389135666	0.212580710
50	H50	-6.519520214	1.096432628	0.540167786
51	C51	6.460207574	0.270247926	-0.060044864
52	H52	6.608163505	1.235548480	-0.558744176
53	H53	6.628668230	-0.542520972	-0.775979956
54	H54	7.167005370	0.176467674	0.765053604

6 lowest-energy conformers used for Boltzmann-averaged ^{13}C NMR data.
Avg. Energy: -1380.388956 au.

V.38. Rheediaxanthone A (B34-8–D31-9).[47]

C	type	δcal	δexp	diff
C1	C	165.4(-2.9)	164.5	0.9 (-2.9)

C2	CH	98.0	99.9	-1.9
C3	C	160.6	161.6	-1.0
C4	C	98.6	102.5	-3.9
C4a	C	153.3	152.8	0.5
C5	C	133.1	134.9	-1.8
C6	C	143.7	147.1	-3.4
C7	C	114.5	115.7	-1.2
C8	CH	116.9 (-2.9)	116.0	0.9 (-2.9)
C8a	C	116.9	119.9	-3.0
C9	C	181.7	181.6	0.1
C9a	C	104.2	104.1	0.1
C10a	C	145.5	147.0	-1.5
C1'	CH	117.8 (-3.7)	113.7	4.1 (-3.7)
C2'	CH	122.2 (+2.8)	128.6	-6.4 (+2.8)
C3'	C	79.7	79.4	0.3
C4'[2]	CH3	30.9	28.8	2.1
C1''	CH	123.3 (-3.7)	122.4	0.9 (-3.7)
C2''	CH	126.6 (+2.8)	133.0	-6.4 (+2.8)
C3''	C	79.9	79.4	0.5
C4''[2]	CH3	30.7	28.7	2.0
RMSD		2.69		
Max Abs		6.39		
RMSD+CFx		2.16		
Max abs+CFx		3.93		

mol2 coordinates for lowest energy conformer

1	C1	1.535333120	1.263761411	-6.232029718
2	C2	1.533601016	-1.261678030	-6.229604445
3	C3	1.303360558	-1.262982284	6.117454716
4	C4	1.299355412	1.265085597	6.112706959
5	O5	-3.845955363	-0.001678349	0.002003305
6	O6	-4.071246024	-0.000262090	-2.597867005
7	O7	1.911395859	-0.001400603	1.615224427
8	C8	-1.727635734	-0.003651135	4.793335439
9	C9	1.530397014	0.002223582	-2.930632183
10	C10	-0.820894180	-0.003005116	5.769184783
11	C11	2.072211467	0.002718698	-4.147412858
12	C12	-2.107917110	0.001361246	-3.877965569
13	C13	-2.186045595	-0.002234000	2.319429338
14	O14	-0.176125379	0.003567557	-5.166529262
15	O15	1.008863006	-0.003563015	4.118700391
16	O16	0.225618239	-0.000334271	-0.456686121
17	C17	-1.313365122	-0.002702204	3.394257021
18	C18	-2.739500277	0.000240603	-2.641504188
19	C19	-0.716019142	0.001931113	-3.937901367
20	C20	0.578518077	-0.001631401	1.850123715
21	C21	0.082488507	0.001291473	-2.781036315
22	C22	0.067551242	-0.002441623	3.143312067
23	C23	-2.612760637	-0.001215497	-0.142256049
24	C24	-1.702668760	-0.001499447	1.007497807
25	C25	-0.322448229	-0.001135156	0.782212991
26	C26	-0.573175856	0.000182606	-1.555398738
27	C27	-1.973277815	-0.000323687	-1.442428111
28	C28	1.254646057	0.002051682	-5.413226130
29	C29	0.668616857	-0.001095898	5.530259098
30	H30	0.926895989	1.266249243	-7.141824401
31	H31	2.591952746	1.306454690	-6.516203221
32	H32	1.295612393	2.154648551	-5.644649498
33	H33	0.925296272	-1.265042079	-7.139488354
34	H34	2.590206099	-1.306453005	-6.513477672

35	H35	1.292509602	-2.151105918	-5.640529872
36	H36	0.865185164	-2.154163650	5.659813523
37	H37	1.136101272	-1.307127265	7.198476583
38	H38	2.382242038	-1.263715800	5.932355940
39	H39	2.378254654	1.268437638	5.927854251
40	H40	0.858549438	2.153150055	5.651469672
41	H41	1.131664574	1.312930654	7.193485972
42	H42	-4.329297049	-0.000984831	-1.642909874
43	H43	2.343117141	-0.001909221	2.482669837
44	H44	-2.790771705	-0.004881112	5.015422299
45	H45	2.143780456	0.002677357	-2.036178368
46	H46	-1.120128751	-0.003531602	6.813741868
47	H47	3.149911679	0.003449390	-4.285063821
48	H48	-2.693369464	0.001847695	-4.788793611
49	H49	-3.260633754	-0.002512550	2.470604747

2 lowest-energy conformers used for Boltzmann-averaged ^{13}C NMR data.
Avg. Energy: -1339.903028 au.

V.39. Mesuarianone (B34-10–D31-10).[48]

No	type	δcal	δexp	diff
C1	C	165.3 (-2.9)	163.3	2.0 (-2.9)
C2	CH	99.5	99.6	-0.1
C3	C	161.3	160.7	0.6
C4	C	101.8	101.6	0.2
C4a	C	153.6	151.8	1.8
C5	C	133.6	132.5	1.1
C6	C	145.8	145.3	0.5
C7	C	115.6	117.8	-2.2
C8	CH	117.1 (-2.9)	113.7	3.4 (-2.9)
C8a	C	117.1	114.8	2.3
C9	C	182.0	180.5	1.5
C9a	C	104.6	103.4	1.2
C10a	C	145.4	145.3	0.1
C1'	CH	120.1 (-3.7)	115.4	4.7 (-3.7)
C2'	CH	124.1 (+2.8)	127.3	-3.2 (+2.8)
C3'	C	78.0	78.4	-0.4
C4'	CH3	28.3	28.5	-0.2
C5'	CH3	27.3	28.5	-1.2
C1''	CH	125.6 (-3.7)	122.2	3.4 (-3.7)
C2''	CH	126.2 (+2.8)	130.0	-3.8 (+2.8)
C3''	C	82.6	81.7	0.9
C4''	CH3	31.2	27.5	3.7
C5''	CH2	43.5	41.8	1.7
C6''	CH2	23.4	22.9	0.5
C7''	CH	125.2	123.6	1.6
C8''	C	133.6	132.3	1.3
C9''	CH3	16.3	17.9	-1.6
C10''	CH3	26.1	25.9	0.2
RMSD		2.05		
Max Abs		4.65		
RMSD+CFx		1.31		
Max abs+CFx		3.66		

mol2 coordinates for lowest energy conformer

1	C1	-0.755381619	-3.949547088	-1.515110438
2	C2	-0.014301525	-4.887528770	-0.807430984
3	C3	0.625178533	-4.507141446	0.368783131
4	C4	0.561463980	-3.193788382	0.869894394
5	C5	-0.833822292	1.236354431	0.551999267
6	C6	-1.469031063	2.280475193	-0.110039944

7	C7	-2.157095063	2.084026143	-1.316007904
8	C8	-2.225602115	0.806347384	-1.845390362
9	C9	-1.618312609	-1.613915848	-1.773348552
10	C10	1.349725442	-2.860558331	2.049226333
11	C11	1.901735020	-3.844235598	2.761235935
12	C12	1.633910000	-5.289732348	2.413045910
13	C13	2.858425568	-6.161237568	2.664815816
14	C14	0.411288480	-5.807508675	3.177089187
15	C15	-2.737454584	3.269318683	-1.938100460
16	C16	-2.518033309	4.478245415	-1.420987149
17	C17	-1.690090814	4.718554649	-0.182189373
18	C18	-2.497099900	5.528161243	0.833842764
19	C19	-0.376312512	5.447764119	-0.520593277
20	C20	0.614819216	4.677660456	-1.404594520
21	C21	1.414039532	3.636391825	-0.667163515
22	C22	1.726987676	2.398825022	-1.065803158
23	C23	1.291934717	1.780317352	-2.367626657
24	C24	2.576837981	1.498418483	-0.206334147
25	O25	-1.366008707	3.482411399	0.503148883
26	C26	-0.188688709	-2.274861189	0.144198296
27	O27	-2.228842064	-1.870432559	-2.823133101
28	C28	-1.598604126	-0.264599224	-1.202191487
29	C29	-0.898885984	-0.038440427	-0.011521997
30	C30	-0.861434002	-2.611737859	-1.041610887
31	O31	-1.370807315	-4.325854391	-2.635423803
32	O32	-0.140047862	1.445571245	1.692762728
33	O33	1.365903504	-5.439123231	0.995259938
34	O34	-0.228925241	-1.014710987	0.649209782
35	H35	0.060100312	-5.906816140	-1.165819985
36	H36	-2.750072937	0.611014880	-2.775328972
37	H37	-3.340797382	3.141454176	-2.832301549
38	H38	-2.933821102	5.369851301	-1.882926187
39	H39	1.490847380	-1.815248732	2.303513914
40	H40	2.517975016	-3.644528393	3.633234838
41	H41	1.313563609	5.413846771	-1.826564336
42	H42	0.081198059	4.246694892	-2.258226945
43	H43	0.110565013	5.726327901	0.423558924
44	H44	-0.652629519	6.386672837	-1.017346490
45	H45	1.807482114	3.970212399	0.295539595
46	H46	-2.734941413	6.518190728	0.431972428
47	H47	-1.920239995	5.656591079	1.754767505
48	H48	-3.430961619	5.012105100	1.072778421
49	H49	2.656703507	-7.193064174	2.362461917
50	H50	3.113783236	-6.152806576	3.729332717
51	H51	3.713711199	-5.788435235	2.094598341
52	H52	0.213100510	-6.851553046	2.913424392
53	H53	0.582614311	-5.741059463	4.256476615
54	H54	-0.470401764	-5.206301808	2.933549150
55	H55	-1.842689526	-3.531455545	-2.988120571
56	H56	-0.103350140	2.407145850	1.812567932
57	H57	0.854411779	0.790090533	-2.192096381
58	H58	0.554746801	2.376627105	-2.909076921
59	H59	2.157609672	1.631972117	-3.025940722
60	H60	2.892913100	1.991123736	0.717588790
61	H61	2.026825674	0.590251250	0.068671849
62	H62	3.474285866	1.177207337	-0.750198915

1 lowest-energy conformers used for Boltzmann-averaged ^{13}C NMR data.
Avg. Energy: -1535.260154 au.

V.40. 12-Hydroxy-2,2-dimethylpyrano[2,3-b]xanthen-11(2H)-one (C21-1).[12]

No	type	δcal	δexp	diff
C1	C	152.0 (-2.9)	148.7	3.3 (-2.9)
C2	C	136.3	135	1.3
C3	C	125.6	129.1	-3.5
C4	CH	102.5	103.2	-0.7
C4a	C	148.8	149.9	-1.1
C5	CH	116.9	117.7	-0.8
C6	CH	134.1	136.9	-2.8
C7	CH	121.8	123.7	-1.9
C8	CH	129.2 (-2.9)	125.8	3.4 (-2.9)
C8a	C	121.7	121.8	-0.1
C9	C	184.0	182	2.0
C9a	C	111.3	109.1	2.2
C10a	C	156.1	156.3	-0.2
C1'	CH	122.5 (-3.7)	120.1	2.4 (-3.7)
C2'	CH	133.0 (+2.8)	135.2	-2.2 (+2.8)
C3'	C	78.0	76.4	1.6
C4'[2]	CH3	31.1	27.7	3.4
RMSD		2.31		
Max Abs		3.53		
RMSD+CFx		1.90		
Max abs+CFx		3.53		

mol2 coordinates for lowest energy conformer

1	O1	-2.561133750	0.000000000	2.178700345
2	C2	1.127154925	0.000000000	-1.251457064
3	C3	0.833272056	0.000000000	5.885805710
4	C4	-0.260720854	0.000000000	-1.396999217
5	C5	-0.568630614	0.000000000	5.907873355
6	C6	1.717243376	0.000000000	0.015995845
7	C7	1.525442473	0.000000000	4.688216616
8	C8	-1.083611904	0.000000000	-0.262681358
9	C9	-1.269563793	0.000000000	4.717845313
10	O10	1.536926428	0.000000000	2.347962250
11	C11	-1.323472258	-0.000000005	2.219551832
12	C12	0.909643444	0.000000000	1.135552996
13	C13	0.807697399	0.000000000	3.489646218
14	C14	-0.493295363	0.000000000	1.023715555
15	C15	-0.590169440	0.000000000	3.491260394
16	C16	1.923985365	0.000000000	-2.475410101
17	C17	1.327637801	0.000000000	-3.665985572
18	C18	-0.169212886	0.000000000	-3.844677606
19	O19	-0.889241829	0.000000000	-2.595725763
20	O20	-2.406245566	0.000000000	-0.434962303
21	C21	-0.608798639	-1.262919057	-4.590676704
22	C22	-0.608798636	1.262919060	-4.590676701
23	H23	1.388188919	0.000000000	6.819032207
24	H24	-1.097505296	0.000000000	6.855040549
25	H25	2.795866690	0.000000000	0.127616892
26	H26	2.609189773	0.000000000	4.652309627
27	H27	-2.354279507	0.000000000	4.694030795
28	H28	3.006629762	0.000000000	-2.386851151
29	H29	1.909219763	0.000000000	-4.584321706
30	H30	-2.809709818	0.000000000	0.465235910
31	H31	-1.697203711	-1.265602675	-4.701801555
32	H32	-0.311941098	-2.153964220	-4.030083437
33	H33	-0.152709199	-1.304778020	-5.585598588
34	H34	-0.311941099	2.153964224	-4.030083445
35	H35	-0.152709203	1.304778019	-5.585598589

36 H36 -1.697203713 1.265602674 -4.701801551

1 lowest-energy conformers used for Boltzmann-averaged ^{13}C NMR data.
Avg. Energy: -995.287359 au.

V.41. 12-Methoxy-2,2-dimethylpyrano[2,3-b]xanthen-11(2H)-one (C21-2).[12]

No	type	δcal	δexp	diff
C1	C	151.2 (-2.9)	147.1	4.1 (-2.9)
C2	C	144.2	141.9	2.3
C3	C	128.9	128.6	0.3
C4	CH	110.0	109.7	0.3
C4a	C	151.1	151.3	-0.2
C5	CH	116.5	117.2	-0.7
C6	CH	133.0	134	-1.0
C7	CH	121.9	123.5	-1.6
C8	CH	129.8 (-2.9)	126.7	3.1 (-2.9)
C8a	C	123.7	128.3	-4.6
C9	C	177.7	176.1	1.6
C9a	C	120.9	116	4.9
C10a	C	155.1	155.3	-0.2
C1'	CH	123.9 (-3.7)	121.5	2.4 (-3.7)
C2'	CH	135.8 (+2.8)	136.5	-0.7 (+2.8)
C3'	C	76.4	76.5	-0.1
C4'[2]	CH3	27.1	27.7	-0.6
C1''	CH3	60.5	61.4	-0.9
RMSD		2.20		
Max Abs		4.91		
RMSD+CFx		1.87		
Max abs+CFx		4.91		

mol2 coordinates for lowest energy conformer

1	O1	-2.175801000	0.444739357	2.398045509
2	C2	1.216454390	-0.590638336	-1.285298345
3	C3	1.507049483	0.303642648	5.792736488
4	C4	-0.141635508	-0.243220691	-1.363932692
5	C5	0.145694178	0.620287608	5.901346115
6	C6	1.845870065	-0.613096269	-0.048944760
7	C7	2.064219167	-0.015845037	4.567072587
8	C8	-0.866522555	0.066810388	-0.215676093
9	C9	-0.649474316	0.612790589	4.771816792
10	O10	1.843092800	-0.342464940	2.258590239
11	C11	-0.969056025	0.275531700	2.315770661
12	C12	1.124205462	-0.307111572	1.099202939
13	C13	1.249381081	-0.014703353	3.433500078
14	C14	-0.243075023	0.009657542	1.052743911
15	C15	-0.106930784	0.298009341	3.518686735
16	C16	1.894445902	-0.916201739	-2.538441848
17	C17	1.299705110	-0.636195063	-3.698801059
18	C18	-0.048813467	0.050093308	-3.737999885
19	O19	-0.801833064	-0.260449010	-2.550954208
20	C20	-3.116638585	-0.566466785	-0.324151759
21	O21	-2.150682539	0.475196201	-0.361127661
22	C22	0.129219765	1.570548092	-3.823580533
23	C23	-0.904059497	-0.474354036	-4.886855574
24	H24	2.135783396	0.306741287	6.677630294
25	H25	-0.279459906	0.867849110	6.868047979
26	H26	2.897837258	-0.862608498	0.039700321
27	H27	3.114090427	-0.266076004	4.461383134
28	H28	-1.708490851	0.845784379	4.815721414
29	H29	2.876457273	-1.377790888	-2.495032427
30	H30	1.779939846	-0.850363858	-4.649567654

31	H31	-4.081819117	-0.085667062	-0.478942477
32	H32	-3.114928122	-1.061647451	0.652496116
33	H33	-2.933198113	-1.294628091	-1.125171424
34	H34	-0.848458164	2.062179500	-3.838596469
35	H35	0.693393992	1.935903424	-2.958800140
36	H36	0.677282817	1.840665849	-4.730932256
37	H37	-1.891282950	-0.003692158	-4.859873219
38	H38	-1.028627154	-1.557534282	-4.803774195
39	H39	-0.433335673	-0.245675198	-5.848036634

1 lowest-energy conformers used for Boltzmann-averaged ^{13}C NMR data.
Avg. Energy: -1034.571188 au.

V.42. 5-Hydroxy-2,2-dimethylpyrano[2,3-b]xanthen-11(2H)-one (C24-1).[12]

No	type	δcal	δexp	diff
C1	CH	106.4 (-2.9)	100.7	5.7 (-2.9)
C2	C	149.9	148.6	1.3
C3	C	114.7	115.3	-0.6
C4	C	141.4	141.1	0.3
C4a	C	138.3	140.4	-2.1
C5	CH	116.1	117.7	-1.6
C6	CH	133.3	134	-0.7
C7	CH	122.3	123.2	-0.9
C8	CH	130.4 (-2.9)	125.8	4.6 (-2.9)
C8a	C	123.3	120.6	2.7
C9	C	177.6	175.8	1.8
C9a	C	124.2	120.8	3.4
C10a	C	154.9	155.2	-0.3
C1'	CH	119.8 (-3.7)	116.3	3.5 (-3.7)
C2'	CH	131.2 (+2.8)	132.9	-1.7 (+2.8)
C3'	C	76.7	75.4	1.3
C4'[2]	CH3	27.9	27.2	0.7
RMSD		2.39		
Max Abs		5.67		
RMSD+CFx		1.61		
Max abs+CFx		3.37		

mol2 coordinates for lowest energy conformer

1	O1	-2.223606182	0.325576648	-2.664852038
2	C2	1.336114262	-0.484240291	0.844224695
3	C3	-5.761935609	0.375562900	0.916719665
4	C4	1.403299043	-0.349840143	-0.559621452
5	C5	-5.842517303	0.519692679	-0.474245145
6	C6	0.099774546	-0.384447258	1.471971997
7	C7	-4.541438931	0.174948775	1.538627509
8	C8	0.267547036	-0.134141647	-1.316207772
9	C9	-4.689985917	0.461540781	-1.234434699
10	O10	-2.213776000	-0.082713901	1.420663760
11	C11	-2.212321705	0.195599052	-1.451524252
12	C12	-1.049058588	-0.161934205	0.703696630
13	C13	-3.385750190	0.117138681	0.758955143
14	C14	-0.975771744	-0.041151567	-0.680322237
15	C15	-3.441685635	0.258602831	-0.630268001
16	C16	2.569887871	-0.764128106	1.569171053
17	C17	3.737247035	-0.643816342	0.935420734
18	C18	3.803939240	-0.166184755	-0.498012287
19	O19	2.588309938	-0.496084674	-1.202505239
20	O20	0.020107803	-0.508767898	2.818600364
21	C21	3.995171020	1.353916749	-0.544679683
22	C22	4.904431023	-0.888850867	-1.267564122
23	H23	-6.663969649	0.419901347	1.519347585

24	H24	-6.804985472	0.676008431	-0.949645057
25	H25	-4.461189365	0.061225895	2.614243670
26	H26	0.324000014	-0.038659124	-2.393742084
27	H27	-4.711295700	0.569078400	-2.314028341
28	H28	2.504844315	-1.073616174	2.606444449
29	H29	4.679115057	-0.847551459	1.437299894
30	H30	-0.912519368	-0.434917774	3.069690471
31	H31	3.162714502	1.857700812	-0.043627218
32	H32	4.041006524	1.695101610	-1.583795499
33	H33	4.922958440	1.638484475	-0.037524493
34	H34	4.902740622	-0.568043098	-2.313211041
35	H35	5.882915883	-0.660461181	-0.833000116
36	H36	4.745683185	-1.970529605	-1.232266841

2 lowest-energy conformers used for Boltzmann-averaged ^{13}C NMR data.
Avg. Energy: -995.282341 au.

V.43. 5-Methoxy-2,2-dimethylpyrano[2,3-b]xanthen-11(2H)-one (C24-2).[12]

No	type	δcal	δexp	diff
C1	CH	111.9 (-2.9)	106.7	5.2 (-2.9)
C2	C	149.1	148.9	0.2
C3	C	123.3	121.6	1.7
C4	C	144.6	143.6	1.0
C4a	C	145.9	144.7	1.2
C5	CH	117.1	118	-0.9
C6	CH	133.4	134.4	-1.0
C7	CH	122.1	123.8	-1.7
C8	CH	129.8 (-2.9)	126.7	3.1 (-2.9)
C8a	C	123.2	121.3	1.9
C9	C	177.8	176.4	1.4
C9a	C	124.9	122.3	2.6
C10a	C	155.7	155.7	0.0
C1'	CH	120.3 (-3.7)	116.4	3.9 (-3.7)
C2'	CH	133.7 (+2.8)	135.6	-1.9 (+2.8)
C3'	C	76.3	76.2	0.1
C4'[2]	CH3	27.3	27.7	-0.4
C1''	CH3	60.5	62.3	-1.8
RMSD		2.07		
Max Abs		5.17		
RMSD+CFx		1.30		
Max abs+CFx		2.58		

mol2 coordinates for lowest energy conformer

1	O1	-0.550718135	2.327266565	-2.819807502
2	C2	0.678076825	-1.341680035	0.419016290
3	C3	-0.313303111	5.726730475	0.887963396
4	C4	0.399688973	-1.350653940	-0.965536733
5	C5	-0.609252952	5.858930703	-0.475745164
6	C6	0.632206187	-0.140389678	1.118914721
7	C7	-0.015952137	4.489893954	1.432218354
8	C8	0.087751238	-0.183312582	-1.629389903
9	C9	-0.603332636	4.740629905	-1.286629269
10	O10	0.284561294	2.181159535	1.195474867
11	C11	-0.297326780	2.281777627	-1.626040715
12	C12	0.310824120	1.045649574	0.443007944
13	C13	-0.013381963	3.366498381	0.602635126
14	C14	0.042483779	1.026788532	-0.926124506
15	C15	-0.304986105	3.476563612	-0.759525081
16	C16	1.046076102	-2.606036006	1.048454375
17	C17	0.862393489	-3.743668576	0.376390839
18	C18	0.228948889	-3.745393298	-0.996997533

19	O19	0.496161872	-2.502450305	-1.678314240
20	O20	0.941635711	-0.119376615	2.448642704
21	C21	-0.188329708	-0.025428687	3.306172167
22	C22	-1.289296097	-3.925762598	-0.882425641
23	C23	0.849620887	-4.813905389	-1.890263020
24	H24	-0.315102464	6.602763251	1.529074112
25	H25	-0.840158057	6.834684018	-0.889699904
26	H26	0.219001602	4.367489338	2.483680967
27	H27	-0.119987030	-0.184646261	-2.693026099
28	H28	-0.826250814	4.801145151	-2.346769099
29	H29	1.471987607	-2.581485576	2.045212929
30	H30	1.125710374	-4.705901816	0.806782520
31	H31	-0.721371560	0.919539039	3.157498094
32	H32	0.197887151	-0.068168882	4.326716752
33	H33	-0.874890514	-0.864623236	3.135995893
34	H34	-1.743803941	-3.926604097	-1.878096715
35	H35	-1.726200417	-3.111150486	-0.296327242
36	H36	-1.525967202	-4.871817534	-0.384331619
37	H37	0.414604264	-4.761248773	-2.892129953
38	H38	1.929992307	-4.663590299	-1.968872722
39	H39	0.659998951	-5.810214990	-1.477799387

1 lowest-energy conformers used for Boltzmann-averaged ^{13}C NMR data.
Avg. Energy: -1034.576938 au.

V.44. 8-Hydroxy-5-methoxy-2,2-dimethylpyrano[2,3-b]xanthen-11(2H)-one (C24-3).[49]

No	type	δcal	δexp	diff
C1	CH	112.0(-2.9)	106.9	5.1(-2.9)
C2	C	149.2	149.1	0.1
C3	C	122.9	121.4	1.5
C4	C	144.6	143.6	1.0
C4a	C	145.8	149.1	-3.3
C5	CH	101.4	103.5	-2.1
C6	C	159.9	161.4	-1.5
C7	CH	110.1	113.9	-3.8
C8	CH	132.5(-2.9)	128.7	3.8(-2.9)
C8a	C	117.1	116.9	0.2
C9	C	176.6	175.6	1.0
C9a	C	125.0	122.4	2.6
C10a	C	157.7	157.3	0.4
C1'	CH	120.4 (-3.7)	116.4	4.0 (-3.7)
C2'	CH	133.5 (+2.8)	135.5	-2.0 (+2.8)
C3'	C	76.1	76.3	-0.2
C4'[2]	CH3	27.2	27.8	-0.6
C1''	CH3	60.5	62.4	-1.9
RMSD		2.40		
Max Abs		3.98		
RMSD+CFx		1.89		
Max abs+CFx		4.84		

mol2 coordinates for lowest energy conformer

1	C1	0.233111449	3.261261215	-0.870196733
2	C2	0.066122320	3.178071736	0.517456462
3	O3	0.074288618	1.994846699	1.180021994
4	C4	0.253677430	0.837052631	0.481648999
5	C5	0.436884405	0.795540354	-0.900539702
6	C6	0.432937427	2.051285672	-1.680670946
7	O7	0.580480445	2.076315740	-2.893658238
8	C8	0.249224469	-0.346491691	1.233187654
9	C9	0.424100096	-1.569488140	0.593523100
10	C10	0.603139583	-1.601186141	-0.806577517

11	C11	0.611710016	-0.435099567	-1.543401871
12	C12	0.206120753	4.529641334	-1.463753606
13	C13	0.020862978	5.668712863	-0.709775866
14	C14	-0.142618872	5.556002009	0.682456577
15	C15	-0.121506533	4.313571395	1.299974065
16	C16	0.475550075	-2.836167806	1.316465103
17	C17	0.424958851	-3.978087378	0.628501874
18	C18	0.255436798	-3.973590079	-0.874749560
19	O19	0.830571729	-2.779183470	-1.442394681
20	O20	0.112464559	-0.307368716	2.591398918
21	C21	-1.225056355	-0.111891949	3.031799322
22	C22	1.026490285	-5.117619505	-1.522683005
23	C23	-1.231841184	-4.023666854	-1.243254640
24	O24	-0.326647034	6.637069538	1.475357006
25	H25	0.755134210	-0.453090283	-2.617671391
26	H26	0.336326562	4.582511364	-2.539676259
27	H27	0.002126477	6.647120701	-1.183154578
28	H28	-0.243762924	4.227339248	2.372890787
29	H29	0.567059051	-2.811382839	2.396717662
30	H30	0.465445789	-4.943458934	1.125968472
31	H31	-1.881009125	-0.900165149	2.641825235
32	H32	-1.603606043	0.867840514	2.721506751
33	H33	-1.200317947	-0.163305509	4.121529364
34	H34	0.631753172	-6.081354914	-1.183463190
35	H35	0.930906479	-5.063467810	-2.611004740
36	H36	2.087000486	-5.058128068	-1.260441573
37	H37	-1.349166919	-4.014566257	-2.331673140
38	H38	-1.694999346	-4.932545581	-0.844588013
39	H39	-1.757612849	-3.159597296	-0.824667643
40	H40	-0.315739382	7.436720920	0.935767546

2 lowest-energy conformers used for Boltzmann-averaged ^{13}C NMR data.
Avg. Energy: -1109.806456 au.

V.45. 5,9-Dimethoxy-2,2-dimethylpyrano[2,3-b]xanthen-11(2H)-one (C24-4).[49]

No	type	δcal	δexp	diff
C1	CH	111.5 (-2.9)	106.5	5.0 (-2.9)
C2	C	148.8	148.8	0.0
C3	C	123.0	121.4	1.6
C4	C	144.7	143.6	1.1
C4a	C	146.0	144.8	1.2
C5	CH	119.1	119.4	-0.3
C6	CH	124.1	124.7	-0.6
C7	C	153.6	155.9	-2.3
C8	CH	108.6 (-2.9)	105.6	3.0 (-2.9)
C8a	C	123.9	121.5	2.4
C9	C	177.8	176.3	1.5
C9a	C	124.3	121.7	2.6
C10a	C	149.9	150.7	-0.8
C1'	CH	120.4 (-3.7)	116.4	4.0 (-3.7)
C2'	CH	133.4 (+2.8)	135.6	-2.2 (+2.8)
C3'	C	76.2	76.2	0.0
C4'[2]	CH3	27.3	27.7	-0.4
C1''	CH3	60.4	62.2	-1.8
C1'''	CH3	54.7	55.9	-1.2
RMSD		2.09		
Max Abs		5.04		
RMSD+CFx		1.35		
Max abs+CFx		2.62		

mol2 coordinates for lowest energy conformer

1	C1	-0.285059000	2.780739132	-0.492603081
2	C2	0.147468429	2.674228621	0.825912487
3	O3	0.531646861	1.495742834	1.387481285
4	C4	0.495312792	0.362115284	0.637115346
5	C5	0.086561192	0.338763624	-0.697226981
6	C6	-0.341552914	1.586638502	-1.357438176
7	O7	-0.713693722	1.629787444	-2.521057663
8	C8	0.902222197	-0.820493993	1.273435263
9	C9	0.893158354	-2.019600819	0.569798726
10	C10	0.469935338	-2.033366627	-0.777886086
11	C11	0.073997223	-0.871047185	-1.403159036
12	C12	-0.667431133	4.032397745	-1.000844778
13	C13	-0.614548440	5.153965558	-0.193298389
14	C14	-0.174208920	5.029364296	1.139240775
15	C15	0.203721438	3.806189570	1.646591779
16	C16	1.352898202	-3.276323933	1.152361520
17	C17	1.123444004	-4.417183567	0.500536136
18	C18	0.341542017	-4.432470787	-0.793897859
19	O19	0.504409498	-3.184059626	-1.497482736
20	O20	1.343368035	-0.798836744	2.565654055
21	C21	0.303973882	-0.695409767	3.530428337
22	C22	-1.149960961	-4.645069520	-0.511004754
23	C23	0.881165380	-5.487111840	-1.753658949
24	O24	-0.958860396	6.408599738	-0.576578706
25	C25	-1.387708435	6.586396104	-1.910014989
26	H26	-0.244474231	-0.874326309	-2.438841138
27	H27	-0.995995483	4.060935858	-2.032817742
28	H28	-0.139006382	5.921712229	1.755056303
29	H29	0.547871672	3.701059519	2.669450230
30	H30	1.884543469	-3.242754707	2.096648836
31	H31	1.456475361	-5.373001905	0.895668646
32	H32	-0.208903318	0.269540749	3.455471723
33	H33	-0.422234486	-1.509012616	3.406729438
34	H34	0.782901353	-0.778868690	4.507815042
35	H35	-1.533875217	-3.842298691	0.126132783
36	H36	-1.310405971	-5.597952364	0.003972511
37	H37	-1.713095855	-4.650974164	-1.449301869
38	H38	1.940498270	-5.309530351	-1.955435306
39	H39	0.765108059	-6.486539022	-1.323282094
40	H40	0.333049582	-5.448091315	-2.698958203
41	H41	-0.604211166	6.302235296	-2.623841820
42	H42	-2.294521740	6.004453003	-2.120576008
43	H43	-1.605524841	7.649459432	-2.016294856

2 lowest-energy conformers used for Boltzmann-averaged ^{13}C NMR data.
Avg. Energy: -1149.104743 au.

V.46. 5-Methoxy-2,2,9-trimethylpyrano[2,3-b]xanthen-11(2H)-one (C24-5).[49]

No	type	δcal	δexp	diff
C1	CH	111.8 (-2.9)	106.7	5.1 (-2.9)
C2	C	148.9	148.8	0.1
C3	C	123.1	120.9	2.2
C4	C	144.6	143.6	1.0
C4a	C	145.9	144.7	1.2
C5	CH	117.3	117.7	-0.4
C6	CH	134.6	135.7	-1.1
C7	C	131.7	133.6	-1.9
C8	CH	129.7 (-2.9)	125.9	3.8 (-2.9)
C8a	C	123.1	121.4	1.7
C9	C	177.8	176.5	1.3
C9a	C	124.9	122.2	2.7

C10a	C	154.0	154	0.0
C1'	CH	120.4 (-3.7)	116.4	4.0 (-3.7)
C2'	CH	133.6 (+2.8)	135.5	-1.9 (+2.8)
C3'	C	76.2	76.1	0.1
C4'2	CH3	27.4	27.7	-0.3
C1''	CH3	60.2	62.2	-2.0
C1'''	CH3	20.7	20.8	-0.1
RMSD		2.10		
Max Abs		5.10		
RMSD+CFx		1.32		
Max abs+CFx		2.70		

mol2 coordinates for lowest energy conformer

1	C1	0.178423053	2.906220181	-0.752560058
2	C2	-0.114663645	2.829655247	0.610149161
3	O3	-0.387065538	1.657085585	1.241308490
4	C4	-0.387276505	0.500476229	0.523262109
5	C5	-0.117662825	0.445673981	-0.844875934
6	C6	0.198822560	1.685618905	-1.582541372
7	O7	0.454905265	1.698793198	-2.776785228
8	C8	-0.682341645	-0.672176274	1.234274367
9	C9	-0.704601542	-1.895138879	0.571043074
10	C10	-0.426968575	-1.939921203	-0.812555064
11	C11	-0.137961661	-0.786187252	-1.510755841
12	C12	0.450324827	4.159205854	-1.317350623
13	C13	0.431377334	5.318187341	-0.558428540
14	C14	0.135239121	5.203402558	0.811221100
15	C15	-0.135246947	3.980996080	1.398387329
16	C16	-1.046813147	-3.146306828	1.239312097
17	C17	-0.844881684	-4.299832011	0.599833640
18	C18	-0.216786903	-4.332429268	-0.775646204
19	O19	-0.499459421	-3.113412218	-1.491505038
20	O20	-0.989653226	-0.620925183	2.563574291
21	C21	0.135551427	-0.451597883	3.416350917
22	C22	1.303449090	-4.491628897	-0.661175424
23	C23	-0.827458121	-5.433763820	-1.635092991
24	C24	0.687832732	6.667141025	-1.181196528
25	H25	0.070931561	-0.814831576	-2.574143041
26	H26	0.676877298	4.185788613	-2.379753488
27	H27	0.119328043	6.098977894	1.427692522
28	H28	-0.364023869	3.894267249	2.455300225
29	H29	-1.469214297	-3.100582472	2.237195347
30	H30	-1.089737271	-5.253570027	1.060038575
31	H31	0.865047493	-1.256054719	3.262212142
32	H32	-0.242679748	-0.495790895	4.438486947
33	H33	0.616329298	0.516962333	3.247430597
34	H34	1.755219886	-4.517869879	-1.658033052
35	H35	1.732401142	-3.654063975	-0.102047484
36	H36	1.553812317	-5.418767303	-0.134803685
37	H37	-1.909398713	-5.296954175	-1.716082424
38	H38	-0.395509777	-5.406773480	-2.639508498
39	H39	-0.626928470	-6.415375211	-1.193989090
40	H40	-0.247487310	7.123196305	-1.527372795
41	H41	1.353040482	6.584591518	-2.045484440
42	H42	1.144907913	7.357713334	-0.465386088

1 lowest-energy conformers used for Boltzmann-averaged ^{13}C NMR data.
Avg. Energy: -1073.895539 au.

V.47. 9-Chloro-5-methoxy-2,2-dimethylpyrano[2,3-b]xanthen-11(2H)-one (C24-6).[49]

No	type	δcal	δexp	diff
C1	CH	111.9 (-2.9)	106.7	5.2 (-2.9)
C2	C	149.5	149.3	0.2
C3	C	123.8	121.9	1.9
C4	C	144.7	144.6	0.1
C4a	C	145.8	143.6	2.2
C5	CH	118.4	119.7	-1.3
C6	CH	134.2	134.5	-0.3
C7	C	127.4	126	1.4
C8	CH	129.7 (-2.9)	129.6	0.1 (-2.9)
C8a	C	123.9	121.9	2.0
C9	C	177.0	175.3	1.7
C9a	C	124.4	122.1	2.3
C10a	C	154.1	154.1	0.0
C1'	CH	120.0 (-3.7)	116.3	3.7 (-3.7)
C2'	CH	134.3 (+2.8)	135.8	-1.5 (+2.8)
C3'	C	76.5	76.3	0.2
C4'[2]	CH3	27.2	27.8	-0.6
C1''	CH3	60.7	62.3	-1.6
RMSD		1.94		
Max Abs		5.25		
RMSD+CFx		1.49		
Max abs+CFx		2.75		

mol2 coordinates for lowest energy conformer

1	C1	-0.349884662	3.442678387	-0.822662607
2	C2	-0.048929768	3.364747329	0.538377102
3	O3	0.266678613	2.198824381	1.154295391
4	C4	0.305033934	1.047419786	0.424768219
5	C5	0.029540518	0.995999257	-0.942392952
6	C6	-0.331454494	2.229341979	-1.666820968
7	O7	-0.594204987	2.251689365	-2.858045321
8	C8	0.645754715	-0.118728730	1.124651654
9	C9	0.703140178	-1.334153616	0.451020631
10	C10	0.417010617	-1.377321936	-0.931617975
11	C11	0.086884648	-0.228619585	-1.619368989
12	C12	-0.670312044	4.685312154	-1.383358742
13	C13	-0.683310733	5.811043297	-0.587374126
14	C14	-0.379017737	5.729639614	0.777289512
15	C15	-0.062233010	4.508353661	1.340516697
16	C16	1.091890067	-2.579183528	1.105923144
17	C17	0.918929682	-3.733276367	0.459739655
18	C18	0.277798884	-3.774281047	-0.909293121
19	O19	0.524622688	-2.542288173	-1.619360850
20	O20	0.958565295	-0.061905330	2.452454213
21	C21	-0.173526970	0.004978041	3.309444474
22	C22	-1.237352975	-3.972500878	-0.782737055
23	C23	0.909051980	-4.853644555	-1.781876689
24	Cl24	-1.082297226	7.365265529	-1.277900191
25	H25	-0.126309549	-0.256505664	-2.681459827
26	H26	-0.901874057	4.727535550	-2.441607936
27	H27	-0.392248578	6.625871806	1.386987856
28	H28	0.180067559	4.418405731	2.393113565
29	H29	1.524146555	-2.527265077	2.098802600
30	H30	1.197881345	-4.682212478	0.909209685
31	H31	-0.745518825	0.923776104	3.138329704
32	H32	-0.824649281	-0.865655774	3.160230706
33	H33	0.214167887	0.003111889	4.329949227
34	H34	-1.696395117	-4.003379343	-1.775789419

35	H35	-1.682929175	-3.150258954	-0.213926645
36	H36	-1.458577951	-4.909750835	-0.261320324
37	H37	1.986896343	-4.691038465	-1.869233027
38	H38	0.734384030	-5.842999849	-1.346646286
39	H39	0.468581602	-4.829023676	-2.782310985

1 lowest-energy conformers used for Boltzmann-averaged ^{13}C NMR data.
Avg. Energy: -1494.123529 au.

V.48. 8-(Diethylamino)-5-methoxy-2,2-dimethylpyrano[2,3-b]xanthen-11(2H)-one (C24-7).[49]

No	type	δcal	δexp	diff
C1	CH	112.0 (-2.9)	109.5	2.5 (-2.9)
C2	C	148.7	148.5	0.2
C3	C	122.1	120.4	1.7
C4	C	144.5	143.3	1.2
C4a	C	145.8	144.4	1.4
C5	CH	96.7	96.1	0.6
C6	C	150.6	152.4	-1.8
C7	CH	106.6	106.9	-0.3
C8	CH	132.1 (-2.9)	128.1	4.0 (-2.9)
C8a	C	113.8	111.1	2.7
C9	C	176.2	174.8	1.4
C9a	C	125.6	122.8	2.8
C10a	C	158.2	158.4	-0.2
C1'	CH	120.5 (-3.7)	116.6	3.9 (-3.7)
C2'	CH	132.8 (+2.8)	134.7	-1.9 (+2.8)
C3'	C	76.0	75.9	0.1
C4'[2]	CH3	27.3	27.6	-0.3
C1''	CH3	60.5	62.3	-1.8
C1'''[2]	CH2	45.3	44.8	0.5
C2'''[2]	CH3	12.2	12.5	-0.3
RMSD		1.77		
Max Abs		4.01		
RMSD+CFx		1.19		
Max abs+CFx		2.76		

mol2 coordinates for lowest energy conformer

1	C1	-1.860134776	-0.143840970	-0.744696776
2	C2	-0.542878327	-0.155437740	-1.211394967
3	O3	0.523632417	-0.245266714	-0.372949712
4	C4	0.309682119	-0.336092761	0.970487348
5	C5	-0.960486777	-0.336034065	1.547310920
6	C6	-2.160324126	-0.231463432	0.685001178
7	O7	-3.296265690	-0.219404985	1.140097921
8	C8	1.452890299	-0.433014008	1.775506948
9	C9	1.320190287	-0.523014713	3.157741969
10	C10	0.031252629	-0.512652053	3.731682717
11	C11	-1.093939636	-0.424655152	2.936798140
12	C12	-2.877058855	-0.044569850	-1.705699727
13	C13	-2.602153240	0.033491233	-3.049810254
14	C14	-1.259215480	0.008069430	-3.520379900
15	C15	-0.234988827	-0.085391217	-2.564430367
16	C16	2.464064096	-0.674510781	4.051376273
17	C17	2.282752575	-0.533534505	5.365793454
18	C18	0.928747771	-0.159702197	5.926450293
19	O19	-0.124305802	-0.654050841	5.074539056
20	O20	2.696067737	-0.474257066	1.208557447
21	C21	3.200061485	0.801619040	0.841266106
22	C22	0.683391896	-0.830330763	7.273614974
23	C23	0.798066029	1.364854712	6.033127316

24	N24	-0.977888603	0.078583617	-4.863297096
25	C25	-2.020177304	-0.008883249	-5.874248589
26	C26	0.387642474	0.171219582	-5.352500957
27	C27	1.064908202	-1.189274166	-5.523411894
28	C28	-2.615491831	1.347513145	-6.249657794
29	H29	-2.087376123	-0.425040306	3.370583506
30	H30	-3.901959937	-0.022896178	-1.348510559
31	H31	-3.425846738	0.134955624	-3.745295070
32	H32	0.810897780	-0.133244907	-2.838129674
33	H33	3.429643660	-0.913411581	3.619111641
34	H34	3.103735594	-0.646412822	6.068858187
35	H35	3.262717241	1.460875340	1.716674020
36	H36	2.571008449	1.269097721	0.076036661
37	H37	4.200798681	0.634924769	0.438225825
38	H38	-0.320800107	-0.588679514	7.633678214
39	H39	1.413570860	-0.479930050	8.010982685
40	H40	0.769642566	-1.916704290	7.179417643
41	H41	-0.185198527	1.629575208	6.434655895
42	H42	1.571548766	1.771543374	6.693516773
43	H43	0.911198646	1.826822286	5.046728876
44	H44	-2.801338966	-0.695709914	-5.535732891
45	H45	-1.578829503	-0.476344200	-6.761493192
46	H46	0.356686985	0.693672234	-6.315093096
47	H47	0.969601063	0.814300005	-4.684475262
48	H48	2.085268765	-1.068403388	-5.902478337
49	H49	1.110060639	-1.728091224	-4.572380431
50	H50	0.508314118	-1.809837807	-6.233715657
51	H51	-1.837780037	2.017054952	-6.631827998
52	H52	-3.073935986	1.829310004	-5.380672936
53	H53	-3.379668632	1.232605130	-7.025538851

4 lowest-energy conformers used for Boltzmann-averaged ^{13}C NMR data.
Avg. Energy: -1247.189741 au.

V.49. Caledonixanthone B (C40-1).[12, 50]

No	type	δcal	δexp	diff
C1	CH	119.2 (-2.9)	117.6	1.6 (-2.9)
C2	CH	121.2	121.7	-0.5
C3	C	122.4	122.4	0
C4	C	142.5	141.3	1.2
C4a	C	145.4	145.7	-0.3
C5	CH	117.5	118.4	-0.9
C6	CH	133.4	134.5	-1.1
C7	CH	122	121.3	0.7
C8	CH	129.7 (-2.9)	126.6	3.1 (-2.9)
C8a	C	123.4	123.8	-0.4
C9	C	178.1	176.9	1.2
C9a	C	125.3	126	-0.7
C10a	C	156.2	156.1	0.1
C1'	CH	123.3 (-3.7)	122	1.3 (-3.7)
C2'	CH	129.4 (+2.8)	133.5	-4.1 (+2.8)
C3'	C	79.3	77.5	1.8
C4'2	CH3	31.2	27.9	3.3
RMSD		1.83		
Max abs	4.13			
RMSD+CFx		1.50		
Max abs+CFx		3.35		

mol2 coordinates for lowest energy conformer

1	O1	-2.760081938	-0.002868404	-3.313469849
2	C2	2.165868735	-0.002212966	-4.361980731

3	C3	-1.734946230	0.002891300	1.625798250
4	C4	1.039461138	-0.003427803	-5.196307803
5	C5	-2.946818066	0.001178655	0.916356814
6	C6	2.028761966	-0.000733794	-2.985267373
7	C7	-0.526179319	0.002854484	0.934999438
8	C8	-0.223650335	-0.003246932	-4.637143255
9	C9	-2.956102005	-0.000389399	-0.461342160
10	O10	0.678247776	0.001108176	-1.075399562
11	C11	-1.737311687	-0.001815288	-2.645187252
12	C12	0.746621840	-0.000497708	-2.431352847
13	C13	-0.538013332	0.001118711	-0.469083581
14	C14	-0.389029027	-0.001825783	-3.245343963
15	C15	-1.745763868	-0.000401855	-1.168821335
16	C16	-1.664242224	0.005062048	3.083969665
17	C17	-0.484927393	0.004824431	3.702958915
18	C18	0.833783320	0.002032369	2.971717259
19	O19	0.682262027	0.005563311	1.532377896
20	C20	1.624877587	-1.263678625	3.309921538
21	C21	1.632216867	1.261978279	3.314004003
22	H22	3.160888609	-0.002405520	-4.796695588
23	H23	1.161618859	-0.004524540	-6.274450982
24	H24	-3.879130561	0.001300923	1.474094338
25	H25	2.887229115	0.000246210	-2.322589383
26	H26	-1.120252349	-0.004191124	-5.248660020
27	H27	-3.878154767	-0.001548954	-1.031650270
28	H28	-2.593978907	0.006971739	3.646078668
29	H29	-0.418113631	0.006010908	4.787755163
30	H30	2.571103913	-1.268996218	2.760445564
31	H31	1.051643732	-2.152584143	3.031640668
32	H32	1.840960721	-1.307407357	4.382615574
33	H33	1.063995342	2.155066705	3.038883743
34	H34	1.848803836	1.300706286	4.386779473
35	H35	2.578350255	1.263841877	2.764348987

1 lowest-energy conformers used for Boltzmann-averaged ^{13}C NMR data.
Avg. Energy: -920.050197 au.

V.50. 5-((3-Methylpent-1-yn-3-yl)oxy)-4a,9a-dihydro-9H-xanthen-9-one (C40-2).[12, 51]

No	type	δcal	δexp	diff
C1	CH	120.7 (-2.9)	118.4	2.3 (-2.9)
C2	CH	120.8	121.3	-0.5
C3	C	126.9	126	0.9
C4	C	142.5	141.6	0.9
C4a	C	146.9	145.7	1.2
C5	CH	117.5	117.4	0.1
C6	CH	133.4	132.6	0.8
C7	CH	122.1	121.7	0.4
C8	CH	129.7 (-2.9)	126.6	3.1 (-2.9)
C8a	C	123.5	122.4	1.1
C9	C	178.1	177	1.1
C9a	C	124.9	122.6	2.3
C10a	C	156.1	156.1	0.0
C1'	CH	125.3 (-3.7)	123.9	1.4 (-3.7)
C2'	CH	132.5 (+2.8)	134.6	-2.1(+2.8)
C3'	C	79.9	80.2	-0.3
C4'	CH3	25.4	25.9	-0.5
C5'	CH2	33.0	33.7	-0.7
C6'	CH3	9.0	8.2	0.8
RMSD		1.37		
Max Abs		3.10		
RMSD+CFx		0.76		

Max abs+CFx 1.25

mol2 coordinates for lowest energy conformer

1	O1	-2.748056448	0.812280899	-3.563782476
2	C2	2.085656893	-0.164535566	-4.592396335
3	C3	-1.847546229	0.224425586	1.365856420
4	C4	1.000998217	0.133382337	-5.428077053
5	C5	-3.020152026	0.526226210	0.652466659
6	C6	1.925581919	-0.230002180	-3.219776974
7	C7	-0.648460189	0.039773906	0.679412624
8	C8	-0.244011022	0.363411169	-4.874622482
9	C9	-2.996710809	0.635019078	-0.721048833
10	O10	0.570914177	-0.072296471	-1.318712065
11	C11	-1.761781567	0.545217769	-2.894002271
12	C12	0.662365126	0.004913753	-2.671908888
13	C13	-0.630130499	0.137134513	-0.719477597
14	C14	-0.432097228	0.302596169	-3.487327649
15	C15	-1.798759609	0.436119641	-1.422538776
16	C16	-1.806529396	0.055482223	2.816650720
17	C17	-0.630755912	-0.028767678	3.440900547
18	C18	0.669281365	0.113900505	2.681311945
19	O19	0.501885147	-0.292747810	1.305600117
20	C20	1.753308812	-0.816548769	3.235589170
21	C21	1.131354976	1.574350568	2.697538015
22	H22	3.065778769	-0.346790843	-5.022642573
23	H23	1.140446036	0.181514373	-6.502853556
24	H24	-3.947095743	0.669484101	1.200472070
25	H25	2.751388987	-0.460164195	-2.555613955
26	H26	-1.108497267	0.596274263	-5.487784989
27	H27	-3.886911075	0.869486650	-1.294742664
28	H28	-2.745678712	-0.005022133	3.359439742
29	H29	-0.566163415	-0.153797981	4.518588682
30	H30	1.975793230	-0.498314893	4.262151286
31	H31	2.664688212	-0.654410365	2.648104329
32	H32	0.373436657	2.217864656	2.239567916
33	H33	1.291537894	1.913655030	3.726076382
34	H34	2.067683248	1.678746090	2.140314593
35	C35	1.369632244	-2.294132817	3.208243312
36	H36	0.482418359	-2.490942176	3.820901072
37	H37	2.187977256	-2.911390876	3.592290623
38	H38	1.147209623	-2.611394739	2.185832914

3 lowest-energy conformers used for Boltzmann-averaged ^{13}C NMR data.
Avg. Energy: -959.375594 au.

V.51. Dehydrocycloguanandin (C40-3).[50]

No	type	δcal	δexp	diff
C1	C	164.0 (-2.9)	161.8	2.2 (-2.9)
C2	CH	109.8	110.3	-0.5
C3	CH	136.7	136.5	0.2
C4	CH	105.1	107.4	-2.3
C4a	C	156.9	156.5	0.4
C5	C	142.3	141	1.3
C6	C	123.1	121*	2.1
C7	CH	121.3	121.5	-0.2
C8	CH	118.5 (-2.9)	116.8	1.7 (-2.9)
C8a	C	123.7	126.7*	-3.0
C9	C	183.9	182.1	1.8
C9a	C	110.1	108	2.1
C10a	C	145.4	145.5	-0.1
C1'	CH	123.0 (-3.7)	121.9	1.1 (-3.7)

C2'	CH	130.0 (+2.8)	134.0	-4.0 (+2.8)
C3'	C	79.6	77.5	2.1
C4'[2]	CH3	31.2	27.9	3.3
RMSD		2.11		
Max Abs		4.01		
RMSD+CFx		1.87		
Max abs+CFx		3.27		

mol2 coordinates for lowest energy conformer

1	O1	-2.545213538	-0.004172013	-3.286337878
2	C2	2.413587847	-0.000661404	-4.113081020
3	C3	-1.751769193	0.003248930	1.685743186
4	C4	1.344938484	-0.002763681	-5.002509076
5	C5	-2.928897536	0.001185494	0.919009892
6	C6	2.226761528	0.000883408	-2.735155443
7	C7	-0.510398561	0.003283033	1.055120325
8	C8	0.040683612	-0.003360173	-4.511409137
9	C9	-2.873660295	-0.000743802	-0.457414233
10	O10	0.783956911	0.001851257	-0.899247694
11	C11	-1.536294375	-0.002379161	-2.567783133
12	C12	0.925946206	0.000288499	-2.249497662
13	C13	-0.455067773	0.001447599	-0.347957909
14	C14	-0.185643436	-0.001811714	-3.109537630
15	C15	-1.628799282	-0.000552107	-1.103276240
16	O16	-0.976958338	-0.005384776	-5.373714399
17	C17	-1.751787312	0.006083286	3.145638187
18	C18	-0.603469863	0.005945451	3.820474390
19	C19	0.749405867	0.002125151	3.155104983
20	O20	0.666934058	0.006002354	1.708966300
21	C21	1.522383077	-1.264493213	3.528831466
22	C22	1.532008615	1.261287206	3.533673182
23	H23	3.426090123	-0.000207080	-4.505702629
24	H24	1.497497830	-0.003960938	-6.075602348
25	H25	-3.886638876	0.001241029	1.431526735
26	H26	3.056676773	0.002523133	-2.038824249
27	H27	-3.768430715	-0.002283782	-1.069236789
28	H28	-1.809660604	-0.005494224	-4.842432706
29	H29	-2.707438046	0.008311647	3.662253490
30	H30	-0.589398412	0.007811671	4.907211690
31	H31	2.492949486	-1.270347063	3.023837276
32	H32	0.961740060	-2.152792187	3.224369567
33	H33	1.688571040	-1.308717896	4.610329208
34	H34	0.977840422	2.155020367	3.233365898
35	H35	1.699299070	1.299524386	4.615222054
36	H36	2.502255145	1.262061312	3.028042345

1 lowest-energy conformers used for Boltzmann-averaged ^{13}C NMR data.
Avg. Energy: -994.936235 au.

V.52. 8,11-Dihydroxy-2,2-dimethylpyrano[3,2-c]xanthen-7(2H)-one (C40-4).[52]

No	type	δcal	δexp	diff
C1	C	155.4 (-2.9)	154.2	1.2 (-2.9)
C2	CH	110.6	109.6	1.0
C3	CH	123.4	124.5	-1.1
C4	C	135.6	138.2	-2.6
C4a	C	143.0	144.6	-1.6
C5	C	141.8	141.7	0.1
C6	C	123.2	121.5*	1.7
C7	CH	121.4	122.3	-0.9
C8	CH	119.1 (-2.9)	117.3	1.8 (-2.9)
C8a	C	123.6	127.6*	-4.0

C9	C	183.7	182.4	1.3
C9a	C	110.0	109.4	0.6
C10a	C	144.5	146.1	-1.6
C1'	CH	122.9 (-3.7)	122.1	0.8 (-3.7)
C2'	CH	130.1 (+2.8)	134.9	-4.8 (+2.8)
C3'	C	79.7	78.6	1.1
C4'[2]	CH3	31.1	27.6	3.5
RMSD		2.23		
Max Abs		4.76		
RMSD+CFx		2.08		
Max abs+CFx		4.00		

mol2 coordinates for lowest energy conformer

1	O1	-2.776479436	0.000000000	-3.151559403
2	C2	2.159910199	0.000000000	-4.170205449
3	C3	-1.764593940	0.000000000	1.786850183
4	C4	1.053878839	0.000000000	-5.015502223
5	C5	-2.972698234	0.000000000	1.070537598
6	C6	2.015297396	0.000000000	-2.788014388
7	C7	-0.553175052	0.000000000	1.101489267
8	C8	-0.236934522	0.000000000	-4.492476968
9	C9	-2.977475196	0.000000000	-0.308113015
10	O10	0.652331450	0.000000000	-0.906595842
11	C11	-1.737322121	0.000000000	-2.481423397
12	C12	0.724442117	0.000000000	-2.267570065
13	C13	-0.561969193	0.000000000	-0.300478800
14	C14	-0.412308095	0.000000000	-3.087268953
15	C15	-1.763800773	0.000000000	-1.009908802
16	C16	-1.698243296	0.000000000	3.245296447
17	C17	-0.519934244	0.000000000	3.866308495
18	C18	0.802367996	0.000000000	3.141380534
19	O19	0.655564328	0.000000000	1.699397476
20	C20	1.594889593	-1.263179239	3.484394299
21	C21	1.594889593	1.263179239	3.484394299
22	O22	-1.284087593	0.000000000	-5.328274821
23	O23	3.105855915	0.000000000	-1.975455540
24	H24	3.164765585	0.000000000	-4.579682845
25	H25	1.177923270	0.000000000	-6.092266184
26	H26	-3.907665811	0.000000000	1.623241614
27	H27	-3.898776757	0.000000000	-0.879121091
28	H28	-2.628885899	0.000000000	3.805420439
29	H29	-0.456361620	0.000000000	4.951169240
30	H30	2.545181124	-1.266554987	2.941749931
31	H31	1.026076662	-2.154206364	3.203978187
32	H32	1.805155564	-1.305218902	4.558163710
33	H33	1.026076662	2.154206365	3.203978187
34	H34	1.805155563	1.305218902	4.558163710
35	H35	2.545181124	1.266554987	2.941749931
36	H36	-2.097655118	0.000000000	-4.774282948
37	H37	2.793423922	0.000000000	-1.059462814

1 lowest-energy conformers used for Boltzmann-averaged ^{13}C NMR data.
Avg. Energy: -1070.128558 au.

V.53. Garciniaxanthone B (C40-5).[53]

No	type	δcal	δexp	diff
C1	C	153.9 (-2.9)	153	0.9 (-2.9)
C2	C	129.1	126.6	2.5
C3	CH	123.8	121.4	2.4
C4	C	135.3	135.1	0.2
C4a	C	141.1	140.4	0.7

C5	C	141.9	141.7	0.2
C6	C	127.4	129	-1.6
C7	CH	120.8	121.8	-1.0
C8	CH	120.5 (-2.9)	117.6	2.9 (-2.9)
C8a	C	123.0	121	2.0
C9	C	184.3	182.2	2.1
C9a	C	110.1	108.5	1.6
C10a	C	146.0	144.9	1.1
C1'	C	41.3	40.3	1.0
C2'	CH	145.7	147	-1.3
C3'	CH2	111.6	110.6	1.0
C4'[2]	CH3	24.5	26.7	-2.2
C1''	CH	124.6 (-3.7)	121.8	2.8 (-3.7)
C2''	CH	132.8 (+2.8)	133.8	-1.0 (+2.8)
C3''	C	77.6	78.3	-0.7
C4''[2]	CH3	27.2	27.8	-0.6
RMSD		1.63		
Max Abs		2.89		
RMSD+CFx		1.49		
Max abs+CFx		2.48		

mol2 coordinates for lowest energy conformer

1	C1	6.323054604	1.039032584	-0.573775647
2	C2	-4.014956851	1.197768418	4.062047001
3	C3	-3.475157636	-1.252066872	3.755716496
4	C4	4.835943165	-0.924668445	-2.136657533
5	C5	4.150065741	1.166220812	-3.368100116
6	O6	-0.586970135	-1.405626002	-3.039075504
7	O7	1.337600307	1.418492078	1.886250781
8	O8	1.931379501	-0.758740853	-3.150743955
9	C9	5.185876068	1.383549137	-1.169875269
10	C10	-5.441643770	-0.593570033	1.181433032
11	C11	-5.333393929	-0.202779181	2.452551465
12	C12	-4.229807438	-1.330898537	-0.910589409
13	C13	-3.068512668	-1.387662358	-1.651525309
14	C14	2.682673771	0.927804104	-0.019818695
15	O15	-3.014435071	0.426759909	2.081154030
16	O16	-0.785294316	0.299007003	0.682899055
17	C17	1.461704974	0.889536087	0.639303586
18	C18	-4.240360126	-0.735130790	0.361870739
19	C19	1.786098286	-0.204711680	-1.934481553
20	C20	2.881016076	0.412464290	-1.304445810
21	C21	-3.063095197	-0.202886831	0.884315254
22	C22	-0.633015517	-0.879045571	-1.919830081
23	C23	-1.881469545	-0.840856133	-1.141732485
24	C24	0.388353646	0.300208380	-0.011146455
25	C25	-1.888576859	-0.251730295	0.122972518
26	C26	0.525630837	-0.257156961	-1.289302115
27	C27	-3.978168309	0.029327920	3.084520455
28	C28	4.262644345	0.493040840	-1.979220996
29	H29	6.926552945	1.776818268	-0.053655934
30	H30	6.699912675	0.020435167	-0.579866354
31	H31	-3.015508259	1.377074467	4.470317279
32	H32	-4.356359793	2.106381398	3.557593032
33	H33	-4.695277742	0.976886372	4.890877298
34	H34	-3.391839621	-2.059817349	3.021905958
35	H35	-4.170924956	-1.571718214	4.538397006
36	H36	-2.491822704	-1.080466177	4.206154801
37	H37	4.168577878	-1.535072112	-2.748651377
38	H38	5.815659239	-0.885958179	-2.625382847

39	H39	4.951760587	-1.412742421	-1.163414921
40	H40	5.152573034	1.294957219	-3.791270382
41	H41	3.684634728	2.154960549	-3.284890990
42	H42	3.553975881	0.560348030	-4.051718639
43	H43	0.417584665	1.309576894	2.165342477
44	H44	1.054657085	-1.132329753	-3.410349917
45	H45	4.872838025	2.428266975	-1.122798651
46	H46	-6.407682296	-0.796411834	0.727978597
47	H47	-6.207197117	-0.079844542	3.086531000
48	H48	-5.153737722	-1.743089221	-1.305175062
49	H49	-3.038838045	-1.842313193	-2.635081629
50	H50	3.513277556	1.378376639	0.512445773

2 lowest-energy conformers used for Boltzmann-averaged ^{13}C NMR data.
Avg. Energy: -1265.858635 au.

V.54. 6-Hydroxy-2,2-dimethylpyrano[3,2-c]xanthen-7(2H)-one (C41-1).[12]

No	type	δcal	δexp	diff
C1	C	155.9 (-2.9)	154.7	1.2 (-2.9)
C2	CH	107.9	106.6	1.3
C3	C	126.4	129	-2.6
C4	C	132.3	132.3	0.0
C4a	C	144.3	144.4	-0.1
C5	CH	117.4	118.3	-0.9
C6	CH	134.2	135.3	-1.1
C7	CH	122.1	124	-1.9
C8	CH	129.1 (-2.9)	125.9	3.2 (-2.9)
C8a	C	122.0	120.6	1.4
C9	C	183.7	181.9	1.8
C9a	C	111.2	108.9	2.3
C10a	C	156.3	156.2	0.1
C1'	CH	123.2 (-3.7)	122.2	1.0 (-3.7)
C2'	CH	132.3 (+2.8)	136.2	-3.9 (+2.8)
C3'	C	78.0	76.6	1.4
C4'[2]	CH3	31.0	27.4	3.6
RMSD		2.12		
Max Abs		3.86		
RMSD+CFx		1.90		
Max abs+CFx		3.65		

mol2 coordinates for lowest energy conformer

1	O1	-2.520620199	0.000000000	3.348702877
2	C2	-1.670588350	0.000000000	-1.616002494
3	C3	2.430194998	0.000000000	4.225309559
4	C4	-2.858167498	0.000000000	-0.881744862
5	C5	1.331770893	0.000000000	5.096286640
6	C6	-0.430223693	0.000000000	-0.974809079
7	C7	2.249130592	0.000000000	2.853939619
8	C8	-2.833391367	0.000000000	0.506150233
9	C9	0.049994313	0.000000000	4.580452178
10	O10	0.832273373	0.000000000	0.992283467
11	C11	-1.513114330	0.000000000	2.630437670
12	C12	-0.399707004	0.000000000	0.417828376
13	C13	0.949351409	0.000000000	2.341683300
14	C14	-1.584825163	0.000000000	1.176423368
15	C15	-0.157797635	0.000000000	3.194443773
16	O16	-3.991744862	0.000000000	1.179380824
17	O17	0.759681646	0.000000000	-1.622952918
18	C18	0.845792675	0.000000000	-3.063427282
19	C19	-0.502237590	0.000000000	-3.738491785
20	C20	-1.657813862	0.000000000	-3.076082738

21	C21	1.624563720	-1.262694806	-3.441693288
22	C22	1.624563721	1.262694806	-3.441693288
23	H23	3.438846692	0.000000000	4.627759426
24	H24	-3.817773216	0.000000000	-1.388776327
25	H25	1.490255698	0.000000000	6.169984534
26	H26	3.086068006	0.000000000	2.163935887
27	H27	-0.825272631	0.000000000	5.222267170
28	H28	-3.770338523	0.000000000	2.138468733
29	H29	-0.479735956	0.000000000	-4.825519072
30	H30	-2.608831451	0.000000000	-3.601363309
31	H31	2.593808343	-1.265473635	-2.934277224
32	H32	1.067144998	-2.153739726	-3.139387682
33	H33	1.793894455	-1.304974452	-4.522925689
34	H34	1.793894455	1.304974453	-4.522925689
35	H35	2.593808343	1.265473635	-2.934277224
36	H36	1.067144998	2.153739726	-3.139387682

1 lowest-energy conformers used for Boltzmann-averaged ^{13}C NMR data.
Avg. Energy: -995.286771au.

V.55. 6-Methoxy-2,2-dimethylpyrano[3,2-c]xanthen-7(2H)-one (C41-2).[12]

No	type	δcal	δexp	diff
C1	C	152.9 (-2.9)	153.7	-0.8 (-2.9)
C2	CH	101.8	102.6	-0.8
C3	C	122.4	126.1	-3.7
C4	C	135.4	134.6	0.8
C4a	C	147.5	146.8	0.7
C5	CH	116.8	117.7	-0.9
C6	CH	132.6	135.2	-2.6
C7	CH	121.9	123.8	-1.9
C8	CH	129.7 (-2.9)	126.7	3.0 (-2.9)
C8a	C	124.4	122.9	1.5
C9	C	176.1	176.4	-0.3
C9a	C	116.0	112.7	3.3
C10a	C	154.9	155.0	-0.1
C1'	CH	122.9 (-3.7)	121.9	1.0 (-3.7)
C2'	CH	131.0 (+2.8)	133.9	-2.9 (+2.8)
C3'	C	78.4	76.7	1.7
C4'[2]	CH3	31.2	27.3	3.9
C1''	CH3	54.2	56.5	-2.3
RMSD		2.26		
Max Abs		3.88		
RMSD+CFx		2.25		
Max abs+CFx		3.88		

mol2 coordinates for lowest energy conformer

1	C1	1.185331962	-0.001635273	1.174963931
2	C2	-0.007493849	0.001498943	0.435284004
3	C3	-0.013162952	0.005551789	-0.966904266
4	C4	1.197204986	0.005316186	-1.640670353
5	C5	2.403755372	0.002291068	-0.928209538
6	C6	2.414192988	-0.000836502	0.458949839
7	C7	1.110973519	-0.004700633	2.654901269
8	C8	-0.269361370	-0.003820651	3.197106783
9	C9	-1.373059388	-0.000366682	2.348915503
10	O10	-1.242368454	0.001862010	0.998588160
11	C11	-0.483907789	-0.006115557	4.581327984
12	C12	-1.766891164	-0.004946301	5.094961351
13	C13	-2.863205972	-0.001483063	4.221933681
14	C14	-2.675721974	0.000801270	2.850411787
15	O15	2.075186620	-0.008329638	3.401407863

16	O16	3.544572405	-0.000740013	1.191740073
17	C17	4.780227130	-0.007485949	0.517029986
18	O18	-1.222026707	0.012504490	-1.576736989
19	C19	-1.352739595	0.005631797	-3.014684336
20	C20	-0.025527771	0.009875320	-3.731233606
21	C21	1.147065477	0.009578878	-3.100094903
22	C22	-2.136787500	-1.262113612	-3.364901155
23	C23	-2.148721021	1.263404507	-3.373206641
24	H24	3.327785097	0.003544804	-1.493596805
25	H25	0.391068755	-0.008665842	5.222843801
26	H26	-1.926891689	-0.006654897	6.168064723
27	H27	-3.873163284	-0.000563445	4.620639935
28	H28	-3.508056666	0.003456547	2.155510120
29	H29	5.540673565	-0.011993201	1.298376788
30	H30	4.896100392	-0.903512831	-0.106196335
31	H31	4.904741363	0.887527029	-0.106670295
32	H32	-0.079829131	0.012446974	-4.816667978
33	H33	2.083141954	0.012180555	-3.651356437
34	H34	-2.338694135	-1.307571898	-4.440427421
35	H35	-3.089959088	-1.268776241	-2.827473503
36	H36	-1.565512763	-2.149567645	-3.077475377
37	H37	-1.585087735	2.157983085	-3.093220640
38	H38	-3.101201974	1.265466698	-2.834443831
39	H39	-2.352649613	1.298957924	-4.448787173

2 lowest-energy conformers used for Boltzmann-averaged ^{13}C NMR data.
Avg. Energy: -1034.569639 au.

V.56. Dulcisxanthone H (C41-3).[55]

No	type	δcal	δexp	diff
C1	C	157.6 (-2.9)	155.2	2.4 (-2.9)
C2	CH	107.5	107	0.5
C3	C	130.7	128.7	2.0
C4	C	131.6	131.8	-0.2
C4a	C	145.3	142.5	2.8
C5	C	142.6	142.5	0.1
C6	C	133.7	135.3	-1.6
C7	CH	125.2	125.1	0.1
C8	CH	118.3 (-2.9)	115.8	2.5 (-2.9)
C8a	C	120.5	119.1	1.4
C9	C	184.0	181.8	2.2
C9a	C	110.3	108.5	1.8
C10a	C	144.1	144.4	-0.3
C1'	CH	125.0 (-3.7)	122.2	2.8 (-3.7)
C2'	CH	135.3 (+2.8)	135.9	-0.6 (+2.8)
C3'	C	76.3	77.1	-0.8
C4'[2]	CH3	26.9	28.8	-1.9
C1''	CH2	31.0	27.4	3.6
C2''	CH	123.5	120.9	2.6
C3''	C	132.4	133.9	-1.5
C4''	CH3	25.5	27.4	-1.9
C5''	CH3	17.7	17.9	-0.2
RMSD		1.85		
Max Abs		3.64		
RMSD+CFx		1.68		
Max abs+CFx		3.64		

mol2 coordinates for lowest energy conformer

1	C1	-0.111467982	-1.545183273	-2.672264031
2	C2	-0.566800636	-1.491284627	-1.343974231
3	C3	-1.048263070	-2.620220352	-0.693269776

4	C4	-1.089961044	-3.833619465	-1.388010437
5	C5	-0.636116053	-3.915299136	-2.704696515
6	C6	-0.144272527	-2.788596150	-3.354394511
7	C7	0.382217256	-0.337817443	-3.328376547
8	C8	0.345721119	0.884231489	-2.517238504
9	C9	-0.130830538	0.814299267	-1.211800312
10	O10	-0.568478618	-0.335706857	-0.629610099
11	C11	0.764648162	2.125740404	-3.011849331
12	C12	0.695182141	3.242311362	-2.201956023
13	C13	0.216535600	3.175395105	-0.882511720
14	C14	-0.194960306	1.943468170	-0.386888799
15	O15	-1.539850361	-2.485479325	0.569193008
16	C16	-1.455719691	-3.646104722	1.420552140
17	C17	-1.845855426	-4.889863292	0.650394408
18	C18	-1.648781218	-4.975683722	-0.666419160
19	O19	0.285269031	-2.901925133	-4.616295125
20	O20	0.800404307	-0.340837127	-4.492993046
21	O21	-0.665344453	1.831919221	0.880079673
22	C22	0.191787263	4.396043909	0.009290907
23	C23	1.498202649	4.549977173	0.749961457
24	C24	1.729063577	4.373287806	2.053864488
25	C25	3.113205985	4.563190041	2.620952508
26	C26	0.691608130	3.978162180	3.069553964
27	C27	-2.440232095	-3.368621063	2.550339448
28	C28	-0.021722874	-3.774668587	1.946370316
29	H29	-0.663571704	-4.857835458	-3.241546094
30	H30	1.132616964	2.179339093	-4.030271904
31	H31	1.014246694	4.207276818	-2.586361333
32	H32	-2.255090814	-5.721262438	1.218084481
33	H33	-1.895850618	-5.874754534	-1.224605928
34	H34	0.585303064	-2.006542973	-4.902131492
35	H35	-0.920762096	0.908363103	1.024294548
36	H36	-0.651521240	4.332207888	0.699326513
37	H37	0.028106041	5.281708771	-0.616434515
38	H38	2.343950438	4.812320156	0.114339745
39	H39	3.474702613	3.635458407	3.082076809
40	H40	3.111221965	5.327231440	3.408891518
41	H41	3.832933788	4.866132838	1.855042960
42	H42	-0.286310794	3.778298419	2.631553448
43	H43	0.584458146	4.757833647	3.834164322
44	H44	1.007708888	3.066629537	3.590548324
45	H45	-2.426911432	-4.190431183	3.273850068
46	H46	-2.168231388	-2.444291197	3.068622851
47	H47	-3.454298561	-3.263406053	2.153524993
48	H48	0.679241545	-3.902035965	1.115675385
49	H49	0.254470748	-2.875470325	2.506975768
50	H50	0.068399423	-4.643885843	2.606375382

4 lowest-energy conformers used for Boltzmann-averaged ^{13}C NMR data.
Avg. Energy: -1265.869666 au.

V.57. Methyl 3,3-dimethyl-7-oxo-3H,7H-pyrano[2,3-c]xanthene-11-acetate (D30-1).[56]

No	type	δcal	δexp	diff
C1	CH	130.9 (-2.9)	127.3	3.6 (-2.9)
C2	CH	113.7	114.2	-0.5
C3	C	158.8	158.2	0.6
C4	C	110.7	109.1	1.6
C4a	C	152.7	151.1	1.6
C5	C	125.1	124.6	0.5
C6	CH	134.3	135.6	-1.3
C7	CH	122.2	121.9	0.3

C8	CH	128.7 (-2.9)	126.1	2.6 (-2.9)
C8a	C	123.8	123.6	0.2
C9	C	177.3	174.5	2.8
C9a	C	117.3	115.1	2.3
C10a	C	154.5	151.0	0.9
C1'	CH	120.2 (-3.7)	115.1	5.1 (-3.7)
C2'	CH	128.3 (+2.8)	130.1	-1.8 (+2.8)
C3'	C	77.9	78.1	-0.2
C4'[2]	CH3	27.8	28.4	-0.6
C1''	CH2	38.7	36.1	2.6
C2''	C	173.3	171.4	1.9
C3''	CH3	53.0	52.3	0.7
RMSD		1.98		
Max Abs		5.08		
RMSD+CFx		1.32		
Max abs+CFx		2.80		

mol2 coordinates for lowest energy conformer

1	C1	2.053233486	1.253301212	-2.200486045
2	C2	0.973937474	1.084383931	-3.039306793
3	C3	-0.247443060	0.648024529	-2.506077594
4	C4	-0.391621975	0.359278522	-1.144390368
5	C5	1.316305286	-0.034554909	3.183245584
6	C6	2.345523763	0.059984798	4.106697326
7	C7	3.615538143	0.525884411	3.744787480
8	C8	3.864069284	0.893835942	2.437195513
9	C9	3.106059490	1.172633268	0.065170173
10	C10	1.591285343	0.353845567	1.864479651
11	C11	2.849847111	0.807494258	1.475532652
12	C12	0.727382368	0.541141260	-0.323808823
13	O13	4.191990768	1.579268699	-0.321099060
14	O14	0.549968391	0.248956542	0.993894418
15	C15	1.950915871	0.990949656	-0.825724669
16	C16	-1.668869035	-0.180203694	-0.684315393
17	C17	-2.717731274	-0.148173844	-1.507920298
18	C18	-2.625729316	0.513493185	-2.862599162
19	O19	-1.267503462	0.462099218	-3.365947276
20	C20	-3.038976073	1.985194326	-2.768749211
21	C21	-3.450298993	-0.231833063	-3.904961722
22	C22	-0.064109884	-0.514951376	3.549095690
23	C23	-0.594341332	-1.650257216	2.690301486
24	O24	-1.759566065	-1.790717448	2.398424750
25	O25	0.371800480	-2.494013589	2.315272478
26	C26	-0.041379384	-3.548561555	1.446188543
27	H27	1.036732772	1.285036176	-4.102836767
28	H28	3.010497650	1.600423223	-2.575236249
29	H29	4.836519375	1.251057408	2.114714718
30	H30	4.399549240	0.590267427	4.491972916
31	H31	2.156759454	-0.238959502	5.134455000
32	H32	-1.740782328	-0.617406481	0.306116027
33	H33	-3.680595246	-0.556218323	-1.213947963
34	H34	-2.963836794	2.463577924	-3.750542591
35	H35	-2.392364197	2.517111012	-2.064420217
36	H36	-4.071140848	2.067168156	-2.413004798
37	H37	-3.131158245	-1.275820218	-3.967316070
38	H38	-4.510918992	-0.203410418	-3.636093527
39	H39	-3.326540472	0.232942852	-4.887407639
40	H40	-0.800967175	0.289770482	3.470994664
41	H41	-0.070297971	-0.858417958	4.589874948
42	H42	-0.805064863	-4.165822344	1.923887132

43	H43	0.856167407	-4.132148805	1.246660151
44	H44	-0.442846173	-3.135653241	0.517230936

2 lowest-energy conformers used for Boltzmann-averaged ^{13}C NMR data.
Avg. Energy: -1187.266756 au.

V.58. 3,3-Dimethyl-7-oxo-3H,7H-pyrano[2,3-c]xanthene-11-acetic acid (D30-2).[56]

No	type	δcal	δexp	diff
C1	CH	130.9 (-2.9)	130.7	4.3 (-2.9)
C2	CH	113.7	113.8	-0.1
C3	C	158.8	157.7	1.1
C4	C	110.7	108.6	2.1
C4a	C	152.6	151	1.6
C5	C	124.1	124.5	-0.4
C6	CH	134.7	136.2	-1.5
C7	CH	122.3	120.7	1.6
C8	CH	129.1 (-2.9)	125	4.1 (-2.9)
C8a	C	123.8	123.7	0.1
C9	C	177.2	174.7	2.5
C9a	C	117.3	114	3.3
C10a	C	154.5	153.5	1.0
C1'	CH	120.1 (-3.7)	114.8	5.3 (-3.7)
C2'	CH	127.9 (+2.8)	126.6	-2.8 (+2.8)
C3'	C	78.0	78	0.0
C4'[2]	CH3	27.8	27.8	0.0
C1''	CH2	37.3	35.6	1.7
C2''	C	172.5	171.7	0.8
RMSD		2.31		
Max Abs		5.34		
RMSD+CFx		1.43		
Max abs+CFx		3.29		

mol2 coordinates for lowest energy conformer

1	C1	-1.708582401	-0.876413679	-2.449284660
2	C2	-2.687297069	-0.996017127	-1.487552613
3	C3	-2.382055524	-0.682025028	-0.155152068
4	C4	-1.100334798	-0.269293738	0.224994391
5	C5	3.354446796	0.740267141	-0.735580658
6	C6	4.421384749	0.866580527	-1.611174936
7	C7	4.275487045	0.623279186	-2.982151360
8	C8	3.046422502	0.242150558	-3.484436392
9	C9	0.627766730	-0.324280382	-3.146242328
10	C10	2.117101375	0.355288239	-1.269791564
11	C11	-0.836549467	-0.049986369	1.644943172
12	C12	-1.864029301	-0.014596560	2.494307937
13	C13	-3.289978020	-0.111964223	2.005710250
14	C14	-3.869501452	1.285106002	1.761375635
15	C15	-4.155585155	-0.913849218	2.971394382
16	C16	1.946940900	0.101503701	-2.629024067
17	C17	-0.132422491	-0.161151782	-0.778995667
18	O18	0.430963390	-0.555182343	-4.330085579
19	O19	-3.357337123	-0.847627713	0.758060250
20	C20	-0.413003929	-0.452226942	-2.116179786
21	C21	3.472940121	1.010462681	0.742301606
22	C22	3.116812070	-0.165404353	1.630557896
23	O23	1.102465300	0.242261686	-0.370012673
24	O24	2.523187722	-0.082716211	2.680109830
25	O25	3.575961433	-1.330565224	1.147261440
26	H26	-3.692653873	-1.321098232	-1.729744000
27	H27	-1.911241219	-1.100006025	-3.491420330
28	H28	2.894255916	0.039337676	-4.539408038

29	H29	5.129299435	0.730484840	-3.642804981
30	H30	5.391327147	1.159751638	-1.218489940
31	H31	0.187623543	0.053627529	1.987432481
32	H32	-1.711358462	0.124846745	3.560696186
33	H33	-5.168809567	-1.017968132	2.572182769
34	H34	-3.732585328	-1.910892823	3.122781461
35	H35	-4.212163758	-0.404240081	3.938538675
36	H36	-3.272135330	1.818381029	1.014906284
37	H37	-3.857908988	1.867582283	2.688462602
38	H38	-4.901481464	1.210963325	1.403383065
39	H39	4.504525870	1.292171102	0.981374212
40	H40	2.828211645	1.836725862	1.052508585
41	H41	3.309891031	-2.013265561	1.784248531

2 lowest-energy conformers used for Boltzmann-averaged ^{13}C NMR data.
Avg. Energy: -1147.963768 au.

V.59. 11-Methoxy-3,3-dimethyl-3H,7H-pyrano[2,3-c]xanthen-7-one (D30-3).[32]

No	type	δcal	δexp	diff
C1	CH	131.3 (-2.9)	129.7	1.6 (-2.9)
C2	CH	112.0	114.1	-2.1
C3	C	158.0	158.5	-0.5
C4	C	106.9	109.3	-2.4
C4a	C	152.9	152	0.9
C5	C	147.8	148.7	-0.9
C6	CH	115.1	115.1	0.0
C7	CH	122.5	122.5	0.0
C8	CH	120.6 (-2.9)	117.4	3.2 (-2.9)
C8a	C	125.1	123.3	1.8
C9	C	177.1	176.4	0.7
C9a	C	116.7	115.5	1.2
C10a	C	147.0	146.3	0.7
C1'	CH	118.1 (-3.7)	115.4	2.7 (-3.7)
C2'	CH	125.1 (+2.8)	127.1	-2.0 (+2.8)
C3'	C	79.8	77.9	1.9
C4'[2]	CH3	31.1	28.1	3.0
C1''	CH3	55.1	56.4	-1.3
RMSD		1.86		
Max Abs		3.25		
RMSD+CFx		1.54		
Max abs+CFx		3.00		

mol2 coordinates for lowest energy conformer

1	C1	-2.101291668	0.002348249	0.510151537
2	C2	-0.740497736	0.000130146	0.192813627
3	C3	-0.288112610	-0.000829574	-1.131688893
4	C4	-1.245442144	0.000966431	-2.147636437
5	C5	-2.619097657	0.003446193	-1.856195696
6	C6	-3.030136548	0.004133314	-0.542074418
7	C7	-2.541771185	0.003247947	1.910468574
8	C8	-1.437625491	0.002252158	2.896970797
9	C9	-0.118505736	0.000299450	2.456633295
10	O10	0.228074489	-0.001248144	1.143653838
11	C11	-1.708379314	0.003825166	4.273193532
12	C12	-0.667089915	0.003288786	5.175403718
13	C13	0.663611285	0.001376700	4.730006776
14	C14	0.953372847	-0.000129024	3.373698940
15	O15	-3.717904852	0.005989795	2.244156288
16	C16	1.121787000	-0.003606619	-1.502768257
17	C17	1.466562908	-0.004295902	-2.789201559
18	C18	0.464892545	-0.001982060	-3.915650378

19	O19	-0.909921048	0.000385054	-3.449107545
20	C20	0.617761314	1.261125150	-4.765270685
21	C21	0.612817338	-1.264988034	-4.766307278
22	O22	2.191469726	-0.001717818	2.827259836
23	C23	3.292819623	-0.002884954	3.707567399
24	H24	-3.325541983	0.004759973	-2.678590098
25	H25	-4.083485970	0.005972018	-0.281574700
26	H26	-2.746620671	0.005385093	4.585960573
27	H27	-0.865132336	0.004420114	6.242484944
28	H28	1.466386629	0.001139492	5.458587375
29	H29	1.865148700	-0.005191239	-0.713302675
30	H30	2.510286406	-0.006427610	-3.092035271
31	H31	-0.124303160	1.264545305	-5.569545605
32	H32	0.472502697	2.151313878	-4.146972672
33	H33	1.617309215	1.304361942	-5.210538744
34	H34	0.463859831	-2.155081976	-4.148754904
35	H35	1.612230784	-1.311908133	-5.211509177
36	H36	-0.129181935	-1.264727358	-5.570654542
37	H37	3.297722704	-0.897832141	4.343056645
38	H38	3.300444446	0.892855129	4.341980008
39	H39	4.180981475	-0.004716897	3.075331830

2 lowest-energy conformers used for Boltzmann-averaged ^{13}C NMR data.
Avg. Energy: -1034.580851 au.

V.60. 8,11-Dihydroxy-3,3-dimethyl-3H,7H-pyrano[2,3-c]xanthen-7-one (D30-4).[57]

No	type	δcal	δexp	diff
C1	C	155.7 (-2.9)	152.6	3.1 (-2.9)
C2	CH	111.3	109.2	2.1
C3	CH	123.1	123.7	-0.6
C4	C	135.0	137.2	-2.2
C4a	C	142.6	143.5	-0.9
C5	C	106.0	108.2	-2.2
C6	C	158.8	158.6	0.2
C7	CH	112.4	113.7	-1.3
C8	CH	131.3 (-2.9)	126.1	5.2 (-2.9)
C8a	C	115.2	114.6	0.6
C9	C	182.8	180.8	2.0
C9a	C	109.7	108.8	0.9
C10a	C	152.2	151.7	0.5
C1'	CH	116.5 (-3.7)	114.1	2.4 (-3.7)
C2'	CH	126.6 (+2.8)	130.6	-4.0 (+2.8)
C3'	C	80.0	78.3	1.7
C4'[2]	CH3	30.9	27.8	3.1
RMSD		2.40		
Max Abs		5.18		
RMSD+CFx		1.72		
Max abs+CFx		3.15		

mol2 coordinates for lowest energy conformer

1	C1	-0.567386794	0.000000000	-4.688527983
2	C2	0.623687462	0.000000000	-5.412696693
3	C3	1.848231907	0.000000000	-4.755115528
4	C4	1.925529895	0.000000000	-3.366808791
5	C5	0.005411247	0.000000000	0.892940225
6	C6	-1.106916232	0.000000000	1.740330666
7	C7	-2.414914523	0.000000000	1.230561811
8	C8	-2.608590795	0.000000000	-0.131930637
9	C9	-1.729290137	0.000000000	-2.466093465
10	C10	-0.227337050	0.000000000	-0.486399515
11	C11	1.332100062	0.000000000	1.497887971

12	C12	1.459884154	0.000000000	2.823476270
13	C13	0.285883247	0.000000000	3.767640113
14	C14	0.293830423	-1.262847244	4.630953874
15	C15	0.293830423	1.262847243	4.630953875
16	O16	-1.733379697	0.000000000	-5.347510617
17	O17	3.140208834	0.000000000	-2.752244565
18	O18	-0.990694111	0.000000000	3.077002403
19	O19	-2.860094893	0.000000000	-2.968649137
20	C20	0.735965011	0.000000000	-2.644887348
21	O21	0.874928377	0.000000000	-1.283137893
22	C22	-1.520578560	0.000000000	-1.018096056
23	C23	-0.515365353	0.000000000	-3.275415075
24	H24	2.775993175	0.000000000	-5.317771761
25	H25	0.573037594	0.000000000	-6.495532375
26	H26	-3.245060500	0.000000000	1.927509022
27	H27	-3.606223015	0.000000000	-0.557810921
28	H28	-2.448556775	0.000000000	-4.671066657
29	H29	2.991392686	0.000000000	-1.797888405
30	H30	2.439517950	0.000000000	3.293353241
31	H31	2.204024879	0.000000000	0.853793816
32	H32	1.202621711	1.303521369	5.240364958
33	H33	0.257252309	2.154212374	3.998411925
34	H34	-0.574408467	1.266310038	5.296813187
35	H35	1.202621711	-1.303521372	5.240364957
36	H36	0.257252310	-2.154212376	3.998411924
37	H37	-0.574408466	-1.266310041	5.296813185

2 lowest-energy conformers used for Boltzmann-averaged ^{13}C NMR data.
Avg. Energy: -1070.519461 au.

V.61. 8,11-Dihydroxy-3,3,10-trimethyl-3H,7H-pyrano[2,3-c]xanthen-7-one (D30-5).[57]

No	type	δcal	δexp	diff
C1	C	155.5 (-2.9)	152.4	3.1 (-2.9)
C2	CH	113.0	111	2.0
C3	C	134.8	135.9	-1.1
C4	C	133.4	134.7	-1.3
C4a	C	141.9	143.6	-1.7
C5	C	105.8	106.2	-0.4
C6	C	158.6	158.5	0.1
C7	CH	112.2	113.6	-1.4
C8	CH	131.3 (-2.9)	126.1	5.2 (-2.9)
C8a	C	115.2	115.2	0.0
C9	C	182.5	180.5	2.0
C9a	C	108.0	109	-1.0
C10	CH3	18.5	17	1.5
C10a	C	152.1	151.7	0.4
C1'	CH	116.6 (-3.7)	114.0	2.6 (-3.7)
C2'	CH	126.3 (+2.8)	130.4	-4.1 (+2.8)
C3'	C	80.0	78.1	1.9
C4'[2]	CH3	30.9	27.8	3.1
RMSD		2.32		
Max Abs		5.19		
RMSD+CFx		1.63		
Max abs+CFx		3.15		

mol2 coordinates for lowest energy conformer

1	C1	-1.214045205	0.000000000	-4.158267004
2	C2	-0.090397124	0.000000000	-4.981395515
3	C3	1.199292543	0.000000000	-4.453053084
4	C4	1.376926563	0.000000000	-3.065610424
5	C5	-0.149312597	-0.000000006	1.345027427

6	C6	-1.179068873	0.000000000	2.290767128
7	C7	-2.527671398	0.000000000	1.902880302
8	C8	-2.844041403	0.000000005	0.563080217
9	C9	-2.180707772	0.000000000	-1.844303376
10	C10	-0.506200128	0.000000000	-0.007378458
11	C11	1.227248108	0.000000000	1.826140650
12	C12	1.477177956	0.000000005	3.134128424
13	C13	0.395289895	0.000000000	4.182610141
14	C14	0.480533519	-1.263244194	5.041190158
15	C15	0.480533506	1.263244196	5.041190157
16	O16	-2.432104049	0.000000000	-4.714857365
17	O17	2.642770098	0.000000000	-2.557137133
18	O18	-0.939740344	0.000000000	3.611553883
19	O19	-3.352717741	0.000000000	-2.243845889
20	C20	0.260150257	0.000000000	-2.243079884
21	O21	0.520787441	0.000000000	-0.898777333
22	C22	-1.842074268	0.000000000	-0.419429300
23	C23	-1.044463653	0.000000000	-2.755541226
24	H24	-0.240669984	0.000000000	-6.055925241
25	H25	-3.290917490	0.000000000	2.672361307
26	H26	-3.876326837	0.000000000	0.229474409
27	H27	-3.085578181	0.000000000	-3.977542967
28	H28	2.575319620	0.000000000	-1.593630818
29	H29	2.496160302	0.000000000	3.511375602
30	H30	2.035551710	0.000000000	1.103783387
31	H31	1.441087608	1.305674923	5.565139997
32	H32	0.385151047	2.154113314	4.414132347
33	H33	-0.323516962	1.265802486	5.783280658
34	H34	1.441087621	-1.305674915	5.565140003
35	H35	0.385151053	-2.154113316	4.414132355
36	H36	-0.323516951	-1.265802497	5.783280653
37	C37	2.415165115	0.000000000	-5.337457054
38	H38	3.037549277	0.879190912	-5.140260605
39	H39	2.132588440	0.000000000	-6.392915925
40	H40	3.037549280	-0.879190915	-5.140260606

1 lowest-energy conformers used for Boltzmann-averaged ^{13}C NMR data.
Avg. Energy: -1109.840977 au.

V.62. 8,11-Dihydroxy-3,3,9-trimethyl-3H,7H-pyrano[2,3-c]xanthen-7-one (D30-6).[57]

No	type	δcal	δexp	diff
C1	C	153.8 (-2.9)	151.7	2.1 (-2.9)
C2	C	121.1	117.9	3.2
C3	CH	124.5	125.3	-0.8
C4	C	134.6	136.3	-1.7
C4a	C	140.6	141.6	-1.0
C5	C	105.9	107.6	-1.7
C6	C	158.7	158.5	0.2
C7	CH	112.1	113.6	-1.5
C8	CH	131.3 (-2.9)	126.2	5.1 (-2.9)
C8a	C	115.1	114.7	0.4
C9	C	183.0	180.9	2.1
C9a	C	109.3	108.8	0.5
C10a	C	152.3	150.2	2.1
C1'	CH	116.6 (-3.7)	113.9	2.7 (-3.7)
C2'	CH	126.3 (+2.8)	130.5	-4.2 (+2.8)
C3'	C	80.0	78.2	1.8
C4'[2]	CH3	31.0	27.8	3.2
C1''	CH3	17.2	14.4	2.8
RMSD		2.47		
Max Abs		5.12		

RMSD+CFx	1.90
Max abs+CFx	3.17

mol2 coordinates for lowest energy conformer

1	C1	-0.688171807	0.000000010	-4.111016188
2	C2	0.478770757	0.000000020	-4.886296568
3	C3	1.710127259	-0.000000029	-4.236535394
4	C4	1.824111824	-0.000000056	-2.849383154
5	C5	0.000415162	-0.000000066	1.455637285
6	C6	-1.094357142	-0.000000051	2.324968347
7	C7	-2.412493233	-0.000000068	1.842116287
8	C8	-2.633087830	-0.000000082	0.483950926
9	C9	-1.802449989	-0.000000031	-1.868046979
10	C10	-0.259419022	-0.000000073	0.080914661
11	C11	1.338987577	-0.000000043	2.034119012
12	C12	1.494016343	0.000000000	3.356700150
13	C13	0.339508638	0.000000017	4.324580691
14	C14	0.364322778	-1.263036452	5.187492125
15	C15	0.364322764	1.263036564	5.187492004
16	O16	-1.871703511	0.000000090	-4.742877354
17	O17	3.057296364	-0.000000087	-2.271732130
18	O18	-0.950648089	-0.000000007	3.659361492
19	O19	-2.944808209	-0.000000060	-2.346045012
20	C20	0.657939628	-0.000000049	-2.096071482
21	O21	0.826107115	-0.000000068	-0.736255779
22	C22	-1.563316472	-0.000000067	-0.424606567
23	C23	-0.605732617	-0.000000025	-2.702277110
24	H24	-3.228365408	-0.000000064	2.555581045
25	H25	-3.639109781	-0.000000075	0.078374811
26	H26	-2.569677309	0.000000135	-4.046986101
27	H27	2.937192658	-0.000000110	-1.313335705
28	H28	2.483002529	0.000000036	3.806432904
29	H29	2.197341164	-0.000000038	1.372108537
30	H30	1.284559765	1.303216037	5.779577332
31	H31	0.316319243	2.154310097	4.555470117
32	H32	-0.491310865	1.267030518	5.869587623
33	H33	1.284559765	-1.303215900	5.779577485
34	H34	0.316319195	-2.154310016	4.555470306
35	H35	-0.491310886	-1.267030393	5.869587711
36	H36	2.628115616	-0.000000029	-4.817073344
37	C37	0.364852257	0.000000079	-6.385637459
38	H38	-0.187305097	0.878734366	-6.735141523
39	H39	-0.187305150	-0.878734131	-6.735141591
40	H40	1.352384017	0.000000115	-6.854641410

2 lowest-energy conformers used for Boltzmann-averaged ^{13}C NMR data.
Avg. Energy: -1109.839691 au.

V.63. 6-Hydroxy-3,3-dimethyl-3H,7H-pyrano[2,3-c]xanthen-7-one (D31-1).[58]

C	Type	Theory	Expt.	diff.
C1	C	165.6 (-2.9)	163.2	2.4 (-2.9)
C2	CH	98.3	99.4	-1.1
C3	C	160.9	161.0	-0.1
C4	C	98.2	101.1	-2.9
C4a	C	153.1	151.8	1.3
C5	CH	116.6	117.6	-1.0
C6	CH	133.7	135.0	-1.3
C7	CH	122.5	124.1	-1.6
C8	CH	129.0 (-2.9)	125.9	3.1 (-2.9)
C8a	C	122.3	120.6	1.7
C9	C	182.1	180.9	1.2

C9a	C	104.7	103.8	0.9
C10a	C	155.3	155.8	-0.5
C1'	CH	117.4 (-3.7)	115.0	2.4 (-3.7)
C2'	CH	122.8 (+2.8)	127.2	-4.4 (+2.8)
C3'	C	79.8	78.3	1.5
C4'2	CH3	30.9	28.3	2.6
RMSD		2.09		
Max Abs		4.43		
RMSD+CFx		1.53		
Max abs+CFx		2.87		

mol2 coordinates for lowest energy conformer

1	C1	-0.092008257	0.000000000	0.619038550
2	C2	-1.381623102	0.000000000	1.179069594
3	C3	-1.565752513	0.000000000	2.614275894
4	C4	-0.327453932	0.000000000	3.406680575
5	C5	0.908100760	0.000000000	2.757228836
6	O6	1.019672760	0.000000000	1.402513094
7	C7	0.128873692	0.000000000	-0.753624101
8	C8	-1.003471796	0.000000000	-1.588114127
9	C9	-2.300277478	0.000000000	-1.079484700
10	C10	-2.496279352	0.000000000	0.294287121
11	C11	1.446574509	0.000000000	-1.371808373
12	C12	1.561485314	0.000000000	-2.699112015
13	C13	0.375231249	0.000000000	-3.627344642
14	O14	-0.895036171	0.000000000	-2.923993309
15	C15	0.369348935	-1.262447519	-4.491229122
16	C16	0.369348936	1.262447520	-4.491229123
17	O17	-3.741503784	0.000000000	0.767631778
18	H18	-3.150263242	0.000000000	-1.750645279
19	H19	2.321734257	0.000000000	-0.731482468
20	H20	2.535395016	0.000000000	-3.181002116
21	H21	-0.507551375	-1.264903498	-5.145649448
22	H22	1.270396442	-1.304521107	-5.112103294
23	H23	0.340192626	-2.153209663	-3.857612388
24	H24	-0.507551374	1.264903499	-5.145649449
25	H25	1.270396444	1.304521103	-5.112103295
26	H26	0.340192627	2.153209663	-3.857612388
27	O27	-2.682509680	0.000000000	3.152423944
28	C28	-0.359449900	0.000000000	4.806674396
29	H29	-1.331307737	0.000000000	5.289354701
30	C30	2.100664250	0.000000000	3.480905065
31	C31	0.815689410	0.000000000	5.534421351
32	H32	0.788089406	0.000000000	6.619144303
33	H33	3.043781878	0.000000000	2.945105075
34	C34	2.045475889	0.000000000	4.864372808
35	H35	2.970664926	0.000000000	5.432783120
36	H36	-3.679269633	0.000000000	1.753889431

1 lowest-energy conformers used for Boltzmann-averaged ^{13}C NMR data.
Avg. Energy: -995.298591 au.

V.64. 6-Methoxy-3,3-dimethyl-3H,7H-pyrano[2,3-c]xanthen-7-one (D31-2).[32]

No	type	δcal	δexp	diff
C1	C	162.7 (-2.9)	161.7	1.0 (-2.9)
C2	CH	95.2	95.6	-0.4
C3	C	158.7	158.8	-0.1
C4	C	100.3	102.2	-1.9
C4a	C	155.2	154.6	0.6
C5	CH	116.0	116.8	-0.8
C6	CH	132.2	133.6	-1.4

C7	CH	122.3	122.8	-0.5
C8	CH	129.8 (-2.9)	127	2.8 (-2.9)
C8a	C	124.4	123.8	0.6
C9	C	175.0	175.4	-0.4
C9a	C	109.1	106.9	2.2
C10a	C	154.2	153.7	0.5
C1'	CH	118.3 (-3.7)	115.4	2.9 (-3.7)
C2'	CH	122.6 (+2.8)	126.7	-4.1 (+2.8)
C3'	C	79.7	78.1	1.6
C4'[2]	CH3	30.7	28.2	2.5
C1''	CH3	55.4	56.3	-0.9
RMSD		1.82		
Max Abs		4.13		
RMSD+CFx		1.36		
Max abs+CFx		2.54		

mol2 coordinates for lowest energy conformer

1	C1	1.045051051	0.000749412	1.219138476
2	C2	-0.259823373	0.000218148	0.699864182
3	C3	-0.544133832	0.000925240	-0.670192419
4	C4	0.538172802	0.001652612	-1.547984198
5	C5	1.856632583	0.002287320	-1.088572814
6	C6	2.113199618	0.001841596	0.276601482
7	C7	1.255901692	0.000475285	2.678351870
8	C8	0.001352627	-0.001251688	3.474097744
9	C9	-1.241264434	-0.001063066	2.849468634
10	O10	-1.363094993	-0.000368769	1.494172862
11	C11	0.049928236	-0.002159469	4.872846506
12	C12	-1.114939517	-0.002689574	5.618599770
13	C13	-2.354766604	-0.002281402	4.967405542
14	C14	-2.426904799	-0.001533874	3.583976063
15	O15	2.342910250	0.001692536	3.234264281
16	O16	3.354892913	0.004756879	0.779184573
17	C17	4.448978538	-0.006247256	-0.111628832
18	C18	-1.888870207	0.001659406	-1.229941047
19	C19	-2.060656919	0.002760036	-2.551098620
20	C20	-0.915963935	0.002627321	-3.531390765
21	O21	0.383054026	0.002469788	-2.881993403
22	C22	-0.947032999	1.265220755	-4.395216578
23	C23	-0.947786920	-1.259938058	-4.395260413
24	H24	2.646784319	0.003934892	-1.826664432
25	H25	1.029504665	-0.002286692	5.339127701
26	H26	-1.070462057	-0.003174625	6.702640836
27	H27	-3.272113498	-0.002480855	5.548241271
28	H28	-3.375779624	-0.001230594	3.058878632
29	H29	5.337790288	-0.010387125	0.519347940
30	H30	4.455801392	0.886984836	-0.748707174
31	H31	4.444600760	-0.904507134	-0.740788637
32	H32	-2.735172333	0.001823391	-0.552748631
33	H33	-3.054156937	0.003708315	-2.990375013
34	H34	-0.099126183	1.268867804	-5.087156138
35	H35	-0.891222164	2.155964684	-3.763182933
36	H36	-1.874002912	1.307064366	-4.976440455
37	H37	-0.099704740	-1.264265300	-5.086994059
38	H38	-1.874694755	-1.301091290	-4.976697839
39	H39	-0.892882026	-2.150727849	-3.763173966

2 lowest-energy conformers used for Boltzmann-averaged ^{13}C NMR data.
Avg. Energy: -1034.578910 au.

V.65. Nigrolineaxanthone F (D31-3).[34]

No	type	δcal	δexp	diff
C1	C	165.5 (-2.9)	162.4	3.1 (-2.9)
C2	CH	98.0	98.4	-0.4
C3	C	161.0	160.2	0.8
C4	C	98.0	103	-5.0
C4a	C	153.3	154.4	-1.1
C5	CH	119.2	119.4	-0.2
C6	CH	121.8	125	-3.2
C7	C	151.2	151.6	-0.4
C8	CH	110.6 (-2.9)	108.1	2.5 (-2.9)
C8a	C	123.2	120.5	2.7
C9	C	181.8	180.4	1.4
C9a	C	104.5	103	1.5
C10a	C	149.1	149.2	-0.1
C1'	CH	117.4 (-3.7)	114.5	2.9 (-3.7)
C2'	CH	122.5 (+2.8)	127.8	-5.3 (+2.8)
C3'	C	79.8	78.6	1.2
C4'[2]	CH3	30.9	28.1	2.8
RMSD		2.56		
Max Abs		5.29		
RMSD+CFx		2.01		
Max abs+CFx		4.95		

mol2 coordinates for lowest energy conformer

1	C1	0.516813359	1.262531039	-4.683243198
2	C2	0.516813358	-1.262531039	-4.683243198
3	O3	-2.769559685	0.000000000	2.865635951
4	O4	0.630112761	0.000000000	6.715027727
5	O5	-3.752804016	0.000000000	0.447001451
6	C6	1.498907573	0.000000000	-1.532359808
7	C7	1.654267916	0.000000000	-2.855488007
8	C8	1.894248302	0.000000000	4.729157194
9	C9	1.991073291	0.000000000	3.351800257
10	C10	-2.255267113	0.000000000	-1.354042947
11	C11	-0.513024976	0.000000000	4.588061491
12	O12	-0.793907887	0.000000000	-3.154493616
13	O13	0.985730072	0.000000000	1.227706870
14	C14	0.637374491	0.000000000	5.356338110
15	C15	-2.493563141	0.000000000	0.012403352
16	C16	-0.942710414	0.000000000	-1.822357361
17	C17	0.162992222	0.000000000	-0.954379043
18	C18	-1.636826622	0.000000000	2.359428074
19	C19	0.827089803	0.000000000	2.580512185
20	C20	-0.424204505	0.000000000	3.190611189
21	C21	-0.099004599	0.000000000	0.412101052
22	C22	-1.406390917	0.000000000	0.932125238
23	C23	0.496815746	0.000000000	-3.819361100
24	H24	-0.340376236	1.265957826	-5.363446238
25	H25	0.469780014	2.153497007	-4.050864836
26	H26	1.435936234	1.303763664	-5.277099156
27	H27	0.469780014	-2.153497007	-4.050864836
28	H28	1.435936234	-1.303763664	-5.277099156
29	H29	-0.340376236	-1.265957826	-5.363446238
30	H30	-0.282595040	0.000000000	7.027711687
31	H31	-3.721313409	0.000000000	1.434488671
32	H32	2.354170911	0.000000000	-0.865772551
33	H33	2.642260372	0.000000000	-3.307452177
34	H34	2.784732242	0.000000000	5.348653645
35	H35	2.955500077	0.000000000	2.855764088

36	H36	-3.083430739	0.000000000	-2.051732928
37	H37	-1.504979458	0.000000000	5.032218163

2 lowest-energy conformers used for Boltzmann-averaged ^{13}C NMR data.
Avg. Energy: -1070.524510 au.

V.66. 6-Deoxyi-5-O-methylsojacareubin (D31-4).[32]

No	type	δcal	δexp	diff
C1	C	165.4 (-2.9)	162.9	2.5 (-2.9)
C2	CH	98.1	99.2	-1.1
C3	C	160.9	160.8	0.1
C4	C	98.5	101.3	-2.8
C4a	C	153.2	151.5	1.7
C5	C	147.7	148.4	-0.7
C6	CH	115.6	115.1	0.5
C7	CH	122.8	123.5	-0.7
C8	CH	119.8 (-2.9)	116.5	3.3 (-2.9)
C8a	C	123.7	121.2	2.5
C9	C	182.3	180.7	1.6
C9a	C	104.7	103.6	1.1
C10a	C	146.9	146	0.9
C1'	CH	117.8 (-3.7)	115.6	2.2 (-3.7)
C2'	CH	122.3 (+2.8)	127	-4.7 (+2.8)
C3'	C	79.9	78.2	1.7
C4'[2]	CH3	30.9	28.2	2.7
C1''	C	55.2	56.3	-1.1
RMSD		2.14		
Max Abs		4.70		
RMSD+CFx		1.61		
Max abs+CFx		2.77		

mol2 coordinates for lowest energy conformer

1	C1	-1.983942595	-0.002462407	-0.474119503
2	C2	-0.614485091	-0.000834536	-0.160611732
3	C3	-0.142960832	-0.000455071	1.147227443
4	C4	-1.100568502	-0.002130443	2.175891382
5	C5	-2.469776634	-0.003767412	1.915584221
6	C6	-2.916304024	-0.003778432	0.601648712
7	C7	-2.426348656	-0.002499145	-1.851232730
8	C8	-1.354742410	-0.001944539	-2.860198680
9	C9	-0.029720420	0.000200212	-2.438995460
10	O10	0.332530577	0.000802777	-1.132557466
11	C11	-1.649633710	-0.002969931	-4.232077097
12	C12	-0.621549269	-0.001690858	-5.148598801
13	C13	0.716498107	0.000870525	-4.723348034
14	C14	1.028322158	0.001786320	-3.372136368
15	O15	-3.623080562	-0.003301867	-2.177041904
16	O16	-4.227256962	-0.005113404	0.365011725
17	C17	1.267808879	0.001520261	1.505633984
18	C18	1.627398025	0.002505337	2.788211687
19	C19	0.635285380	0.001368751	3.922147728
20	O20	-0.745030121	-0.002494013	3.469191933
21	C21	0.786923407	1.265675381	4.770350436
22	C22	0.793021263	-1.260467644	4.772877712
23	O23	2.272795705	0.004901166	-2.843919073
24	C24	3.362778034	0.001694024	-3.739589634
25	H25	-3.179755994	-0.004957866	2.733041962
26	H26	-2.691307937	-0.004611263	-4.531799094
27	H27	-0.835866143	-0.002431436	-6.212240423
28	H28	1.506964829	0.002402379	-5.464863156
29	H29	-4.345266319	-0.004213253	-0.616724087

30	H30	2.004109388	0.002326791	0.709384092
31	H31	2.673571802	0.004213836	3.081021202
32	H32	0.052197158	1.265758143	5.581354186
33	H33	0.631085657	2.154510706	4.152561760
34	H34	1.790321556	1.314886276	5.206050620
35	H35	1.796541204	-1.303736225	5.208922448
36	H36	0.058088335	-1.262664823	5.583732664
37	H37	0.641847008	-2.151252711	4.156801233
38	H38	3.360820239	0.895785619	-4.376284130
39	H39	4.259186458	0.001946576	-3.118765920
40	H40	3.359501012	-0.895377802	-4.371543840

2 lowest-energy conformers used for Boltzmann-averaged ^{13}C NMR data.
Avg. Energy: -1109.822410 au.

V.67. Isojacareubin (D31-5).[59]

No	type	δcal	δexp	diff
C1	C	165.9 (-2.9)	164.2	1.7 (-2.9)
C2	CH	100.0	99.4	0.6
C3	C	161.0	161.2	-0.2
C4	C	100.3	101.8	-1.5
C4a	C	152.3	153	-0.7
C5	C	130.0	133.5	-3.5
C6	C	149.6	152.7	-3.1
C7	CH	112.2	113.9	-1.7
C8	CH	121.6 (-2.9)	117.5	4.1 (-2.9)
C8a	C	115.1	114.6	0.5
C9	C	182.0	181.4	0.6
C9a	C	104.6	103.5	1.1
C10a	C	143.9	147	-3.1
C1'	CH	118.1 (-3.7)	115.9	2.2 (-3.7)
C2'	CH	125.2 (+2.8)	128.0	-2.8 (+2.8)
C3'	C	78.1	78.9	-0.8
C4'[2]	CH3	28.0	28.3	-0.3
RMSD		2.01		
Max Abs		4.08		
RMSD+CFx		1.58		
Max abs+CFx		3.49		

mol2 coordinates for lowest energy conformer

1	C1	-2.659043814	-0.108540902	0.381595258
2	C2	-2.237258140	-0.253218161	1.698589714
3	C3	-0.878468674	-0.341495682	1.975318995
4	C4	0.100428177	-0.306405650	0.962215653
5	C5	1.316364312	0.031993133	-3.520917287
6	C6	1.049209005	0.174555035	-4.881561296
7	C7	-0.270813009	0.299613995	-5.329773951
8	C8	-1.311446336	0.281796609	-4.421182500
9	C9	-2.137312244	0.108258801	-2.057358145
10	C10	0.258871795	0.015429502	-2.619323273
11	C11	1.491268258	-0.510318205	1.344974715
12	C12	1.830702148	-0.465414672	2.633846447
13	C13	0.804571302	-0.122315475	3.687799783
14	C14	1.045216165	-0.904376343	4.973617813
15	C15	0.780323408	1.387294159	3.946326668
16	C16	-0.348561676	-0.157785685	-0.344785390
17	C17	-1.062778539	0.139175178	-3.050308145
18	C18	-1.709000696	-0.050199488	-0.675949436
19	O19	-3.962880606	-0.012704256	0.134397138
20	O20	-0.521902141	-0.521540566	3.260098813
21	O21	-3.330693621	0.206407162	-2.378861421

22	O22	0.614915131	-0.126336660	-1.310667930
23	H23	-2.341881672	0.375212579	-4.745115090
24	H24	-0.446432049	0.408659149	-6.393911460
25	H25	2.222614763	-0.732552904	0.575214620
26	H26	2.850552500	-0.639656271	2.963876330
27	H27	0.548035135	1.928089728	3.023515399
28	H28	1.757328926	1.726624804	4.305412779
29	H29	0.023281503	1.629227480	4.699002039
30	H30	2.013456318	-0.630167263	5.403966496
31	H31	1.040399393	-1.979006677	4.771911071
32	H32	0.262508896	-0.680015939	5.703925472
33	H33	-2.962800837	-0.288922081	2.501502170
34	H34	-4.067668158	0.094921048	-0.843716744
35	O35	2.621897248	-0.085525897	-3.143230167
36	H36	2.657780305	-0.165198085	-2.180789118
37	O37	2.064996262	0.193885006	-5.768786486
38	H38	2.894221264	0.080553494	-5.280869536

2 lowest-energy conformers used for Boltzmann-averaged ^{13}C NMR data.
Avg. Energy: -1145.755869 au.

V.68. di-O-Methyllorostemin (D31-6).[32]

No	type	δcal	δexp	diff
C1	C	163.0 (-2.9)	161.5	1.5 (-2.9)
C2	CH	98.9	95.5	3.4
C3	C	158.4	158.1	0.3
C4	C	102.8	102.1	0.7
C4a	C	155.3	153.7	1.6
C5	CH	101.7	98.9	2.8
C6	C	153.8	154.2	-0.4
C7	C	146.2	146.4	-0.2
C8	CH	111.2 (-2.9)	105.7	5.5 (-2.9)
C8a	C	118.2	115.4	2.8
C9	C	174.7	174.6	0.1
C9a	C	110.4	106.5	3.9
C10a	C	149.8	150.5	-0.7
C1'	CH	119.5 (-3.7)	115.8	3.7 (-3.7)
C2'	CH	123.7 (+2.8)	126.9	-3.2 (+2.8)
C3'	C	78.5	77.9	0.6
C4'[2]	CH3	29.0	28.2	0.8
C1''	CH3	56.9	56.3	0.6
C1'''	CH3	55.3	56.2	-0.9
C1''''	CH3	55.1	56.2	-1.1
RMSD		2.26		
Max Abs		5.50		
RMSD+CFx		1.68		
Max abs+CFx		3.88		

mol2 coordinates for lowest energy conformer

1	C1	-0.449596283	0.176156202	-1.369066809
2	C2	-0.432009077	-0.001079107	0.023527350
3	C3	-1.590618926	-0.079261380	0.805738966
4	C4	-2.814619102	0.027868991	0.150136242
5	C5	-2.892837929	0.206926328	-1.232406068
6	C6	-1.728856566	0.280714297	-1.986935757
7	C7	0.820670270	0.246073914	-2.118663636
8	C8	2.025363435	0.124534158	-1.270987824
9	C9	1.924014544	-0.046693936	0.095111351
10	O10	0.721762341	-0.111567298	0.731101624
11	C11	3.305892710	0.180800133	-1.851214769
12	C12	4.441185210	0.068646702	-1.080502467

13	C13	4.312662489	-0.107718919	0.327920227
14	C14	3.057100510	-0.164082541	0.907399207
15	O15	0.918211930	0.391747455	-3.328259888
16	O16	-1.739212631	0.453780688	-3.316037554
17	C17	-1.593146618	-0.264145070	2.250188607
18	C18	-2.747730239	-0.325972511	2.912096071
19	C19	-4.091341453	-0.213155576	2.238199666
20	O20	-3.989056503	-0.033266571	0.801101049
21	C21	-4.900732600	-1.495967108	2.439764627
22	C22	-4.851759385	1.009288135	2.756358276
23	C23	-2.980320881	0.552536735	-3.978565193
24	O24	5.714067870	0.109485091	-1.540440095
25	O25	5.474139078	-0.209483480	1.003246008
26	C26	5.411123142	-0.394653681	2.399769936
27	C27	5.888571231	0.273837799	-2.929664374
28	H28	-3.876252196	0.284520841	-1.675512478
29	H29	3.344567040	0.315349603	-2.925629305
30	H30	2.919073792	-0.296551362	1.972850352
31	H31	-0.643660306	-0.349436622	2.766076721
32	H32	-2.773847925	-0.463403680	3.989532875
33	H33	-5.868546661	-1.414524594	1.934989974
34	H34	-4.358494344	-2.351722257	2.027956171
35	H35	-5.075693745	-1.673618767	3.506038526
36	H36	-4.274564504	1.918970544	2.568175591
37	H37	-5.025463230	0.922560002	3.834027867
38	H38	-5.819071995	1.093627883	2.251300599
39	H39	-3.578118859	-0.357773395	-3.848347101
40	H40	-3.554429461	1.419662364	-3.629017993
41	H41	-2.737837442	0.679036309	-5.033701291
42	H42	4.886125505	-1.323377969	2.656903209
43	H43	4.916423968	0.451152988	2.894134483
44	H44	6.445228536	-0.457692047	2.739384809
45	H45	5.462835331	1.223510650	-3.278566662
46	H46	6.966198091	0.275696358	-3.097870904
47	H47	5.432601838	-0.551336303	-3.491640214

7 lowest-energy conformers used for Boltzmann-averaged ^{13}C NMR data.
Avg. Energy: -1263.631757 au.

V.69. 6,10,11-Trihydroxy-8-(3-hydroxy-3-methylbutyl)-3,3-dimethyl-9-(3-methyl-2-buten-1-yl)-3H,7H-pyrano[2,3-c] xanthen-7-one (D31-11).[52]

No	type	δcal	δexp	diff
C1	C	166.2 (-2.9)	163.8	2.4 (-2.9)
C2	CH	100.1	99	1.1
C3	C	161.2	160.3	0.9
C4	C	100.0	104	-4.0
C4a	C	151.6	150.9	0.7
C5	C	128.2	130	-1.8
C6	C	147.8	151.3	-3.5
C7	C	124.7	125.6	-0.9
C8	C	143.0 (-2.9)	136.7	6.3 (-2.9)
C8a	C	113.1	111.6	1.5
C9	C	184.6	183.1	1.5
C9a	C	105.0	101.1	3.9
C10a	C	144.2	146.8	-2.6
C1'	CH	118.4 (-3.7)	115.9	2.5 (-3.7)
C2'	CH	125.2 (+2.8)	127.2	-2.0 (+2.8)
C3'	C	78.0	78.3	-0.3
C4'[2]	CH3	27.6	28	-0.4
C1''	CH2	26.0	25.1	0.9
C2''	CH	126.5	123.7	2.8

C3''	C	131.5	131.4	0.1
C4''	CH3	18.1	17.9	0.2
C5''	CH3	25.7	25.6	0.1
C1'''	CH2	26.1	24.8	1.3
C2'''	CH2	43.9	45.3	-1.4
C3'''	C	68.8	69.9	-1.1
C4'''[2]	CH3	30.2	28.9	1.3
RMSD		2.18		
Max Abs		6.26		
RMSD+CFx		1.82		
Max abs+CFx		3.98		

mol2 coordinates for lowest energy conformer

1	O1	1.805971837	0.387832091	-0.953387429
2	C2	2.333589792	-4.353750529	0.543965126
3	C3	-2.563406976	0.685397662	1.685526896
4	C4	3.115683874	-3.556683942	-0.282514375
5	C5	-1.975379215	1.642344435	0.845049053
6	C6	1.155798502	-3.875073724	1.148733696
7	C7	-1.988650057	-0.565777731	1.857791711
8	C8	2.726872941	-2.248564779	-0.547199501
9	C9	-0.805042479	1.326792848	0.148310954
10	O10	-0.346892034	-2.117872559	1.452684431
11	C11	1.093070511	-0.358846078	-0.253782738
12	C12	0.786426584	-2.567522501	0.857103850
13	C13	-0.807965982	-0.859942839	1.197626697
14	C14	1.523920516	-1.724349152	0.014275687
15	C15	-0.179415339	0.064066336	0.348066879
16	O16	3.494988542	-1.515275545	-1.347831770
17	O17	-3.713827684	0.975998598	2.332253757
18	O18	-2.624363828	-1.446138096	2.685298228
19	C19	-0.222748029	2.347108687	-0.801108889
20	C20	0.872176209	3.195014232	-0.136462300
21	C21	-2.675762825	2.982175489	0.710203435
22	C22	1.766094315	3.949767271	-1.137463125
23	C23	0.978762996	4.998509584	-1.921182729
24	C24	2.926290571	4.611173568	-0.384711073
25	C25	-2.789318644	5.134158210	-1.563364320
26	C26	-3.669102314	3.916126714	-1.454932343
27	C27	-3.609678920	3.010578234	-0.473046465
28	C28	-4.677762383	3.778637823	-2.566948255
29	O29	2.287921102	3.047685419	-2.105713495
30	C30	0.470646346	-4.742858851	2.097392756
31	C31	0.807534658	-6.031551060	2.167707343
32	C32	1.822279243	-6.612247055	1.211269056
33	O33	2.773522467	-5.598238166	0.797877805
34	C34	1.133476782	-7.149602678	-0.046644900
35	C35	2.672850473	-7.683284879	1.882971443
36	H36	4.022714931	-3.949929975	-0.724094444
37	H37	3.069407646	-0.626372012	-1.415831489
38	H38	-3.962522720	0.202588644	2.859976575
39	H39	-2.139232652	-2.281765615	2.671968641
40	H40	-1.025660032	2.985167932	-1.174679642
41	H41	0.202334705	1.846294485	-1.670266085
42	H42	1.515360504	2.537884346	0.462446343
43	H43	0.425478740	3.910364295	0.566804229
44	H44	-1.936207141	3.783701022	0.658624178
45	H45	-3.255325502	3.156282500	1.623120885
46	H46	0.163633523	4.530344642	-2.480390953
47	H47	0.559742202	5.758976106	-1.252855235

48	H48	1.638136861	5.492905240	-2.641035498
49	H49	3.520457362	3.854397520	0.139576167
50	H50	3.582004241	5.127462873	-1.092346467
51	H51	2.566301890	5.337649458	0.353359001
52	H52	-2.023821434	5.193461123	-0.786265387
53	H53	-2.279961945	5.154695196	-2.534990921
54	H54	-3.395060156	6.047724678	-1.506009436
55	H55	-4.310384517	2.176090131	-0.504377696
56	H56	-5.304013595	2.890619508	-2.442321253
57	H57	-4.176521822	3.710003107	-3.541193593
58	H58	-5.334216125	4.657441396	-2.610801913
59	H59	2.372175029	2.179012381	-1.686191962
60	H60	-0.271985548	-4.315240112	2.762879476
61	H61	0.346973742	-6.705860312	2.883683253
62	H62	1.876384041	-7.555200130	-0.740861694
63	H63	0.579238192	-6.350218785	-0.548135936
64	H64	0.426728568	-7.942667282	0.217869897
65	H65	3.432569427	-8.052091211	1.188075297
66	H66	3.172372017	-7.274891624	2.765483493
67	H67	2.044338014	-8.524616559	2.190967074

21 lowest-energy conformers used for Boltzmann-averaged ^{13}C NMR data.
Avg. Energy: -1612.916353 au.

V.70. Cudratricusxanthone H (D31-12).[60]

No	type	δcal	δexp	diff
C1	C	165.3 (-2.9)	163.8	1.5 (-2.9)
C2	C	117.6	117.6	0.0
C3	C	160.7	160.3	0.4
C4	C	101.5	102.5	-1.0
C4a	C	151.1	151.6	-0.5
C5	C	129.9	133.4	-3.5
C6	C	149.6	153	-3.4
C7	CH	112.2	114.1	-1.9
C8	CH	121.7 (-2.9)	118.2	3.5 (-2.9)
C8a	C	115.0	115.1	-0.1
C9	C	182.6	182.1	0.5
C9a	C	104.5	103.6	0.9
C10a	C	143.7	147.1	-3.4
C1'	C	41.9	42	-0.1
C2'	CH	152.2	151.8	0.4
C3'	CH2	109.0	108	1.0
C4'[2]	CH3	29.3	29.9	-0.6
C1''	CH	118.3 (-3.7)	116.8	1.5 (-3.7)
C2''	CH	125.6 (+2.8)	127.8	-2.2 (+2.8)
C3''	C	78.0	79.4	-1.4
C4''[2]	CH3	27.5	28.3	-0.8
RMSD		1.73		
Max Abs		3.54		
RMSD+CFx		1.54		
Max abs+CFx		3.54		

mol2 coordinates for lowest energy conformer

1	C1	-0.437330682	-4.712713587	0.310232513
2	C2	2.268910193	-2.339474379	1.706626792
3	C3	3.229211968	-3.134393158	-0.487422103
4	C4	-3.035709284	-2.769026062	-0.489033614
5	C5	-1.114253570	-3.042144399	-2.048235450
6	O6	-3.346153539	2.429946606	-0.200056251
7	O7	0.610201250	7.298981039	0.840035145
8	O8	2.012561401	4.996376849	0.666053433

9	O9	-3.125166135	-0.089863859	-0.532134058
10	C10	-0.925810141	-3.485601892	0.456352505
11	C11	2.431382284	0.428389404	-0.047042609
12	C12	3.162867923	-0.681157869	0.058469951
13	C13	-1.442656100	6.151626771	0.485962060
14	C14	-2.121532152	4.969683210	0.262496707
15	O15	1.189844756	-1.988675341	-0.426462975
16	O16	0.723906954	2.640808768	0.222484188
17	C17	-0.048755787	6.143015399	0.620191645
18	C18	0.656436221	4.944444209	0.527317701
19	C19	-1.807988746	0.006965794	-0.365753556
20	C20	0.976259484	0.338401586	-0.072583703
21	C21	0.383199993	-0.922080480	-0.280492840
22	C22	-1.000791136	-1.143236142	-0.409702047
23	C23	-2.112684864	2.482985842	-0.068286604
24	C24	-1.429762284	3.755306734	0.166574259
25	C25	-0.041412838	3.764533308	0.299020135
26	C26	0.129956822	1.431676569	0.008368397
27	C27	-1.256954103	1.305852079	-0.134452135
28	C28	2.479153734	-2.016099817	0.224777301
29	C29	-1.504941838	-2.587957547	-0.630367275
30	H30	-0.132562103	-5.298872289	1.172732175
31	H31	-0.316925073	-5.179962336	-0.662446285
32	H32	1.658039775	-1.565978844	2.183793518
33	H33	3.233822498	-2.378140016	2.222960598
34	H34	1.764903138	-3.304656899	1.812850681
35	H35	2.664006675	-4.067712955	-0.407087513
36	H36	3.359763332	-2.891590248	-1.545794411
37	H37	4.215223327	-3.277286071	-0.034175281
38	H38	-3.406428393	-2.412566092	0.475516731
39	H39	-3.248589044	-3.840299712	-0.563217684
40	H40	-3.581774722	-2.245795250	-1.274399419
41	H41	-0.030471282	-3.064163996	-2.180377506
42	H42	-1.544085835	-2.354940944	-2.783734374
43	H43	-1.513193844	-4.041105819	-2.254919285
44	H44	1.556528422	7.100635032	0.901526050
45	H45	2.361254590	4.096319187	0.625170984
46	H46	-3.506694083	0.823750240	-0.441782216
47	H47	-1.016580247	-3.073508270	1.462225132
48	H48	2.895410679	1.403803181	-0.149711575
49	H49	4.248756312	-0.657191100	0.057587104
50	H50	-1.962016528	7.100266715	0.563413129
51	H51	-3.200377380	4.952426853	0.156931935

4 lowest-energy conformers used for Boltzmann-averaged ^{13}C NMR data.
Avg. Energy: -1341.091179 au.

V.71. Nigrolineaxanthone H (D32-1).[42]

No	type	δcal	δexp	diff
C1	CH	113.4 (-2.9)	108.7	4.7 (-2.9)
C2	C	141.2	142.1	-0.9
C3	C	145.3	146.5	-1.2
C4	C	107.1	109.2	-2.1
C4a	C	146.1	146.9	-0.8
C5	CH	104.3	106.7	-2.4
C6	CH	136.0	135.9	0.1
C7	CH	110.2	110.3	-0.1
C8	C	164.1 (-2.9)	161.8	2.3 (-2.9)
C8a	C	109.7	108.5	1.2
C9	C	183.1	181.3	1.8
C9a	C	116.1	114.1	2.0

C10a	C	156.3	156.1	0.2
C1'	CH	117.9 (-3.7)	115.4	2.5 (-3.7)
C2'	CH	125.5 (+2.8)	129.9	-4.4 (+2.8)
C3'	C	80.9	79.5	1.4
C4'[2]	CH3	30.9	28.3	2.6
RMSD		2.24		
Max Abs		4.69		
RMSD+CFx		1.58		
Max abs+CFx		2.56		

mol2 coordinates for lowest energy conformer

1	C1	0.440996525	-1.263711329	4.504760724
2	C2	0.440996527	1.263711331	4.504760722
3	O3	-2.658850325	0.000000000	-3.109356292
4	O4	-3.285844361	0.000000000	1.945540256
5	O5	-1.529239257	0.000000000	-5.460125179
6	C6	1.506713580	0.000000000	1.375197514
7	C7	1.618330589	0.000000000	2.702731143
8	C8	2.042559474	0.000000000	-4.890490652
9	C9	0.820133056	0.000000000	-5.551848940
10	C10	2.129275455	0.000000000	-3.502153684
11	C11	-2.432352843	0.000000000	-0.275197188
12	O12	-0.833093920	0.000000000	2.937845282
13	O13	1.070325060	0.000000000	-1.417367246
14	C14	-2.238382602	0.000000000	1.086874275
15	C15	-0.362861451	0.000000000	-4.813839888
16	C16	0.186782538	0.000000000	0.753830206
17	C17	-0.920700771	0.000000000	1.589687182
18	C18	-1.529783887	0.000000000	-2.598122115
19	C19	0.948439923	0.000000000	-2.772036599
20	C20	-1.332104850	0.000000000	-1.149529033
21	C21	-0.038246198	0.000000000	-0.629932315
22	C22	-0.309627817	0.000000000	-3.395390374
23	C23	0.439136469	0.000000000	3.643568474
24	H24	1.349116696	-1.307016706	5.114765643
25	H25	0.403686687	-2.153978538	3.870807806
26	H26	-0.426521545	-1.267037271	5.172263175
27	H27	0.403686690	2.153978542	3.870807805
28	H28	-0.426521544	1.267037277	5.172263175
29	H29	1.349116697	1.307016709	5.114765640
30	H30	-2.920753248	0.000000000	2.842575566
31	H31	-2.241802179	0.000000000	-4.775041187
32	H32	2.381670864	0.000000000	0.735364717
33	H33	2.593034187	0.000000000	3.182707475
34	H34	2.958047467	0.000000000	-5.474548776
35	H35	0.758504742	0.000000000	-6.633896121
36	H36	3.080487064	0.000000000	-2.983430851
37	H37	-3.434353491	0.000000000	-0.688810342

1 lowest-energy conformers used for Boltzmann-averaged ^{13}C NMR data.
Avg. Energy: -1070.524369 au.

V.72. Oblongixanthone A (D32-2).[61]

No	type	δcal	δexp	diff
C1	C	165.7 (-2.9)	162.5	3.2 (-2.9)
C2	CH	96.3	97.8	-1.5
C3	C	161.8	164.9	-3.1
C4	CH	91.9	93.8	-1.9
C4a	C	158.0	157.1	0.9
C5	C	107.1	109.4	-2.3
C6	C	144.7	145.3	-0.6

C7	C	141.2	143.4	-2.2
C8	CH	113.4 (-2.9)	108.7	4.7 (-2.9)
C8a	C	116.1	112.6	3.5
C9	C	181.6	178.9	2.7
C9a	C	104.7	101.6	3.1
C10a	C	145.8	147.3	-1.5
C1'	CH	118.1 (-3.7)	114.7	3.4 (-3.7)
C2'	CH	125.5 (+2.8)	130.8	-5.3 (+2.8)
C3'	C	80.8	77.8	3.0
C4'[2]	CH3	30.8	27.6	3.2
RMSD		2.98		
Max Abs		5.33		
RMSD+CFx		2.33		
Max abs+CFx		3.48		

mol2 coordinates for lowest energy conformer

1	C1	-0.873563145	0.000000000	-4.573990893
2	C2	0.231581345	0.000000000	-5.417474958
3	C3	1.516093045	0.000000000	-4.869859034
4	C4	1.725374831	0.000000000	-3.490958130
5	C5	0.181951202	0.000000000	0.912578080
6	C6	-0.841215718	0.000000000	1.850035595
7	C7	-2.199488452	0.000000000	1.472090589
8	C8	-2.521257034	0.000000000	0.133582417
9	C9	-1.838409733	0.000000000	-2.266437613
10	O10	-3.010195282	0.000000000	-2.673542141
11	C11	1.554541311	0.000000000	1.405634040
12	C12	1.791747093	0.000000000	2.716769517
13	C13	0.707243030	0.000000000	3.765179079
14	C14	0.788378596	1.263632794	4.622230448
15	C15	0.788378596	-1.263632794	4.622230448
16	O16	-0.626516127	0.000000000	3.183740008
17	C17	-0.172160732	0.000000000	-0.443106134
18	C18	0.614999965	0.000000000	-2.664818687
19	O19	0.860788924	0.000000000	-1.329803120
20	C20	-1.508936604	0.000000000	-0.839876566
21	C21	-0.698983862	0.000000000	-3.164769575
22	O22	-2.092148503	0.000000000	-5.111694215
23	O23	2.620093317	0.000000000	-5.649275449
24	O24	-3.163509758	0.000000000	2.424221041
25	H25	0.068990323	0.000000000	-6.491164114
26	H26	2.727415550	0.000000000	-3.081541154
27	H27	-3.557802058	0.000000000	-0.184065163
28	H28	2.808083267	0.000000000	3.101445517
29	H29	2.364728349	0.000000000	0.685333504
30	H30	1.749755341	-1.310041983	5.144147571
31	H31	0.689324508	-2.153371472	3.994565365
32	H32	-0.012382866	-1.265066997	5.368262065
33	H33	0.689324508	2.153371472	3.994565365
34	H34	-0.012382866	1.265066997	5.368262065
35	H35	1.749755341	1.310041983	5.144147571
36	H36	-2.738704973	0.000000000	-4.362517581
37	H37	2.355808330	0.000000000	-6.577292629
38	H38	-2.716699060	0.000000000	3.283166873

2 lowest-energy conformers used for Boltzmann-averaged ^{13}C NMR data.
Avg. Energy: -1145.755254 au.

V.73. Isocudraxanthone K (D32-3).[62]

No	type	δcal	δexp	diff
C1	C	164.7 (-2.9)	162.8	1.9 (-2.9)

C2	C	110.1	114.8	-4.7
C3	C	164.2	163.7	0.5
C4	CH	95.3	94.3	1.0
C4a	C	156.6	155.6	1.0
C5	C	106.9	109.5	-2.6
C6	C	144.8	147.1	-2.3
C7	C	141.0	143.2	-2.2
C8	CH	113.4 (-2.9)	108.7	4.7 (-2.9)
C8a	C	115.9	113.5	2.4
C9	C	182.2	180	2.2
C9a	C	104.3	102.5	1.8
C10a	C	145.7	145.7	0.0
C1'	C	42.2	40.9	1.3
C2'	CH	150.5	150	0.5
C3'	CH2	113.6	107.8	5.8
C4'^{2}	CH3	28.2	28.4	-0.2
C1''	CH	118.0 (-3.7)	115.0	3.0 (-3.7)
C2''	CH	125.3 (+2.8)	130.6	-5.3 (+2.8)
C3''	C	80.7	78.4	2.3
C4''^{2}	CH3	30.9	27.3	3.6
RMSD		2.84		
Max Abs		5.78		
RMSD+CFx		2.40		
Max abs+CFx		5.78		

mol2 coordinates for lowest energy conformer

1	C1	-0.241895431	1.475012755	2.297825654
2	C2	-0.038272712	0.630971230	3.402075241
3	C3	0.268813268	-0.721024424	3.101270929
4	C4	0.337459894	-1.218220834	1.799237868
5	C5	0.061612492	-0.748987539	-2.836780981
6	C6	-0.155597767	0.018328074	-3.972182224
7	C7	-0.457987349	1.393154133	-3.898067927
8	C8	-0.541014796	1.992901624	-2.661750680
9	C9	-0.415001510	1.872639981	-0.173977611
10	O10	-0.668557011	3.083995495	-0.051496957
11	C11	-0.008973265	1.110639722	4.871624802
12	C12	-0.829439449	0.151788264	5.725931124
13	C13	-0.457878692	-0.430813233	6.867300366
14	C14	-0.673012248	2.483005752	5.137320226
15	C15	1.460355576	1.201395712	5.323595201
16	O16	0.184715098	-0.896765818	-0.500581668
17	O17	-0.483398593	2.774024641	2.481166409
18	O18	0.542552649	-1.643580362	4.046142972
19	C19	-0.031386540	-0.114070744	-1.591253570
20	C20	0.109244008	-0.361990269	0.745633903
21	C21	0.373546889	-2.161809029	-3.020588703
22	C22	0.434661037	-2.676154320	-4.247890717
23	C23	0.191957178	-1.878255755	-5.505068440
24	C24	-1.027628778	-2.417856184	-6.253492180
25	C25	1.438598745	-1.881488205	-6.390605843
26	C26	-0.186999303	0.992054641	0.956180317
27	C27	-0.328626884	1.243354197	-1.492801433
28	O28	-0.661715706	2.099601653	-5.036388740
29	O29	-0.095175956	-0.479537530	-5.227650817
30	H30	0.565936279	-2.263430406	1.635337526
31	H31	-0.771186644	3.048706303	-2.576353967
32	H32	-0.611214225	3.179926144	1.581067000
33	H33	0.262748212	-1.331051329	4.923430962
34	H34	-1.147087710	-1.057086154	7.425552683

35	H35	0.534559020	-0.299880213	7.290578350
36	H36	-1.841416336	-0.016344515	5.355427814
37	H37	1.987518582	1.915270522	4.684360884
38	H38	1.976699973	0.238875101	5.247616770
39	H39	1.534362144	1.556769472	6.357468883
40	H40	-0.114018834	3.296326682	4.677005903
41	H41	-1.697028124	2.521877377	4.757422416
42	H42	-0.698813747	2.638498168	6.221246072
43	H43	-0.553848572	1.484839651	-5.776830936
44	H44	0.665534747	-3.726309522	-4.404373691
45	H45	0.552886208	-2.771701781	-2.142591379
46	H46	1.693379590	-2.904576867	-6.685660272
47	H47	1.260290111	-1.292834542	-7.296029112
48	H48	2.287040799	-1.452442158	-5.850580740
49	H49	-1.914405055	-2.370493814	-5.615362412
50	H50	-1.209054188	-1.827265248	-7.157059539
51	H51	-0.863837076	-3.459986498	-6.546399738

2 lowest-energy conformers used for Boltzmann-averaged ^{13}C NMR data.
Avg. Energy: -1341.094688 au.

V.74. Mangostenone A (D32-6).[63]

No	type	δcal	δexp	diff
C1	C	160.6 (-2.9)	157.8	2.8 (-2.9)
C2	C	114.6	116.5	-1.9
C3	C	165.9	165.7	0.2
C4	CH	89.2	88.8	0.4
C4a	C	158.0	158	0.0
C5	C	107.0	109.6	-2.6
C6	C	144.6	147.4	-2.8
C7	C	141.0	143.2	-2.2
C8	CH	113.3 (-2.9)	108.1	5.2 (-2.9)
C8a	C	116.0	113.1	2.9
C9	C	182.2	180.1	2.1
C9a	C	105.5	103.1	2.4
C10a	C	145.8	146.1	-0.3
C1'	C	43.7	43.1	0.6
C2'	CH3	25.6	24.2	1.4
C3'	CH3	21.3	19.5	1.8
C4'	CH	90.8	90.8	0.0
C5'	CH3	15.2	13.2	2.0
C1''	CH	118.1 (-3.7)	114.7	3.4 (-3.7)
C2''	CH	125.3 (+2.8)	130.3	-5.0 (+2.8)
C3''	C	80.7	78.2	2.5
C4''	CH3	30.9	26.7	4.2
C5''	CH3	30.9	26.7	4.2
RMSD		2.66		
Max Abs		5.18		
RMSD+CFx		2.11		
Max abs+CFx		4.19		

mol2 coordinates for lowest energy conformer

1	C1	-5.969595202	-1.209448751	2.982999164
2	C2	-5.894443474	1.313969397	3.094276020
3	O3	-0.580648851	0.236513510	-3.202603817
4	O4	-5.363062267	0.258290351	-1.444919224
5	O5	2.008798887	0.171670847	-3.291179000
6	C6	-2.663753673	-0.018671237	2.546534395
7	C7	-3.792019431	-0.023056048	3.254975376
8	C8	1.944635518	-0.041285451	0.889177114
9	O9	-5.124992713	0.109837892	1.188234536

10	O10	-0.382809147	0.012999298	0.881122592
11	C11	-4.121010276	0.199951089	-0.906846351
12	C12	1.982555408	0.127473740	-1.956104145
13	C13	3.118584589	-0.015871766	0.149515050
14	C14	3.164617902	0.084202066	-1.240658363
15	C15	-2.716339206	0.059328814	1.091060839
16	C16	-2.998054641	0.216838285	-1.702918351
17	C17	-3.966485498	0.121282204	0.492119825
18	C18	-0.516889598	0.174508003	-1.963445856
19	C19	0.766595669	0.028550715	0.157080486
20	C20	-1.589588671	0.077373140	0.257604453
21	C21	0.742971669	0.116045919	-1.248057094
22	C22	-1.718188454	0.155676498	-1.127458107
23	C23	-5.168028916	0.046917536	2.640868548
24	C24	4.605197332	-0.022929700	-1.692219943
25	C25	5.307552646	0.235861363	-0.324807975
26	O26	4.341949248	-0.096886432	0.705312031
27	C27	4.860352032	-1.430827126	-2.255014901
28	C28	5.021651001	1.036102217	-2.717010935
29	C29	6.594430736	-0.507740571	-0.043439780
30	H30	-6.963096578	-1.159670659	2.526103084
31	H31	-5.453665362	-2.099517073	2.613330568
32	H32	-6.090069245	-1.300854444	4.067488960
33	H33	-5.322582936	2.201153594	2.807375516
34	H34	-6.016880050	1.314120563	4.182438598
35	H35	-6.885649914	1.366607472	2.633124158
36	H36	-5.993918777	0.232767443	-0.710818164
37	H37	1.064738735	0.202348162	-3.594211556
38	H38	-1.693994354	-0.070785059	3.028175581
39	H39	-3.772207312	-0.078054146	4.340062054
40	H40	1.935734935	-0.107988726	1.969304274
41	H41	-3.088114961	0.277128153	-2.781576111
42	H42	5.482542873	1.317714684	-0.235299874
43	H43	5.906002729	-1.555924138	-2.558968517
44	H44	4.227585921	-1.589232814	-3.133196153
45	H45	4.617434527	-2.204207262	-1.518746372
46	H46	6.099593264	0.978232376	-2.910975720
47	H47	4.493772216	0.882480327	-3.660956379
48	H48	4.788705828	2.044043150	-2.359262324
49	H49	6.991990963	-0.215104239	0.931827725
50	H50	6.436736170	-1.589305945	-0.043171519
51	H51	7.341358711	-0.262627225	-0.806244415

2 lowest-energy conformers used for Boltzmann-averaged ^{13}C NMR data.
Avg. Energy: -1341.134155 au.

V.75. Cudratrixanthone J (D32-7).[64]

No	type	δcal	δexp	diff
C1	C	160.7 (-2.9)	158.7	2.0 (-2.9)
C2	C	115.0	117.4	-2.4
C3	C	164.7	166.5	-1.8
C4	CH	89.3	90	-0.7
C4a	C	157.9	158.8	-0.9
C5	C	109.3	110.4	-1.1
C6	C	145.1	147	-1.9
C7	C	142.0	143.7	-1.7
C8	CH	113.1 (-2.9)	109.3	3.8 (-2.9)
C8a	C	116.5	114.4	2.1
C9	C	182.4	181	1.4
C9a	C	105.9	104.2	1.7
C10a	C	145.7	148	-2.3

C1'	C	43.3	43.6	-0.3
C2'	CH3	21.7	20.6	1.1
C3'	CH3	26.6	26.8	-0.2
C4'	CH	95.2	95.6	-0.4
C5'	CH2	60.8	61.3	-0.5
C1''	CH	119.2 (-3.7)	115.8	3.4 (-3.7)
C2''	CH	126.9 (+2.8)	131.5	-4.6 (+2.8)
C3''	C	79.7	79.4	0.3
C4''	CH3	28.1	28.1	0.0
C5''	CH3	29.2	28.1	1.1
RMSD		1.95		
Max Abs		4.62		
RMSD+CFx		1.33		
Max abs+CFx		2.42		

mol2 coordinates for lowest energy conformer

1	O1	-0.744748464	0.625639293	-3.133066717
2	C2	0.587357503	-2.939001896	0.102617597
3	C3	-0.031794949	4.119659723	0.382580644
4	C4	0.227457280	-3.032315269	-1.238590763
5	C5	-0.424548540	4.229385040	-0.965465434
6	C6	0.654185975	-1.751653440	0.815102686
7	C7	0.251439364	2.895377316	0.975002304
8	C8	-0.116401490	-1.881375769	-1.925543210
9	C9	-0.546279098	3.083012428	-1.718949496
10	O10	0.402552672	0.562982821	0.796626741
11	C11	-0.390458222	0.603576709	-1.943392900
12	C12	0.326633645	-0.603087639	0.104887413
13	C13	0.120018506	1.745062457	0.185358758
14	C14	-0.057224607	-0.626208377	-1.248996552
15	C15	-0.275309696	1.828246325	-1.148641898
16	O16	0.866102984	-4.142166118	0.650864217
17	C17	0.925063924	-5.115149905	-0.425163468
18	C18	0.193247434	-4.487209313	-1.653565386
19	O19	0.112719323	5.289861093	1.051334360
20	C20	-0.028262302	5.271603777	2.497007726
21	C21	0.629893509	4.034254818	3.067122539
22	C22	0.721672151	2.908802786	2.356661012
23	C23	0.668564944	6.542044250	2.967274307
24	C24	-1.521879987	5.286223693	2.831945831
25	C25	-1.262587164	-4.948798247	-1.841742435
26	C26	0.991919365	-4.775480822	-2.931277600
27	C27	0.418567214	-6.431443102	0.151079747
28	O28	-0.497949877	-1.955568324	-3.202171927
29	O29	-0.674111657	5.449888619	-1.496392190
30	O30	-0.817966016	-6.301423005	0.806816242
31	H31	0.950141895	-1.707597359	1.854832706
32	H32	-0.856093942	3.133223713	-2.756505083
33	H33	1.987842033	-5.232849848	-0.679532397
34	H34	0.985794021	4.089739561	4.092107201
35	H35	1.157980920	2.005539784	2.768348732
36	H36	0.583756096	6.636435634	4.054437783
37	H37	1.728019118	6.519262569	2.697020046
38	H38	0.206067925	7.419932042	2.506400834
39	H39	-2.014283014	4.399962762	2.420459812
40	H40	-1.996038123	6.181751005	2.417327702
41	H41	-1.665709853	5.283542407	3.917377390
42	H42	-1.712558458	-4.375486335	-2.658220029
43	H43	-1.853515068	-4.804233350	-0.936099111
44	H44	-1.308542090	-6.012064867	-2.104294968

45	H45	2.011357467	-4.383552494	-2.859078266
46	H46	1.048392168	-5.855934295	-3.109075432
47	H47	0.509643577	-4.311488827	-3.793807714
48	H48	1.185479892	-6.830220368	0.833029977
49	H49	0.288192636	-7.156918549	-0.657443062
50	H50	-0.684562481	-1.028658399	-3.499383177
51	H51	-0.539414669	6.099884137	-0.791006578
52	H52	-0.719823775	-5.555008844	1.413781486

5 lowest-energy conformers used for Boltzmann-averaged ^{13}C NMR data.
Avg. Energy: -1416.352255 au.

V.76. Caledonixanthone E (D32-8).[65]

No	type	δcal	δexp	diff
C1	C	160.7 (-2.9)	155.3	5.4 (-2.9)
C2	C	132.8	131.8	1.0
C3	C	156.8	153.9	2.9
C4	C	101.8	101.3	0.5
C4a	C	147.9	146.7	1.2
C5	C	145.0	144.2	0.8
C6	CH	119.3	120.8	-1.5
C7	CH	124.2	124.8	-0.6
C8	CH	118.4 (-2.9)	117	1.4 (-2.9)
C8a	C	122.5	120.4	2.1
C9	C	183.2	181.1	2.1
C9a	C	105.6	103.5	2.1
C10a	C	143.4	144.3	-0.9
C1'	CH	118.3 (-3.7)	114.7	3.6 (-3.7)
C2'	CH	126.1 (+2.8)	127.8	-1.7 (+2.8)
C3'	C	78.1	78.7	-0.6
C4'[2]	CH3	27.5	28.1	-0.6
C1''	CH3	59.4	61.1	-1.7
RMSD		2.05		
Max Abs		5.38		
RMSD+CFx		1.49		
Max abs+CFx		2.89		

mol2 coordinates for lowest energy conformer

1	C1	0.447566489	-0.607516223	-2.072020805
2	C2	0.348755251	-1.745459862	-1.267899498
3	C3	0.030104100	-1.598054158	0.085550446
4	C4	-0.173160722	-0.335822324	0.674917986
5	C5	-0.389637325	4.321297767	0.422153025
6	C6	-0.324416459	5.526176252	-0.258344185
7	C7	-0.033519692	5.550466162	-1.627174024
8	C8	0.194301602	4.377038788	-2.322506884
9	C9	0.362221901	1.872532632	-2.338892374
10	O10	0.588496916	-2.982601563	-1.784685020
11	C11	-0.588743723	-0.299451381	2.071705185
12	C12	-0.515772573	-1.408133174	2.809237538
13	C13	0.069114209	-2.677062384	2.234573100
14	C14	1.575356795	-2.749167713	2.501507920
15	C15	-0.651367962	-3.912766344	2.759331728
16	C16	-0.543706703	-3.568706860	-2.409903253
17	O17	0.623231424	1.825876668	-3.549077326
18	O18	-0.668334487	4.296275488	1.746552860
19	O19	-0.248083917	1.991442673	0.449360156
20	O20	0.731139794	-0.769215016	-3.361976346
21	C21	0.130289361	3.150170758	-1.649514711
22	C22	-0.162596846	3.134750580	-0.289942650
23	C23	-0.048552998	0.780253169	-0.142806678

24	C24	0.259265090	0.684236699	-1.509925181
25	O25	-0.130843200	-2.723500379	0.799315925
26	H26	-0.221742267	-4.546027970	-2.774089772
27	H27	-1.361926494	-3.699013727	-1.691415002
28	H28	-0.884726467	-2.959585074	-3.254378775
29	H29	-1.721261317	-3.850866127	2.541681195
30	H30	-0.516270808	-3.995428026	3.842427566
31	H31	-0.247271988	-4.812456302	2.286616892
32	H32	1.992979891	-3.661623741	2.064429716
33	H33	2.081143912	-1.883542795	2.062436312
34	H34	1.769494687	-2.749144825	3.579029904
35	H35	-0.645729366	3.375799516	2.042363997
36	H36	0.767278609	0.134482864	-3.763617883
37	H37	-0.986305138	0.624652690	2.477380031
38	H38	-0.841363221	-1.427618008	3.844972774
39	H39	0.013481026	6.504676801	-2.141160169
40	H40	-0.502485651	6.441664422	0.295643237
41	H41	0.423598266	4.370970044	-3.381856958

1 lowest-energy conformers used for Boltzmann-averaged ^{13}C NMR data.
Avg. Energy: -1185.043486 au.

VI-1. Pyranoxanthones whose structures have been revised in this study and validated by DFT ^{13}C NMR calculations.

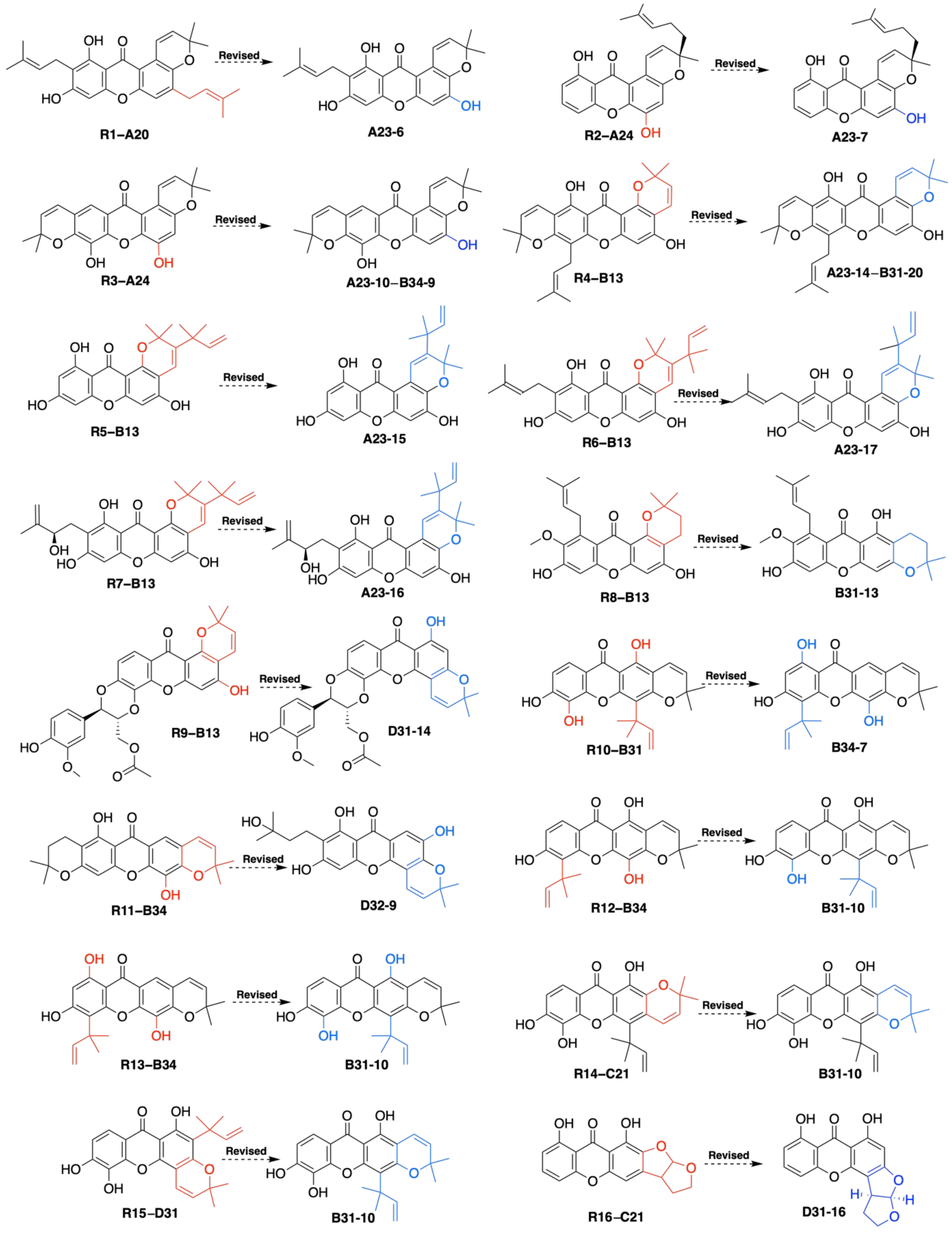

VI-2. Pyranoxanthones whose structures have been revised in this study and validated by DFT ^{13}C NMR calculations.

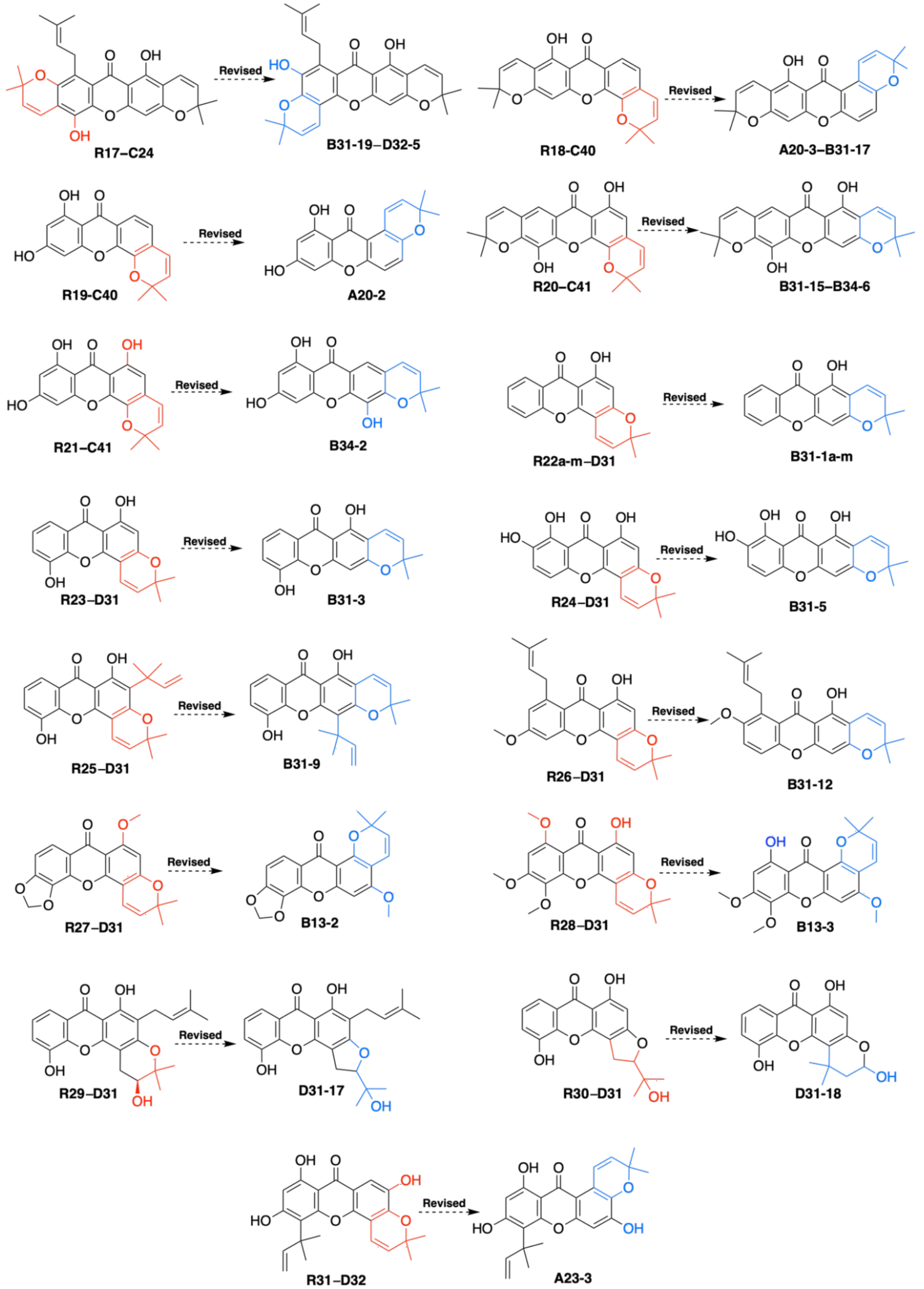

VII. 13C NMR chemical shifts (δ, ppm) of revised pyranoxanthones compared with reference compounds (shaded columns). Chemical shifts are listed in decreasing order. Multiplicities (mult.) are indicated. Deviations from expected values are marked with $ symbol.

VII.1. Table SI-4. The ^{13}C NMR chemical shifts of the revised pyranoxanthone structures, along with the corresponding data for the reference compounds.

R1-A20[68]		A23-6[23]		R4–B13[69]		A23-14–B31-20[26]		R5–B13[70]		A23-15[71]		R6–B13[72]		A23-17[73]	
mult.	δ(ppm)	mult.	δ(ppm)	mult.	δexp.	mult.	δexp.	mult.	δ(ppm)	mult.	δ(ppm)	mult.	δ(ppm)	mult.	δ(ppm)
C	182.5	C	181.5	C	182.7	C	182.7	C	183.1	C	183.2	C	183.1	C	183.1
C	159.8	C	162.4	C	157.3	C	157.4	C	165.4	C	165.5	C	162.9	C	162.9
C	158.0	C	159.7	C	155.7	C	155.8	C	164.7	C	164.8	C	161.5	C	161.5
C	156.5	C	154.3	C	153.7	C	153.7	C	158.1	C	158.2	C	155.9	C	155.9
C	153.1	C	153.2	C	152.9	C	153.0	C	155.6	C	155.7	C	154.1	C	154.1
C	150.9	C	152.3	C	150.7	C	150.8	C	153.6	C	153.7	C	153.3	C	153.3
C	136.9	C	138.1	C	136.7	C	136.8	C	149.9	C	150.0	C	149.7	C	149.7
CH	132.3	C	132.6	C	132.3	CH	132.2	CH	147.9	CH	148.0	CH	147.9	CH	147.9
C	131.3	CH	130.4	CH	132.2	C	131.3	C	137.8	C	137.9	C	137.6	C	137.6
CH	121.1	CH	122.5	CH	126.9	CH	127.0	C	122.8	C	122.9	C	131.4	C	131.4
CH	121.0	CH	120.4	CH	122.2	CH	122.3	CH	118.7	CH	118.8	CH	123.4	CH	123.4
C	119.7	C	119.7	CH	120.9	C	121.0	CH_2	112.3	CH_2	112.4	C	122.8	C	122.8
C	108.6	C	109.7	C	119.6	CH	119.6	C	108.3	C	108.3	CH	118.8	CH	118.8
C	104.4	C	106.8	CH	115.9	CH	116.0	C	103.9	C	104.0	CH_2	112.2	CH_2	112.2
C	103.9	C	102.7	C	108.3	C	108.4	CH	103.0	CH	103.1	C	110.9	C	110.9
CH	102.4	CH	102.1	C	106.5	C	106.6	CH	98.7	CH	98.8	C	108.4	C	108.4
CH	94.3	CH	92.4	C	104.1	C	104.2	CH	93.9	CH	94.0	C	103.8	C	103.8
C	78.0	C	75.1	C	103.6	C	103.7	C	80.4	C	80.5	CH	102.9	CH	102.9
CH_3	27.4	CH_3	26.8	CH	102.3	CH	102.3	C	42.7	C	42.8	CH	93.2	CH	93.2
CH_3	27.4	CH_3	26.8	C	77.7	C	77.8	CH_3	28.6	CH_3	28.7	C	80.3	C	80.3
CH_3	25.8	CH_3	25.6	C	77.1	C	76.9	CH_3	28.6	CH_3	28.7	C	42.7	C	42.7
CH_2	21.5	CH_2	21.0	CH_3	28.2	CH_3	28.3	CH_3	27.3	CH_3	27.4	CH_3	28.6	CH_3	28.6
CH_3	18.0	CH_3	17.8	CH_3	28.2	CH_3	28.3	CH_3	27.3	CH_3	27.4	CH_3	28.6	CH_3	28.6
CH_2	22.6[$]			CH_3	27.2	CH_3	27.3					CH_3	27.3	CH_3	27.3
CH	121.5[$]			CH_3	27.2	CH_3	27.3					CH_3	27.3	CH_3	27.3
C	132.6[$]			CH_3	25.7	CH_3	25.8					CH_3	25.9	CH_3	25.9
CH_3	25.9[$]			CH_2	21.2	CH_2	21.3					CH_2	21.9	CH_2	21.9
CH_3	17.9[$]			CH_3	17.9	CH_3	18.0					CH_3	17.8	CH_3	17.8

VII.2. Table SI-4. The ^{13}C NMR chemical shifts of the revised pyranoxanthone structures, along with the corresponding data for the reference compounds (continue).

R7–B13[74]		A23-16[71]		R8–B13[75]		B31-13[76, 77]		R10–B31[5]		B34-7[5]		R16–C21a[78]		D31-16[79]	
mult.	δ(ppm)	mult.	δ(ppm)	mult.	δ(ppm)	mult.	δ(ppm)	mult.	δexp.	mult.	δexp.	mult.	δ(ppm)	mult.	δ(ppm)
C	182.5	C	182.6	C	183.1	C	182.0	C	180.9	C	180.9	C	184.4	C	184.6
C	163.2	C	163.3	C	161.8	C	160.7	C	162.7	C	162.7	C	166.0	C	166.2
C	161.1	C	161.2	C	161.5	C	160.6	C	162.0	C	162.0	C	164.1	C	164.3
C	155.8	C	155.9	C	158.3	C	155.9	C	155.9	C	155.9	C	161.4	C	161.6
C	153.4	C	153.4	C	156.8	C	154.7	CH	150.6	CH	150.6	C	155.7	C	155.9
C	150.6	C	150.7	C	155.9	C	154.5	C	145.2	C	145.2	C	155.7$^{\#}$	C	152.2
C	148.8	C	148.9	C	144.8	C	142.4	C	144.9	C	144.9	CH	145.3	CH	145.6
CH	146.8	CH	146.9	C	138.4	C	136.9	C	132.6	C	132.6	CH	136.8	CH	137.0
C	146.7	C	146.8	C	131.6	C	132.0	CH	131.2	CH	131.2	CH	113.1	CH	113.3
C	135.7	C	135.8	CH	125.2	CH	123.7	CH	121.6	CH	121.6	CH	111.3	CH	111.6
C	121.7	C	121.8	C	111.9	C	112.1	CH	118.2	CH	118.2	C	107.4	C	107.6
CH	118.0	CH	118.1	C	104.6	C	110.0	C	114.4	C	114.4	C	107.4	CH	107.0
CH_2	112.0	CH_2	112.1	C	103.5	C	103.8	CH	113.4	CH	113.4	C	107.4	C	105.5
CH_2	110.2	CH_2	110.3	CH	102.9	CH	101.6	CH_2	112.6	CH_2	112.6	CH	106.8$^{\#}$	C	103.1
C	108.5	C	108.6	CH	94.8	CH	94.0	C	110.7	C	110.7	CH	102.4	CH	102.6
C	107.4	C	107.5	C	77.0	C	76.0	C	104.1	C	104.1	CH	94.4	CH	94.6
C	103.4	C	103.5	CH_3	61.3	CH3	62.0	C	100.6	C	100.6	CH	47.5	CH	47.7
CH	101.8	CH	101.9	CH_2	32.8	CH_2	31.9	C	79.1	C	79.1				
CH	94.5	CH	94.6	CH_3	27.1	CH_2	26.7	C	41.4	C	41.4				
C	80.6	C	80.7	CH_3	27.1	CH_3	26.5	CH_3	28.7	CH_3	28.7				
CH	77.5	CH	77.7	CH_2	26.0	CH_3	26.5	CH_3	28.7	CH_3	28.7				
C	42.7	C	42.2	CH_3	26.0	CH_3	25.8	CH_3	27.9	CH_3	27.9				
CH_3	28.3	CH_3	28.4	CH_2	18.3	CH_3	18.2	CH_3	27.9	CH_3	27.9				
CH_3	28.3	CH_3	28.3	CH_3	18.3	CH_2	16.1								
CH_2	28.1	CH_2	28.1												
CH_3	27.4	CH_3	27.5												
CH_3	27.4	CH_3	27.5												
CH_3	18.7	CH_3	18.8												

VII.3. Table SI-4. The ^{13}C NMR chemical shifts of the revised pyranoxanthone structures, along with the corresponding data for the reference compounds (continue).

R12–B34[4]		R13–B34[5]		R14–C21[6]		R15–D31[7]		B31-10[2]		R17–C24[80]		B31-19–D32-5[28]		R18–C40[81]		A20-3–B31-17[14]	
mult	δ(ppm)	mult	δ(ppm)	mult	δ(ppm)	mult	δ(ppm)	mult	δexp.	mult	δexp.	mult	δ(ppm)	mult	δ(ppm)	mult	δ(ppm)
C	181.1	C	181.0	C	180.9	C	181.4	C	181.1	C	183.4	C	182.4	C	183.4	C	183.9
C	158.8	C	159.1	C	159.0	C	159.2	C	158.8	C	160.6	C	159.6	C	160.5	C	160.5
C	156.5	C	157.0	C	156.8	C	157.0	C	156.5	C	158.6	C	157.9	C	157.9	C	158.0
C	155.3	C	154.3	C	154.2	C	155.5	C	155.2	C	157.0	C	156.0	C	156.7	C	157.0
CH	151.8	CH	157.0$^{\#}$	CH	156.9$^{\#}$	CH	152.8	CH	152.0	C	147.9	C	147.3	C	151.7	C	152.0
C	151.2	C	149.3	C	149.1	C	151.3	C	151.0	C	146.9	C	144.7	C	149.3	C	149.5
C	146.0	C	144.8	C	144.6	C	146.3	C	146.0	C	141.5	C	139.4	CH	132.7	CH	132.6
C	133.0	C	131.3	C	131.1	C	133.2	C	132.8	C	131.5	C	132.4	CH	127.3	CH	127.2
CH	127.5	CH	127.4	CH	127.3	CH	127.3	CH	127.2	CH	130.8	CH	129.0	CH	124.3	CH	124.2
CH	116.2	CH	117.7	CH	117.6	CH	116.8	CH	116.2	C	128.9	C	127.9	CH	120.8	CH	120.8
CH	115.6	CH	116.3	CH	116.2	CH	116.2	CH	115.5	CH	128.4	CH	127.0	C	119.9	C	118.1
C	113.5	C	113.9	C	113.8	C	114.0	C	113.6	CH	124.2	CH	122.4	CH	117.8	CH	117.7
CH	112.9	C	113.3	C	113.2	C	113.8	C	113.4	CH	116.0	CH	115.8	CH	115.6	CH	115.5
C	104.9$^{\#}$	C	113.0	CH	112.9	CH	113.4	CH	112.8	CH	115.8	CH	115.5	C	115.1	C	114.5
CH_2	106.7	CH_2	103.6$^{\#}$	CH_2	103.4$^{\#}$	CH_2	106.7	CH_2	106.5	C	113.0	C	111.9	C	104.4	C	104.3
C	104.9	CH	105.8	C	105.7	C	105.4	C	104.9	C	108.0	C	106.9	C	104.3	C	104.0
C	102.8	C	105.8	C	103.4	C	103.4	C	102.9	C	105.0	C	104.3	CH	94.3	CH	94.2
C	78.2	C	78.5	C	78.3	C	78.9	C	78.2	C	104.3	C	103.8	C	78.2	C	78.2
C	41.0	C	41.7	C	41.5	C	41.5	C	41.0	CH	94.6	CH	93.9	C	75.5	C	75.4
CH_3	29.3	CH_3	28.4	CH_3	28.3	CH_3	29.8	CH_3	29.1	C	79.6	C	79.2	CH_3	28.4	CH_3	28.3
CH_3	29.3	CH_3	28.4	CH_3	28.3	CH_3	29.8	CH_3	29.1	C	78.7	C	77.8	CH_3	28.4	CH_3	28.3
CH_3	27.2	CH_3	28.2	CH_3	28.0	CH_3	27.9	CH_3	27.2	CH_3	28.4	CH_3	28.4	CH_3	27.3	CH_3	27.3
CH_3	27.2	CH_3	28.2	CH_3	28.0	CH_3	27.9	CH_3	27.2	CH_3	28.4	CH_3	28.4	CH_3	27.3	CH_3	27.3
										CH_3	28.2	CH_3	28.2				
										CH_3	28.2	CH_3	28.2				
										CH_2	26.6	CH_3	26.0				
										CH_3	26.0	CH_2	25.7				
										CH_3	18.2	CH_3	18.1				

VII.4. Table SI-4. The ^{13}C NMR chemical shifts of the revised pyranoxanthone structures, along with the corresponding data for the reference compounds (continue).

R19–C40[82]		A20-2[13]		R20–C41[6]		B31-15–B34-6[26]		R21–C41[83]		B34-2[43]		R22b–D31[84]		B31-1b[58]	
mult.	δ(ppm)	mult.	δ(ppm)	mult.	δ(ppm)	mult.	δ(ppm)	mult.	δexp.	mult.	δexp.	mult.	δ(ppm)	mult.	δ(ppm)
C	183.6	C	183.6	C	180.3	C	178.8	C	181.5	C	179.4	C	180.9	C	180.8
C	164.5	C	164.2	C	160.6	C	159.4	C	167.2	C	165.2	C	161.0	C	160.9
C	163.1	C	163.1	C	157.8	C	157.8	C	164.7	C	162.8	C	157.8	C	157.7
C	157.6	C	157.6	C	156.9	C	156.9	C	159.3	C	157.2	C	157.2	C	157.1
C	151.7	C	151.9	C	145.2	C	145.0	C	147.6	C	146.0	C	156.0	C	155.9
C	149.6	C	149.6	C	144.8	C	144.8	C	147.3	C	146.0	CH	134.9	CH	134.9
CH	133.0	CH	133.0	C	132.2	C	132.1	C	134.5	C	133.0	CH	127.6	CH	127.6
CH	124.6	CH	124.6	CH	131.1	CH	131.1	CH	132.7	CH	131.6	CH	125.8	CH	125.8
C	121.0	C	121.0	CH	127.6	CH	127.6	CH	122.4	CH	121.1	CH	124.0	CH	124.0
CH	120.2	CH	120.2	CH	121.5	CH	121.5	C	119.8	C	118.2	C	120.6	C	120.5
CH	117.9	CH	117.9	C	117.9	C	117.9	C	115.6	C	113.7	CH	117.7	CH	117.6
C	115.1	C	115.1	CH	115.5	CH	115.5	CH	113.5	CH	112.0	CH	115.5	CH	115.4
C	107.7	C	107.7	C	114.8	C	114.7	C	103.3	C	101.6	C	104.7	C	104.6
CH	98.4	CH	98.4	CH	113.6	CH	113.6	CH	99.2	CH	97.9	C	103.8	C	103.8
CH	93.6	CH	93.6	C	104.9	C	104.8	CH	95.2	CH	93.9	CH	95.2	CH	107.1[#]
C	75.7	C	75.7	C	103.3	C	103.8	C	79.2	C	77.6	C	78.4	C	78.3
CH_3	27.5	CH_3	27.5	CH	95.4	CH	95.4	CH_3	28.4	CH_3	27.9	CH_3	28.5	CH_3	28.4
CH_3	27.5	CH_3	27.5	C	79.0	C	79.0	CH_3	28.4	CH_3	27.9	CH_3	28.5	CH_3	28.4
				C	78.3	C	78.2								
				CH_3	28.6	CH_3	28.5								
				CH_3	28.6	CH_3	28.5								
				CH_3	28.5	CH_3	28.4								
				CH_3	28.5	CH_3	28.4								

VII.5. Table SI-4. The 13C NMR chemical shifts of the revised pyranoxanthone structures, along with the corresponding data for the reference compounds (continue).

R22i–D31[84]		B31-1i[85]		R23–D31[86]		B31-3[26]		R25–D31[87]		B31-9[2]		R26–D31[88]		B31-12[17]	
mult.	δ(ppm)	mult.	δ(ppm)	mult.	δ(ppm)	mult.	δ(ppm)	mult.	δ(ppm)	mult.	δ(ppm)	mult.	δexp.	mult.	δexp.
C	175.1	C	175.1	C	182.0	C	181.0	C	181.3	C	181.3	C	183.4	C	183.2
C	159.2	C	159.2	C	161.8	C	160.8	C	159.4	C	159.4	C	160.4	C	160.2
C	158.6	C	158.6	C	158.6	C	157.7	C	156.6	C	156.7	C	158.2	C	158.0
C	156.3	C	158.0	C	157.8	C	156.9	CH	155.8	CH	155.8	C	155.7	C	156.0
C	155.0	C	155.1	C	147.0	C	146.1	C	153.9	C	153.9	C	153.6	C	153.5
CH	133.9	CH	133.9	C	146.1	C	145.2	C	145.3	C	145.3	C	151.7	C	151.7
C	133.9	C	133.8	CH	129.1	CH	128.1	C	144.1	C	144.1	C	132.1	C	131.9
CH	130.2	CH	130.2	CH	125.2	CH	124.2	CH	127.3	CH	127.3	C	132.0	C	—
CH	126.5	CH	126.6	C	122.2	C	121.3	CH	124.2	CH	124.2	CH	127.2	CH	115.7[#]
C	123.7	CH	123.8	CH	121.6	CH	120.7	C	120.4	CH	120.5	CH	122.8	CH	122.8
CH	122.5	C	122.7	CH	116.4	CH	115.4	CH	119.6	C	119.6	CH	118.7	CH	118.7
CH	117.1	CH	117.2	CH	115.7	CH	114.8	CH	116.0	CH	116.0	CH	116.0	C	118.7
CH	115.9	CH	116.1	C	105.4	C	104.5	CH	116.0	CH	116.0	C	115.9	C	115.8
CH	112.2	C	112.3	C	104.3	C	103.3	C	113.2	C	113.1	CH	115.8	CH	115.8
C	100.6	CH	100.7	CH	95.8	C	94.9	C	105.5	C	105.5	C	104.3	CH	104.1
C	77.9	C	78.5	C	79.3	C	78.4	CH_2	104.0	CH_2	104.0	C	104.3	C	104.1
CH_3	62.6	CH3	62.7	CH_3	28.6	CH_3	27.7	C	103.8	C	103.6	CH	94.1	C	94.0
CH_3	28.3	CH3	28.4	CH_3	28.6	CH_3	27.7	C	78.6	C	78.4	C	78.1	CH	—
CH_3	28.3	CH3	28.4					C	41.3	C	41.3	CH_3	56.8	CH_3	56.7
								CH_3	28.2	CH_3	28.2	CH_3	28.4	CH_3	28.4
								CH_3	28.2	CH_3	28.2	CH_3	28.4	CH_3	28.4
								CH_3	27.9	CH_3	27.9	CH_3	26.1	CH_2	25.6
								CH_3	27.9	CH_3	27.9	CH_2	25.7	CH_3	25.0
												CH_3	18.2	CH_3	18.1

VII.6. Table SI-4. The ^{13}C NMR chemical shifts of the revised pyranoxanthone structures, along with the corresponding data for the reference compounds (continue).

**R27–D31**[89]		**B13-2**[33]		**R29–D31**[90]		**D31-17**[91]		**R30–D31**[92]		**D31-18**[93]		**R31–D32**[94]		**A23-3**[17]	
mult.	δ(ppm)	mult.	δ(ppm)	mult.	δ(ppm)	mult.	δ(ppm)	mult.	δ(ppm)	mult.	δ(ppm)	mult.	δ(ppm)	mult.	δ(ppm)
C	174.0	C	174.0	C	180.1	C	181.5	C	182.1	C	181.2	C	183.2	C	182.8
C	159.4	C	159.4	C	165.4	C	167.3	C	162.1	C	161.1	C	162.4	C	162.0
C	158.1	C	158.0	C	160.5	C	161.9	C	161.6	C	160.6	C	162.2	C	161.8
C	155.4	C	155.3	C	149.8	C	151.2	C	156.7	C	155.7	C	155.6	C	155.2
C	155.4	C	152.2	C	145.5	C	146.8	C	147.4	C	146.8	C	153.0	C	152.6
C	152.3[#]	C	140.0	C	144.5	C	145.9	C	146.3	C	145.3	C	151.4	C	151.0
C	133.6	C	133.5	C	130.7	C	132.0	CH	124.8	CH	123.9	CH	149.7	CH	149.3
CH	127.7	CH	127.6	CH	123.4	CH	124.7	C	122.3	C	121.2	C	137.4	C	136.9
CH	121.4	CH	121.3	C	121.3	CH	122.7	CH	120.9	CH	119.9	CH	132.8	CH	132.4
C	119.3	C	119.2	CH	121.3	C	122.3	CH	116.1	CH	114.8	CH	121.3	CH	120.9
CH	115.8	CH	115.8	CH	120.2	CH	121.5	C	110.6	C	109.6	C	120.0	C	119.6
C	112.8	C	107.2	CH	115.2	CH	116.5	C	105.1	C	104.1	CH_2	113.7	CH_2	113.3
C	107.2	C	106.8	C	105.7	C	107.0	CH	100.0	CH	99.0	C	109.4	C	109.0
CH	107.2	CH	105.5	C	103.2	C	104.6	CH	94.0	CH	93.1	C	108.5	C	108.2
CH_2	102.9	CH_2	102.9	C	102.7	C	103.8	CH_2	46.8	CH_2	45.9	C	105.0	C	104.6
CH	91.4	CH	91.4	CH	91.2	CH	92.6	C	109.2[#]	C	31.9	CH	102.3	CH	101.9
C	79.1	C	77.6	C	70.2	C	71.6	CH_3	32.8[#]	CH_3	28.1	CH	100.6	CH	100.2
CH_3	55.9	CH_3	55.9	CH_2	26.5	CH_2	27.9	CH_3	32.8[#]	CH_3	28.0	C	77.5	C	—
CH_3	27.8	CH_3	27.8	CH_3	25.0	CH_3	26.4					C	41.3	C	40.9
CH_3	27.8	CH_3	17.4	CH_3	24.5	CH_3	25.9					CH_3	28.4	CH_3	28.0
				CH_3	23.7	CH_3	25.0					CH_3	28.4	CH_3	28.0
				CH_2	21.0	CH_2	22.4					CH_3	27.7	CH_3	27.3
				CH_3	16.5	CH_3	17.9					CH_3	27.7	CH_3	27.3

VIII. ^{13}C NMR data experimental and calculated and the cartesian coordinates of the global minimum conformer of the revised pyranoxantones.

VIII.1. Dulcisxanthone A (R1–A20)[68] and its revised structure garcinone B (A23-6).[23]

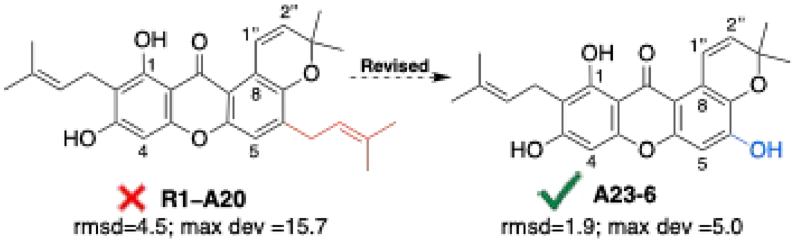

VIII.1a. Dulcisxanthone A (R1–A20).[68]

No	type	δcal	δexp	diff
C1	C	162.4	158	4.4
C3	C	164.7	159.8	4.9
C4	CH	93.8	94.3	-0.5
C4a	C	156.5	156.5	0
C5	CH	118.1	102.4	15.7
C6	C	138.5	136.9	1.6
C7	C	147.6	150.9	-3.3
C8	C	123.9	119.7	4.2
C8a	C	115.6	108.6	7
C9	C	185.3	182.5	2.8
C9a	C	104.8	103.9	0.9
C10a	C	151.3	153.1	-1.8
C1'	CH2	22.8	21.5	1.3
C2'	CH	126.8	121.1	5.7
C3'	C	136.1	131.3	4.8
C4'	CH3	16.8	18	-1.2
C5'	CH3	25.4	25.8	-0.4
C1''	CH	124.6	121	3.6
C2''	CH	131.8	132.3	-0.5
C3''	C	75.5	78	-2.5
C4''^{2}	CH3	27.2	27.4	-0.2
C1'''	CH2	32.7	22.6	10.1
C2'''	CH	124.4	121.5	2.9
C3'''	C	132.1	132.6	-0.5
C4'''	CH3	25.7	25.9	-0.2
C5'''	CH3	19.3	17.9	1.4
RMSD		4.53		
Max abs	15.74			

mol2 coordinates for lowest energy conformer

1	C1	-0.629882629	4.538847341	-2.150634550
2	C2	1.703780655	5.456524783	-2.434592626
3	O3	0.620133538	-0.964008466	-2.224316559
4	C4	-0.544163737	-5.793833136	-0.658609276
5	C5	1.214182192	1.808292876	-2.301794932
6	C6	0.299557051	-6.878731993	-0.033959493
7	C7	1.248539859	3.029136048	-2.840130151
8	C8	0.585566408	2.791198299	1.262843145
9	C9	0.294631581	1.578809332	1.858343247
10	C10	-0.450018239	-2.999902672	1.974984916
11	C11	-0.023566810	-3.322269648	-0.781076598
12	O12	1.191616993	4.014653960	-0.649484932
13	O13	-0.861614642	-5.284112333	2.179435197

14	O14	-0.057749138	-0.712118333	1.803079210
15	C15	-0.350597994	-4.438619096	-0.010659142
16	C16	0.854626007	2.810835196	-0.116679023
17	C17	-0.547608218	-4.252272613	1.370665753
18	C18	0.842189231	1.650407983	-0.892288653
19	C19	0.428875558	-0.867420124	-0.997976268
20	C20	0.256787689	0.410089622	1.100255174
21	C21	-0.137381976	-1.911963382	1.182446391
22	C22	0.095048263	-2.028902226	-0.196729184
23	C23	0.518118326	0.412213698	-0.274045165
24	C24	0.868676231	4.246827679	-2.034308255
25	C25	0.902189637	-9.041176585	1.017647438
26	C26	-0.097544844	-8.069241723	0.442516085
27	C27	-1.518969971	-8.571689247	0.441169994
28	O28	0.166326497	-3.486361507	-2.093430318
29	C29	0.555516466	4.082362205	2.045896291
30	C30	-0.735073514	4.822993005	1.798127070
31	C31	-0.895717301	6.039397406	1.268953034
32	C32	0.228471465	6.933181676	0.814531261
33	C33	-2.272599354	6.628871584	1.098514194
34	H34	-0.898929406	4.728757538	-3.194694850
35	H35	-0.891393951	5.414570183	-1.548100351
36	H36	-1.213763265	3.684612111	-1.796519186
37	H37	1.446786352	6.318447069	-1.812715637
38	H38	1.514116273	5.713685464	-3.481361895
39	H39	2.768582478	5.239571804	-2.313262631
40	H40	-0.277366180	-5.701696884	-1.715479118
41	H41	-1.607751378	-6.058988682	-0.633449522
42	H42	1.453357319	0.925563045	-2.876612209
43	H43	1.362136896	-6.638689993	0.023385934
44	H44	1.526820336	3.176290693	-3.880319899
45	H45	0.075183414	1.515488555	2.919441586
46	H46	-0.608223311	-2.888721761	3.039981700
47	H47	-0.652183914	-6.118971574	1.725802798
48	H48	0.897002304	-9.979396016	0.449345199
49	H49	1.918473476	-8.638978341	1.003641762
50	H50	0.646200744	-9.299660482	2.053660162
51	H51	-2.245292218	-7.829388614	0.103425799
52	H52	-1.606763854	-9.447040555	-0.213480694
53	H53	-1.810757125	-8.901142644	1.446279511
54	H54	0.380676232	-2.591102552	-2.463433759
55	H55	0.635692817	3.846592917	3.114002689
56	H56	1.422085897	4.693129382	1.786092221
57	H57	-1.628472729	4.268349660	2.087110117
58	H58	1.174996004	6.403962427	0.699285300
59	H59	-0.019578865	7.389689383	-0.152357315
60	H60	0.375601739	7.759411071	1.522054611
61	H61	-2.354300139	7.591054140	1.620261516
62	H62	-3.053652591	5.965644375	1.480607057
63	H63	-2.481628634	6.826938669	0.038715830

6 lowest-energy conformers used for Boltzmann-averaged ^{13}C NMR data.
Avg. Energy: 1461.232862 au.

VIII.1b. Garcinone B (A23-6).[23]

No	type	δcal	δexp	diff
C1	C	162.3 (-2.9)	158	4.3 (-2.9)
C2	C	105.5	104.4	1.1
C3	C	164.3	159.8	4.5
C4	CH	93.9	94.3	-0.4
C4a	C	156.5	156.5	0.0

C5	CH	102.6	102.4	0.2
C6	C	152.1	150.9	1.2
C7	C	135.9	136.9	-1.0
C8	C	124.0 (-2.9)	119.7	4.3 (-2.9)
C8a	C	109.8	108.6	1.2
C9	C	184.6	182.5	2.1
C9a	C	104.4	103.9	0.5
C10a	C	153.9	153.1	0.8
C1'	CH2	22.7	22.6	0.1
C2'	CH	126.6	121.5	5.1
C3'	C	136.3	132.6	3.7
C4'	CH3	16.8	17.9	-1.1
C5'	CH3	25.5	25.8	-0.3
C1''	CH	125.3 (-3.7)	121	4.3 (-3.7)
C2''	CH	131.1 (+2.8)	132.3	-1.2 (+2.8)
C3''	C	76.7	78	-1.3
C4''[2]	CH3	26.5	27.4	-0.9
RMSD		2.39		
Max abs		5.06		
RMSD+CFx		1.88		
Max abs+CFx		5.06		

mol2 coordinates for lowest energy conformer in **SI: V12**
2 lowest-energy conformers used for Boltzmann-averaged ^{13}C NMR data.
Avg. Energy: -1341.111126 au.

VIII.2. Globulixanthone B (R2–A24)[95] and its revised structure (A23-7).

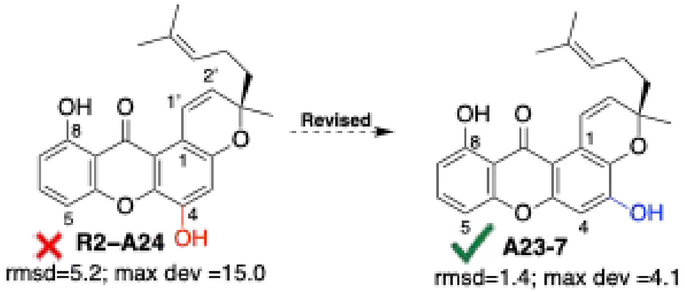

VIII.2a. Globulixanthone B (R2–A24)[95]

No	type	δcal	δexp	diff
C1	C	112.8	109.1	3.7
C2	C	152.1	137.1	15
C3	CH	109.8	102.4	7.4
C4	C	145.8	153.4	-7.6
C4a	C	138.6	151.4	-12.8
C5	CH	103.3	106.3	-3
C6	CH	136.8	135.8	1
C7	CH	110.4	110.1	0.3
C8	C	164.5	161.9	2.6
C8a	C	110.1	108.6	1.5
C9	C	187.3	183.7	3.6
C9a	C	117.5	119.5	-2
C10a	C	155	155.7	-0.7
C1'	CH	122.7	123.6	-0.9
C2'	CH	127.3	131.7	-4.4
C3'	C	80.5	79.6	0.9
C4'	CH2	42.9	40.4	2.5
C5'	CH2	25.4	22.8	2.6
C6'	CH	125.9	121.2	4.7
C7'	C	132.3	132.2	0.1

C8'	CH3	25.2	25.6	-0.4
C9'	CH3	18.1	17.7	0.4
C10'	CH3	30.6	25.7	4.9
RMSD		5.24		
Max abs		14.96		

mol2 coordinates for lowest energy conformer

1	C1	2.151429232	1.294047687	1.424487585
2	C2	2.751970111	-0.245860113	-0.486791684
3	C3	-1.381828075	2.469876457	-3.796167986
4	O4	-0.306417167	-2.786559838	-0.459229283
5	O5	-0.396306382	-4.986928799	0.889609760
6	O6	-2.076722871	1.817036642	2.935341050
7	C7	-0.325774902	-0.359101535	-1.961323017
8	C8	-0.107071837	0.504585949	-2.953011601
9	C9	2.154577487	2.177357427	-0.885065321
10	C10	-1.597058177	-4.130565833	4.187589065
11	C11	-1.130300684	-4.876467513	3.112864398
12	C12	-1.792502018	-2.756915596	4.096945652
13	C13	-1.407573479	2.065158577	0.679090303
14	C14	2.267907788	2.208047520	-2.383033457
15	C15	1.017916557	2.755477838	-3.089269657
16	O16	-0.751316205	2.373564588	-1.512819263
17	O17	-1.698953111	-0.796487164	2.826641931
18	C18	2.349452736	1.141820263	-0.061887086
19	C19	-0.845352046	-4.246301483	1.901764706
20	C20	-1.650734729	1.267545292	1.776981619
21	C21	-0.970629938	1.492929931	-0.517980839
22	C22	-0.768608580	0.115979166	-0.650545968
23	C23	-0.749318343	-2.157560710	0.514354360
24	C24	-1.508199699	-2.136552591	2.888725448
25	C25	-1.438757838	-0.114238003	1.670845655
26	C26	-1.037699600	-2.842384145	1.770983640
27	C27	-0.994044371	-0.703344561	0.485029520
28	C28	-0.294019140	1.989176277	-2.829969106
29	H29	1.826970265	2.302898518	1.694440237
30	H30	3.075923212	1.069511487	1.971922965
31	H31	1.392113101	0.586862796	1.783012013
32	H32	3.692127792	-0.535033360	-0.000038046
33	H33	2.883740177	-0.351188014	-1.564783662
34	H34	1.994482390	-0.976062053	-0.175225682
35	H35	-2.332841062	1.984828284	-3.558215569
36	H36	-1.115880846	2.227069060	-4.830037847
37	H37	-1.508235849	3.554099098	-3.711906192
38	H38	-0.243021684	-4.375187566	0.129448933
39	H39	-2.179112704	1.105832702	3.583379921
40	H40	-0.179016261	-1.419495606	-2.103703868
41	H41	0.221203136	0.152468655	-3.927840062
42	H42	1.853699665	3.125451009	-0.436539873
43	H43	-1.813849600	-4.633152843	5.125289639
44	H44	-0.976737483	-5.946737163	3.187105012
45	H45	-2.152943193	-2.168987037	4.932499807
46	H46	-1.550897017	3.137265945	0.741673261
47	H47	2.496463775	1.214555518	-2.780116537
48	H48	3.110143046	2.854346932	-2.668504211
49	H49	0.841426269	3.797872447	-2.794560838
50	H50	1.200178152	2.759445464	-4.171459821

3 lowest-energy conformers used for Boltzmann-averaged ^{13}C NMR data.
Avg. Energy: -1265.870115 au

VIII.2b. A23-7 (Unreported natural product)

No	type	δcal	δexp	diff
C1	C	121.2 (-2.9)	119.5	1.7 (-2.9)
C2	C	137.4	137.1	0.3
C3	C	152	151.4*	0.6
C4	CH	102.3	102.4	-0.1
C4a	C	153.7	153.4*	0.3
C5	CH	104.2	106.3	-2.1
C6	CH	136.1	135.8	0.3
C7	CH	109.9	110.1	-0.2
C8	C	164 (-2.9)	161.9	2.1 (-2.9)
C8a	C	110.1	108.6	1.5
C9	C	185.8	183.7	2.1
C9a	C	110	109.1	0.9
C10a	C	155.9	155.7	0.2
C1'	CH	124.5 (-3.7)	121.2	3.3 (-3.7)
C2'	CH	129.2 (+2.8)	131.7	-2.5 (+2.8)
C3'	C	80.9	79.6	1.3
C4'	CH2	42	40.4	1.6
C5'	CH2	23.7	22.8	0.9
C6'	CH	127.1	124.5	2.6
C7'	C	133.1	132.2	0.9
C8'	CH3	25.8	25.6	0.2
C9'	CH3	16.9	17.7	-0.8
C10'	CH3	29.8	25.7	4.1
RMSD		1.70		
Max abs		4.13		
RMSD+CFx		1.40		
Max abs+CFx		4.13		

mol2 coordinates for lowest energy conformer

1	C1	2.345518419	2.595072894	1.636590863
2	C2	2.384512625	0.280680741	0.613503424
3	C3	-1.354679175	2.522973135	-3.786641163
4	O4	-0.903063494	-3.081779431	-0.715710855
5	O5	-1.066329406	-5.317626438	0.571084335
6	C6	-0.751291466	-0.591760571	-2.102243992
7	C7	-0.520860636	0.289149869	-3.077286269
8	C8	2.160950210	2.343201857	-0.819121438
9	C9	-1.448527319	-4.470500169	4.065986042
10	C10	-1.324829653	-5.220930253	2.902516472
11	C11	-1.436916395	-3.080945579	4.046971402
12	C12	-0.973777730	1.807002772	0.754549371
13	C13	2.122976494	1.682489755	-2.170549511
14	C14	1.054521589	2.251500191	-3.113808773
15	O15	-0.784097539	2.147503182	-1.526112363
16	O16	-1.286314979	-1.091329799	2.835033043
17	C17	2.286122897	1.765193400	0.380283020
18	C18	-1.184183635	-4.577195971	1.673924653
19	C19	-1.121164984	0.997763273	1.860908759
20	C20	-0.875598408	1.230523491	-0.527847224
21	C21	-0.887467460	-0.136995039	-0.714356395
22	C22	-1.023630032	-2.450761836	0.349196057
23	C23	-1.297021054	-2.446290718	2.819890116
24	C24	-1.147750550	-0.385303605	1.682923083
25	C25	-1.169557219	-3.156531513	1.618380237
26	C26	-1.025003189	-0.988050666	0.422756235
27	C27	-0.388583428	1.774004246	-2.866856095
28	H28	2.281082962	3.666132729	1.425263682
29	H29	3.278058413	2.407243096	2.184223377

30	H30	1.520704173	2.331791282	2.310311976
31	H31	3.387263273	0.016375466	0.973563655
32	H32	2.179362852	-0.315724428	-0.277809345
33	H33	1.676716335	-0.026793280	1.392305490
34	H34	-2.379860200	2.185939178	-3.611771380
35	H35	-1.100514613	2.344822660	-4.836319512
36	H36	-1.299689658	3.599027925	-3.593846023
37	H37	-0.975700647	-4.688967341	-0.186335093
38	H38	-0.835795289	-1.649065607	-2.306830058
39	H39	-0.412011982	-0.047102265	-4.105337709
40	H40	2.114966280	3.434104992	-0.849781508
41	H41	-1.557177115	-4.983905117	5.016676434
42	H42	-1.333736412	-6.304640082	2.921107991
43	H43	-1.531345817	-2.487924457	4.948635585
44	H44	1.992990312	0.598626902	-2.086193212
45	H45	3.098397640	1.833243992	-2.654178246
46	H46	1.062762886	3.348743474	-3.065063617
47	H47	1.305087367	1.983770261	-4.148344961
48	O48	-0.918138548	3.142309763	0.883295629
49	H49	-0.734006743	3.498020866	-0.001216148
50	H50	-1.203369953	1.422912773	2.853679961

4 lowest-energy conformers used for Boltzmann-averaged ^{13}C NMR data.
Avg. Energy: -1265.877172 au

VIII.3. Cudracuspixanthone G (R3–A24)[96] and its revised structure (A23-10–B34-9).

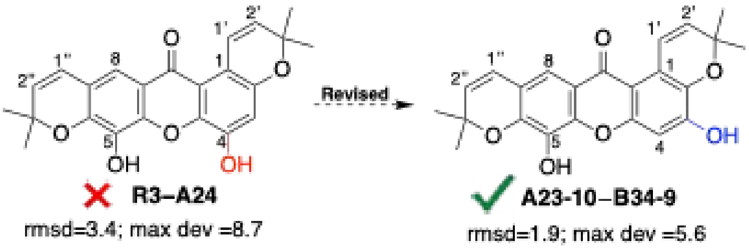

VIII.3a. Cudracuspixanthone G (R3–A24)[96]

No	type	δcal	δexp	diff
C1	C	114.6	116.7*	-2.1
C2	C	151	151.9	-0.9
C3	CH	109.7	102.8	6.9
C4	C	146.7	153.1	-6.4
C4a	C	140.1	137.7	2.4
C5	C	134.2	133.1	1.1
C6	C	143.5	144.4	-0.9
C7	C	117.9	118	-0.1
C8	CH	118	113	5.0
C8a	C	119.5	120.1*	-0.6
C9	C	179.9	176.6	3.3
C9a	C	118.7	127.4	-8.7
C10a	C	144.3	145.4	-1.1
C1'	CH	124.3	121	3.3
C2'	CH	128.4	132.3	-3.9
C3'	C	75.8	75.7	0.1
C4'[2]	CH3	27.1	26.3	0.8
C1''	CH	125.3	121.6	3.7
C2''	CH	129.1	131.2	-2.1
C3''	C	78.3	77.5	0.8
C4''[2]	CH3	27.4	27.4	0.0
RMSD		3.37		
Max abs		8.66		

mol2 coordinates for lowest energy conformer

1	C1	1.020500028	6.522169285	2.499696567
2	C2	-1.219534272	5.365193172	2.676680530
3	O3	-0.954200781	-0.093383297	-2.232913468
4	C4	0.440239981	-3.927458640	0.866568484
5	C5	-0.083751887	-3.952207264	-0.427278450
6	C6	0.656622384	-2.717255738	1.493526237
7	C7	-0.424217903	-2.782073208	-1.117680030
8	C8	-0.402265075	2.246736359	-0.776340056
9	C9	0.692527053	1.913484674	1.785733558
10	C10	-0.291689895	4.745456668	-0.444834479
11	C11	-0.172433315	5.740703680	0.435875709
12	O12	0.816925172	4.220562649	2.055962275
13	O13	0.631279626	-0.407427087	1.529105333
14	C14	-0.104326901	3.362273839	-0.012206572
15	C15	0.442446886	3.176634159	1.270018152
16	C16	-0.485425869	-0.225186498	-1.111035614
17	C17	0.346872587	-1.532935825	0.806531017
18	C18	0.380105677	0.805853573	0.991226209
19	C19	-0.191195930	-1.536644793	-0.480900848
20	C20	-0.167101186	0.957416086	-0.283443407
21	C21	0.096030082	5.469679860	1.900297855
22	O22	-0.299164231	-5.179429754	-0.958622460
23	C23	-0.272139457	-5.305741557	-2.396988196
24	C24	-1.018064959	-4.152352225	-3.021069588
25	C25	-1.056242371	-2.956925581	-2.427516007
26	O26	1.174312400	-2.675886384	2.740284842
27	O27	1.227085839	1.734805227	3.016948318
28	C28	-0.956868577	-6.637699087	-2.679424802
29	C29	1.189532465	-5.321830575	-2.857185290
30	H30	1.241753343	6.280914653	3.543900286
31	H31	1.959344397	6.566231386	1.940955903
32	H32	0.542908565	7.506334103	2.465521556
33	H33	-1.021533816	5.168950543	3.735779468
34	H34	-1.784839510	6.298902155	2.592511713
35	H35	-1.835990042	4.555489759	2.273433131
36	H36	0.681598113	-4.855697515	1.371078697
37	H37	-0.821130959	2.342502214	-1.772899861
38	H38	-0.519399537	4.934964275	-1.489911578
39	H39	-0.307651660	6.778744499	0.144826778
40	H40	-1.494001566	-4.319297512	-3.983635143
41	H41	-1.552347922	-2.110433492	-2.880826170
42	H42	1.264564395	-1.746436470	2.998459538
43	H43	1.353877029	2.619892728	3.390696487
44	H44	-0.951202092	-6.843880905	-3.754627750
45	H45	-1.993288724	-6.613912223	-2.330297516
46	H46	-0.431059367	-7.447927848	-2.165419948
47	H47	1.726593251	-6.151051762	-2.384610471
48	H48	1.243265914	-5.439095102	-3.944510684
49	H49	1.682682619	-4.381725202	-2.591440258

1 lowest-energy conformers used for Boltzmann-averaged ^{13}C NMR data.
Avg. Energy: -1339.405621 au

VIII.3b. A23-10–B34-9 (Unreported natural product)

C	Type	Theory	Expt.	diff.
C1	C	125.1 (-2.9)	127.4*	-2.3 (-2.9)
C2	C	135.7	137.7*	-2
C3	C	151.6	151.9*	-0.3
C4	CH	103	102.8*	0.2

C4a	C	154.2	153.1*	1.1
C5	C	134.7	133.1	1.6
C6	C	143.2	144.4	-1.2
C7	C	117.4	118	-0.6
C8	CH	117.1 (-2.9)	113	4.1 (-2.9)
C8a	C	119.5	120.1*	-0.6
C9	C	179.2	176.6	2.6
C9a	C	111.1	116.7*	-5.6
C10a	C	145.2	145.4	-0.2
C1'	CH	125.5 (-3.7)	121	4.5 (-3.7)
C2'	CH	130.8 (+2.8)	132.3	-1.5 (+2.8)
C3'	C	76.8	75.7	1.1
C4'[2]	CH3	26.6	26.3	0.3
C1''	CH	125.5 (-3.7)	121.6	3.9 (-3.7)
C2''	CH	128.6 (+2.8)	131.2	-2.6 (+2.8)
C3''	C	78	77.5	0.5
C4''[2]	CH3	27.5	27.4	0.1
RMSD		2.31		
Max abs		5.62		
RMSD+CFx		1.88		
Max abs+CFx		5.62		

mol2 coordinates for lowest energy conformer

1	C1	1.062673975	6.606323511	2.508342343
2	C2	-1.189445705	5.483144349	2.736912379
3	O3	-1.030108371	-0.024326117	-2.146418832
4	C4	0.494177545	-3.840336708	0.901781360
5	C5	-0.066776161	-3.856164837	-0.388222830
6	C6	0.712310613	-2.634544724	1.535273544
7	C7	-0.427192968	-2.692966744	-1.046071241
8	C8	-0.449987776	2.324066025	-0.712743790
9	C9	0.692111315	1.996849051	1.829213772
10	C10	-0.325874095	4.822391290	-0.395812831
11	C11	-0.179863234	5.822818762	0.474632667
12	O12	0.819128090	4.304102588	2.091911318
13	O13	0.643109495	-0.326018102	1.580640373
14	C14	-0.138771175	3.441441096	0.041792939
15	C15	0.430685084	3.258049970	1.313395080
16	C16	-0.527997048	-0.147309369	-1.034581223
17	C17	0.369636646	-1.455642674	0.873293887
18	C18	0.371497571	0.884080785	1.043633550
19	C19	-0.200089198	-1.440750055	-0.408809969
20	C20	-0.202067988	1.038425569	-0.219171672
21	C21	0.113486629	5.560476009	1.935860797
22	O22	-0.278328499	-5.102383464	-0.904767142
23	C23	-0.236464005	-5.223900832	-2.346143429
24	C24	-0.986356885	-4.066204084	-2.961375961
25	C25	-1.053639091	-2.875293448	-2.361159229
26	O26	1.249728749	1.832432932	3.050932858
27	C27	-0.918417848	-6.553405886	-2.643936249
28	C28	1.226782691	-5.229330295	-2.798809623
29	H29	1.296121515	6.374861943	3.551476042
30	H30	1.993589814	6.629764774	1.936095020
31	H31	0.599499628	7.596823742	2.469174306
32	H32	-0.975657409	5.293375319	3.793962655
33	H33	-1.742687687	6.424122518	2.653976978
34	H34	-1.824205132	4.679067348	2.352787241
35	H35	-0.888202695	2.414721149	-1.701091108
36	H36	-0.572014089	5.007544740	-1.437563348
37	H37	-0.308844188	6.859240732	0.176008941

38	H38	-1.441496966	-4.228936039	-3.934531358
39	H39	-1.552725298	-2.030929432	-2.814720049
40	H40	1.376675646	2.721847172	3.413280667
41	H41	-0.907196947	-6.748341897	-3.721347724
42	H42	-1.956524349	-6.533534671	-2.300836992
43	H43	-0.394405951	-7.370250602	-2.138575904
44	H44	1.764513865	-6.064334724	-2.337213338
45	H45	1.285322794	-5.332669880	-3.886822586
46	H46	1.717521867	-4.292200439	-2.519554508
47	O47	0.812828261	-5.003002896	1.500569504
48	H48	0.590177586	-5.708413004	0.872101496
49	H49	1.153761381	-2.594780453	2.523231218

2 lowest-energy conformers used for Boltzmann-averaged ^{13}C NMR data.
Avg. Energy: -1339.890692 au

VIII.4. Inophinone (R4–B13)[69] and its revised structure calophinone (A23-14–B31-20).[26]

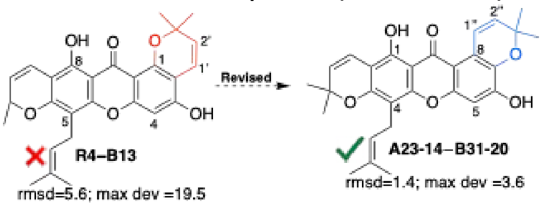

VIII.4a. Inophinone (R4–B13).[69]

No	type	δcal	δexp	diff
C1	C	158.8	150.7	8.1
C2	C	106	119.6	-13.6
C3	C	156.2	136.7	19.5
C4	CH	93.8	102.3	-8.5
C4a	C	158.6	152.9	5.7
C5	C	106	106.5	-0.5
C6	C	158.6	157.3	1.3
C7	C	106.1	104.1	2
C8	C	159.2	155.7	3.5
C8a	C	105	103.6	1.4
C9	C	181.8	182.7	-0.9
C9a	C	107.9	108.3	-0.4
C10a	C	153.9	153.7	0.2
C1'	CH	119.1	120.9	-1.8
C2'	CH	125.7	132.2	-6.5
C3'	C	78.1	77.1	1
C4'2	CH3	27.3	27.2	0.1
C1''	CH2	23.2	21.2	2
C2''	CH	125.7	122.2	3.5
C3''	C	129.5	132.3	-2.8
C4''	CH3	19.1	17.9	1.2
C5''	CH3	25.6	25.7	-0.1
C1'''	CH	121.3	115.9	5.4
C2'''	CH	123.9	126.9	-3
C3'''	C	77.8	77.7	0.1
C4'''2	CH3	27.8	28.2	-0.4
RMSD		5.56		
Max abs		19.48		

mol2 coordinates for lowest energy conformer

1	C1	5.822351974	-1.281563264	1.428651435
2	C2	4.768216544	0.985951579	1.075973791
3	O3	-1.072093187	-0.111018063	-3.182717686
4	C4	3.863585990	-0.668439089	-1.623567499
5	C5	4.931395779	-0.639047751	-0.824394796

6	C6	-4.689683522	-0.370896024	0.407273653
7	C7	-3.447949852	-0.543627233	1.001824187
8	C8	1.172846309	-0.892534986	1.000978052
9	C9	1.370424494	-0.452131057	-1.798399571
10	O10	3.502368016	-1.062609106	1.095091019
11	O11	-1.148843130	-0.707674507	0.862895564
12	C12	2.528891112	-0.629629113	-1.039213464
13	C13	-4.815954757	-0.181958716	-0.976747472
14	C14	2.405314948	-0.841435510	0.342072260
15	C15	-3.656788493	-0.167242961	-1.759916144
16	C16	-1.119943796	-0.298928663	-1.958335057
17	C17	-2.314261910	-0.532993227	0.197005313
18	C18	0.047278523	-0.698190115	0.209884852
19	C19	0.097924655	-0.482474732	-1.175998841
20	C20	-2.370165339	-0.345008853	-1.192649524
21	C21	4.763876432	-0.491083449	0.670280258
22	O22	1.494781564	-0.259532231	-3.112440039
23	C23	1.102746127	-1.071735018	2.499844776
24	C24	3.470097288	-0.474673354	4.452060042
25	C25	2.398447443	0.500864086	4.047815794
26	C26	1.388899837	0.224832098	3.216007747
27	C27	2.535135318	1.867020859	4.671480483
28	O28	-5.836454407	-0.384503365	1.125550780
29	O29	-3.746466929	-0.041866643	-3.087841150
30	C30	-4.882577388	0.650093067	-3.661501036
31	C31	-6.149564322	0.263790075	-2.937875585
32	C32	-6.105540031	-0.092098706	-1.653601659
33	C33	-4.620221194	2.154159409	-3.545442968
34	C34	-4.900620959	0.198956574	-5.116757680
35	H35	5.778499593	-2.338382054	1.151038120
36	H36	5.663310378	-1.192706026	2.506907988
37	H37	6.819303446	-0.897002460	1.190971954
38	H38	3.962800000	1.521604587	0.564030526
39	H39	4.620764636	1.082211276	2.156420187
40	H40	5.719837204	1.451675278	0.800286610
41	H41	3.950774411	-0.737483980	-2.702403237
42	H42	5.943918379	-0.682515894	-1.215710495
43	H43	-3.334302435	-0.686791832	2.071371748
44	H44	0.573415129	-0.163375654	-3.468849332
45	H45	1.800648351	-1.856447983	2.802188788
46	H46	0.098590507	-1.417873550	2.767664872
47	H47	3.357847911	-0.759389942	5.506266348
48	H48	3.471739049	-1.382383363	3.845786491
49	H49	4.460782198	-0.011068358	4.358430729
50	H50	0.677032568	1.020702304	2.996436256
51	H51	3.477744987	2.341935547	4.366813010
52	H52	2.557873124	1.798987812	5.766483768
53	H53	1.712897544	2.530736754	4.387710095
54	H54	-5.631412061	-0.555293287	2.052652901
55	H55	-7.084614856	0.315541274	-3.488062692
56	H56	-7.000506574	-0.346816156	-1.096697252
57	H57	-5.434582342	2.717601393	-4.012544500
58	H58	-4.559064012	2.450455544	-2.493516886
59	H59	-3.678433832	2.408421736	-4.041456050
60	H60	-5.075433691	-0.878666852	-5.178761717
61	H61	-3.940937053	0.425551612	-5.589303536
62	H62	-5.695945697	0.718000267	-5.661444531

10 lowest-energy conformers used for Boltzmann-averaged ^{13}C NMR data.
Avg. Energy: -1535.258390 au

VIII.4b. Calophinone (A23-14–B31-20).[26]

No	type	δcal	δexp	diff
C1	C	158.9 (-2.9)	155.7	3.2 (-2.9)
C2	C	106.0	104.1	1.9
C3	C	159.5	157.3	2.2
C4	C	106.6	106.5	0.1
C4a	C	154.3	153.7	0.6
C5	CH	102.5	102.3	0.2
C6	C	152.2	150.7	1.5
C7	C	136	136.7	-0.7
C8	C	124.1 (-2.9)	119.6	4.5 (-2.9)
C8a	C	109.7	108.3	1.4
C9	C	185.1	182.7	2.4
C9a	C	105.2	103.6	1.6
C10a	C	153.7	152.9	0.8
C1'	CH	121.2 (-3.7)	115.9	5.3 (-3.7)
C2'	CH	124.3 (+2.8)	126.9	-2.6 (+2.8)
C3'	C	78	77.7	0.3
C4'[2]	CH3	27.8	28.2	-0.4
C1''	CH2	23.2	21.2	2
C2''	CH	125.8	122.2	3.6
C3''	C	129.9	132.3	-2.4
C4''	CH3	19.1	17.9	1.2
C5''	CH3	25.7	25.7	0
C1'''	CH	125.2 (-3.7)	120.9	4.3 (-3.7)
C2'''	CH	131.3 (+2.8)	132.2	-0.9 (+2.8)
C3'''	C	76.8	77.2	-0.4
C4'''[2]	CH3	26.5	27.2	-0.7
RMSD		2.17		
Max abs		5.27		
RMSD+CFx		1.43		
Max abs+CFx		3.59		

mol2 coordinates for lowest energy conformer

1	C1	-1.609593640	-5.954447035	1.240809662
2	C2	0.758246369	-5.078074506	1.182625707
3	O3	0.610734134	0.759565484	-3.238989246
4	C4	-0.467526032	-4.100383575	-1.720072133
5	C5	-0.612392830	-5.153042989	-0.913952442
6	C6	0.172506917	4.443101487	0.267576209
7	C7	-0.166086928	3.232029928	0.836485500
8	C8	-0.838518820	-1.354002446	0.831313873
9	C9	-0.062345240	-1.631752988	-1.888663606
10	O10	-1.173969020	-3.663622322	0.923410684
11	O11	-0.474504691	0.945253550	0.695555930
12	C12	-0.412208889	-2.760477979	-1.148680801
13	C13	0.560630853	4.493034045	-1.084147955
14	C14	-0.786247764	-2.597741941	0.195265854
15	C15	0.629681873	3.356518372	-1.871580529
16	C16	0.294621111	0.836500955	-2.036698058
17	C17	-0.114095111	2.083046942	0.048066774
18	C18	-0.463585276	-0.256758773	0.064925188
19	C19	-0.076759938	-0.348033542	-1.283096043
20	C20	0.274031203	2.102802114	-1.298624201
21	C21	-0.648000727	-4.970720459	0.585421597
22	O22	0.280340975	-1.791196667	-3.168185751
23	C23	-1.201423231	-1.248781119	2.294641757
24	C24	-1.004405388	-3.624506161	4.317431554
25	C25	0.082070678	-2.619984124	4.031297163
26	C26	-0.025081901	-1.605120601	3.168258809

27	C27	1.349240969	-2.830134145	4.820694361
28	O28	0.136164025	5.577339844	0.989151394
29	C29	1.109364332	3.564101506	-3.242813812
30	C30	1.168925685	4.803258094	-3.737110888
31	C31	0.705959038	5.999248039	-2.937807137
32	O32	0.917990437	5.731761507	-1.531810585
33	H33	-2.614504554	-5.837478712	0.825737578
34	H34	-1.650690190	-5.783370932	2.320323656
35	H35	-1.273436466	-6.980955912	1.063549199
36	H36	1.416760191	-4.324101158	0.740296381
37	H37	0.722179808	-4.919429671	2.264971080
38	H38	1.182139076	-6.066775409	0.978750771
39	H39	-0.403833233	-4.203279360	-2.797738083
40	H40	-0.673027602	-6.167352205	-1.298466349
41	H41	-0.476931823	3.170842975	1.872082343
42	H42	0.478244216	-0.886664859	-3.520438004
43	H43	-2.060043934	-1.891744845	2.499769928
44	H44	-1.513049697	-0.221872851	2.510855428
45	H45	-1.427480041	-3.463992616	5.318044485
46	H46	-1.817012933	-3.595415286	3.589910831
47	H47	-0.590179270	-4.640905263	4.313549499
48	H48	0.834543433	-0.944883205	3.046636589
49	H49	1.795661586	-3.806334800	4.588711641
50	H50	1.145458428	-2.825951855	5.899576404
51	H51	2.094721505	-2.057593387	4.611957476
52	H52	0.394842795	6.296440042	0.390359834
53	H53	1.524271137	4.985479537	-4.747667373
54	H54	1.403637104	2.701613209	-3.823045957
55	C55	1.551834958	7.230614849	-3.233690845
56	H56	2.608023281	7.022581958	-3.042310252
57	H57	1.237157862	8.068283844	-2.603798495
58	H58	1.434588359	7.522868177	-4.281724646
59	C59	-0.782322366	6.270273206	-3.172426915
60	H60	-0.964528607	6.481123413	-4.230557135
61	H61	-1.111435800	7.131809432	-2.581334854
62	H62	-1.379350393	5.397391193	-2.892583045

6 lowest-energy conformers used for Boltzmann-averaged ^{13}C NMR data.
Avg. Energy: -1535.262702 au

VIII.5. 3-(1,1-Dimethyl-2-propen-1-yl)-5,9,11-trihydroxy-2,2-dimethyl-2H,12H-pyrano[2,3-a]xanthen-12-one (R5–B13),[70] and its revised structure calotetrapterin D (A23-15).[71]

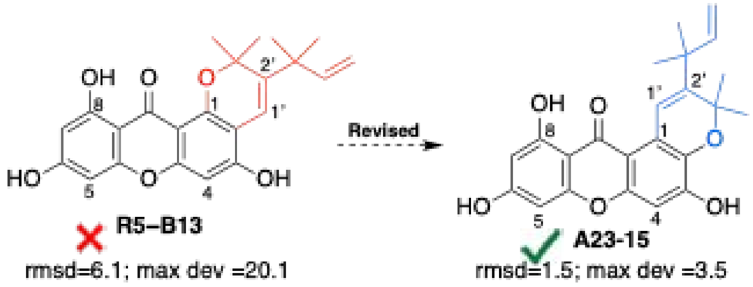

VIII.5a. 3-(1,1-Dimethyl-2-propen-1-yl)-5,9,11-trihydroxy-2,2-dimethyl-2H,12H-pyrano[2,3-a]xanthen-12-one (R5–B13)[70]

No	type	δcal	δexp	diff
C1	C	157.9	137.8	20.1
C1´	CH	116.1	118.7	-2.6
C2	C	107.6	122.8	-15.2
C3	C	156.2	153.6	2.6

C4	CH	93.8	103	-9.2
C4a	C	158.4	155.6	2.8
C5	CH	91.7	93.9	-2.2
C6	C	161.3	165.4	-4.1
C7	CH	96.5	98.7	-2.2
C8	C	166.0	164.7	1.3
C8a	C	104.7	103.9	0.8
C9a	C	107.7	108.3	-0.6
C10a	C	157.2	158.1	-0.9
C20	C	181.3	183.1	-1.8
C1'	C	41.5	42.7	-1.2
C2'	C	141.4	149.9	-8.5
C3'	C	82.3	80.4	1.9
C4'[2]	CH3	28.0	27.3	0.7
C2''[2]	CH3	28.0	28.6	-0.6
C4''	CH	146.0	147.9	-1.9
C5''	CH2	112.8	112.3	0.5
RMSD		6.10		
Max abs		20.06		

mol2 coordinates for lowest energy conformer

1	C1	2.216624570	-1.651885870	-1.229283491
2	C2	0.696027787	-0.038138344	-2.404427580
3	O3	2.039995358	1.748743238	1.196666048
4	C4	-1.487616105	-1.649591004	-0.691337603
5	C5	-0.362138885	-2.047022411	-1.303866635
6	C6	-2.755982689	0.119980795	0.566633141
7	C7	-2.762752798	1.244233318	1.381147202
8	C8	-0.766987937	4.716004197	3.711609233
9	C9	1.832390452	4.021800791	2.880691849
10	O10	0.790229102	-0.309143403	-0.015206548
11	O11	-1.656411060	2.931968813	2.509770910
12	C12	1.634794583	5.122259251	3.709579980
13	C13	-1.555870714	-0.411219343	0.070592542
14	C14	0.343488390	5.457825884	4.115782037
15	C15	-0.358474031	0.217415615	0.409346947
16	C16	0.912390379	2.079936368	1.591585328
17	C17	-1.550948581	1.841681124	1.710060821
18	C18	-0.550106390	3.625920584	2.888374331
19	C19	0.725584109	3.241663731	2.452313432
20	C20	-0.317206598	1.363275299	1.240684294
21	C21	0.813352396	-1.056225938	-1.262819800
22	O22	3.072708917	3.719292542	2.503638579
23	C23	-0.290299515	-3.375011141	-2.078040520
24	C24	1.251686551	-3.701645231	-4.091059688
25	C25	0.213393927	-3.127458978	-3.490089149
26	C26	0.555560957	-4.413838410	-1.316496450
27	C27	-1.695114956	-3.993256812	-2.257338270
28	O28	-3.896649573	-0.521353297	0.221994382
29	O29	0.103719389	6.518356687	4.919738752
30	H30	2.337382440	-2.300380350	-0.358793129
31	H31	2.932622056	-0.830763464	-1.137745172
32	H32	2.431681980	-2.209846345	-2.141748930
33	H33	1.456602132	0.738568731	-2.276879661
34	H34	0.852061164	-0.530713246	-3.367836143
35	H35	-0.292772978	0.430859550	-2.408995415
36	H36	-2.391341435	-2.244711185	-0.716010268
37	H37	-3.682376428	1.668153069	1.771046463
38	H38	-1.765213173	4.987240317	4.030114916
39	H39	2.498942612	5.700506777	4.022429497

40	H40	3.001640854	2.915694327	1.924153673
41	H41	1.490128718	-3.481828284	-5.127311046
42	H42	1.903251709	-4.406966647	-3.582499098
43	H43	-0.404655916	-2.433608113	-4.061866817
44	H44	0.489431419	-5.387848884	-1.813817952
45	H45	1.610418727	-4.145648269	-1.250390846
46	H46	0.170136390	-4.523599740	-0.298391844
47	H47	-2.383286728	-3.302254860	-2.754890021
48	H48	-2.130365577	-4.286069915	-1.295990151
49	H49	-1.612277558	-4.893184933	-2.874428904
50	H50	-4.647191376	-0.088852983	0.646382598
51	H51	0.939793935	6.950686390	5.133224178

16 lowest-energy conformers used for Boltzmann-averaged ^{13}C NMR data.
Avg. Energy: -1341.092293 au

VIII.5b. Calotetrapterin D (A23-15).[71]

No	type	δcal	δexp	diff
C1	C	125.4 (-2.9)	122.8	2.6 (-2.9)
C2	C	135.4	137.8	-2.4
C3	C	152	153.6	-1.6
C4	CH	102	103	-1
C4a	C	153.9	155.6	-1.7
C5	CH	91.7	93.9	-2.2
C6	C	161.9	165.4	-3.5
C7	CH	96.4	98.7	-2.3
C8	C	165.8 (-2.9)	164.7	1.1 (-2.9)
C8a	C	105.1	103.9	1.2
C9a	C	109.6	108.3	1.3
C10a	C	157.8	158.1	-0.3
C20	C	184.5	183.1	1.4
C1'	CH	122.4 (-3.7)	118.7	3.7 (-3.7)
C2'	C	147.3 (+2.8)	149.9	-2.6 (+2.8)
C3'	C	80.9	80.4	0.5
C4'[2]	CH3	27.6	27.3	0.3
C1''	C	42.2	42.7	-0.5
C2''	CH	146.0	147.9	-1.9
C3''	CH2	112.8	112.3	0.5
C4''[2]	CH3	28.2	28.6	-0.4
RMSD		1.79		
Max abs		3.72		
RMSD+CFx		1.45		
Max abs+CFx		3.46		

mol2 coordinates for lowest energy conformer

1	C1	0.172763046	3.924482045	-1.501335397
2	C2	-1.825513253	2.578151317	-0.799731201
3	O3	0.228978275	-2.505592433	-0.007001774
4	C4	0.412307905	0.258837221	-0.703471739
5	C5	0.239727724	1.315459412	-1.515258182
6	C6	-0.013948027	1.861488660	2.636868675
7	C7	-0.088133712	0.795487863	3.511538052
8	C8	-0.162284434	-3.702423908	4.620582155
9	C9	0.078511838	-4.554591077	1.946109159
10	O10	0.233065175	2.716878567	0.470756663
11	O11	-0.122560229	-1.477563322	3.939053319
12	C12	-0.004270979	-5.489551288	2.971715442
13	C13	0.120990101	1.615946812	1.259244755
14	C14	-0.122185506	-5.057255422	4.294238492
15	C15	0.194278228	0.339936644	0.739663610
16	C16	0.133793457	-2.167637153	1.189131087

17	C17	-0.031731493	-0.499900736	2.997039447
18	C18	-0.077692448	-2.784609827	3.587522999
19	C19	0.044180535	-3.163770034	2.241217961
20	C20	0.102036779	-0.770152543	1.627072355
21	C21	-0.293566089	2.612854616	-0.879455742
22	O22	0.190299947	-4.989667989	0.692533841
23	C23	0.472572362	1.186324831	-3.032677447
24	C24	-0.830511629	2.574955604	-4.737673808
25	C25	-0.763387775	1.645644984	-3.788385671
26	C26	1.749507164	1.934574308	-3.462461632
27	C27	0.665131872	-0.291022799	-3.444920376
28	O28	-0.067384461	3.128874597	3.082921892
29	O29	-0.204050998	-5.931652626	5.321427934
30	H30	1.261582792	4.002132734	-1.466412570
31	H31	-0.246499362	4.752129544	-0.920798993
32	H32	-0.173272798	4.018310916	-2.531329407
33	H33	-2.186566422	3.445930857	-0.237321076
34	H34	-2.253869585	2.614154247	-1.804912026
35	H35	-2.173502526	1.665764270	-0.305760975
36	H36	0.713271349	-0.703840086	-1.082729795
37	H37	-0.199606649	0.954529267	4.576858189
38	H38	-0.254795421	-3.378259766	5.648909956
39	H39	0.025784129	-6.544882799	2.718484785
40	H40	0.232557671	-4.182737104	0.116881338
41	H41	-1.763658141	2.785107330	-5.251548599
42	H42	0.034270534	3.157738906	-5.043213480
43	H43	-1.665538334	1.086162551	-3.536981193
44	H44	1.974150824	1.716140059	-4.512041801
45	H45	1.674392482	3.017377456	-3.359358091
46	H46	2.594045703	1.594211362	-2.856209854
47	H47	-0.175310351	-0.916731051	-3.129809237
48	H48	1.583472955	-0.709771953	-3.021682123
49	H49	0.742872953	-0.346664625	-4.534872901
50	H50	-0.011118694	3.701129453	2.300500305
51	H51	-0.163586487	-6.832437892	4.977082677

9 lowest-energy conformers used for Boltzmann-averaged ^{13}C NMR data.
Avg. Energy: -1341.096992 au

VIII.6. 3-(1,1-Dimethyl-2-propen-1-yl)-5,9,11-trihydroxy-2,2-dimethyl-10-(3-methyl-2-buten-1-yl)-2H,12H-pyrano[2,3-a]xanthen-12-one (R6–B13)[72] and its revised structure calotetrapterin A (A23-17).[73]

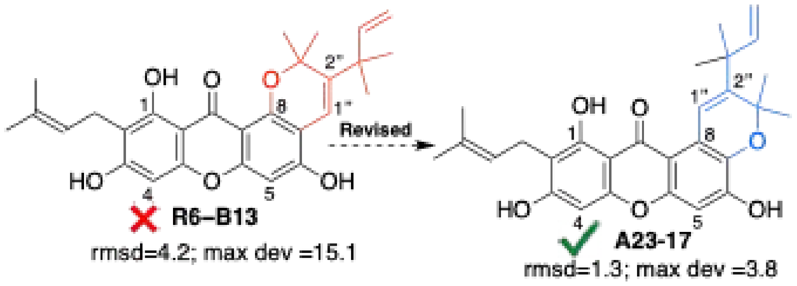

VIII.6a. 3-(1,1-Dimethyl-2-propen-1-yl)-5,9,11-trihydroxy-2,2-dimethyl-10-(3-methyl-2-buten-1-yl)-2H,12H-pyrano[2,3-a]xanthen-12-one (R6–B13)[72]

No	type	δcal	δexp	diff
C1	C	162.4	161.5	0.9
C2	C	106.1	110.9	-4.8
C3	C	163.4	162.9	0.5
C4	CH	93.8	93.2	0.6
C4a	C	155.8	155.9	-0.1
C5	CH	93.7	102.9	-9.2
C6	C	156.1	154.1	2
C7	C	107.5	108.4	-0.9

C8	C	157.9	149.7	8.2
C8a	C	107.7	122.8	-15.1
C9	C	181.5	183.1	-1.6
C9a	C	104.2	103.8	0.4
C10a	C	158.5	153.3	5.2
C1'	CH2	22.6	21.9	0.7
C2'	CH	125.8	123.4	2.4
C3'	C	137.1	131.4	5.7
C4'	CH3	17.1	17.8	-0.7
C5'	CH3	25.5	25.9	-0.4
C1''	CH	116.5	118.8	-2.3
C2''	C	140.9	137.6	3.3
C3''	C	82.2	80.3	1.9
C4''^{2}	CH3	28.0	27.3	0.7
C1'''	C	41.4	42.7	-1.3
C2'''	CH	146.1	147.9	-1.8
C3'''	CH2	112.8	112.2	0.6
C4'''^{2}	CH3	28.0	28.6	-0.6
RMSD		4.24		
Max abs		15.08		

mol2 coordinates for lowest energy conformer

1	O1	0.879451237	-1.070754482	-0.329697941
2	C2	-2.671026850	2.594967620	-0.257692594
3	C3	3.118733743	1.088871236	3.605368975
4	C4	-2.561661816	1.466858026	-1.084918870
5	C5	3.462297698	-0.064191018	2.878468981
6	C6	-1.742748038	2.844160596	0.742801596
7	C7	2.011177878	1.871983703	3.278559132
8	C8	-1.489474730	0.596883316	-0.889384497
9	C9	2.641133570	-0.435413320	1.810856073
10	O10	0.155122641	2.270609721	1.928898798
11	C11	0.633347846	-0.065376945	0.354049975
12	C12	-0.694907991	1.947461151	0.925034141
13	C13	1.225521337	1.479909071	2.212942815
14	C14	-0.523632584	0.806176002	0.125432901
15	C15	1.497367571	0.330825248	1.459419687
16	C16	-3.571926146	1.098061114	-2.065721246
17	C17	-3.365666756	0.127196261	-2.968571954
18	C18	-1.951336628	-0.477077802	-2.990026803
19	O19	-1.402974418	-0.499467436	-1.643748261
20	C20	-1.832143765	-1.944496518	-3.387585501
21	C21	-1.015845988	0.380476381	-3.852317954
22	O22	-3.726458929	3.413464740	-0.477499519
23	C23	-4.449856193	-0.278619489	-3.982093668
24	C24	-5.023396207	-1.672133599	-3.659925209
25	C25	-5.640154553	0.705650930	-3.948774354
26	C26	-3.895037052	-0.182751559	-5.393759078
27	O27	3.860177919	1.506632219	4.652824790
28	C28	4.707203000	-0.861238016	3.207278211
29	C29	4.747621358	-1.327179211	4.642354766
30	C30	5.730021290	-1.166105986	5.543008354
31	C31	5.595019252	-1.728179789	6.936544746
32	C32	7.039683177	-0.465859833	5.281667199
33	O33	2.956018789	-1.529607368	1.112293457
34	C34	-3.869160267	-1.137754819	-6.319268060
35	H35	-1.810410980	3.712401237	1.390061166
36	H36	1.771924896	2.755330727	3.856609959
37	H37	-4.509545373	1.635823392	-2.008476761
38	H38	-0.784070245	-2.238750730	-3.287820898

39	H39	-2.422061561	-2.574187476	-2.717790658
40	H40	-2.147410937	-2.101631820	-4.420322056
41	H41	-1.309853976	0.318825549	-4.904258018
42	H42	0.010430821	0.012519401	-3.749562598
43	H43	-1.049480947	1.429669836	-3.541267627
44	H44	-3.715441051	4.128692249	0.170222549
45	H45	-5.366902204	-1.695140613	-2.621098151
46	H46	-4.301236718	-2.477925206	-3.793702210
47	H47	-5.881233353	-1.882602581	-4.308205009
48	H48	-6.161522071	0.670321254	-2.986622418
49	H49	-6.355087165	0.425318667	-4.728082109
50	H50	-5.322290760	1.736551449	-4.135001511
51	H51	4.423760349	0.773480429	4.954164879
52	H52	5.595790303	-0.269646549	2.959050731
53	H53	4.727437534	-1.735010222	2.550663911
54	H54	3.851454224	-1.862428741	4.959937527
55	H55	4.632393913	-2.224186259	7.086451025
56	H56	5.697179943	-0.936920019	7.690217961
57	H57	6.390297987	-2.456756580	7.136009117
58	H58	7.236252171	0.280334479	6.060279330
59	H59	7.864794390	-1.186501029	5.320958812
60	H60	7.081847470	0.036495666	4.312735726
61	H61	2.262054301	-1.622140705	0.406259469
62	H62	-4.222568292	-2.146881257	-6.125386780
63	H63	-3.487390895	-0.940916986	-7.316719029
64	H64	-3.531601168	0.811882294	-5.656125420

28 lowest-energy conformers used for Boltzmann-averaged ^{13}C NMR data.
Avg. Energy: -1536.446708 au

VIII.6b. Calotetrapterin A (A23-17)[73]

No	type	δcal	δexp	diff
C1	C	162.1 (-2.9)	161.5	0.6 (-2.9)
C2	C	105.9	108.7	-2.8
C3	C	164.1	162.9	1.2
C4	CH	94	93.2	0.8
C4a	C	156.4	155.9	0.5
C5	CH	102	102.9	-0.9
C6	C	151.9	153.3	-1.4
C7	C	135.2	131.4*	3.8
C8	C	125.3 (-2.9)	122.8	2.5 (-2.9)
C8a	C	109.6	110.9	-1.3
C9	C	184.7	183.1	1.6
C9a	C	104.6	103.8	0.8
C10a	C	154	154.1	-0.1
C1'	CH2	22.6	21.9	0.7
C2'	CH	125.6	123.4	2.2
C3'	C	137.5	137.6*	-0.1
C4'	CH3	25.4	25.9	-0.5
C5'	CH3	17.1	17.8	-0.7
C1''	CH	122.7 (-3.7)	122.5	3.9 (-3.7)
C2''	C	147 (+2.8)	146.9	-2.7 (+2.8)
C3''	C	80.7	80.3	0.4
C4''^{2}	CH3	27.5	27.3	0.2
C1'''	C	42.2	42.7	-0.5
C2'''	CH	146.2	147.9	-1.7
C3'''	CH2	112.7	112.2	0.5
C4'''^{2}	CH3	28.2	28.6	-0.4
RMSD		1.60		
Max abs		3.87		
RMSD+CFx		1.30		

Max abs+CFx 3.82

mol2 coordinates for lowest energy conformer

1	O1	-0.392004647	-0.941487153	0.346938264
2	C2	0.197345029	3.318002098	-2.411752897
3	C3	3.122557775	0.698568791	3.521939652
4	C4	-0.622299070	2.192041388	-2.603990397
5	C5	2.373160165	-0.492913935	3.523957098
6	C6	1.038414906	3.365267938	-1.318363876
7	C7	2.956225251	1.682097926	2.547428513
8	C8	-0.613434732	1.115184214	-1.741855643
9	C9	1.449945605	-0.686543875	2.495965366
10	O10	1.902171481	2.436846823	0.614017879
11	C11	0.311135802	0.087030912	0.400892536
12	C12	1.045534547	2.289211704	-0.430341495
13	C13	2.031748259	1.460884880	1.545056534
14	C14	0.237109643	1.155594123	-0.599654173
15	C15	1.264648654	0.288767638	1.476073301
16	O16	-1.388467374	2.204223595	-3.726582532
17	C17	-2.661709490	1.509261232	-3.653724979
18	C18	-2.420810164	0.105977655	-3.066178348
19	C19	-1.463072892	-0.009440415	-2.130089903
20	O20	0.150388267	4.330628295	-3.296023505
21	O21	4.034983285	0.955266918	4.481145085
22	C22	2.526304347	-1.519895790	4.627031314
23	C23	3.951172532	-1.986726702	4.801531164
24	C24	4.686169203	-2.023332937	5.923717902
25	C25	6.102460909	-2.539681571	5.898687763
26	C26	4.205672245	-1.595371836	7.286847896
27	O27	0.728726354	-1.811718902	2.487988372
28	H28	1.675666142	4.222657145	-1.140583589
29	H29	3.549077205	2.587426755	2.571928023
30	H30	-0.512449412	4.085760610	-3.961903946
31	H31	4.258988906	0.125324546	4.936359180
32	H32	1.900434334	-2.380414531	4.372652471
33	H33	2.125373388	-1.114461914	5.563464813
34	H34	4.420075420	-2.330742669	3.878749792
35	H35	6.421207745	-2.811232136	4.888920195
36	H36	6.200552604	-3.425752841	6.538848308
37	H37	6.799666432	-1.788776751	6.290026280
38	H38	4.893498109	-0.859982083	7.722325559
39	H39	3.204043318	-1.161327224	7.281402243
40	H40	4.193328419	-2.456253370	7.967414353
41	H41	0.136146189	-1.761613296	1.693498637
42	H42	-1.279723762	-0.939803138	-1.617128278
43	C43	-3.295558291	-1.102648126	-3.449595523
44	C44	-2.939950631	-1.620252173	-4.856833818
45	H45	-3.467556254	-2.560238542	-5.052138731
46	H46	-1.864817317	-1.816028376	-4.913690594
47	H47	-3.199492561	-0.924183181	-5.655058368
48	C48	-3.075712829	-2.282631620	-2.475803803
49	H49	-3.771037039	-3.087571004	-2.732582148
50	H50	-3.255038827	-1.994587667	-1.435331504
51	H51	-2.058769396	-2.680540242	-2.551817037
52	C52	-4.765095700	-0.743076340	-3.306430106
53	H53	-5.042840260	-0.424892763	-2.300664743
54	C54	-5.716765656	-0.843608351	-4.230640942
55	H55	-6.750693228	-0.606715999	-3.997873454
56	H56	-5.509876389	-1.166495317	-5.247396084
57	C57	-3.143537496	1.577701146	-5.098218365

58 H58 -2.423533462 1.103253594 -5.768620530
59 H59 -3.232233246 2.630366550 -5.384367362
60 H60 -4.121009109 1.107334848 -5.210695815
61 C61 -3.599696762 2.313827007 -2.744714879
62 H62 -4.600927595 1.877063326 -2.759989281
63 H63 -3.666950978 3.346999492 -3.101493027
64 H64 -3.238867901 2.318371619 -1.712678820

16 lowest-energy conformers used for Boltzmann-averaged ^{13}C NMR data.
Avg. Energy: -1536.451322 au

VIII.7. 5,9,11-trihydroxy-10-(2-hydroxy-3-methylbut-3-en-1-yl)-3,3-dimethyl-2-(2-methylbut-3-en-2-yl)pyrano[3,2-a]xanthen-12(3H)-one (R7–B13)[74] and its revised structure calotetrapterin E (A23-16).[71]

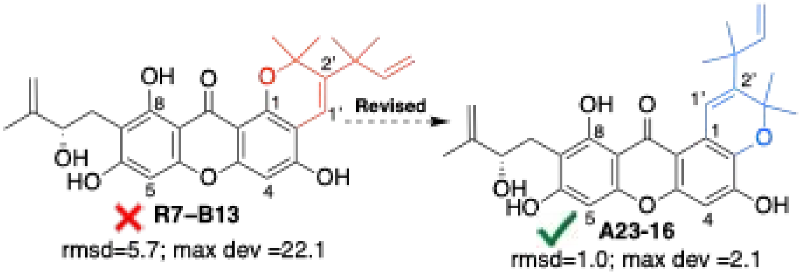

VIII.7a. 5,9,11-trihydroxy-10-(2-hydroxy-3-methylbut-3-en-1-yl)-3,3-dimethyl-2-(2-methylbut-3-en-2-yl)pyrano[3,2-*a*]xanthen-12(*3H*)-one (R7–B13) [74]

No	type	δcal	δexp	diff
C1	C	157.8	135.7	22.1
C2	C	107.3	121.7	-14.4
C3	C	156.1	150.6	5.5
C4	CH	93.8	101.8	-8
C4a	C	158.6	153.4	5.2
C5	CH	94.8	94.5	0.3
C6	C	164.2	163.2	1.0
C7	C	105.3	107.4	-2.1
C8	C	163.2	161.1	2.1
C8a	C	103.6	103.4	0.2
C9	C	181.4	182.5	-1.1
C9a	C	107.7	108.5	-0.8
C10a	C	155.7	155.8	-0.1
C1'	CH	116.4	118.0	-1.6
C2'	C	140.9	148.8	-7.9
C3'	C	82.0	80.6	1.4
C4'	CH3	27.9	27.4	0.5
C5'	CH3	28.3	27.4	0.9
C1''	C	41.4	42.7	-1.3
C2''	CH	146.2	146.8	-0.6
C3''	CH2	112.7	112.0	0.7
C4''	CH3	27.7	28.3	-0.6
C5''	CH3	28.3	28.3	0
C1'''	CH2	27.0	28.1	-1.1
C2'''	CH	77.2	77.5	-0.3
C3'''	C	147.7	146.7	1
C4'''	CH2	110.1	110.2	-0.1
C5'''	CH3	20.2	18.7	1.5
RMSD		5.68		
Max abs		22.12		

mol2 coordinates for lowest energy conformer
1 C1 -0.897478722 2.255338973 -2.281335442
2 C2 0.436006347 0.852608746 -3.877937643
3 O3 -0.150791496 -1.462593509 -0.452668306
4 C4 2.063679640 3.185695610 -1.448908969

5	C5	1.525544999	2.870867154	-2.636853133
6	C6	2.173054896	2.828283609	1.037092461
7	C7	1.971926360	2.016679596	2.144353516
8	C8	0.474542874	-1.815664016	4.280779212
9	C9	-0.497468031	-2.977136061	1.929233604
10	O10	0.853031874	0.747372383	-1.613342360
11	O11	1.212873192	0.054784371	3.103261880
12	C12	-0.630413015	-3.677925059	3.130072486
13	C13	1.780061014	2.417885801	-0.245765609
14	C14	-0.160146629	-3.061312368	4.305571473
15	C15	1.178300457	1.168668203	-0.390337361
16	C16	0.277562665	-0.990317291	0.613121283
17	C17	1.372653201	0.773652435	1.967051245
18	C18	0.607592466	-1.164303978	3.072789804
19	C19	0.127829005	-1.703568889	1.872425212
20	C20	0.952756802	0.307168494	0.710603107
21	C21	0.499423680	1.724610853	-2.628618705
22	O22	-0.961195724	-3.536795804	0.804086350
23	C23	1.852837469	3.672590841	-3.908641174
24	C24	0.181940445	3.933732237	-5.825625175
25	C25	0.567491678	4.141161512	-4.569635770
26	C26	2.623903460	4.967386458	-3.568651445
27	C27	2.751454256	2.859778734	-4.860043253
28	O28	2.763085672	4.042171753	1.136239159
29	O29	-0.278922321	-3.636971345	5.512670054
30	C30	-1.213744432	-5.070526571	3.112060451
31	C31	-2.606556577	-5.231634637	3.748256564
32	C32	-3.580703710	-4.158991544	3.313254847
33	O33	-2.405336763	-5.266735048	5.161935995
34	C34	-4.132290281	-4.320782821	1.923580291
35	C35	-3.881780731	-3.123766719	4.097491648
36	H36	-1.580108336	1.411235996	-2.139775015
37	H37	-1.276177377	2.884432430	-3.091720538
38	H38	-0.871065084	2.848826308	-1.361939745
39	H39	-0.287867554	0.053122797	-3.698675756
40	H40	1.406246145	0.389525380	-4.071350203
41	H41	0.118846171	1.430202663	-4.748029297
42	H42	2.750196866	4.015116386	-1.334584575
43	H43	2.268217931	2.320085564	3.143473124
44	H44	0.827720438	-1.367171867	5.200606824
45	H45	-0.762476436	-2.892840757	0.069231847
46	H46	-0.741860307	4.359599645	-6.205610701
47	H47	0.763139861	3.335012122	-6.521804191
48	H48	-0.059331941	4.758533740	-3.924046455
49	H49	2.770144683	5.545461427	-4.486108636
50	H50	2.075621833	5.592505588	-2.856614004
51	H51	3.611889053	4.748205769	-3.150421157
52	H52	2.256494589	1.980838170	-5.274227106
53	H53	3.642736747	2.523445623	-4.321653043
54	H54	3.074990551	3.487329803	-5.698120759
55	H55	3.012971677	4.200396129	2.053848528
56	H56	-0.978069154	-4.323468766	5.492747513
57	H57	-1.272713937	-5.400441612	2.072651731
58	H58	-0.546851351	-5.770886120	3.631033008
59	H59	-3.001251679	-6.210913553	3.437291211
60	H60	-3.255847056	-5.114254791	5.595408989
61	H61	-3.330159015	-4.207130226	1.185562828
62	H62	-4.896790591	-3.569708868	1.711083372
63	H63	-4.573913392	-5.314386538	1.782429587
64	H64	-3.419288334	-2.979149884	5.070430506

65 H65 -4.576169024 -2.354934658 3.77131581

12 lowest-energy conformers used for Boltzmann-averaged ^{13}C NMR data.
Avg. Energy: -1611.675668 au

VIII.7b. Calotetrapterin E (A23-16)[71]

No	type	δcal	δexp	diff
C1	C	125.3 (-2.9)	121.7	3.6 (-2.9)
C2	C	135.2	135.7	-0.5
C3	C	151.8	150.6	1.2
C4	CH	102.1	101.8	0.3
C4a	C	154.0	153.4	0.6
C5	CH	95	94.5	0.5
C6	C	164.8	163.2	1.6
C7	C	105.3	107.4	-2.1
C8	C	162.7 (-2.9)	161.1	1.6 (-2.9)
C8a	C	104.1	103.4	0.7
C9	C	184.5	182.5	2
C9a	C	109.6	108.5	1.1
C10a	C	156.4	155.8	0.6
C1'	CH	122.8 (-3.7)	118.0	4.8 (-3.7)
C2'	C	146.9 (+2.8)	148.8	-1.9 (+2.8)
C3'	C	80.7	80.6	0.1
C4'	CH3	26.8	27.4	-0.6
C5'	CH3	28.1	27.4	0.7
C1''	C	42.2	42.7	-0.5
C2''	CH	146.2	146.8	-0.6
C3''	CH2	112.6	112.0	0.6
C4''	CH3	28.1	28.3	-0.2
C5''	CH3	28.3	28.3	0
C1'''	CH2	27.2	28.1	-0.9
C2'''	CH	77.1	77.5	-0.4
C3'''	C	147.4	146.7	0.7
C4'''	CH3	20.1	18.7	1.4
C5'''	CH2	110.1	110.2	-0.1
RMSD		1.50		
Max abs		4.77		
RMSD+CFx		0.95		
Max abs+CFx		2.08		

mol2 coordinates for lowest energy conformer

1	C1	-4.246557377	2.413925001	-0.251093154
2	C2	-5.631067989	0.770325783	-1.543621995
3	O3	0.875691046	0.434783396	-0.514536177
4	C4	-1.916548915	0.370025510	-1.088835989
5	C5	-3.025173346	0.776528130	-1.730608494
6	C6	-3.314657517	-0.468173989	2.264477330
7	C7	-2.198330174	-0.657243447	3.054114853
8	C8	2.361342359	-0.754544286	3.877845154
9	C9	3.045704770	-0.001395624	1.270584656
10	O10	-4.299032596	0.032969424	0.198638172
11	O11	0.094681271	-0.661968650	3.353068462
12	C12	4.065745316	-0.220881606	2.196206925
13	C13	-3.153230518	-0.098178327	0.917860494
14	C14	3.698894498	-0.625970065	3.496562699
15	C15	-1.910934692	0.069007003	0.341910172
16	C16	0.617799326	0.088387886	0.657160909
17	C17	-0.936211161	-0.468439970	2.489802593
18	C18	1.377483444	-0.513410984	2.941008493
19	C19	1.674855869	-0.144681794	1.620767313

20	C20	-0.749545281	-0.104109338	1.147981628
21	C21	-4.283324895	1.012176510	-0.873574728
22	O22	3.377578046	0.364777855	0.024396578
23	C23	-2.991979817	1.089033836	-3.238487596
24	C24	-4.496872682	2.864778121	-4.296177364
25	C25	-3.508412829	2.496642731	-3.485530979
26	C26	-1.542751532	1.093616996	-3.775309586
27	C27	-3.758112590	0.020539302	-4.042144669
28	O28	-4.553095309	-0.630955698	2.764221071
29	O29	4.608312055	-0.894169765	4.445398310
30	C30	5.500118924	0.020994000	1.788832040
31	C31	6.391499038	-1.230458581	1.676040563
32	C32	5.711306591	-2.373405846	0.954314534
33	O33	6.790078399	-1.563400056	3.006457065
34	C34	5.612085120	-2.213355188	-0.537510046
35	C35	5.204150880	-3.417408280	1.609042173
36	H36	-5.079061568	2.531656661	0.450518832
37	H37	-4.343036108	3.171717670	-1.032184126
38	H38	-3.307449027	2.582730941	0.282804667
39	H39	-6.420147851	0.937395090	-0.803762656
40	H40	-5.708377947	-0.261043170	-1.894856138
41	H41	-5.788385923	1.458161910	-2.375165683
42	H42	-0.974730638	0.254061498	-1.600338547
43	H43	-2.292902912	-0.932029905	4.097407859
44	H44	2.109513428	-1.061997610	4.884756417
45	H45	2.523667729	0.483697063	-0.470887313
46	H46	-4.746382902	3.912320073	-4.435394702
47	H47	-5.091473021	2.145819452	-4.853453014
48	H48	-2.942385451	3.270073906	-2.964300262
49	H49	-1.555440417	1.397157997	-4.826424037
50	H50	-0.904634170	1.791667353	-3.224943746
51	H51	-1.091417213	0.098187954	-3.718515682
52	H52	-4.832837891	0.026673509	-3.856749608
53	H53	-3.375654152	-0.972637586	-3.787596784
54	H54	-3.605271462	0.179885034	-5.115275658
55	H55	-5.171837057	-0.429695543	2.043612911
56	H56	5.483611675	-1.072138373	4.040411903
57	H57	5.497696147	0.530032306	0.823140808
58	H58	5.992961087	0.695255912	2.500789624
59	H59	7.293240826	-0.946034025	1.112363893
60	H60	7.149893891	-2.459833107	3.004500561
61	H61	5.002721132	-1.336129791	-0.786064016
62	H62	5.149929506	-3.090337242	-0.999044996
63	H63	6.602496711	-2.066869342	-0.987439565
64	H64	5.224846371	-3.487683931	2.692907821
65	H65	4.709359473	-4.226424695	1.079919831

9 lowest-energy conformers used for Boltzmann-averaged ^{13}C NMR data.
Avg. Energy: -1611.680197 au

VIII.8. 1-isomangostin (R8–B13)[75] and its revised structure 3-isomangostin (B31-13).[76, 77]

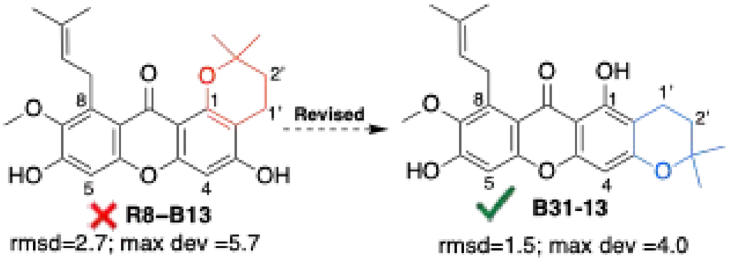

VIII.8a. 1-isomangostin (R8–B13)[75]

No	type	δcal	δexp	diff
C1	C	157.9	161.5	-3.6
C2	C	101.9	103.5	-1.6
C3	C	157.3	161.8	-4.5
C4	CH	92.6	94.8	-2.2
C4a	C	157.3	158.3	-1
C5	CH	100.4	102.9	-2.5
C6	C	153.6	155.9	-2.3
C7	C	140.3	144.8	-4.5
C8	C	140.2	138.4	1.8
C8a	C	116.9	111.9	5.0
C9	C	178.1	183.1	-5.0
C9a	C	110.3	104.6	5.7
C10a	C	155.0	156.8	-1.8
C1'	CH2	17.6	18.3	-0.7
C2'	CH2	30.5	32.8	-2.3
C3'	C	74.5	77.0	-2.5
C4'[2]	CH3	26.5	27.1	-0.6
C1''	CH2	26.8	26.0	0.8
C2''	CH	126.1	125.2	0.9
C3''	C	132.4	131.6	0.8
C4''	CH3	19.1	18.3	0.8
C5''	CH3	25.9	26.0	-0.1
C1'''	CH3	61.4	61.3	0.1
RMSD		2.71		
Max abs		5.66		

mol2 coordinates for lowest energy conformer

1	O1	-0.288849691	-0.232935127	-0.819949731
2	C2	-0.274565804	-3.621193418	2.954054088
3	C3	-0.596448800	3.459629512	2.657484161
4	C4	-0.390223497	-3.832945507	1.573735098
5	C5	-0.637055940	3.505398332	1.251532163
6	C6	-0.235898326	-2.348867819	3.501294179
7	C7	-0.466962979	2.249729483	3.307222234
8	C8	-0.438649252	-2.717751485	0.735983781
9	C9	-0.596293865	2.358620410	0.473906706
10	O10	-0.269899003	-0.050291695	3.251905477
11	C11	-0.376659713	-0.184717065	0.399260090
12	C12	-0.306752531	-1.258889789	2.640305020
13	C13	-0.396274055	1.097168362	2.531566125
14	C14	-0.393424606	-1.390735726	1.252734885
15	C15	-0.457622315	1.105384041	1.132188735
16	C16	-0.744894280	2.514583005	-1.028544302
17	C17	0.574961547	2.501814009	-1.757605642
18	C18	1.042555697	3.414846425	-2.616034279
19	C19	2.377712212	3.218852106	-3.290816584
20	C20	0.326682044	4.679714352	-3.016933479
21	O21	-0.769791745	4.770487413	0.707460498
22	C22	0.486880948	5.375269272	0.394831807
23	O23	-0.696864960	4.607614165	3.357648817
24	O24	-0.215787595	-4.734822393	3.726453694
25	C25	-0.488673028	-5.235013563	1.027657965
26	C26	-0.997665622	-5.212308283	-0.410677542
27	C27	-0.310458549	-4.111390415	-1.219413538
28	O28	-0.563667170	-2.837510504	-0.593684518
29	C29	-0.924449293	-3.971671544	-2.605444208
30	C30	1.204640854	-4.303736289	-1.311883128

31	H31	-0.157691720	-2.177816749	4.569782414
32	H32	-0.436117067	2.190036884	4.387772019
33	H33	-1.349118801	1.687632659	-1.407584445
34	H34	-1.286275754	3.444162318	-1.208616506
35	H35	1.174678920	1.614937655	-1.569470692
36	H36	2.857582256	2.287571502	-2.978521541
37	H37	3.058567258	4.049519748	-3.062964559
38	H38	2.264897967	3.195320645	-4.382619349
39	H39	0.983172948	5.550301854	-2.883292985
40	H40	-0.588894442	4.860410631	-2.448877722
41	H41	0.061894651	4.648005228	-4.081629443
42	H42	0.259580835	6.334361587	-0.074730790
43	H43	1.077282350	5.541433374	1.305776545
44	H44	1.055084959	4.745075030	-0.293708316
45	H45	-0.925648037	5.293914203	2.708441147
46	H46	-0.164287044	-4.475463359	4.654606565
47	H47	0.488909399	-5.730746100	1.082852031
48	H48	-1.158573015	-5.827768879	1.658025914
49	H49	-2.076200924	-5.015180261	-0.419440392
50	H50	-0.837708753	-6.180853652	-0.895921785
51	H51	-0.493130843	-3.107596186	-3.119569074
52	H52	-0.731693765	-4.872386982	-3.197869707
53	H53	-2.006046313	-3.823022155	-2.530800023
54	H54	1.643125522	-3.496077656	-1.904132676
55	H55	1.438625896	-5.260351240	-1.790479201
56	H56	1.672382832	-4.289750364	-0.323265998

18 lowest-energy conformers used for Boltzmann-averaged ^{13}C NMR data.
Avg. Energy: -1381.616480 au

VIII.8b. 3-Isomangostin (B31-13).[76, 77]

No	type	δcal	δexp	diff
C1	C	162.9 (-2.9)	161.5	1.4 (-2.9)
C2	C	102.9	103.5	-0.6
C3	C	161.1	161.8	-0.7
C4	CH	94.3	94.8	-0.5
C4a	C	155.4	158.3	-2.9
C5	CH	101.2	102.9	-1.7
C6	C	155.4	155.9	-0.5
C7	C	140.7	144.8	-4.1
C8	C	140.5 (-2.9)	138.4	2.1 (-2.9)
C8a	C	113.5	111.9	1.6
C9a	C	104.2	104.6	-0.4
C10a	C	156.5	156.8	-0.3
C21	C	184.4	183.1	1.3
C1'	CH2	17.3	18.3	-1.0
C2'	CH2	31.3	32.8	-1.5
C3'	C	75.1	77	-1.9
C4'[2]	CH3	26.4	27.1	-0.7
C1''	CH2	28.1	26	2.1
C2''	CH	127.0	125.2	1.8
C3''	C	131.0	131.6	-0.6
C4''	CH3	18.4	18.3	0.1
C5''	CH3	25.5	26	-0.5
C1'''	CH3	61.1	61.3	-0.2
RMSD		1.52		
Max abs		4.08		
RMSD+CFx		1.48		
Max abs+CFx		4.08		

mol2 coordinates for lowest energy conformer

1	O1	-1.340831480	1.045224344	-0.686734619
2	C2	0.001367587	-1.041371968	3.660928702
3	C3	0.994661365	-2.749297089	-3.169302743
4	C4	-0.759545027	0.104599534	3.391976960
5	C5	0.304264528	-1.619356315	-3.651191761
6	C6	0.521044147	-1.843551083	2.639884063
7	C7	1.147514123	-2.944133081	-1.812036259
8	C8	-0.976371647	0.452516299	2.058792005
9	C9	-0.286348441	-0.700673393	-2.801196808
10	O10	0.792131268	-2.281561006	0.367359891
11	C11	-0.698089721	0.020019115	-0.391321110
12	C12	0.273670778	-1.480776839	1.332555936
13	C13	0.590019335	-2.007948497	-0.946886946
14	C14	-0.465533657	-0.336967642	0.994961842
15	C15	-0.132336303	-0.888086135	-1.398009488
16	C16	-1.078403486	0.436236412	-3.419168772
17	C17	-0.299554643	1.725977988	-3.484220085
18	C18	-0.095403682	2.502227524	-4.554416316
19	C19	0.675517015	3.792305605	-4.429915925
20	C20	-0.603924361	2.218754328	-5.944826794
21	O21	0.222018240	-1.523300225	-5.027132699
22	C22	1.273433995	-0.738959439	-5.594418167
23	O23	1.494772003	-3.636512311	-4.048535388
24	O24	0.269213174	-1.459393196	4.914640686
25	C25	-1.360422731	0.923262875	4.503411912
26	C26	-1.337146469	0.128053492	5.806368864
27	C27	0.012911106	-0.561687660	6.019143826
28	O28	-1.691444347	1.553505554	1.814058575
29	C29	-0.018897787	-1.465826879	7.243468905
30	C30	1.172112868	0.432900167	6.104541811
31	H31	1.104650710	-2.723947756	2.876532394
32	H32	1.675574892	-3.805419136	-1.422151094
33	H33	-1.394377765	0.114025092	-4.412025968
34	H34	-1.973407979	0.607693369	-2.816883947
35	H35	0.098150640	2.056309112	-2.526920052
36	H36	1.018450902	3.964948889	-3.405761097
37	H37	0.056133838	4.647523265	-4.730409153
38	H38	1.552937195	3.791770403	-5.090051813
39	H39	-1.343721185	2.973302981	-6.241595181
40	H40	-1.069735918	1.236164861	-6.046828686
41	H41	0.215751693	2.280331560	-6.672632948
42	H42	2.249359337	-1.205072782	-5.409527818
43	H43	1.083721113	-0.697901733	-6.668443339
44	H44	1.268975462	0.269065894	-5.173247154
45	H45	1.152074046	-3.370763940	-4.918601773
46	H46	-2.386873929	1.198038923	4.241395321
47	H47	-0.818309733	1.870881920	4.616792896
48	H48	-2.112582048	-0.647082928	5.780739776
49	H49	-1.551858830	0.776502892	6.662559595
50	H50	-0.830034059	-2.194815070	7.159640158
51	H51	-0.173923136	-0.870762980	8.148976810
52	H52	0.925929818	-2.008846893	7.340218330
53	H53	2.110637668	-0.101381313	6.278172571
54	H54	1.011813121	1.134867603	6.929557876
55	H55	1.275681145	1.007565503	5.179705196
56	H56	-1.745414750	1.640821786	0.828009004

8 lowest-energy conformers used for Boltzmann-averaged ^{13}C NMR data.
Avg. Energy: -1381.638489 au

VIII.9. Hypejaponol B (R9–B13)[97] and its revised structure (D31-14) (Unreported natura product).

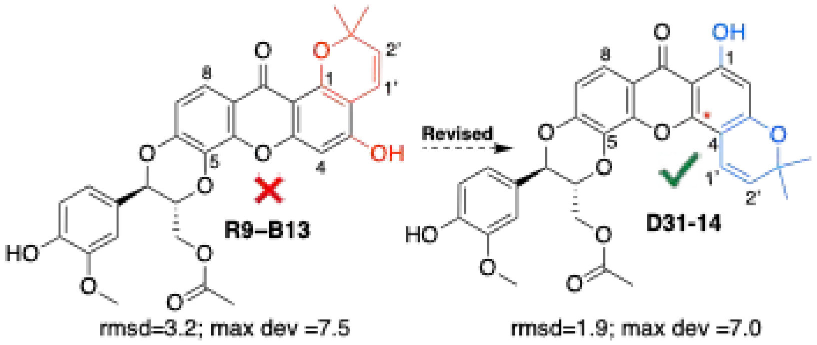

VIII.9a. Hypejaponol B (R9–B13)[97]

No	type	δcal	δexp	diff
C1	C	158.9	160.5	-1.6
C2	C	106.0	101.4	4.6
C3	C	155.5	163	-7.5
C4	CH	93.8	99.4	-5.6
C4a	C	159.0	160.5	-1.5
C5	C	131.5	131.3	0.2
C6	C	148	146	2
C7	CH	112.7	114.9	-2.2
C8	CH	122.3	117.9	4.4
C8a	C	119.7	115	4.7
C9	C	174.4	180	-5.6
C9a	C	109.9	103.3	6.6
C10a	C	145.9	148.7	-2.8
C1'	CH	119.3	115.0	4.3
C2'	CH	125.7	127.2	-1.5
C3'	C	77.9	78.2	-0.3
C4'	CH3	27.4	29.7	-2.3
C5'	CH3	27.3	28.3	-1
C1''	C	126.3	126.0	0.3
C2''	CH	112.4	109.3	3.1
C3''	C	144.1	147.1	-3
C4''	C	147.1	146.9	0.2
C5''	CH	114.6	113.0	1.6
C6''	CH	121.8	120.9	0.9
C7''	CH	80.5	77.2	3.3
C8''	CH	74.6	75.7	-1.1
C9''	CH2	62.2	62.5	-0.3
C10''	C	172.9	170.4	2.5
C11''	CH3	20.1	20.7	-0.6
C3''a	CH3	54.7	56.4	-1.7
RMSD		3.24		
Max abs		7.49		

mol2 coordinates for lowest energy conformer

1	O1	1.563153883	-4.550316317	-0.910096795
2	C2	-1.965763695	-2.932863185	-4.244701954
3	C3	1.320482876	-0.123946646	1.422295239
4	C4	-1.282891487	-4.155995693	-4.191625211
5	C5	2.073303448	-1.277130019	1.677602215
6	C6	-1.714941512	-1.927737565	-3.322398636
7	C7	0.423159321	-0.083366999	0.360485219
8	C8	-0.336217327	-4.351549258	-3.179443834
9	C9	1.936182223	-2.376377029	0.857802243
10	O10	-0.588277956	-1.104203351	-1.482846141

11	C11	0.920039930	-3.533838017	-1.117210367
12	C12	-0.760505974	-2.147633750	-2.334643879
13	C13	0.302845055	-1.207070730	-0.465018187
14	C14	-0.044846243	-3.350316041	-2.220153911
15	C15	1.054825410	-2.353145561	-0.231930686
16	O16	1.487500749	0.946254128	2.242192225
17	C17	0.967348369	2.179207490	1.748136467
18	C18	-0.435454668	1.931759099	1.172644508
19	O19	-0.348032002	1.008654045	0.095369423
20	C20	0.991854933	3.175795514	2.875545313
21	C21	-1.094878960	3.164283558	0.566957305
22	O22	-1.725064349	3.908380668	1.604333857
23	C23	0.563260150	2.815760220	4.148351921
24	C24	0.551050546	3.751377221	5.180624679
25	C25	0.955159175	5.055877295	4.939577822
26	C26	1.379321438	5.426580815	3.651054902
27	C27	1.411971832	4.487180414	2.631511329
28	O28	0.930830003	5.975183901	5.932876102
29	O29	1.730714840	6.738687965	3.543774621
30	C30	1.975837759	7.252621425	2.245213520
31	C31	-1.490894066	5.233502344	1.664252816
32	C32	-2.109628359	5.817247483	2.902617971
33	O33	-0.856160238	5.843653250	0.836097602
34	C34	-1.445559190	-5.202637027	-5.195459342
35	C35	-0.906712906	-6.404021190	-4.983941912
36	C36	-0.206638557	-6.699250244	-3.679515434
37	O37	0.367710444	-5.487629419	-3.134111261
38	O38	-2.870401920	-2.782525455	-5.241136898
39	C39	0.987073992	-7.625902428	-3.871960176
40	C40	-1.188497359	-7.259744798	-2.646274944
41	H41	2.757801215	-1.269089922	2.518542416
42	H42	-2.237087998	-0.976890163	-3.347022309
43	H43	2.508041532	-3.282740969	1.022585654
44	H44	1.605256427	2.524457253	0.921157094
45	H45	-1.075635439	1.520223113	1.965217398
46	H46	-0.376968873	3.792624423	0.034575006
47	H47	-1.863482414	2.831512993	-0.134988035
48	H48	0.253945021	1.794481717	4.342887285
49	H49	0.227528579	3.483474626	6.180866589
50	H50	1.734633372	4.772683611	1.635942052
51	H51	1.245829823	6.809765159	5.554928913
52	H52	1.114802868	7.078373652	1.590966231
53	H53	2.139595121	8.323346913	2.371983618
54	H54	2.875256197	6.800721482	1.809526615
55	H55	-3.165332010	5.542973785	2.967027936
56	H56	-1.602983505	5.401093805	3.778767241
57	H57	-1.998577816	6.901444415	2.890806940
58	H58	-1.982582162	-4.965430488	-6.107215409
59	H59	-0.980239442	-7.204187567	-5.714730623
60	H60	-3.250531845	-1.897367743	-5.194930622
61	H61	1.511883046	-7.757368025	-2.922019631
62	H62	1.683445587	-7.202737370	-4.601213743
63	H63	0.650372899	-8.603912760	-4.230628986
64	H64	-2.011998321	-6.556672469	-2.484377508
65	H65	-1.610888148	-8.205880695	-3.000477044
66	H66	-0.674343319	-7.429704889	-1.695024807

8 lowest-energy conformers used for Boltzmann-averaged ^{13}C NMR data.
Avg. Energy: -1911.172080 au

VIII.9b. D31-14 (Unreported natural product)

No	type	δcal	δexp	diff
C1	C	165.5 (-2.9)	163.0	2.5 (-2.9)
C2	CH	99.5	99.4	0.1
C3	C	161.3	160.5	0.8
C4	C	101.6	101.4	0.2
C4a	C	153.5	160.5	-7.0
C5	C	131.4	131.3	0.1
C6	C	147.3	146.0	1.3
C7	CH	113.7	114.9	-1.2
C8	CH	121.5 (-2.9)	117.9	3.6 (-2.9)
C8a	C	117.3	115.0	2.3
C9	C	182.1	180.0	2.1
C9a	C	105.0	103.3	1.7
C10a	C	146.8	148.7	-1.9
C1'	CH	120.6 (-3.7)	115.0	5.6 (-3.7)
C2'	CH	124.3 (+2.8)	127.2	-2.9 (+2.8)
C3'	C	78.1	78.2	-0.1
C4'	CH3	28.2	29.7	-1.5
C5'	CH3	27.4	28.3	-0.9
C1''	C	124.0	126.0	-2
C2''	CH	110.7	109.3	1.4
C3''	C	144.9	147.1	-2.2
C4''	C	147.0	146.9	0.1
C5''	CH	114.3	113.0	1.3
C6''	CH	122.1	120.9	1.2
C7''	CH	74.9	77.2	-2.3
C8''	CH	74.9	75.7	-0.8
C9''	CH2	60.9	62.5	-1.6
C10''	C	173.5	170.4	3.1
C11''	CH3	21.4	20.7	0.7
C3''a	CH3	54.8	56.4	-1.6
RMSD		2.36		
Max abs		7.05		
RMSD+CFx		1.93		
Max abs+CFx		7.05		

mol2 coordinates for lowest energy conformer

1	O1	4.654338301	-1.589658405	-1.369731091
2	C2	0.074067208	-3.573165824	-2.009297469
3	C3	2.711580137	1.860879478	1.751010341
4	C4	1.298133364	-3.906451304	-2.581711586
5	C5	4.002642300	1.710468419	1.226158071
6	C6	-0.058300890	-2.535272077	-1.067763158
7	C7	1.728183376	0.917469921	1.474968068
8	C8	2.437831802	-3.197697854	-2.219622378
9	C9	4.285327044	0.647453739	0.394636245
10	O10	0.950375708	-0.892216319	0.236551292
11	C11	3.528276181	-1.366974499	-0.900063199
12	C12	1.103364692	-1.865252178	-0.699961447
13	C13	2.002672714	-0.103697737	0.564322709
14	C14	2.359541184	-2.154111172	-1.254967459
15	C15	3.281646181	-0.265087311	0.034930072
16	O16	2.429973313	2.937185452	2.527444827
17	C17	1.027905013	3.274507852	2.573585750
18	C18	0.267984711	2.001467755	2.959062852
19	O19	0.473689663	0.981776704	1.990126292
20	C20	0.583984034	3.970517142	1.304285836
21	C21	-1.239618051	2.177998401	3.088031501
22	O22	-1.820861987	0.899204669	3.330360968

23	C23	0.826955468	5.335427062	1.175949394
24	C24	0.485260741	6.019815694	0.011258291
25	C25	-0.110028810	5.340398075	-1.040304205
26	C26	-0.365795718	3.962845288	-0.917035747
27	C27	-0.018649481	3.284361770	0.237377086
28	O28	-0.450928099	5.991485842	-2.174350272
29	O29	-0.962004233	3.407326926	-2.008192155
30	C30	-1.227308683	2.014410972	-1.974940014
31	C31	-2.280628209	0.225534511	2.255190866
32	C32	-2.573137977	-1.205301519	2.607263198
33	O33	-2.415511348	0.712233440	1.156775023
34	C34	-1.398396683	-2.146953263	-0.645766423
35	C35	-2.425056617	-2.949047121	-0.934816202
36	C36	-2.189243305	-4.289909481	-1.589901763
37	O37	3.601562492	-3.517419900	-2.782711899
38	O38	-1.009489092	-4.246679840	-2.434932822
39	C39	-3.324338130	-4.655492356	-2.538629392
40	C40	-1.970677125	-5.376935572	-0.532771789
41	H41	4.749713630	2.455165629	1.476061277
42	H42	5.275040292	0.507699171	-0.025250809
43	H43	0.673793068	1.646076273	3.915992636
44	H44	-1.470504536	2.798211958	3.956246273
45	H45	-1.672390668	2.619801048	2.190677791
46	H46	1.299347061	5.877426233	1.990597280
47	H47	0.672721590	7.083366659	-0.094829587
48	H48	-0.243992234	2.229944951	0.320664391
49	H49	-0.838650726	5.335377695	-2.772155205
50	H50	-1.900713432	1.756590096	-1.150511471
51	H51	-1.699397826	1.776833858	-2.928139382
52	H52	-0.297272043	1.441476991	-1.874707207
53	H53	-1.642367367	-1.772415130	2.501373596
54	H54	-2.918551541	-1.297605158	3.638978523
55	H55	-3.305494749	-1.615245502	1.911447806
56	H56	-1.543180670	-1.188050757	-0.156983410
57	H57	-3.449015548	-2.678943742	-0.689805340
58	H58	-3.108809279	-5.603560319	-3.040042765
59	H59	-3.450872395	-3.877622035	-3.297301899
60	H60	-4.261201604	-4.760186199	-1.982221811
61	H61	-1.112531789	-5.126445368	0.099354117
62	H62	-2.854299218	-5.461903640	0.107949647
63	H63	-1.787716563	-6.344110037	-1.012537651
64	H64	1.362686577	-4.705885759	-3.309427149
65	H65	4.284545936	-2.908413844	-2.406118407
66	H66	0.963792844	3.976971550	3.408870542

8 lowest-energy conformers used for Boltzmann-averaged ^{13}C NMR data.
Avg. Energy: -1911.192636 au

VIII.10. 2H,6H-Pyrano[3,2-b]xanthen-6-one,12-(1,1-dimethyl-2-propen-1-yl)-5,9,10-trihydroxy-2,2-dimethyl (R10–B31)[5] and its revised structure pedunxanthone C demethoxylated (B34-7).[5]

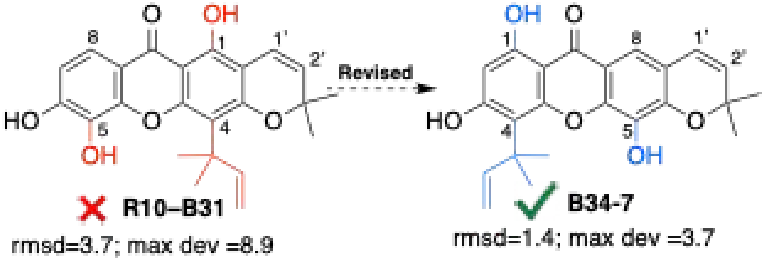

VIII.10a. 2H,6H-Pyrano[3,2-b]xanthen-6-one,12-(1,1-dimethyl-2-propen-1-yl)-5,9,10-trihydroxy-2,2-dimethyl (R10–B31)[5]

No	type	δcal	δexp	diff
C1	C	159.9	162.0	-2.1
C2	C	106.8	100.6	6.2
C3	C	159.2	162.7	-3.5
C4	C	111.3	110.7	0.6
C4a	C	155.9	155.9	0
C5	C	131.2	132.6	-1.4
C6	C	150.4	145.2	5.2
C7	CH	112.1	113.4	-1.3
C8	CH	120.8	118.2	2.6
C8a	C	114.5	114.4	0.1
C9	C	182.9	180.9	2
C9a	C	105.2	104.1	1.1
C10a	C	144.3	144.9	-0.6
C1'	CH	121.3	121.6	-0.3
C2'	CH	123.7	131.2	-7.5
C3'	C	77.8	79.1	-1.3
C4'^{2}	CH3	27.5	27.9	-0.4
C1''	C	42.2	41.4	0.8
C2''	CH	159.5	150.6	8.9
C3''	CH2	104.2	112.6	-8.4
C4''^{2}	CH3	29.5	28.7	0.8
RMSD		3.66		
Max abs		8.92		

mol2 coordinates for lowest energy conformer

1	O1	-1.992253806	0.207763138	-3.934465406
2	O2	-4.127921636	-0.406464007	1.642088332
3	O3	0.577769042	0.469160667	-3.916533127
4	C4	2.958717906	0.792460126	-2.467827274
5	C5	4.081033151	0.726932710	-1.752201846
6	C6	-5.582215060	-0.341211549	-1.702449709
7	C7	-5.449997511	-0.427108268	-0.310302155
8	C8	-4.463924804	-0.158838633	-2.492958669
9	O9	2.744515499	0.604635173	0.263227760
10	O10	-1.903710666	-0.017263729	0.138235978
11	C11	-4.194044924	-0.322377544	0.286487755
12	C12	0.506050625	0.374953010	-2.589350032
13	C13	1.677060620	0.484436003	-1.843550124
14	C14	1.614933593	0.389547639	-0.439703763
15	C15	0.422598026	0.152606537	0.270707861
16	C16	-1.973629999	0.127504316	-2.695913018
17	C17	-3.192591617	-0.051034518	-1.913201204
18	C18	-3.083113542	-0.124901926	-0.524893626
19	C19	-0.727461072	0.112470183	-0.528855085
20	C20	-0.732867809	0.193475647	-1.931988683
21	C21	4.029160649	0.270087144	-0.316345377
22	C22	0.295432903	0.038947864	1.806220692
23	C23	-1.483342533	-1.145319780	3.210522318
24	C24	-0.652049312	-1.100502610	2.168241690
25	C25	1.610808612	-0.353647719	2.527388398
26	C26	-0.175181509	1.393268158	2.365783866
27	O27	-6.540841540	-0.617308600	0.459355212
28	H28	-3.200653340	-0.518912591	1.922813534
29	H29	-0.351192861	0.400915655	-4.258163883
30	H30	-6.573705962	-0.429567876	-2.132165545
31	H31	-4.541833296	-0.098333280	-3.572681994

32	H32	-2.033332912	-2.052231217	3.446492200
33	H33	-1.621352318	-0.303680028	3.884381551
34	H34	-0.544157519	-1.991046208	1.548857172
35	H35	1.374185867	-0.558540730	3.575851618
36	H36	2.053925654	-1.256208303	2.094846747
37	H37	2.349213483	0.445169164	2.480899732
38	H38	-0.200768406	1.381312832	3.460848240
39	H39	0.523740987	2.176913191	2.058594884
40	H40	-1.172044154	1.652938231	1.997554591
41	H41	-6.244439612	-0.666466970	1.381045653
42	H42	5.052223482	0.961411170	-2.177798442
43	H43	2.957315813	1.085783571	-3.511707962
44	C44	5.037183639	1.015506749	0.549323045
45	H45	6.054933735	0.803581694	0.207068482
46	H46	4.949218929	0.699519553	1.593227193
47	H47	4.864244782	2.093493623	0.490421567
48	C48	4.219122002	-1.246257936	-0.219014527
49	H49	5.185065352	-1.533094731	-0.646675790
50	H50	3.432979072	-1.763586083	-0.778166681
51	H51	4.187194297	-1.570888913	0.826427859

4 lowest-energy conformers used for Boltzmann-averaged ^{13}C NMR data.
Avg. Energy: -1341.097086 au

VIII.10b. Dedunxanthone C demethoxylated (B34-7).[5]

No	type	δcal	δexp	diff
C1	C	164.2 (-2.9)	162.7	1.5 (-2.9)
C2	CH	100.5	100.6	-0.1
C3	C	163.7	162.0	1.7
C4	C	107.0	110.7	-3.7
C4a	C	157.3	155.9	1.4
C5	C	134.6	132.6	2
C6	C	143.7	144.9	-1.2
C7	C	118.1	118.2	-0.1
C8	CH	116.5 (-2.9)	113.4	3.1 (-2.9)
C8a	C	117.6	114.4	3.2
C9	C	182.3	180.9	1.4
C9a	C	105.2	104.1	1.1
C10a	C	145.1	145.2	-0.1
C1'	C	42.0	41.4	0.6
C2'	CH	149.2	150.6	-1.4
C3'	CH2	114.4	112.6	1.8
C4'[2]	CH3	29.0	28.7	0.3
C1''	CH	125.3 (-3.7)	121.6	3.7 (-3.7)
C2''	CH	129.3 (+2.8)	131.2	-1.9 (+2.8)
C3''	C	78.4	79.1	-0.7
C4''[2]	CH3	27.2	27.9	-0.7
RMSD		1.78		
Max abs		3.69		
RMSD+CFx		1.43		
Max abs+CFx		3.69		

mol2 coordinates for lowest energy conformer

1	O1	-0.794737687	0.062341364	-3.998104967
2	O2	1.420169824	-0.412934002	1.524237622
3	O3	-3.387258615	0.208774535	-3.947227025
4	C4	2.843062510	-0.332700790	-1.874005807
5	C5	2.736558982	-0.396987133	-0.473793703
6	C6	1.685691840	-0.198440765	-2.622499458
7	O7	-5.566983019	0.015325385	0.136045072

8	O8	-0.809259241	-0.130780905	0.075123447
9	C9	1.511919590	-0.336508391	0.175625972
10	C10	-3.287209949	0.151674975	-2.619298156
11	C11	-4.435686608	0.146743937	-1.853799923
12	C12	-4.362767710	0.095123914	-0.459760527
13	C13	-3.133315949	0.114299552	0.247275291
14	C14	-0.803423934	0.009440532	-2.757748329
15	C15	0.439060837	-0.123758691	-1.992964671
16	C16	0.358068966	-0.195446186	-0.602048081
17	C17	-1.997838957	0.028680189	-0.568221324
18	C18	-2.023338545	0.070938208	-1.976218154
19	C19	-3.050595468	0.080627438	1.791145490
20	O20	3.820601004	-0.580232845	0.325840768
21	C21	-5.076177814	0.345965343	3.333597573
22	C22	-4.241524683	0.807756073	2.400974265
23	C23	-1.848133136	0.874211503	2.375593252
24	C24	-2.962933690	-1.382805989	2.251893767
25	C25	4.189595058	-0.442421671	-2.430266544
26	C26	5.250156892	-0.349260635	-1.625964662
27	C27	5.092032104	-0.066490560	-0.147271052
28	C28	5.129499037	1.441268490	0.116030827
29	C29	6.137523712	-0.806475410	0.676759320
30	H30	2.327403538	-0.485866846	1.857615089
31	H31	-2.467162065	0.177257784	-4.307410463
32	H32	1.717498470	-0.150640012	-3.706166456
33	H33	-5.479523999	0.025648595	1.103804004
34	H34	-5.408642453	0.160027919	-2.328981020
35	H35	-5.851966386	0.980996198	3.751003088
36	H36	-5.017180930	-0.668041798	3.717688160
37	H37	-4.354449155	1.838586665	2.062366257
38	H38	-2.027302626	1.035140677	3.443686214
39	H39	-1.751347503	1.854339741	1.895884785
40	H40	-0.905597094	0.341839426	2.262982545
41	H41	-2.861824361	-1.450457344	3.340996862
42	H42	-3.848383411	-1.951120236	1.945791648
43	H43	-2.084224563	-1.853620122	1.803166403
44	H44	4.297758218	-0.609013310	-3.498089906
45	H45	6.263817345	-0.428279178	-2.008413268
46	H46	5.016231334	1.643689557	1.186315349
47	H47	6.081996184	1.861811682	-0.221588181
48	H48	4.323921605	1.943260800	-0.428147813
49	H49	5.976392843	-0.625172573	1.743615239
50	H50	7.140384735	-0.456156547	0.413609712
51	H51	6.079444925	-1.882158541	0.489321467

5 lowest-energy conformers used for Boltzmann-averaged ^{13}C NMR data.
Avg. Energy: -1341.092571 au

VIII.11. Nigrolineaxanthone G (R11–B34)[42] and its revised structure (D32-9).

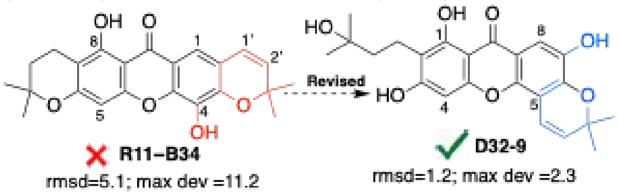

VIII.11a. Nigrolineaxanthone G (R11–B34)[42]

No	type	δcal	δexp	diff
C1	CH	116.4	108.7	7.7
C2	C	118	109.1	8.9
C3	C	144.3	145.7	-1.4

C4	C	135.1	146.4	-11.3
C4a	C	145.7	141.7	4
C5	CH	95.3	94.6	0.7
C6	C	161.4	162.1	-0.7
C7	C	103	111.5	-8.5
C8	C	162.7	160.2	2.5
C8a	C	104.2	102.8	1.4
C9	C	182.3	180.1	2.2
C9a	C	117.6	114.8	2.8
C10a	C	156.3	155.8	0.5
C1'	CH	125.4	115.6	9.8
C2'	CH	129.3	129.7	-0.4
C3'	C	78.1	79.2	-1.1
C4'[2]	CH3	27.2	28.3	-1.1
C1''	CH2	17.3	16	1.3
C2''	CH2	31.3	41.1	-9.8
C3''	C	75.2	72.7	2.5
C4''[2]	CH3	26.4	29.3	-2.9
RMSD		5.10		
Max abs	11.25			

mol2 coordinates for lowest energy conformer

1	C1	-1.381925838	0.641219389	-0.983510820
2	C2	-1.397410553	-0.154395016	0.168102204
3	O3	-0.275502793	-0.608768166	0.774494987
4	C4	0.944587487	-0.299395460	0.260031507
5	C5	1.074838179	0.493341654	-0.895313236
6	C6	-0.092936758	1.009834774	-1.578181531
7	O7	-0.021147780	1.718723925	-2.595195323
8	C8	2.040669352	-0.809458782	0.924208708
9	C9	3.315989111	-0.522827815	0.425752927
10	C10	3.511694610	0.272522876	-0.712386179
11	C11	2.384085684	0.767237831	-1.366181723
12	C12	-2.586735362	1.071937183	-1.549388674
13	C13	-3.797648406	0.727079482	-0.974216167
14	C14	-3.788340957	-0.063630542	0.187386635
15	C15	-2.609500493	-0.515419948	0.766906207
16	O16	4.340899481	-1.048430244	1.128013606
17	C17	5.656456303	-1.064175489	0.528022344
18	C18	5.925977567	0.293430709	-0.126569111
19	C19	4.893198604	0.609701912	-1.205865142
20	C20	-5.109146498	1.099672743	-1.498892446
21	O21	2.573039968	1.521217601	-2.453741854
22	O22	-4.936153865	-0.482767308	0.784115210
23	C23	-6.102823940	0.369145765	0.655589326
24	C24	6.604544800	-1.308928575	1.694104713
25	C25	5.711193584	-2.220647224	-0.471863517
26	O26	-2.617838387	-1.284447851	1.878568569
27	C27	-6.200625010	0.907957375	-0.755785914
28	C28	-7.281477891	-0.535649509	0.989998595
29	C29	-5.969253137	1.517532239	1.660082249
30	H30	1.921559614	-1.427413050	1.804880584
31	H31	-2.543132625	1.680629638	-2.446802333
32	H32	6.939156898	0.300800522	-0.541994802
33	H33	5.888482919	1.059178313	0.657574223
34	H34	4.936576172	1.669185334	-1.477808243
35	H35	5.112896723	0.056263436	-2.128023527
36	H36	-5.168144395	1.519225954	-2.499171540
37	H37	1.675988349	1.787122928	-2.777733490
38	H38	6.541644207	-0.490556869	2.417621563

39	H39	6.348489457	-2.241991954	2.204594845
40	H40	7.635518953	-1.379832558	1.333038493
41	H41	4.955967608	-2.110049055	-1.255381060
42	H42	5.527771705	-3.167604656	0.043746403
43	H43	6.696368413	-2.267061076	-0.947600838
44	H44	-3.547988873	-1.395057704	2.127145019
45	H45	-7.190244387	1.169124507	-1.119869724
46	H46	-7.169474462	-0.948303366	1.997211167
47	H47	-8.215434922	0.033855589	0.950844207
48	H48	-7.342923073	-1.361703670	0.276113500
49	H49	-5.891864473	1.123874556	2.679065124
50	H50	-6.842022197	2.175829891	1.603929296
51	H51	-5.077898670	2.112869759	1.440334983

2 lowest-energy conformers used for Boltzmann-averaged ^{13}C NMR data.
Avg. Energy: -1341.138916 au

VIII.11b. D32-9 (Unreported natural product)

No	type	δcal	δexp	diff
C1	C	162.7 (-2.9)	160.2	2.5 (-2.9)
C2	C	108.7	111.5	-2.8
C3	C	164.3	162.1	2.2
C4	CH	95.1	94.6	0.5
C4a	C	156.5	155.8	0.7
C5	C	111.0	109.1	1.9
C6	C	144.9	146.4	-1.5
C7	C	142.2	141.7	0.5
C8	CH	113.2 (-2.9)	108.7	4.5 (-2.9)
C8a	C	117.0	114.8	2.2
C9	C	181.9	180.1	1.8
C9a	C	104.1	102.8	1.3
C10a	C	145.9	145.7	0.2
C1'	CH2	19.4	16	3.4
C2'	CH2	38.2	41.1	-2.9
C3'	C	72.2	72.7	-0.5
C4'^{2}	CH3	30.5	29.3	1.2
C1''	CH	120.6 (-3.7)	115.6	5.0 (-3.7)
C2''	CH	127.5 (+2.8)	129.7	-2.2 (+2.8)
C3''	C	78.6	79.2	-0.6
C4''^{2}	CH3	27.1	28.3	-1.2
RMSD		2.20		
Max abs		4.97		
RMSD+CFx		1.18		
Max abs+CFx		2.27		

mol2 coordinates for lowest energy conformer

1	C1	1.597304009	-1.426014146	-0.744590136
2	C2	1.785173840	-0.710059936	0.437104442
3	O3	0.770288400	-0.081701854	1.088397517
4	C4	-0.493287796	-0.127240393	0.586669509
5	C5	-0.786432000	-0.832602272	-0.593031514
6	C6	0.253761777	-1.529125936	-1.320782649
7	O7	0.035429093	-2.168271081	-2.363006692
8	C8	-1.461776243	0.567595971	1.283833155
9	C9	-2.774032680	0.559075739	0.801476715
10	C10	-3.139173941	-0.164772280	-0.350203760
11	C11	-2.132220025	-0.835472129	-1.043851029
12	C12	2.693376006	-2.041766442	-1.370516620
13	C13	3.952622403	-1.944423542	-0.821122666
14	C14	4.116198808	-1.228761555	0.379236472
15	C15	3.055117191	-0.599510713	1.019300449

16	O16	-3.668904985	1.283450509	1.495807652
17	C17	-5.006287052	0.671641774	-1.950581446
18	C18	-4.567264273	-0.278048521	-0.823306248
19	O19	-2.467677793	-1.501170582	-2.156279670
20	O20	5.386688006	-1.141653522	0.846954802
21	C21	-5.020616414	2.175691727	-1.627002757
22	C22	-3.635978904	2.822503729	-1.728580221
23	C23	-5.997077066	2.892611026	-2.561061351
24	O24	-5.515051336	2.288233209	-0.274443014
25	H25	-1.212604263	1.131844259	2.173526526
26	H26	-4.427642596	1.525309772	0.920969710
27	H27	-4.369069118	0.511463601	-2.826980784
28	H28	-6.022516761	0.377017214	-2.239208558
29	H29	-4.709410372	-1.296047565	-1.198887769
30	H30	-5.248935006	-0.167707835	0.025858159
31	H31	-1.638864877	-1.918880186	-2.502410069
32	H32	-3.694176186	3.888159920	-1.473546828
33	H33	-2.913251476	2.348327452	-1.062541859
34	H34	-3.254998309	2.746170629	-2.752167407
35	H35	-5.709262650	2.742241113	-3.606711719
36	H36	-6.004651999	3.973040486	-2.370189264
37	H37	-7.011110074	2.506554751	-2.419402251
38	H38	-5.576248496	3.227726092	-0.059442845
39	O39	5.024951061	-2.526300213	-1.410146067
40	C40	3.355460882	0.167491702	2.224287825
41	H41	2.602067158	0.842929617	2.613956770
42	C42	4.551920955	0.036258257	2.800195215
43	H43	4.819231974	0.600347019	3.689258461
44	C44	5.572610800	-0.949361089	2.273701185
45	C45	6.990592922	-0.409256296	2.408946826
46	H46	7.707840834	-1.114984259	1.979199271
47	H47	7.237833752	-0.264949950	3.465227893
48	H48	7.084996339	0.549133526	1.890940415
49	C49	5.415929300	-2.306991185	2.963671361
50	H50	6.152040162	-3.019090338	2.575660775
51	H51	4.411376688	-2.706632243	2.794961465
52	H52	5.564292556	-2.201607770	4.043148988
53	H53	2.530658360	-2.598672458	-2.286264838
54	H54	5.790759417	-2.343742802	-0.846031530

4 lowest-energy conformers used for Boltzmann-averaged ^{13}C NMR data.
Avg. Energy: -1417.575878 au

VIII.12. Soulattrin (R12–B34)[4] and its revised structure macluraxanthone (B31-10).[2, 9, 40]

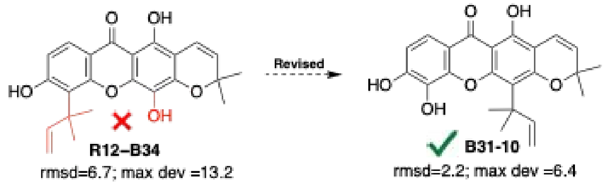

VIII.12a. Soulattrin (R12–B34)[4]

No	type	δcal	δexp	diff
C1	C	152.7	156.5	-3.8
C2	C	105.4	104.9	0.5
C3	C	146.4	158.8	-12.4
C4	C	125.8	133	-7.2
C4a	C	144.2	155.3	-11.1
C5	C	116.3	113.5	2.8
C6	C	161.5	151.2	10.3

C7	CH	114.6	112.9	1.7
C8	CH	129.4	116.2	13.2
C8a	C	115.1	102.8	12.3
C9	C	182.7	181.1	1.6
C9a	C	105.3	104.9	0.4
C10a	C	157.1	146	11.1
C1'	CH	121.6	115.6	6.0
C2'	CH	124.0	127.5	-3.5
C3'	C	78.7	78.2	0.5
C4'2	CH3	27.3	27.2	0.1
C1''	C	41.7	41	0.7
C2''	CH	149.5	151.8	-2.3
C3''	CH2	113.7	106.7	7.0
C4''2	CH3	27.0	29.3	-2.3
RMSD		6.68		
Max abs		13.19		

mol2 coordinates for lowest energy conformer

1	C1	1.530319801	-4.947658810	-0.883851422
2	C2	-0.750176458	-6.034666259	-0.833907754
3	O3	0.560111265	1.786604394	-3.639438513
4	O4	0.415528312	-0.750163420	-4.235700233
5	C5	-0.044213011	-3.430766026	-3.348253784
6	C6	-0.044282311	-4.650690161	-2.806767126
7	C7	0.302788661	4.809684428	-0.730595291
8	C8	0.067405203	4.426191858	0.605740162
9	C9	0.418943635	3.866671320	-1.715523936
10	O10	-0.518944880	-3.699832132	-0.619259785
11	O11	-0.171385834	0.849788585	0.261987330
12	C12	0.012676337	3.073570310	0.994472835
13	C13	0.182283054	-0.955011018	-2.934973551
14	C14	-0.022512496	-2.259312355	-2.481789508
15	C15	-0.273779819	-2.463472092	-1.119042746
16	C16	-0.337586700	-1.423918575	-0.196098774
17	C17	0.342463696	1.493984313	-2.452718347
18	C18	0.274841933	2.508295654	-1.402933142
19	C19	0.051650217	2.148392372	-0.069629270
20	C20	-0.123425616	-0.134886347	-0.665354925
21	C21	0.137891960	0.122502467	-2.019598513
22	C22	0.064264646	-4.839200094	-1.309473126
23	C23	0.314166387	3.653403975	3.436383768
24	C24	-0.188972259	2.604277919	2.455162313
25	C25	-0.367015923	4.207212801	4.440899968
26	O26	-0.591193564	-1.647557014	1.122011058
27	C27	-1.673175699	2.271250487	2.672719123
28	C28	0.680122158	1.366501002	2.820995474
29	O29	-0.098099710	5.470829100	1.439462654
30	H30	2.001488878	-5.807878557	-1.369438728
31	H31	2.078950516	-4.048504611	-1.178726967
32	H32	1.605756087	-5.071992971	0.201761714
33	H33	-1.797283359	-5.923168486	-1.127198375
34	H34	-0.696119595	-6.123389291	0.255259140
35	H35	-0.356940301	-6.954927539	-1.276013628
36	H36	0.522487654	0.226570285	-4.353737858
37	H37	-0.081293114	-3.276178183	-4.420877370
38	H38	-0.080964085	-5.546792067	-3.419121697
39	H39	0.369107876	5.870927295	-0.941059945
40	H40	0.598675496	4.138517038	-2.749835366
41	H41	1.370089952	3.904569301	3.320012584
42	H42	-1.417352781	3.994998303	4.618729113

43	H43	0.116170625	4.892690371	5.130191593
44	H44	-0.671805004	-2.607282182	1.223867890
45	H45	-1.979912866	1.487361220	1.975776468
46	H46	-2.308622016	3.148506067	2.505498193
47	H47	-1.845427435	1.901863890	3.689774708
48	H48	1.707151178	1.488987468	2.460345046
49	H49	0.276189245	0.442232694	2.412124227
50	H50	0.712900572	1.273693811	3.911678311
51	H51	-0.273940512	5.167169459	2.346066007

2 lowest-energy conformers used for Boltzmann-averaged ^{13}C NMR data.
Avg. Energy: -1341.091702 au

VIII.12b. Macluraxanthone (B31-10).[2, 9, 40]

No	type	δcal	δexp	diff
C1	C	159.9 (-2.9)	156.5	3.4 (-2.9)
C2	C	106.8	104.9	1.9
C3	C	159.2	158.8	0.4
C4	C	111.3	104.9*	6.4
C4a	C	155.9	155.3	0.6
C5	C	131.2	133.0*	-1.8
C6	C	150.3	151.2	-0.9
C7	CH	112.1	112.9	-0.8
C8	CH	120.8 (-2.9)	116.2	4.6 (-2.9)
C8a	C	114.5	113.5	1.0
C9	C	182.9	181.1	1.8
C9a	C	105.2	102.8*	2.4
C10a	C	144.3	146.0	-1.7
C1'	CH	121.3 (-3.7)	115.6	5.7 (-3.7)
C2'	CH	123.8 (+2.8)	127.5	-3.7 (+2.8)
C3'	C	77.8	78.2	-0.4
C4'[2]	CH3	27.5	27.2	0.3
C1''	C	42.3	41	1.3
C2''	CH	157.0	151.8	5.2
C3''	CH2	104.2	106.7	-2.5
C4''[2]	CH3	29.4	29.3	0.1
RMSD		3.63		
Max abs		11.67		
RMSD+CFx		2.16		
Max abs+CFx		6.45		

mol2 coordinates for lowest energy conformer in **SI: V.28**
4 lowest-energy conformers used for Boltzmann-averaged ^{13}C NMR data.
Avg. Energy: -1341.097063 au.

VIII.13. 1,3,3-Trihydroxy-6',6'-dimethylpyrano(2',3':6,7)-4-(1,1-dimethylprop-2-enyl)-xanthone (R13–B34),[5] and its revised structure macluraxanthone (B31-10).[2, 9, 40]

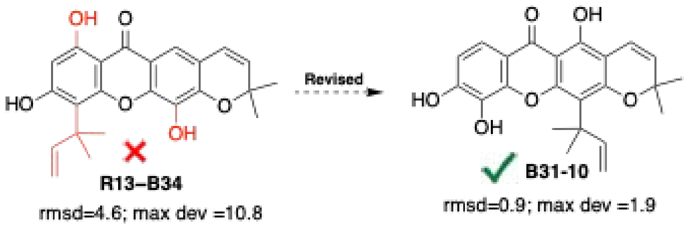

VIII.13a. 1,3,3-Trihydroxy-6',6'-dimethylpyrano(2',3':6,7)-4-(1,1-dimethylprop-2-enyl)-xanthone (R13–B34)[5]

No	type	δcal	δexp	diff
C1	CH	116.5	117.7	-1.2
C2	C	118.1	113	5.1

C3	C	143.7	149.3	-5.6
C4	C	134.6	131.3	3.3
C4a	C	145.1	144.8	0.3
C5	C	107	113.3	-6.3
C6	C	163.7	159.1	4.6
C7	CH	100.5	105.8	-5.3
C8	C	164.2	157	7.2
C8a	C	105.2	105.8	-0.6
C9	C	182.3	181	1.3
C9a	C	117.6	113.9	3.7
C10a	C	157.3	154.3	3
C1'	CH	125.3	116.3	9
C2'	CH	129.3	127.4	1.9
C3'	C	78.4	78.5	-0.1
C4'[2]	CH3	27.2	28.2	-1
C1''	C	42	41.7	0.3
C2''	CH	149.2	157	-7.8
C3''	CH2	114.4	103.6	10.8
C4''[2]	CH3	29	28.4	0.6
RMSD		4.65		
Max abs		10.76		

mol2 coordinates for lowest energy conformer

1	O1	-0.794737687	0.062341364	-3.998104967
2	O2	1.420169824	-0.412934002	1.524237622
3	O3	-3.387258615	0.208774535	-3.947227025
4	C4	2.843062510	-0.332700790	-1.874005807
5	C5	2.736558982	-0.396987133	-0.473793703
6	C6	1.685691840	-0.198440765	-2.622499458
7	O7	-5.566983019	0.015325385	0.136045072
8	O8	-0.809259241	-0.130780905	0.075123447
9	C9	1.511919590	-0.336508391	0.175625972
10	C10	-3.287209949	0.151674975	-2.619298156
11	C11	-4.435686608	0.146743937	-1.853799923
12	C12	-4.362767710	0.095123914	-0.459760527
13	C13	-3.133315949	0.114299552	0.247275291
14	C14	-0.803423934	0.009440532	-2.757748329
15	C15	0.439060837	-0.123758691	-1.992964671
16	C16	0.358068966	-0.195446186	-0.602048081
17	C17	-1.997838957	0.028680189	-0.568221324
18	C18	-2.023338545	0.070938208	-1.976218154
19	C19	-3.050595468	0.080627438	1.791145490
20	O20	3.820601004	-0.580232845	0.325840768
21	C21	-5.076177814	0.345965343	3.333597573
22	C22	-4.241524683	0.807756073	2.400974265
23	C23	-1.848133136	0.874211503	2.375593252
24	C24	-2.962933690	-1.382805989	2.251893767
25	C25	4.189595058	-0.442421671	-2.430266544
26	C26	5.250156892	-0.349260635	-1.625964662
27	C27	5.092032104	-0.066490560	-0.147271052
28	C28	5.129499037	1.441268490	0.116030827
29	C29	6.137523712	-0.806475410	0.676759320
30	H30	2.327403538	-0.485866846	1.857615089
31	H31	-2.467162065	0.177257784	-4.307410463
32	H32	1.717498470	-0.150640012	-3.706166456
33	H33	-5.479523999	0.025648595	1.103804004
34	H34	-5.408642453	0.160027919	-2.328981020
35	H35	-5.851966386	0.980996198	3.751003088
36	H36	-5.017180930	-0.668041798	3.717688160
37	H37	-4.354449155	1.838586665	2.062366257

38	H38	-2.027302626	1.035140677	3.443686214
39	H39	-1.751347503	1.854339741	1.895884785
40	H40	-0.905597094	0.341839426	2.262982545
41	H41	-2.861824361	-1.450457344	3.340996862
42	H42	-3.848383411	-1.951120236	1.945791648
43	H43	-2.084224563	-1.853620122	1.803166403
44	H44	4.297758218	-0.609013310	-3.498089906
45	H45	6.263817345	-0.428279178	-2.008413268
46	H46	5.016231334	1.643689557	1.186315349
47	H47	6.081996184	1.861811682	-0.221588181
48	H48	4.323921605	1.943260800	-0.428147813
49	H49	5.976392843	-0.625172573	1.743615239
50	H50	7.140384735	-0.456156547	0.413609712
51	H51	6.079444925	-1.882158541	0.489321467

5 lowest-energy conformers used for Boltzmann-averaged ^{13}C NMR data.
Avg. Energy: -1341.092571 au

VIII.13b. Macluraxanthone (B31-10).[2, 9, 40]

No	type	δcal	δexp	diff
C1	C	159.9 (-2.9)	157	2.9 (-2.9)
C2	C	106.8	105.8	1
C3	C	159.2	159.1	0.1
C4	C	111.4	113.3	-1.9
C4a	C	155.9	154.3	1.6
C5	C	131.2	131.3	-0.1
C6	C	150.3	149.3	1.0
C7	CH	112.1	113	-0.9
C8	CH	120.8 (-2.9)	117.7	3.1 (-2.9)
C8a	C	114.5	113.9	0.6
C9	C	182.9	181	1.9
C9a	C	105.2	105.8	-0.6
C10a	C	144.3	144.8	-0.5
C1'	CH	121.3 (-3.7)	116.3	5 (-3.7)
C2'	CH	123.7 (+2.8)	127.4	-3.7 (+2.8)
C3'	C	77.8	78.5	-0.7
C4'[2]	CH3	27.5	28.2	-0.7
C1''	C	42.2	41.7	0.5
C2''	CH	157.0	157	0.0
C3''	CH2	104.2	103.6	0.6
C4''[2]	CH3	29.5	28.4	1.1
RMSD		1.88		
Max abs		5.03		
RMSD+CFx		0.93		
Max abs+CFx		1.87		

mol2 coordinates for lowest energy conformer in **SI: V.28**
4 lowest-energy conformers used for Boltzmann-averaged ^{13}C NMR data.
Avg. Energy: -1341.097063 au.

VIII.14. Mesuaferrin A (R14–C21)[6,98] and its revised structure macluraxanthone (B31-10)[2, 9, 40]

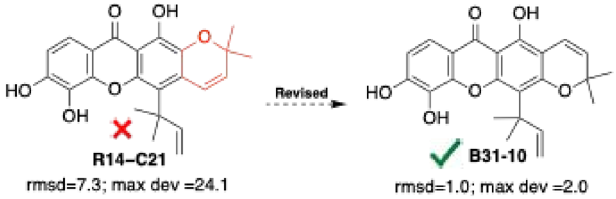

VIII.14a. Mesuaferrin A (R14–C21)[6,98]

No	type	δcal	δexp	diff
C1	C	154.2	156.8	-2.6
C2	C	138.9	159.0	-20.1
C3	C	129.8	105.7	24.1
C4	C	116.3	113.2	3.1
C4a	C	147.4	154.2	-6.8
C5	C	130.9	131.1	-0.2
C6	C	150.3	149.1	1.2
C7	CH	111.8	112.9	-1.1
C8	CH	121.0	117.6	3.4
C8a	C	114.2	113.8	0.4
C9	C	183.8	180.9	2.9
C9a	C	110.1	103.4	6.7
C10a	C	144.5	144.6	-0.1
C1'	CH	124.2	116.2	8
C2'	CH	131.5	127.3	4.2
C3'	C	73.8	78.3	-4.5
C4'[2]	CH3	25.8	28	-2.2
C1''	C	42.1	41.5	0.6
C2''	CH	158.5	156.9	1.6
C3''	CH2	103.2	103.4	-0.2
C4''[2]	CH3	27.7	28.3	-0.6
RMSD		7.32		
Max abs		24.11		

mol2 coordinates for lowest energy conformer

1	O1	-1.940556520	0.404159606	-3.654186024
2	O2	-4.224210614	-0.000065326	1.881458062
3	O3	0.653117062	0.459277440	-3.606055135
4	O4	2.860273119	0.579366856	-2.172287336
5	C5	4.055396916	0.089428025	-1.540740085
6	C6	-5.598485116	0.164711723	-1.493857817
7	C7	-5.500738687	0.079819819	-0.098100826
8	C8	-4.453423661	0.244340373	-2.261253304
9	C9	2.908592804	0.673558536	0.554957315
10	O10	-1.942558521	0.167744671	0.421182479
11	C11	-4.253637459	0.077591241	0.523865405
12	C12	0.532734225	0.383021271	-2.282491420
13	C13	1.694615506	0.410767466	-1.501874576
14	C14	1.632212634	0.358972273	-0.103885883
15	C15	0.401847150	0.132913844	0.571120397
16	C16	-1.948513666	0.327223223	-2.417677113
17	C17	-3.188914449	0.244388740	-1.655349948
18	C18	-3.110977071	0.164786170	-0.264324146
19	C19	-0.739823037	0.197190115	-0.225517252
20	C20	-0.712005734	0.310324552	-1.630801120
21	C21	4.059190170	0.581181535	-0.114267131
22	C22	0.308262979	-0.154085049	2.089018133
23	C23	-1.678337923	-0.929576306	3.504474110
24	C24	-0.898536025	-1.023717861	2.426355126

25	C25	1.475275359	-1.062719117	2.568460661
26	C26	0.290500866	1.165605874	2.877242898
27	C27	5.196306705	0.698327356	-2.344959144
28	C28	4.078318364	-1.440068664	-1.609533921
29	O29	-6.620037416	-0.003826049	0.649466262
30	H30	-3.306021088	-0.126162868	2.185988840
31	H31	-0.265466094	0.461950724	-3.970379708
32	H32	-6.585316737	0.161926258	-1.942546670
33	H33	-4.503360192	0.308058052	-3.342404313
34	H34	2.897992526	1.079713475	1.558151940
35	H35	5.002462023	0.875867491	0.337027554
36	H36	-2.426270856	-1.689432345	3.714716668
37	H37	-1.590281033	-0.122853761	4.226960014
38	H38	-1.034656012	-1.875920291	1.760215203
39	H39	1.288171306	-1.359762915	3.604635452
40	H40	1.533201100	-1.970134965	1.958111629
41	H41	2.449101204	-0.577363409	2.533539302
42	H42	0.341238181	0.985692920	3.956317075
43	H43	1.143080234	1.794153878	2.606302353
44	H44	-0.621263099	1.731159633	2.659930749
45	H45	5.123009647	0.388187648	-3.390989174
46	H46	6.159913190	0.365909412	-1.945399146
47	H47	5.151759030	1.790276285	-2.301219627
48	H48	4.033535445	-1.767588857	-2.652843414
49	H49	4.996561382	-1.825429156	-1.155275624
50	H50	3.226572829	-1.864233151	-1.069568058
51	H51	-6.349850945	-0.064656393	1.578290290

2 lowest-energy conformers used for Boltzmann-averaged ^{13}C NMR data.
Avg. Energy: -1341.079521 au

VIII.14b. Macluraxanthone (B31-10).[2, 9, 40]

No	type	δcal	δexp	diff
C1	C	159.9 (-2.9)	156.8	3.1 (-2.9)
C2	C	106.8	105.7	1.1
C3	C	159.2	159	0.2
C4	C	111.4	113.2	-1.8
C4a	C	155.9	154.2	1.7
C5	C	131.2	131.1	0.1
C6	C	150.4	149.1	1.3
C7	CH	112.1	112.9	-0.8
C8	CH	120.8 (-2.9)	117.6	3.2 (-2.9)
C8a	C	114.5	113.8	0.7
C9	C	182.9	180.9	2
C9a	C	105.2	103.4	1.8
C10a	C	144.3	144.6	-0.3
C1'	CH	121.3 (-3.7)	116.2	5.1 (-3.7)
C2'	CH	123.7 (+2.8)	127.3	-3.6 (+2.8)
C3'	C	77.8	78.3	-0.5
C4'[2]	CH3	27.5	28.0	-0.5
C1''	C	42.2	41.5	0.7
C2''	CH	157.0	156.9	0.1
C3''	CH2	104.2	103.4	0.8
C4''[2]	CH3	29.5	28.3	1.2
RMSD		1.95		
Max abs		5.13		
RMSD+CFx		1.00		
Max abs+CFx		1.97		

mol2 coordinates for lowest energy conformer in **SI: V.28**
4 lowest-energy conformers used for Boltzmann-averaged ^{13}C NMR data.
Avg. Energy: -1341.097063 au.

VIII.15. Inophyllin B (R15–D31)[7, 8] and its revised structure macluraxanthone (B31-10).[2, 9]

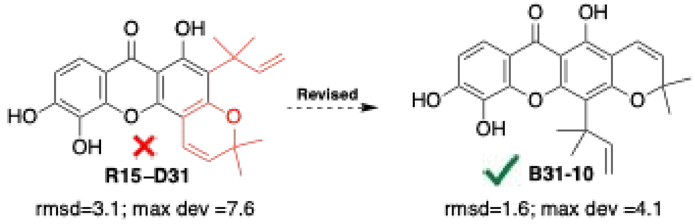

VIII.15a. Inophyllin B (R15–D31)[7, 8]

No	type	δcal	δexp	diff
C1	C	165.30	159.20	6.10
C2	C	117.60	114.00	3.60
C3	C	160.70	157.00	3.70
C4	C	101.50	105.40	-3.90
C4a	C	151.10	155.50	-4.40
C5	C	129.90	133.20	-3.30
C6	C	149.60	146.30	3.30
C7	CH	112.20	113.40	-1.20
C8	CH	121.70	116.80	4.90
C8a	C	115.00	113.80	1.20
C9	C	182.60	181.40	1.20
C9a	C	104.50	103.40	1.10
C10a	C	143.70	151.30	-7.60
C1'	C	41.90	41.50	0.40
C2'	CH	152.20	152.80	-0.60
C3'	CH2	109.00	106.70	2.30
C4'[2]	CH3	29.30	29.80	-0.50
C1''	CH	118.30	116.20	2.10
C2''	CH	125.60	127.30	-1.70
C3''	C	78.00	78.90	-0.90
C4''[2]	CH3	27.50	27.90	-0.40
RMSD		3.11		
Max abs		7.63		

mol2 coordinates for lowest energy conformer

1	C1	-0.437330682	-4.712713587	0.310232513
2	C2	2.268910193	-2.339474379	1.706626792
3	C3	3.229211968	-3.134393158	-0.487422103
4	C4	-3.035709284	-2.769026062	-0.489033614
5	C5	-1.114253570	-3.042144399	-2.048235450
6	O6	-3.346153539	2.429946606	-0.200056251
7	O7	0.610201250	7.298981039	0.840035145
8	O8	2.012561401	4.996376849	0.666053433
9	O9	-3.125166135	-0.089863859	-0.532134058
10	C10	-0.925810141	-3.485601892	0.456352505
11	C11	2.431382284	0.428389404	-0.047042609
12	C12	3.162867923	-0.681157869	0.058469951
13	C13	-1.442656100	6.151626771	0.485962060
14	C14	-2.121532152	4.969683210	0.262496707
15	O15	1.189844756	-1.988675341	-0.426462975
16	O16	0.723906954	2.640808768	0.222484188
17	C17	-0.048755787	6.143015399	0.620191645
18	C18	0.656436221	4.944444209	0.527317701
19	C19	-1.807988746	0.006965794	-0.365753556
20	C20	0.976259484	0.338401586	-0.072583703
21	C21	0.383199993	-0.922080480	-0.280492840
22	C22	-1.000791136	-1.143236142	-0.409702047
23	C23	-2.112684864	2.482985842	-0.068286604
24	C24	-1.429762284	3.755306734	0.166574259
25	C25	-0.041412838	3.764533308	0.299020135

26	C26	0.129956822	1.431676569	0.008368397
27	C27	-1.256954103	1.305852079	-0.134452135
28	C28	2.479153734	-2.016099817	0.224777301
29	C29	-1.504941838	-2.587957547	-0.630367275
30	H30	-0.132562103	-5.298872289	1.172732175
31	H31	-0.316925073	-5.179962336	-0.662446285
32	H32	1.658039775	-1.565978844	2.183793518
33	H33	3.233822498	-2.378140016	2.222960598
34	H34	1.764903138	-3.304656899	1.812850681
35	H35	2.664006675	-4.067712955	-0.407087513
36	H36	3.359763332	-2.891590248	-1.545794411
37	H37	4.215223327	-3.277286071	-0.034175281
38	H38	-3.406428393	-2.412566092	0.475516731
39	H39	-3.248589044	-3.840299712	-0.563217684
40	H40	-3.581774722	-2.245795250	-1.274399419
41	H41	-0.030471282	-3.064163996	-2.180377506
42	H42	-1.544085835	-2.354940944	-2.783734374
43	H43	-1.513193844	-4.041105819	-2.254919285
44	H44	1.556528422	7.100635032	0.901526050
45	H45	2.361254590	4.096319187	0.625170984
46	H46	-3.506694083	0.823750240	-0.441782216
47	H47	-1.016580247	-3.073508270	1.462225132
48	H48	2.895410679	1.403803181	-0.149711575
49	H49	4.248756312	-0.657191100	0.057587104
50	H50	-1.962016528	7.100266715	0.563413129
51	H51	-3.200377380	4.952426853	0.156931935

4 lowest-energy conformers used for Boltzmann-averaged ^{13}C NMR data.
Avg. Energy: -1341.091179 au

VIII.15b. Macluraxanthone (B31-10).[2, 9]

No	type	δcal	δexp	diff
C1	C	159.90 (-2.9)	157.00	2.90 (-2.9)
C2	C	106.80	105.40	1.40
C3	C	159.20	159.20	0.00
C4	C	111.40	114.00	-2.60
C4a	C	155.90	155.50	0.40
C5	C	131.20	133.20*	-2.00
C6	C	150.40	151.30	-0.90
C7	CH	112.10	113.40	-1.30
C8	CH	120.80 (-2.9)	116.80	4.00 (-2.9)
C8a	C	114.50	113.80	0.70
C9	C	182.90	181.40	1.50
C9a	C	105.20	103.40	1.80
C10a	C	144.30	146.30*	-2.00
C1'	CH	121.30 (-3.7)	116.20	5.10 (-3.7)
C2'	CH	123.70 (+2.8)	127.30	-3.60 (+2.8)
C3'	C	77.80	78.90	-1.10
C4'[2]	CH3	27.50	27.90	-0.40
C1''	C	42.20	41.50	0.70
C2''	CH	157.00	152.80	4.20
C3''	CH2	104.20	106.70	-2.60
C4''[2]	CH3	29.50	29.80	-0.30
RMSD		2.48		
Max abs		6.62		
RMSD+CFx		1.65		
Max abs+CFx		4.12		

mol2 coordinates for lowest energy conformer in **SI: V.28**
4 lowest-energy conformers used for Boltzmann-averaged ^{13}C NMR data.
Avg. Energy: -1341.097063 au.

VIII.16. Sterigmatin (analogo) (R16–C21a)[78] and its revised structure demethylsterigmatocystin (D31-16).[79]

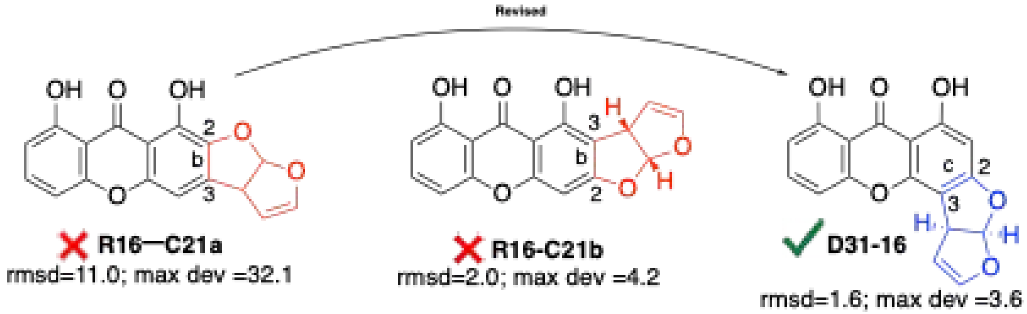

VIII.16a. Sterigmatin (analogo) (**R16–C21a**)[78]

No	type	δcal	δexp	diff
C1	C	148.0	164.1	-16.1
C2	C	140.6	166.0	-25.4
C3	C	139.5	107.4	32.1
C4	CH	100.9	94.4	6.5
C4a	C	151.2	155.7	-4.5
C5	CH	104.9	106.8	-1.9
C6	CH	137.6	136.8	0.8
C7	CH	110.2	111.3	-1.1
C8	C	163.6	161.4	2.2
C8a	C	108.2	107.4	0.8
C9	C	188.7	184.4	4.3
C9a	C	109.5	107.4	2.1
C10a	C	156.6	155.7	0.9
C1'	CH	113.8	113.1	0.7
C2'	CH	52.8	47.5	5.3
C3'	CH	100.5	102.4	-1.9
C4'	CH	147.1	145.3	1.8
RMSD		11.02		
Max abs		32.12		

mol2 coordinates for lowest energy conformer

1	O1	0.358788311	1.487293972	-2.728755659
2	C2	-0.559177598	-2.062689418	0.683913730
3	C3	0.132388605	5.009296123	0.846731909
4	C4	-0.338399813	-2.137536306	-0.681399700
5	C5	0.365039365	5.114960077	-0.520018609
6	C6	-0.547735730	-0.857953252	1.369019956
7	C7	-0.091786203	3.783647212	1.463754716
8	C8	-0.100707513	-1.003550658	-1.448817290
9	C9	0.377192806	3.966684947	-1.309042992
10	O10	-0.303530863	1.463490168	1.308946640
11	C11	0.156404069	1.477995965	-1.490949164
12	C12	-0.309801512	0.288101743	0.623299119
13	C13	-0.079915782	2.640955650	0.675730440
14	C14	-0.085778058	0.243323510	-0.767814925
15	C15	0.151687747	2.698510223	-0.709686908
16	O16	0.098468988	-1.118804817	-2.760859317
17	O17	0.601559192	4.089231538	-2.618075681
18	C18	-0.735890997	-3.472071617	1.191970384
19	C19	-0.639560190	-4.304239516	-0.125146784
20	O20	-0.403520215	-3.402488473	-1.192035259
21	C21	0.449344956	-3.999484607	1.964799018
22	C22	1.051346684	-4.921574928	1.221807697
23	O23	0.454932659	-5.191833602	0.023368182

24	H24	-1.511839064	-4.902324209	-0.387864402
25	H25	-1.688517410	-3.606000257	1.714401192
26	H26	0.125956633	5.911101668	1.451269008
27	H27	0.538593232	6.073471223	-0.994564393
28	H28	-0.712415607	-0.788321295	2.437557316
29	H29	-0.273230882	3.697375073	2.528013859
30	H30	0.244764439	-0.211128313	-3.111047569
31	H31	0.571692310	3.187572189	-3.011918758
32	H32	0.768017780	-3.641946334	2.932956435
33	H33	1.935629661	-5.511063677	1.420457808

1 lowest-energy conformers used for Boltzmann-averaged ^{13}C NMR data.
Avg. Energy: -1105.216110 au

VIII.16b. Sterigmatin (R16–C21b)[79]

C	Type	Theory	Expt.	diff.
C1	C	159.1	161.4	-2.3
C2	C	109.8	107.4	2.4
C3	C	166.3	166.0	0.3
C4	CH	90.2	94.4	-4.2
C4a	C	158.7	155.7	3.0
C5	CH	105.1	106.8	-1.7
C6	CH	137.1	136.8	0.3
C7	CH	110.7	111.3	-0.6
C8	C	163.3	164.1	-0.8
C8a	C	108.3	107.4	0.9
C9	C	187.0	184.4	2.6
C9a	C	104.4	107.4	-3.0
C10a	C	156.4	155.7	0.7
C1'	CH	115.6	113.1	2.5
C2'	CH	48.9	47.5	1.4
C3'	CH	102.9	102.4	0.5
C4'	CH	145.0	145.3	-0.3
RMSD		1.98		
Max abs		4.22		

mol2 coordinates for lowest energy conformer

1	O1	-1.361240199	-1.207828179	-2.109712201
2	C2	1.973882719	0.986328129	0.897010487
3	C3	-5.101170337	0.507286191	0.777245659
4	C4	2.152875220	0.236676060	-0.263082753
5	C5	-5.126015451	-0.271025289	-0.374295568
6	C6	0.734508108	1.284495364	1.440129257
7	C7	-3.910289964	0.957611779	1.337116104
8	C8	1.058867295	-0.277812646	-0.927381368
9	C9	-3.929731488	-0.618998614	-0.999292611
10	O10	-1.576454607	1.061930453	1.287840473
11	C11	-1.426043283	-0.510060145	-1.065915738
12	C12	-0.362350396	0.772323044	0.757888037
13	C13	-2.721814985	0.607847856	0.712949956
14	C14	-0.243428500	-0.002549967	-0.414438257
15	C15	-2.697601438	-0.175218611	-0.452864583
16	O16	1.244429327	-1.014830154	-2.023523958
17	O17	-3.973474722	-1.366399248	-2.103492024
18	C18	3.625386691	0.078161322	-0.517248030
19	C19	4.228745838	0.943702678	0.629308304
20	O20	3.137501713	1.412522198	1.432341043
21	C21	4.178613423	-1.285288445	-0.174458481
22	C22	4.937431632	-1.167087265	0.907390826
23	O23	5.065570260	0.110958810	1.394629426
24	H24	4.802574105	1.821972779	0.334971955

25	H25	3.920589866	0.420165972	-1.514026785
26	H26	-6.039537225	0.771729360	1.254801424
27	H27	-6.057626184	-0.619291535	-0.804382277
28	H28	0.614302058	1.873671147	2.338981767
29	H29	-3.886453732	1.563722003	2.234369458
30	H30	0.352401758	-1.281068157	-2.350141579
31	H31	-3.046011684	-1.511026804	-2.401363220
32	H32	3.941508431	-2.193545440	-0.708275823
33	H33	5.490055751	-1.909074645	1.466921080

1 lowest-energy conformers used for Boltzmann-averaged ^{13}C NMR data.
Avg. Energy: -1105.229139 au

VIII.16c. Demethylsterigmatocystin (D31-16).[79]

No	type	δcal	δexp	diff
C1	C	166.2 (-2.9)	164.1*	2.1 (-2.9)
C2	CH	94.5	94.4	0.1
C3	C	166.6	166.0	0.6
C4	C	105.1	107.4	-2.3
C4a	C	152.9	155.7	-2.8
C5	CH	104.7	106.8	-2.1
C6	CH	137.0	136.8	0.2
C7	CH	111.1	111.3	-0.2
C8	C	163.6 (-2.9)	161.4*	2.2 (-2.9)
C8a	C	108.3	107.4	0.9
C9	C	186.5	184.4	2.1
C9a	C	103.8	107.4	-3.6
C10a	C	156.2	155.7	0.5
C1'	CH	115.4	113.1	2.3
C2'	CH	48.8	47.5	1.3
C3'	CH	102.3	102.4	-0.1
C4'	CH	145.6	145.3	0.3
RMSD		1.77		
Max abs		3.62		
RMSD+CFx		1.62		
Max abs+CFx		3.62		

mol2 coordinates for lowest energy conformer

1	O1	-2.366382351	-2.160167955	-1.384520827
2	C2	2.304853419	-1.488689570	0.241916073
3	C3	-3.654547931	2.401560431	0.286529683
4	C4	1.753414025	-2.578464218	-0.411052188
5	C5	-4.399189932	1.427169880	-0.365503029
6	C6	1.593548586	-0.339766418	0.580304395
7	C7	-2.309752752	2.217222739	0.593263979
8	C8	0.404432486	-2.498865182	-0.753812521
9	C9	-3.796412705	0.223270481	-0.729909085
10	O10	-0.398156878	0.873455737	0.545481633
11	C11	-1.756676428	-1.234643575	-0.792967432
12	C12	0.266501632	-0.264988021	0.227418262
13	C13	-1.715290098	1.019136145	0.229485748
14	C14	-0.363275714	-1.338937083	-0.438589901
15	C15	-2.427077743	0.003997000	-0.431221470
16	O16	-0.148362250	-3.534480359	-1.382380998
17	O17	-4.531044517	-0.697198939	-1.355373741
18	C18	2.526568250	0.643727504	1.228904374
19	C19	3.844999878	-0.187837341	1.286678997
20	O20	3.595976840	-1.437564627	0.636773568
21	C21	2.948028885	1.795599641	0.346661002
22	C22	4.224701426	1.628719811	0.024490094
23	O23	4.830247074	0.532997851	0.584748310

24	H24	4.237061434	-0.417616753	2.277095729
25	H25	2.183366921	0.965662697	2.217127486
26	H26	-4.134930608	3.334451798	0.565048525
27	H27	-5.446158358	1.575721305	-0.602489965
28	H28	-1.724857845	2.973642611	1.102221392
29	H29	-1.094831752	-3.313751405	-1.548029473
30	H30	-3.949065401	-1.471582018	-1.533750760
31	H31	2.291897101	2.583766730	0.008403992
32	H32	4.878519323	2.224171360	-0.597530473
33	H33	2.331895981	-3.459720255	-0.655421377

1 lowest-energy conformers used for Boltzmann-averaged ^{13}C NMR data.
Avg. Energy: -1105.227573 au

VIII.17. Garcimangosone A (R17–C24)[80] and its revised structure mangostenone A (B31-19–D32-5).[28]

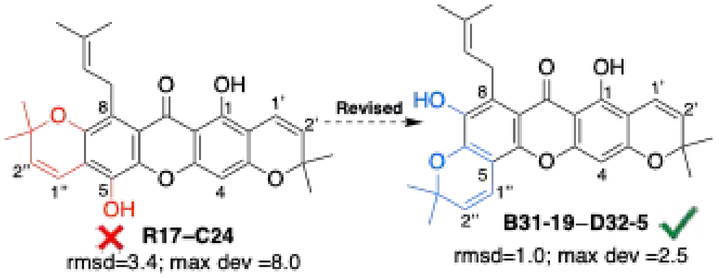

VIII.17a. Garcimangosone A (R17–C24)[80]

No	type	δcal	δexp	diff
C1	C	161.5	160.6	0.9
C2	C	105.2	105	0.2
C3	C	161.2	158.6	2.6
C4	CH	93.3	94.6	-1.3
C4a	C	156.2	157	-0.8
C5	C	139.2	141.5	-2.3
C6	C	116	108	8
C7	C	148.5	147.9	0.6
C8	C	124.1	128.9	-4.8
C8a	C	120.1	113	7.1
C9	C	185.4	183.4	2
C9a	C	105.2	104.3	0.9
C10a	C	139.3	146.9	-7.6
C1'	CH	120.5	116	4.5
C2'	CH	123.5	128.4	-4.9
C3'	C	78.4	78.7	-0.3
C4'[2]	CH3	28.2	28.4	-0.2
C1''	CH	120.3	115.8	4.5
C2''	CH	133.2	130.8	2.4
C3''	C	76.3	79.6	-3.3
C4''[2]	CH3	27.0	28.2	-1.2
C1'''	CH2	26.0	26.6	-0.6
C2'''	CH	126.9	124.2	2.7
C3'''	C	128.7	131.5	-2.8
C4'''	CH3	25.7	26.0	-0.3
C5'''	CH3	19.4	18.2	1.2
RMSD		3.37		
Max abs		8.03		

mol2 coordinates for lowest energy conformer

1	C1	0.402929897	0.476475580	-0.125482928
2	C2	0.356398901	0.736621114	1.248936335
3	O3	-0.794563715	0.792758849	1.988693649
4	C4	-1.996752905	0.579724356	1.402842235
5	C5	-2.076691713	0.285245696	0.032555453

6	C6	-0.883698570	0.193879970	-0.792956897
7	O7	-0.964618757	-0.121938432	-1.991611668
8	C8	-3.110698446	0.669486669	2.219149664
9	C9	-4.360577525	0.447699063	1.644381711
10	C10	-4.510495599	0.128896965	0.283818516
11	C11	-3.368606726	0.047777578	-0.516253442
12	C12	1.657245009	0.461414334	-0.782756883
13	C13	2.794343798	0.661044382	-0.004588177
14	C14	2.746024265	0.916584744	1.378353726
15	C15	1.508973826	0.959357647	2.005035432
16	O16	-5.423941842	0.495499858	2.464661974
17	C17	-6.745682069	0.727694668	1.911050394
18	C18	-6.903213686	0.040275143	0.575651584
19	C19	-5.845863560	-0.197256583	-0.200870778
20	C20	1.852099887	0.201566645	-2.264058191
21	C21	2.057786989	-1.268993128	-2.519053037
22	O22	3.999903017	0.667487872	-0.633272219
23	O23	-3.522191799	-0.265470197	-1.801209638
24	C24	-6.924516470	2.240963289	1.761164366
25	C25	-7.701684227	0.153771191	2.949995176
26	C26	3.125561313	-1.870758124	-3.052322740
27	C27	3.157625573	-3.369393782	-3.227310779
28	C28	4.359079374	-1.160574588	-3.545490001
29	H29	-3.019681915	0.903494428	3.272172234
30	H30	-7.911931333	-0.213887383	0.262678822
31	H31	-5.940745798	-0.655451058	-1.179269479
32	H32	2.708257578	0.785308788	-2.602308312
33	H33	0.975146855	0.538056892	-2.816115809
34	H34	1.214559841	-1.891473068	-2.218126725
35	H35	-2.612559201	-0.299219354	-2.195412730
36	H36	-7.923425444	2.467132829	1.375736792
37	H37	-6.798839232	2.737221502	2.729450073
38	H38	-6.188636580	2.641204397	1.057514446
39	H39	-8.737739944	0.322680935	2.640767159
40	H40	-7.543701118	0.636980832	3.918943948
41	H41	-7.539346722	-0.921468524	3.062434381
42	H42	3.325946351	-3.641052493	-4.276944171
43	H43	2.224126209	-3.838176207	-2.902126108
44	H44	3.982113167	-3.812514885	-2.652169331
45	H45	5.263901401	-1.640544941	-3.147439392
46	H46	4.428999985	-1.227842621	-4.638666129
47	H47	4.387669115	-0.106866617	-3.262911291
48	O48	1.435133388	1.207643145	3.334672272
49	H49	0.501161142	1.201113894	3.586126687
50	C50	5.142356768	0.199657890	0.109981700
51	C51	4.000985457	1.145232836	2.086847657
52	H52	3.964835622	1.564569367	3.086288080
53	C53	5.148926722	0.827814960	1.486008771
54	H54	6.108343484	0.974772215	1.974325428
55	C55	5.072399782	-1.327719682	0.215197368
56	H56	4.177688219	-1.632882340	0.766302982
57	H57	5.950127708	-1.713157880	0.744322427
58	H58	5.031362164	-1.770848757	-0.784316839
59	C59	6.347386934	0.645827719	-0.708482946
60	H60	6.360784561	1.735364261	-0.802830157
61	H61	6.306693568	0.206804568	-1.708921381
62	H62	7.273527028	0.322383574	-0.222783264

4 lowest-energy conformers used for Boltzmann-averaged ^{13}C NMR data.
Avg. Energy: -1535.256498 au

VIII.17b. Mangostenone A (B31-19–D32-5)[28]

No	type	δcal	δexp	diff
C1	C	161.1 (-2.9)	158.6*	2.5 (-2.9)
C2	C	105.3	105.0	0.3
C3	C	160.9	160.6*	0.3
C4	CH	93.9	94.6	-0.7
C4a	C	156.6	157.0	-0.4
C5	C	108.8	108.0	0.8
C6	C	144.4	146.9	-2.5
C7	C	140.0	141.5	-1.5
C8	C	132.7 (-2.9)	128.9	3.8 (-2.9)
C8a	C	115.2	113	2.2
C9	C	184.7	183.4	1.3
C9a	C	105.2	104.3	0.9
C10a	C	147.3	147.9	-0.6
C1'	CH	121 (-3.7)	116	5 (-3.7)
C2'	CH	123.5 (+2.8)	128.4	-4.9 (+2.8)
C3'	C	78.0	78.7	-0.7
C4'[2]	CH3	27.9	28.4	-0.5
C1''	CH	120.6 (-3.7)	115.8	4.8 (-3.7)
C2''	CH	127.0 (+2.8)	130.8	-3.8 (+2.8)
C3''	C	78.8	79.6	-0.8
C4''[2]	CH3	27.2	28.2	-1
C1'''	CH2	27.0	26.6	0.4
C2'''	CH	124.7	124.2	0.5
C3'''	C	131.6	131.5	0.1
C4'''	CH3	18.6	18.2	0.4
C5'''	CH3	25.7	26.0	-0.3
RMSD		2.14		
Max abs		4.99		
RMSD+CFx		1.05		
Max abs+CFx		2.50		

mol2 coordinates for lowest energy conformer in **SI: V.32**
5 lowest-energy conformers used for Boltzmann-averaged ^{13}C NMR data.
Avg. Energy: -1535.258263 au

VIII.18. Venuloxanthone (R18–C40)[81] and its revised structure Thwaitesixanthone (A20-3–B31-17).[14]

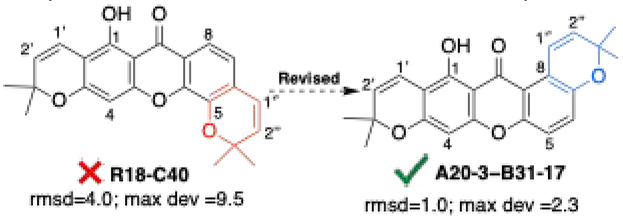

VIII.18a. Venuloxanthone (R18–C40)[81]

No	type	δcal	δexp	diff
C1	C	160.5	160.5	0
C2	C	102.0	104.3	-2.3
C3	C	160.8	157.9	2.9
C4	CH	93.8	94.3	-0.5
C4a	C	158.2	156.7	1.5
C5	C	142.2	151.7	-9.5
C6	C	122.6	119.9	2.7
C7	CH	121.2	117.8	3.4
C8	CH	118.3	124.3	-6
C8a	C	123.7	115.1	8.6
C9	C	182.3	183.4	-1.1
C9a	C	104.7	104.4	0.3
C10a	C	145.0	149.3	-4.3

C1'	CH	118.6	115.6	3
C2'	CH	121.9	127.3	-5.4
C3'	C	80.0	78.2	1.8
C4'[2]	CH3	31.0	28.4	2.6
C1''	CH	123.1	120.8	2.3
C2''	CH	129.6	132.7	-3.1
C3''	C	79.5	75.5	4
C4''[2]	CH3	31.2	27.3	3.9
RMSD		4.02		
Max abs		9.48		

mol2 coordinates for lowest energy conformer

1	C1	-1.756499079	0.002185751	-0.872485616
2	C2	-0.394210058	-0.000356037	-0.521728505
3	C3	0.612904904	-0.002220331	-1.471119593
4	C4	0.250107246	-0.001524032	-2.816765071
5	C5	-1.093955047	0.000953913	-3.227988121
6	C6	-2.090758003	0.002864304	-2.253222573
7	C7	-2.784292734	0.004118699	0.145777212
8	C8	-2.297866383	0.002901991	1.532203241
9	C9	-0.922515782	0.000331478	1.767313228
10	O10	0.003273858	-0.001173181	0.774237727
11	C11	-3.188563143	0.004142531	2.614700746
12	C12	-2.699047322	0.002752835	3.903094502
13	C13	-1.316055440	0.000124082	4.146835852
14	C14	-0.422434954	-0.001018403	3.078628026
15	C15	-0.741777304	-0.002110230	5.489099755
16	C16	0.579478617	-0.003170067	5.658433110
17	C17	1.561420893	-0.001566454	4.514189928
18	O18	0.917142139	-0.004172192	3.217560768
19	O19	1.261486434	-0.003368176	-3.697338240
20	C20	1.070477325	-0.002283110	-5.138122573
21	C21	-0.381982775	-0.000025022	-5.538134348
22	C22	-1.379563536	0.001497776	-4.655472161
23	C23	2.427229749	-1.262646350	4.553586358
24	C24	2.421605949	1.263460909	4.552479904
25	O25	-3.996994395	0.005586001	-0.119998528
26	O26	-3.366882143	0.005571010	-2.642280863
27	C27	1.766801408	-1.265770789	-5.646892287
28	C28	1.770426502	1.259767391	-5.645501745
29	H29	1.654550113	-0.004147671	-1.177510941
30	H30	-4.251697369	0.006116529	2.403171895
31	H31	-3.379233517	0.003555393	4.750133081
32	H32	-1.417307371	-0.002915857	6.339944438
33	H33	1.019563736	-0.004734300	6.652135599
34	H34	-0.573306916	0.000366183	-6.607761217
35	H35	-2.417006511	0.003156227	-4.970615308
36	H36	3.117560220	-1.265802378	3.704741264
37	H37	1.797543230	-2.155100555	4.498399910
38	H38	3.009379569	-1.300852077	5.480371015
39	H39	1.787948846	2.153017797	4.495767712
40	H40	3.002946614	1.305559163	5.479607655
41	H41	3.112448705	1.268606748	3.704053619
42	H42	-3.918745946	0.007367891	-1.819139290
43	H43	1.723614579	-1.310365005	-6.740143168
44	H44	2.816676299	-1.268629164	-5.337503339
45	H45	1.276136212	-2.155308748	-5.242154421
46	H46	1.727414140	1.305688582	-6.738705678
47	H47	1.282267371	2.150258686	-5.239822893
48	H48	2.820291071	1.259308262	-5.336060064

1 lowest-energy conformers used for Boltzmann-averaged ^{13}C NMR data.
Avg. Energy: -1264.224582 au

VIII.18b. Thwaitesixanthone (A20-3–B31-17)[14]

No	type	δcal	δexp	diff
C1	C	160.9 (-2.9)	157.9*	3.0 (-2.9)
C2	C	105.0	104.3	0.7
C3	C	161.5	160.5*	1.0
C4	CH	94.3	94.3	0
C4a	C	157.2	156.7	0.5
C5	CH	117.9	117.8	0.1
C6	CH	124.8	124.3	0.5
C7	C	149.3	149.3	0
C8	C	123.7 (-2.9)	119.9	3.8 (-2.9)
C8a	C	117.2	115.1	2.1
C9	C	185.7	183.4	2.3
C9a	C	105.5	104.4	1.1
C10a	C	151.1	151.7	-0.6
C1'	CH	120.7 (-3.7)	115.6	5.1 (-3.7)
C2'	CH	123.6 (+2.8)	127.3	-3.7 (+2.8)
C3'	C	78.3	78.2	0.1
C4'^{2}	CH3	28.0	28.4	-0.4
C1''	CH	124.2 (-3.7)	120.8	3.4 (-3.7)
C2''	CH	131.9 (+2.8)	132.7	-0.8 (+2.8)
C3''	C	75.6	75.5	0.1
C4''^{2}	CH3	27.0	27.3	-0.3
RMSD		1.98		
Max abs		5.09		
RMSD+CFx		0.97		
Max abs+CFx		2.33		

mol2 coordinates for lowest energy conformer in **SI: V.3**
1 lowest-energy conformers used for Boltzmann-averaged 13C NMR data.
Avg. Energy: -1264.676515 au

VIII.19. Tetrandraxanthone (R19–C40)[82] **and its revised structure toxyloxanthone E (A20-2).**[13]

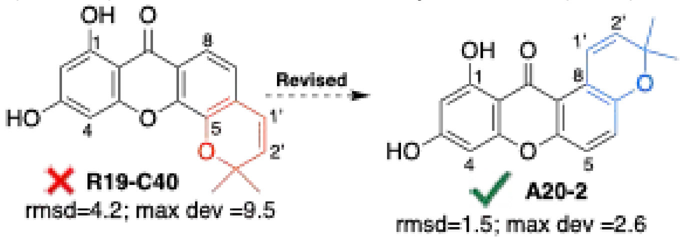

VIII.19a. Tetrandraxanthone (R19–C40)[82]

No	type	δcal	δexp	diff
C1	C	165.9	163.5	1.3
C2	CH	96.2	98.4	-2.2
C3	C	162.4	163.1	-0.7
C4	CH	92.5	93.6	-1.1
C4a	C	158.6	157.6	1.0
C5	C	142.2	151.7	-9.5
C6	C	122.8	121	1.8
C7	CH	121.4	117.9	3.5
C8	CH	118.4	124.6	-6.2
C8a	C	123.8	115.1	8.7
C9	C	182.4	183.6	-1.2
C9a	C	105.1	107.7	-2.6
C10a	C	145.1	149.6	-4.5
C1'	CH	123	120.2	2.8

C2'	CH	129.9	133	-3.1
C3'	C	79.5	75.7	3.8
C4'[2]	CH3	31.2	27.5	3.7
RMSD		4.19		
Max abs		9.54		

mol2 coordinates for lowest energy conformer

1	C1	-0.639343694	0.001840717	-2.900987725
2	C2	0.569007960	-0.001122813	-2.179750061
3	C3	1.804389140	-0.002792656	-2.803419910
4	C4	1.834754065	-0.001470423	-4.197961955
5	C5	0.664344697	0.001306110	-4.960341075
6	C6	-0.569024371	0.002905373	-4.320386253
7	C7	-1.914259510	0.003814027	-2.213727817
8	C8	-1.838596748	0.003496224	-0.746494894
9	C9	-0.585394911	-0.000434153	-0.132798695
10	O10	0.583554449	-0.002752506	-0.824201706
11	C11	-2.998708574	0.006132104	0.040350947
12	C12	-2.892687073	0.004720931	1.414446595
13	C13	-1.634530418	0.000158996	2.038268218
14	C14	-0.475433236	-0.002543592	1.266250282
15	C15	-1.463717448	-0.002827746	3.488149785
16	C16	-0.244490495	-0.005002357	4.024179117
17	C17	1.021172990	-0.003985775	3.204355590
18	O18	0.770500408	-0.008789864	1.778441179
19	O19	3.055980776	-0.002971177	-4.776648226
20	C20	1.839604448	-1.264549941	3.492157158
21	C21	1.833939790	1.261688576	3.486479444
22	O22	-3.001297930	0.005656632	-2.811986051
23	O23	-1.677521008	0.005488222	-5.059042022
24	H24	2.719305971	-0.005045611	-2.225515952
25	H25	-3.958841413	0.009088077	-0.462491754
26	H26	-3.784161452	0.006706285	2.035041830
27	H27	-2.352632813	-0.003173046	4.112587790
28	H28	-0.103564603	-0.006945080	5.101779864
29	H29	2.746652300	-1.267426063	2.879940942
30	H30	1.253353945	-2.157153327	3.255971155
31	H31	2.128032579	-1.303247755	4.547919400
32	H32	1.243902261	2.150617011	3.245632146
33	H33	2.121499613	1.306823469	4.542203645
34	H34	2.741311959	1.265603645	2.874761280
35	H35	-2.442826129	0.005000928	-4.431014712
36	H36	0.691079375	0.002297001	-6.045606118
37	H37	2.954645099	-0.001110446	-5.736541438

2 lowest-energy conformers used for Boltzmann-averaged ^{13}C NMR data.
Avg. Energy: -1070.521731 au

VIII.19b. Toxyloxanthone E (A20-2)[13]

No	type	δcal	δexp	diff
C1	C	165.9 (-2.9)	163.1*	2.8 (-2.9)
C2	CH	96.0	98.4	-2.4
C3	C	162.4	164.2*	-1.8
C4	CH	91.8	93.6	-1.8
C4a	C	157.9	157.6	0.3
C5	CH	117.9	117.9	0
C6	CH	124.8	124.6	0.2
C7	C	149.3	151.9	-2.6
C8	C	123.8 (-2.9)	121.0	2.8 (-2.9)
C8a	C	117.2	115.1	2.1
C9	C	185.5	183.6	1.9

C9a	C	105.3	107.7	-2.4
C10a	C	151.1	149.6	1.5
C1'	CH	124.2 (-3.7)	120.2	4 (-3.7)
C2'	CH	132.0 (+2.8)	133.0	-1 (+2.8)
C3'	C	75.5	75.7	-0.2
C4'[2]	CH3	26.9	27.5	-0.6
RMSD		1.95		
Max abs		4.04		
RMSD+CFx		1.47		
Max abs+CFx		2.58		

mol2 coordinates for lowest energy conformer in **SI: V.2**
2 lowest-energy conformers used for Boltzmann-averaged ^{13}C NMR data.
Avg. Energy: -1070.523469 au

VIII.20. Mesuaferrin B (R20–C41)[6] and its revised structure pyranojacareubin (B31-15–B34-6).[26]

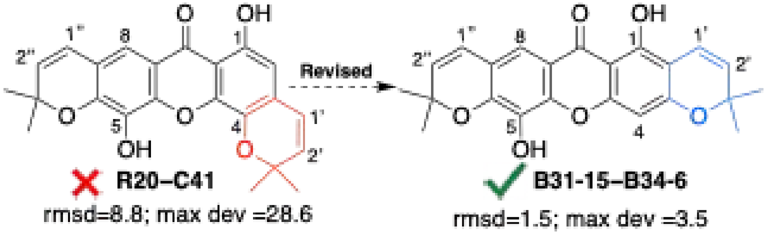

VIII.20a. Mesuaferrin B (R20–C41)[6]

No	type	δcal	δexp	diff
C1	C	156	157.8	-1.8
C2	CH	107.8	95.4	12.4
C3	C	125.9	104.9	21.0
C4	C	132.0	160.6	-28.6
C4a	C	144.0	156.9	-12.9
C5	C	133.2	132.2	1
C6	C	145.1	144.8	0.3
C7	C	115.4	117.9	-2.5
C8	CH	116.9	113.6	3.3
C8a	C	115.7	114.8	0.9
C9	C	183.1	180.3	2.8
C9a	C	110.6	103.3	7.3
C10a	C	146.2	145.2	1
C1'	CH	123.2	115.5	7.7
C2'	CH	132.0	127.6	4.4
C3'	C	77.9	78.3	-0.4
C4'[2]	CH3	31.0	28.5	2.5
C1''	CH	123.2	121.5	1.7
C2''	CH	127.2	131.1	-3.9
C3''	C	79.5	79.0	0.5
C4''[2]	CH3	30.9	28.6	2.3
RMSD		8.80		
Max abs		28.55		

mol2 coordinates for lowest energy conformer

1	C1	1.265743298	1.265996235	5.828985606
2	C2	1.266250390	-1.260772187	5.835521176
3	O3	-4.050713385	0.004304698	-0.154621454
4	C4	-0.997169653	-0.004133583	-4.166250943
5	C5	-2.388825055	-0.000451407	-4.061078156
6	C6	-0.194684863	-0.005572461	-3.023873313
7	C7	-3.007998372	0.001507863	-2.816396619

8	C8	-2.317255779	0.001643204	2.138315171
9	C9	0.433221404	-0.003682524	1.576466410
10	C10	-1.787751251	0.001585554	4.583341063
11	C11	-0.863268242	0.001558721	5.541321096
12	O12	0.928236124	-0.002881737	3.847945309
13	O13	0.013786858	-0.004619244	-0.697735229
14	C14	-1.401207491	0.000569760	3.175994853
15	C15	-0.018054170	-0.001868490	2.895041437
16	C16	-2.822548252	0.001760337	-0.317047871
17	C17	-0.812877350	-0.003186695	-1.778405696
18	C18	-0.516493229	-0.002665903	0.553463459
19	C19	-2.211577696	0.000097579	-1.645039635
20	C20	-1.888829097	0.000188498	0.807283473
21	C21	0.617412479	0.000983967	5.262386408
22	O22	-4.345176112	0.004641511	-2.753209416
23	O23	1.760180990	-0.006296576	1.308294595
24	O24	1.162921461	-0.011422433	-3.043340616
25	C25	1.906181911	-0.002861200	-4.281183281
26	C26	1.023560122	-0.006690643	-5.504085858
27	C27	-0.307381927	-0.007051606	-5.453660458
28	C28	2.763101854	1.265909806	-4.256835538
29	C29	2.779703190	-1.260178163	-4.260833833
30	H30	0.811050494	2.154858812	5.382782419
31	H31	1.132229734	1.311693842	6.914918342
32	H32	2.336852735	1.268148236	5.605666159
33	H33	2.337356126	-1.263629022	5.612193392
34	H34	1.132826656	-1.300845303	6.921687305
35	H35	0.811949843	-2.152139129	5.393972332
36	H36	-3.006944204	0.000650387	-4.952820088
37	H37	-3.384156047	0.003651012	2.337452956
38	H38	-2.847233665	0.002328356	4.823440494
39	H39	-1.140962496	0.002419441	6.591961259
40	H40	-4.587849244	0.005376620	-1.796885425
41	H41	1.860031261	-0.007495199	0.344753503
42	H42	1.548253167	-0.008066178	-6.456039091
43	H43	-0.905830925	-0.009090987	-6.360071685
44	H44	3.394138885	1.271519555	-3.362634759
45	H45	3.408294253	1.313769508	-5.140429154
46	H46	2.123409555	2.152907383	-4.241399600
47	H47	2.151752710	-2.155544277	-4.249536773
48	H48	3.409809182	-1.261015587	-3.365966604
49	H49	3.426533824	-1.295910352	-5.143807123

2 lowest-energy conformers used for Boltzmann-averaged ^{13}C NMR data.
Avg. Energy: -1339.890365 au

VIII.20b. Pyranojacareubin (B31-15–B34-6).[26]

No	type	δcal	δexp	diff
C1	C	160.7 (-2.9)	157.8	2.9 (-2.9)
C2	C	105.2	104.8	0.4
C3	C	161.3	159.4	1.9
C4	CH	95.3	95.4	-0.1
C4a	C	157.9	156.9	1
C5	C	135.2	132.1	3.1
C6	C	144.3	144.8	-0.5
C7	C	118.4	117.9	0.5
C8	CH	116.2 (-2.9)	113.6	2.6 (-2.9)
C8a	C	117.6	114.7	2.9
C9	C	182.3	178.8	3.5
C9a	C	104.9	103.8	1.1
C10a	C	145.5	145.0	0.5

C1'	CH	120.7(-3.7)	115.5	5.2(-3.7)
C2'	CH	123.7 (+2.8)	127.6	-3.9 (+2.8)
C3'	C	78.1	78.2	-0.1
C4'2	CH3	28.0	28.4	-0.4
C1''	CH	125.2 (-3.7)	121.5	3.7(-3.7)
C2''	CH	129.7 (+2.8)	131.1	-1.4 (+2.8)
C3''	C	78.1	79.0	-0.9
C4''2	CH3	27.1	28.5	-1.4
RMSD		2.25		
Max abs		5.19		
RMSD+CFx		1.45		
Max abs+CFx		3.54		

mol2 coordinates for lowest energy conformer in **SI: V.30**
2 lowest-energy conformers used for Boltzmann-averaged ^{13}C NMR data.
Avg. Energy: -1339.906692 au

VIII.20c. Rheediaxanthone A (B34-8–D31-9).[47]

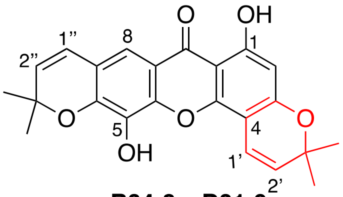

B34-8—D31-9

C	Type	Theory	Expt.	diff.
C1	C	165.3 (-2.9)	157.8	7.5 (-2.9)
C2	CH	99.5	95.4	4.1
C3	C	161.4	159.4	2.0
C4	C	101.7	104.8	-3.1
C4a	C	153.5	156.9	-3.4
C5	C	135.0	132.1	2.9
C6	C	144.0	144.8	-0.8
C7	C	118.1	117.9	0.2
C8	CH	116.5 (-2.9)	113.6	2.9 (-2.9)
C8a	C	117.9	114.7	3.2
C9	C	182.2	178.8	3.4
C9a	C	104.9	103.8	1.1
C10a	C	145.3	145.0	0.3
C1'	CH	120.0 (-3.7)	115.5	4.5 (-3.7)
C2'	CH	124.0 (+2.8)	127.6	-3.6 (+2.8)
C3'	C	78.1	78.2	-0.1
C4'2	CH3	27.9	28.4	-0.5
C1''	CH	125.2 (-3.7)	121.5	3.7 (-3.7)
C2''	CH	129.3 (+2.8)	131.1	-1.8 (+2.8)
C3''	C	78.4	79.0	-0.6
C4''2	CH3	27.4	28.5	-1.1
RMSD		2.89		
Max abs		7.53		
RMSD+CFx		2.15		
Max abs+CFx		5.13		

mol2 coordinates for lowest energy conformer in **SI: V.38**
2 lowest-energy conformers used for Boltzmann-averaged ^{13}C NMR data.
Avg. Energy: -1339.903028 au.

VIII.21. 6,8,10-Trihydroxy-2,2-dimethylpyrano[3,2-*c*]xanthen-7(2*H*)-one (R21–C41)[83] and its revised structure 7,9,12-trihydroxy-2,2-dimethyl-2*H*,6*H*-pyrano[3,2-*b*]xanthen-6-one (B34-2).[43]

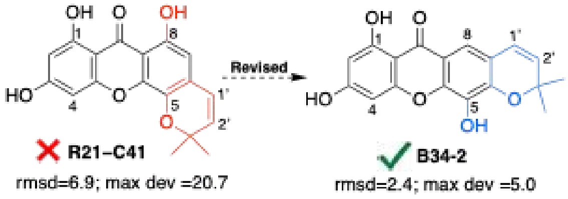

VIII.21a. 6,8,10-Trihydroxy-2,2-dimethylpyrano[3,2-c]xanthen-7(2H)-one (R21–C41)[83]

No	type	δcal	δexp	diff
C1	C	165.2	167.2*	-2
C2	CH	96.5	99.2	-2.7
C3	C	163	164.7*	-1.7
C4	CH	92.8	95.2	-2.4
C4a	C	158.8	159.3	-0.5
C5	C	132.4	147.3	-14.9
C6	C	126.8	119.8*	7
C7	CH	108.3	113.5*	-5.2
C8	C	155.3	134.5	20.8
C8a	C	109.2	115.6*	-6.4
C9	C	186.2	181.5	4.7
C9a	C	103.5	103.3	0.2
C10a	C	144	147.6	-3.6
C1'	CH	123	122.4	0.6
C2'	CH	132.8	132.7	0.1
C3'	C	78.1	79.2	-1.1
C4'[2]	CH3	31	28.4	2.6
RMSD		6.86		
Max abs		20.75		

mol2 coordinates for lowest energy conformer

1	C1	1.934096270	1.262875908	3.548407205
2	C2	1.934096270	-1.262875908	3.548407204
3	O3	-2.869024003	0.000000000	-2.793062929
4	O4	3.195383324	0.000000000	-4.717975169
5	O5	-1.532460399	0.000000000	-5.037493103
6	O6	0.892433021	0.000000000	1.824320570
7	C7	-1.367986340	0.000000000	3.513123498
8	C8	-0.151453132	0.000000000	4.055575563
9	C9	0.805239324	0.000000000	-4.916841688
10	C10	1.933037721	0.000000000	-2.753620387
11	C11	-2.896812654	0.000000000	0.066022596
12	O12	0.699950054	0.000000000	-0.784554186
13	C13	1.972068718	0.000000000	-4.148097338
14	C14	-2.781172639	0.000000000	1.449596179
15	C15	-0.431886648	0.000000000	-4.285408340
16	C16	-0.355890054	0.000000000	1.299335446
17	C17	-1.526160182	0.000000000	2.061903192
18	C18	-1.776095096	0.000000000	-2.174849487
19	C19	0.694920211	0.000000000	-2.136959703
20	C20	-1.721177880	0.000000000	-0.724320955
21	C21	-0.466454724	0.000000000	-0.088510154
22	C22	-0.510043302	0.000000000	-2.866341064
23	C23	1.122371392	0.000000000	3.248773414
24	H24	2.849220682	1.265626108	2.948879705
25	H25	1.350030139	2.153722052	3.300987617
26	H26	2.208186719	1.305804738	4.607811015
27	H27	1.350030139	-2.153722052	3.300987616

28	H28	2.208186719	-1.305804739	4.607811015
29	H29	2.849220682	-1.265626108	2.948879705
30	H30	3.102576107	0.000000000	-5.678328999
31	H31	-2.306112254	0.000000000	-4.426454342
32	H32	-2.261595580	0.000000000	4.130950664
33	H33	-0.020460215	0.000000000	5.134871136
34	H34	0.836993365	0.000000000	-6.002308487
35	H35	2.844847389	0.000000000	-2.170264667
36	O36	-4.119635435	0.000000000	-0.483000883
37	H37	-4.003256632	0.000000000	-1.458365050
38	H38	-3.685211076	0.000000000	2.050113592

2 lowest-energy conformers used for Boltzmann-averaged ^{13}C NMR data.
Avg. Energy: -1145.756873 au

VIII.21b. 7,9,12-Trihydroxy-2,2-dimethyl-2H,6H-pyrano[3,2-b]xanthen-6-one (B34-2)[43]

No	type	δcal	δexp	diff
C1	C	165.7 (-2.9)	164.7*	1.0 (-2.9)
C2	CH	96.4	99.2	-2.8
C3	C	162.2	167.2*	-5.0
C4	CH	92.5	95.2	-2.7
C4a	C	158.5	159.3	-0.8
C5	C	133.2	134.5*	-1.3
C6	C	144.0	147.3*	-3.3
C7	C	114.7	115.6*	-0.9
C8	CH	116.8 (-2.9)	113.5*	3.3 (-2.9)
C8a	C	116.7	119.8*	-3.1
C9	C	181.9	181.5	0.4
C9a	C	104.7	103.3	1.4
C10a	C	145.8	147.6	-1.8
C1'	CH	123.3 (-3.7)	122.4	0.9 (-3.7)
C2'	CH	126.8 (+2.8)	132.7	-5.9 (+2.8)
C3'	C	80.0	79.2	0.8
C4'[2]	CH3	30.8	28.4	2.4
RMSD		2.67		
Max abs		5.85		
RMSD+CFx		2.38		
Max abs+CFx		5.03		

mol2 coordinates for lowest energy conformer in **SI: V.34**
4 lowest-energy conformers used for Boltzmann-averaged ^{13}C NMR data.
Avg. Energy: -1145.751907au

VIII.22. Synthesis of angular D31-subtype pyranoxanthones (R22a-D31 – R22q-D31) under catalytic conditions, and revised linear B31-subtype pyranoxanthones (B31-1a – B31-1q).

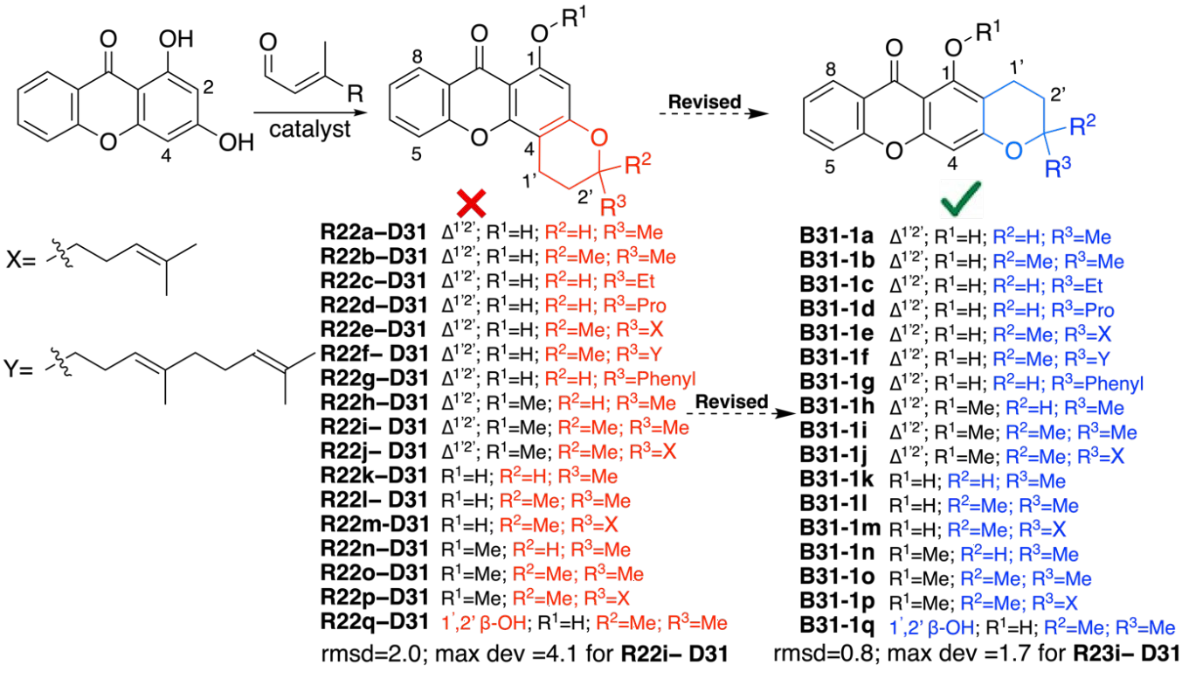

VIII.22-1. 6-Hydroxy-3,3-dimethyl-3H,7H-pyrano[2,3-c]xanthen-7-one (R22b–D31)[84] and its revised structure 5-Hydroxy-2,2-dimethyl-2H,6H-pyrano[3,2-b]xanthen-6-one (B31-1b).[58]

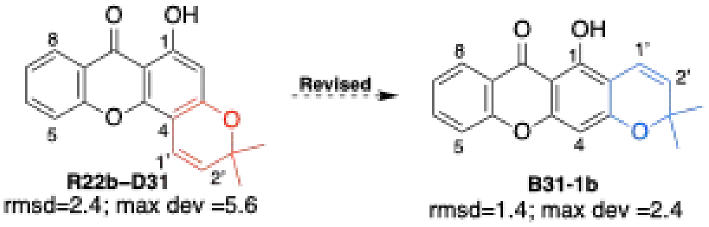

VIII.22-1a. 6-Hydroxy-3,3-dimethyl-3H,7H-pyrano[2,3-c]xanthen-7-one (R22b–D31)[84]

No	type	δcal	δexp	diff
C1	C	165.6 (-2.9)	161.0	4.6 (-2.9)
C2	CH	98.3	95.2	3.1
C3	C	160.9	157.8	3.1
C4	C	98.2	103.8	-5.6
C4a	C	153.1	157.2	-4.1
C5	CH	116.6	117.7	-1.1
C6	CH	133.7	134.9	-1.2
C7	CH	122.5	124	-1.5
C8	CH	129.0 (-2.9)	127.6	1.4 (-2.9)
C8a	C	122.3	120.6	1.7
C9	C	182.1	180.9	1.2
C9a	C	104.7	104.7	0.0
C10a	C	155.3	156	-0.7
C1'	CH	117.4 (-3.7)	115.5	1.9 (-3.7)
C2'	CH	122.8 (+2.8)	125.8	-3.0 (+2.8)
C3'	C	79.8	78.4	1.4
C4'[2]	CH3	30.9	28.5	2.4
RMSD		2.64		
Max abs		5.57		
RMSD		2.33		
Max abs		5.57		

mol2 coordinates for lowest energy conformer in **SI: V.63** (**D31-1**)[58]
1 lowest-energy conformers used for Boltzmann-averaged ^{13}C NMR data.
Avg. Energy: -995.298591 au

VIII.22-1b. 5-Hydroxy-2,2-dimethyl-2H,6H-pyrano[3,2-b]xanthen-6-one (B31-1b)[58]

No	type	δcal	δexp	diff
C1	C	160.6 (-2.9)	157.8	2.8 (-2.9)
C2	C	102.3	103.8	-1.5
C3	C	160.9	161.0	-0.1
C4	CH	93.8	95.2	-1.4
C4a	C	157.8	157.2	0.6
C5	CH	117.0	117.7	-0.7
C6	CH	133.7	134.9	-1.2
C7	CH	122.4	124	-1.6
C8	CH	128.9 (-2.9)	127.6	1.3 (-2.9)
C8a	C	122.2	120.6	1.6
C9	C	182.5	180.9	1.6
C9a	C	104.8	104.7	0.1
C10a	C	155.6	156	-0.4
C1'	CH	118.4 (-3.7)	115.5	2.9 (-3.7)
C2'	CH	122.3 (+2.8)	125.8	-3.5 (+2.8)
C3'	C	80.1	78.4	1.7
C4'[2]	CH3	31.0	28.5	2.5
RMSD		1.82		
Max abs		3.53		
RMSD+CFx		1.35		
Max abs+CFx		2.45		

mol2 coordinates for lowest energy conformer

1	C1	0.624627991	0.000000000	0.987613365
2	C2	-0.774352620	0.000000000	1.134150749
3	C3	-1.382682758	0.000000000	2.446902583
4	C4	-0.439556860	0.000000000	3.573733195
5	C5	0.935002978	0.000000000	3.324287159
6	O6	1.450788826	0.000000000	2.066502671
7	C7	1.236773163	0.000000000	-0.252741067
8	C8	0.426917482	0.000000000	-1.388514956
9	C9	-0.975395911	0.000000000	-1.305005235
10	C10	-1.569149365	0.000000000	-0.043389046
11	C11	-1.742005169	0.000000000	-2.542883594
12	C12	-1.114894080	0.000000000	-3.717831874
13	C13	0.385909550	0.000000000	-3.849837830
14	O14	1.067588896	0.000000000	-2.565968957
15	C15	0.862179883	1.262851926	-4.570032512
16	C16	0.862179883	-1.262851926	-4.570032512
17	O17	-2.900510378	0.000000000	0.036225180
18	H18	-2.824117705	0.000000000	-2.475731707
19	H19	-1.667335090	0.000000000	-4.653368281
20	H20	1.953869947	1.263951876	-4.647943634
21	H21	0.438746497	1.308927503	-5.578732829
22	H22	0.546171884	2.152792968	-4.018492052
23	H23	1.953869947	-1.263951877	-4.647943634
24	H24	0.438746496	-1.308927503	-5.578732829
25	H25	0.546171884	-2.152792968	-4.018492052
26	O26	-2.611239531	0.000000000	2.622864272
27	C27	-0.889011057	0.000000000	4.900576456
28	H28	-1.960385442	0.000000000	5.071473636
29	C29	1.856070007	0.000000000	4.372623609
30	C30	0.014314077	0.000000000	5.946575574

31	H31	-0.337001354	0.000000000	6.972981030
32	H32	2.915791866	0.000000000	4.142973272
33	C33	1.389141723	0.000000000	5.675488050
34	H34	2.101228704	0.000000000	6.494977475
35	H35	-3.133685492	0.000000000	0.998992952
36	H36	2.315231125	0.000000000	-0.343266624

1 lowest-energy conformers used for Boltzmann-averaged ^{13}C NMR data.
Avg. Energy: -995.299817 au

VIII.22-2. 6-Hydroxy-3-methyl-3*H*,7*H*-pyrano[2,3-*c*]xanthen-7-one (R22a–D31)[84] and its revised structure (B31-1a).

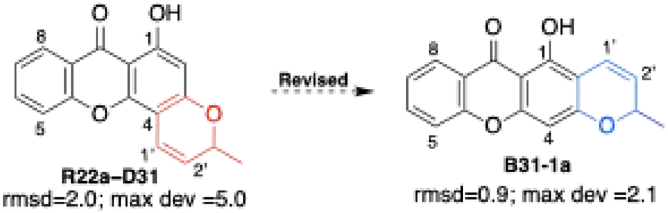

VIII.22-2a. 6-Hydroxy-3-methyl-3*H*,7*H*-pyrano[2,3-*c*]xanthen-7-one (R22a–D31)[84]

C	Type	Theory	Expt.	diff.
C1	C	165.6 (-2.9)	161.1	4.5 (-2.9)
C2	CH	99.8	94.8	5
C3	C	161.1	157.6	3.5
C4	C	101.4	103.8	-2.4
C4a	C	153.3	155.8	-2.5
C5	CH	116.8	117.5	0.7
C6	CH	133.9	134.8	-0.9
C7	CH	122.6	123.4	-0.8
C8	CH	129.1 (-2.9)	125.7	3.4 (-2.9)
C8a	C	122.2	120.5	1.7
C9	C	182.6	180.7	1.9
C9a	C	105.3	104.9	0.4
C10a	C	155.4	157.1	-1.7
C1'	CH	119.9 (-3.7)	117.5	2.4 (-3.7)
C2'	CH	120.4 (+2.8)	123.9	-3.5 (+2.8)
C3'	CH	72.6	72.8	-0.2
C4'	CH3	21.5	21.7	-0.2
RMSD		2.56		
Max abs		5.02		
RMSD+CFx		1.98		
Max abs+CFx		5.02		

mol2 coordinates for lowest energy conformer

1	O1	-0.279351563	1.446363844	-3.411209901
2	C2	0.572503718	-2.271783895	-0.137963803
3	C3	-0.339479988	4.789186563	0.346043209
4	C4	0.452630386	-2.290951140	-1.523729854
5	C5	-0.505082520	4.943400272	-1.036484181
6	C6	0.484632754	-1.083146244	0.612598893
7	C7	-0.114021178	3.540217000	0.899686599
8	C8	0.218570308	-1.102686654	-2.203943730
9	C9	-0.443329320	3.835320139	-1.860291664
10	O10	0.168968722	1.227806324	0.653545844
11	C11	-0.142883972	1.371479792	-2.181044211
12	C12	0.248294584	0.093320523	-0.091499234
13	C13	-0.053282131	2.429458468	0.057806622
14	C14	0.103299907	0.122999854	-1.489054589
15	C15	-0.216027879	2.561846327	-1.322214233
16	O16	0.092396199	-1.132744701	-3.528812991
17	C17	0.731953390	-1.149261641	2.047886824
18	C18	0.788089071	-2.340272086	2.646094702

19	C19	0.493243743	-3.590388471	1.861568060
20	O20	0.834641592	-3.444313527	0.469296069
21	C21	-0.970154335	-4.011723908	1.980327491
22	H22	-0.387054279	5.657279704	0.996261018
23	H23	0.532475684	-3.224432727	-2.066540617
24	H24	-0.680463238	5.928137276	-1.456363329
25	H25	0.016154660	3.401552583	1.967287538
26	H26	-0.566291965	3.914865164	-2.935303091
27	H27	-0.077392636	-0.205112205	-3.827611064
28	H28	0.900254323	-0.226033197	2.591232338
29	H29	0.999647886	-2.443156751	3.705621631
30	H30	1.137066058	-4.408387141	2.196173235
31	H31	-1.155431488	-4.918783697	1.397681156
32	H32	-1.221379580	-4.206987234	3.028053772
33	H33	-1.623196914	-3.213068614	1.614901490

1 lowest-energy conformers used for Boltzmann-averaged ^{13}C NMR data.
Avg. Energy: -955.978189 au

VIII.22-2b. B-31-1a (Previously unreported product)

C	Type	Theory	Expt.	diff.
C1	C	160.9 (-2.9)	157.6	3.3 (-2.9)
C2	C	105.4	104.9	0.5
C3	C	161	161.1	-0.1
C4	CH	95.2	94.8	0.4
C4a	C	157.7	157.1	0.6
C5	CH	117	117.5	-0.5
C6	CH	133.9	134.8	-0.9
C7	CH	122.5	123.4	-0.9
C8	CH	129 (-2.9)	125.7	3.3 (-2.9)
C8a	C	122.1	120.5	1.6
C9	C	182.8	180.7	2.1
C9a	C	105.3	103.8	1.5
C10a	C	155.6	155.8	-0.2
C1'	CH	120.9 (-3.7)	116.7	4.2 (-3.7)
C2'	CH	119.9 (+2.8)	123.9	-4 (+2.8)
C3'	CH	72.9	72.8	0.1
C4'	CH3	21.8	21.7	0.1
RMSD		1.99		
Max abs		4.21		
RMSD+CFx		0.92		
Max abs+CFx		2.15		

mol2 coordinates for lowest energy conformer

1	O1	-1.653371388	1.289980942	-2.484172751
2	C2	0.008793466	-1.473729520	1.371959706
3	C3	1.253931853	5.123782575	-1.022172202
4	C4	-0.915241756	-1.785115800	0.357975416
5	C5	0.364508920	4.989377284	-2.096532715
6	C6	0.639191473	-0.233859959	1.452314173
7	C7	1.453735795	4.084046521	-0.130397565
8	C8	-1.194958016	-0.815592151	-0.605692970
9	C9	-0.321931753	3.802958343	-2.270896860
10	O10	0.984901876	1.904995898	0.592549608
11	C11	-0.853591238	1.468031570	-1.551742976
12	C12	0.343586650	0.711479358	0.485799129
13	C13	0.754014041	2.890794053	-0.315015367
14	C14	-0.562156573	0.455018239	-0.560122352
15	C15	-0.135051770	2.737020456	-1.381180792
16	O16	-2.074740042	-1.102462857	-1.565006388
17	C17	-1.597716330	-3.072557894	0.416677792

18	C18	-1.170637874	-4.001677508	1.273065778
19	C19	0.065877359	-3.759895119	2.095889717
20	O20	0.282244494	-2.357128378	2.349300499
21	C21	1.314842054	-4.352887904	1.445553212
22	H22	1.795269550	6.054320552	-0.882140442
23	H23	0.217606750	5.813115201	-2.786905879
24	H24	2.137032437	4.170293945	0.707047605
25	H25	-1.018269344	3.661315125	-3.090688747
26	H26	-2.146461570	-0.298271768	-2.141094336
27	H27	-1.661775976	-4.964559969	1.371685025
28	H28	-0.057394919	-4.182719850	3.097229514
29	H29	2.196440941	-4.160332800	2.063669852
30	H30	1.198095760	-5.434617386	1.324303621
31	H31	1.469471779	-3.912374264	0.455384442
32	H32	1.343226184	-0.021315694	2.246267764
33	H33	-2.459472833	-3.227431242	-0.222910513

1 lowest-energy conformers used for Boltzmann-averaged ^{13}C NMR data.
Avg. Energy: -955.979322 au

VIII.22-3. 6-Methoxy-3,3-dimethyl-3*H*,7*H*-pyrano[2,3-*c*]xanthen-7-one (R21i–D31)[32,84] and its revised structure 5-Methoxy-2,2-dimethyl-2*H*,6*H*-pyrano[3,2-*b*]xanthen-6-one (B31-1i).[85]

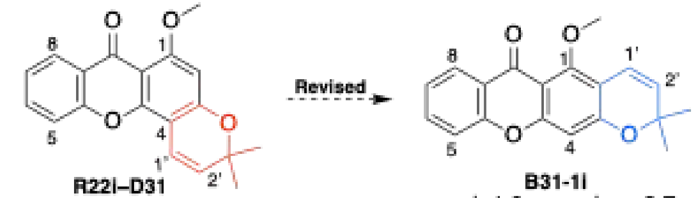

VIII.22-3a. 6-Methoxy-3,3-dimethyl-3*H*,7*H*-pyrano[2,3-*c*]xanthen-7-one (R22i–D31)[84]

C	Type	Theory	Expt.	diff.
C1	C	162.7 (-2.9)	159.2	3.5 (-2.9)
C2	CH	95.1	100.6	-5.5
C3	C	158.6	158.6	0
C4	C	100	112.2	-12.2
C4a	C	155.2	156.3	-1.1
C5	CH	116	117.1	-1.1
C6	CH	132.2	133.9	-1.7
C7	CH	122.3	122.5	-0.2
C8	CH	129.8 (-2.9)	126.5	3.3 (-2.9)
C8a	C	124.4	123.7	0.7
C9	C	175	175.1	-0.1
C10a	C	154.2	155	-0.8
C1'	CH	118.1 (-3.7)	115.9	2.2 (-3.7)
C2'	CH	122.4 (+2.8)	130.2	-7.8 (+2.8)
C3'	C	79.8	77.9	1.9
C4'2	CH3	31	28.3	2.7
C1''	CH3	55.2	62.6	-7.4
RMSD		4.60		
Max abs		12.2		
RMSD+CFx		4.20		
Max abs+CFx		12.20		

mol2 coordinates for lowest energy conformer [32]

1	C1	-0.259826724	0.000192103	0.699850228
2	C2	1.045046375	0.000697410	1.219116197
3	C3	1.255895005	0.000405598	2.678317098
4	C4	0.001348816	-0.001268044	3.474049246
5	C5	-1.241267290	-0.001052818	2.849422812
6	O6	-1.363103724	-0.000368465	1.494138984
7	C7	-0.544128253	0.000905024	-0.670191107

8	C8	0.538183176	0.001614447	-1.547976905
9	C9	1.856642528	0.002226306	-1.088581360
10	C10	2.113201059	0.001778698	0.276584145
11	C11	-1.888865430	0.001653208	-1.229917260
12	C12	-2.060663439	0.002752906	-2.551070441
13	C13	-0.915970211	0.002616667	-3.531360747
14	O14	0.383054750	0.002426936	-2.881978018
15	C15	-0.947018993	1.265236837	-4.395139518
16	C16	-0.947807895	-1.259944464	-4.395226519
17	O17	3.354884960	0.004683849	0.779173527
18	H18	2.646792866	0.003851428	-1.826673717
19	H19	-2.735146387	0.001823117	-0.552703198
20	H20	-3.054165766	0.003708142	-2.990341803
21	H21	-0.099118169	1.268893926	-5.087087484
22	H22	-1.873996566	1.307130366	-4.976348009
23	H23	-0.891180708	2.155943851	-3.763053354
24	H24	-0.099733474	-1.264278149	-5.086970922
25	H25	-1.874725387	-1.301101836	-4.976648772
26	H26	-0.892896361	-2.150722602	-3.763121707
27	O27	2.342893813	0.001548158	3.234222403
28	C28	0.049925221	-0.002169925	4.872793191
29	H29	1.029506256	-0.002320899	5.339067993
30	C30	-2.426906809	-0.001489629	3.583925489
31	C31	-1.114943900	-0.002668472	5.618543523
32	H32	-1.070466106	-0.003154103	6.702585345
33	H33	-3.375771600	-0.001166933	3.058807164
34	C34	-2.354769394	-0.002232849	4.967352437
35	H35	-3.272116163	-0.002410651	5.548189496
36	C36	4.448983990	-0.006175162	-0.111625810
37	H37	4.444761021	-0.904440772	-0.740785193
38	H38	5.337781599	-0.010174709	0.519375065
39	H39	4.455687313	0.887051507	-0.748712501

2 lowest-energy conformers used for Boltzmann-averaged ^{13}C NMR data.
Avg. Energy: -1034.578873 au

VIII.22-3b. 5-Methoxy-2,2-dimethyl-2*H*,6*H*-pyrano[3,2-*b*]xanthen-6-one (B31-1i).[85]

C	Type	Theory	Expt.	diff.
C1	C	159.5 (-2.9)	156.3	3.2 (-2.9)
C2	C	113.6	112.2	1.4
C3	C	159.6	159.2	0.4
C4	CH	101.4	100.6	0.8
C4a	C	159	158.6	0.4
C5	CH	116.6	117.1	-0.5
C6	CH	132.9	133.9	-1
C7	CH	122.3	122.5	-0.2
C8	CH	129.7 (-2.9)	126.5	3.2 (-2.9)
C8a	C	123.8	123.7	0.1
C9	C	176.6	175.1	1.5
C9a	C	114.2	115.9	-1.7
C10a	C	154.8	155	-0.2
C1'	CH	121 (-3.7)	115.9	5.1 (-3.7)
C2'	CH	127.2 (+2.8)	130.2	-3 (+2.8)
C3'	C	77.8	77.9	-0.1
C4'[2]	CH3	27.8	28.3	-0.5
C1''	CH3	61.7	62.6	-0.9
RMSD		1.84		
Max abs		5.09		

RMSD+CFx	0.82
Max abs+CFx	1.70

mol2 coordinates for lowest energy conformer

1	C1	-0.578133694	-0.816333202	0.893430338
2	C2	0.133130198	0.364577932	1.177017291
3	C3	0.589520702	0.626395964	2.554551601
4	C4	0.177018169	-0.399463908	3.539351258
5	C5	-0.523677845	-1.534077998	3.137360467
6	O6	-0.870623055	-1.746067452	1.840432571
7	C7	-1.029976556	-1.134454640	-0.382168245
8	C8	-0.790914909	-0.243875741	-1.413969561
9	C9	-0.120223792	0.974331055	-1.183792587
10	C10	0.332808444	1.263520391	0.099507715
11	C11	-0.011843458	1.909319182	-2.298715203
12	C12	-0.306690821	1.495400585	-3.531789700
13	C13	-0.667025010	0.053607859	-3.800908575
14	O14	-1.274936401	-0.551341344	-2.632055844
15	C15	0.584827829	-0.756730408	-4.152355040
16	C16	-1.729315077	-0.065378321	-4.887386735
17	O17	0.919685683	2.474630848	0.288723898
18	H18	-0.262230627	2.165683854	-4.385609007
19	H19	1.060801221	-0.349546823	-5.050290599
20	H20	1.308623572	-0.712894883	-3.332324503
21	H21	0.320159689	-1.802959834	-4.336163823
22	H22	-2.621745007	0.503115439	-4.611193637
23	H23	-2.009458897	-1.113409255	-5.027395540
24	H24	-1.343979261	0.322634009	-5.835677652
25	O25	1.261820529	1.588969368	2.895835484
26	C26	0.504129721	-0.255055285	4.893542759
27	H27	1.054582661	0.633889061	5.183921603
28	C28	-0.901565287	-2.516895203	4.053148092
29	C29	0.134321412	-1.218032939	5.813971441
30	H30	0.388832908	-1.099541116	6.862095780
31	H31	-1.446363727	-3.386049628	3.701117281
32	C32	-0.570384421	-2.350817125	5.387071875
33	H33	-0.862187441	-3.110224655	6.106246557
34	C34	2.340854297	2.464182725	0.271103272
35	H35	2.734441065	1.852190265	1.085697654
36	H36	2.652371826	3.499315705	0.417422658
37	H37	2.709071146	2.099664739	-0.697015984
38	H38	-1.563116110	-2.060259943	-0.559051766
39	H39	0.277390326	2.931980726	-2.083685596

1 lowest-energy conformers used for Boltzmann-averaged ^{13}C NMR data.
Avg. Energy: -1034.578956 au

VIII.23. Dehydrocycloguanandin B (R23–D31)[86] and its revised structure 6-deoxyjacareubin (B31-3).[26]

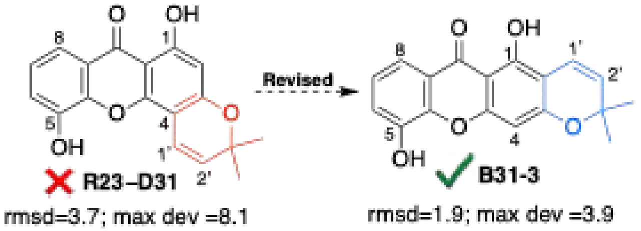

VIII.23a. Dehydrocycloguanandin B (R23–D31)[86]

No	type	δcal	δexp	diff
C1	C	165.9	157.8	8.1
C2	CH	98.6	95.8	2.8
C3	C	160.7	161.8	-1.1

C4	C	97.6	105.4	-7.8
C4a	C	152.2	158.6	-6.4
C5	C	144.9	147.0	-2.1
C6	CH	119.2	121.6	-2.4
C7	CH	124.2	125.2	-1.0
C8	CH	118.5	116.4	2.1
C8a	C	122.9	122.2	0.7
C9	C	182.4	182.0	0.4
C9a	C	104.6	104.3	0.3
C10a	C	143.3	146.1	-2.8
C1'	CH	116.5	115.7	0.8
C2'	CH	123.4	129.1	-5.7
C3'	C	79.8	79.3	0.5
C4'[2]	CH3	30.8	28.6	2.2
RMSD		3.67		
Max abs		8.12		

mol2 coordinates for lowest energy conformer

1	C1	0.569780813	-1.262845662	-4.514929287
2	C2	0.569844484	1.262818174	-4.514758981
3	O3	-2.975555360	0.000219082	2.914474344
4	C4	1.446471499	-0.000138929	-1.333826200
5	C5	1.646327950	-0.000154624	-2.650866173
6	C6	1.642975398	-0.000052577	4.934825072
7	C7	-2.308891176	0.000062728	-1.279668467
8	C8	1.778958603	-0.000081425	3.555804275
9	C9	-2.594169444	0.000119698	0.078947692
10	O10	-0.789350424	-0.000097592	-3.029824357
11	O11	0.836928855	-0.000042801	1.418030555
12	C12	-0.983123381	-0.000022978	-1.703753532
13	C13	0.372438757	0.000044724	5.520125528
14	C14	0.093414230	-0.000051075	-0.797276369
15	C15	-0.766607663	0.000110029	4.735216431
16	C16	-1.823768339	0.000135718	2.458738666
17	C17	0.620647525	-0.000010290	2.765991559
18	C18	-1.540316627	0.000084228	1.036136421
19	C19	-0.218575946	-0.000002655	0.557379539
20	C20	-0.645916254	0.000082137	3.340414530
21	C21	0.522023925	-0.000070816	-3.652721463
22	O22	3.009701821	-0.000175054	2.991850834
23	O23	-3.867585157	0.000207554	0.466420257
24	H24	-0.265862051	-1.265742217	-5.221243994
25	H25	1.506718032	-1.304364525	-5.080138992
26	H26	0.502538661	-2.153865979	-3.884519597
27	H27	-0.265776629	1.265836860	-5.221098229
28	H28	1.506799991	1.304386286	-5.079935489
29	H29	0.502614263	2.153755563	-3.884230368
30	H30	2.284536650	-0.000186046	-0.645571004
31	H31	2.649191420	-0.000211493	-3.068710416
32	H32	2.542788062	-0.000107014	5.540893361
33	H33	-3.113583335	0.000086286	-2.004269731
34	H34	0.287688049	0.000065566	6.601920952
35	H35	-1.762121792	0.000183273	5.164368370
36	H36	2.901666647	-0.000203480	2.031086673
37	H37	-3.872852055	0.000229326	1.454717591

2 lowest-energy conformers used for Boltzmann-averaged ^{13}C NMR data.
Avg. Energy: -1070.524456 au

VIII.23b. 6-Deoxyjacareubin (B31-3)[26]

No	type	δcal	δexp	diff

C1	C	160.9 (-2.9)	158.6	2.3 (-2.9)
C2	C	102.6	105.4	-2.8
C3	C	160.9	161.8	-0.9
C4	CH	93.0	95.8	-2.8
C4a	C	156.7	157.8	-1.1
C5	C	144.9	147.0	-2.1
C6	CH	119.1	121.6	-2.5
C7	CH	124.2	125.2	-1.0
C8	CH	118.2 (-2.9)	116.4	1.8 (-2.9)
C8a	C	122.7	122.2	0.5
C9	C	182.6	182.0	0.6
C9a	C	104.5	104.3	0.2
C10a	C	143.4	146.1	-2.7
C1'	CH	118.3 (-3.7)	115.7	2.6 (-3.7)
C2'	CH	122.4 (+2.8)	129.1	-6.7 (+2.8)
C3'	C	80.4	79.3	1.1
C4'^{2}	CH3	30.9	28.6	2.3
RMSD		2.45		
Max abs		6.67		
RMSD+CFx		1.92		
Max abs+CFx		3.87		

mol2 coordinates for lowest energy conformer in **SI: IV.22**

2 lowest-energy conformers used for Boltzmann-averaged ^{13}C NMR data.

Avg. Energy: -1070.140445 au

VIII.24. Globulixanthone C (R24–D31)[99] and its revised structure (B31-5).

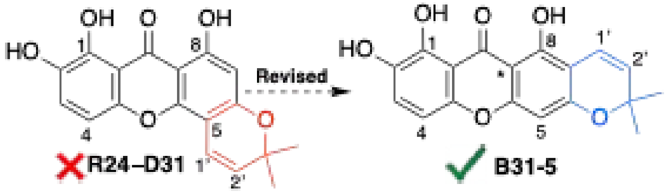

VIII.24a. Globulixanthone C (R24–D31)[99]

No	type	δcal	δexp	diff
C1	C	147.7	143.5	4.2
C2	C	140.7	137.3	3.4
C3	CH	121.7	123.9	-2.2
C4	CH	106	109.7	-3.7
C4a	C	147.6	151.7	-4.1
C5	C	98.6	104.1	-5.5
C6	C	162.0	156.1	5.9
C7	CH	98.2	95	3.2
C8	C	165	160.8	4.2
C8a	C	102.9	102	0.9
C9	C	186.6	184.1	2.5
C9a	C	108.7	128.8	-20.1
C10a	C	153.6	156	-2.4
C1'	CH	117.2	114.2	3
C2'	CH	123	128.8	-5.8
C3'	C	80.2	78.7	1.5
C4'^{2}	CH3	30.8	28	2.8
RMSD		5.94		
Max abs		20.11		

mol2 coordinates for lowest energy conformer

1	C1	0.401507498	1.262833073	-4.825205991
2	C2	0.401507503	-1.262833072	-4.825205991

3	O3	-2.588649182	0.000000000	2.843987690
4	O4	0.889191342	0.000000000	6.561229618
5	O5	-3.670112296	0.000000000	0.459936750
6	O6	-1.413284629	0.000000000	5.184688452
7	C7	1.506080606	0.000000000	-1.715435061
8	C8	1.608905653	0.000000000	-3.043353143
9	C9	2.137252644	0.000000000	4.532304075
10	C10	2.192705901	0.000000000	3.140072796
11	C11	-2.240263736	0.000000000	-1.391631651
12	O12	-0.849362039	0.000000000	-3.245855500
13	O13	1.099604203	0.000000000	1.060872624
14	C14	0.922527384	0.000000000	5.206268302
15	C15	-2.426098135	0.000000000	-0.018172716
16	C16	-0.946114676	0.000000000	-1.910163425
17	C17	-0.269740869	0.000000000	4.475483123
18	C18	0.193271912	0.000000000	-1.085727742
19	C19	-1.461042631	0.000000000	2.287389629
20	C20	1.008728184	0.000000000	2.422041085
21	C21	-0.013437554	0.000000000	0.288948243
22	C22	-1.302681762	0.000000000	0.858155044
23	C23	-0.236574091	0.000000000	3.070096797
24	C24	0.415115479	0.000000000	-3.961725844
25	H25	-0.480249175	1.266013108	-5.473409861
26	H26	1.298030722	1.304250197	-5.452472339
27	H27	0.377614079	2.153782507	-4.191339593
28	H28	-0.480249167	-1.266013116	-5.473409864
29	H29	1.298030732	-1.304250184	-5.452472334
30	H30	0.377614088	-2.153782509	-4.191339596
31	H31	-0.042240219	0.000000000	6.827698345
32	H32	-3.611551664	0.000000000	1.443434309
33	H33	-2.155773058	0.000000000	4.535433711
34	H34	2.386152219	0.000000000	-1.082322139
35	H35	2.578133461	0.000000000	-3.533870677
36	H36	3.049858168	0.000000000	5.118808919
37	H37	3.139617337	0.000000000	2.613602644
38	H38	-3.094024232	0.000000000	-2.057338691

1 lowest-energy conformers used for Boltzmann-averaged ^{13}C NMR data.
Avg. Energy: -1145.348397 au

VIII.24b. B31-5 (Unreported natural product)

No	type	δcal	δexp	diff
C1	C	147.6 (-2.9)	143.5	4.1 (-2.9)
C2	C	140.5	137.3	3.2
C3	CH	121.9	123.9	-2
C4	CH	106.4	109.7	-3.3
C4a	C	147.8	151.7	-3.9
C5	CH	94.1	95	-0.9
C6	C	162	160.8*	1.2
C7	C	102.2	104.1	-1.9
C8	C	159.8 (-2.9)	156.1*	3.7 (-2.9)
C8a	C	103	102	1
C9	C	186.6	184.1	2.5
C9a	C	108.7	128.8	-20.1
C10a	C	158.4	156	2.4
C1'	CH	118 (-3.7)	114.2	3.8 (-3.7)
C2'	CH	122.8 (+2.8)	128.8	-6 (+2.8)
C3'	C	80.4	78.7	1.7
C4’[2]	CH3	30.9	28	2.9
RMSD		5.59		
Max abs		20.10		

RMSD+CFx 5.24
Max abs+CFx 20.10
The signal assigned to C9a must be a mistake. If excluded:
RMSD+CFx 2.31
Max abs+CFx 3.90

mol2 coordinates for lowest energy conformer

1	C1	0.728574354	-1.262951298	-4.929136615
2	C2	0.729357287	1.262712269	-4.929538860
3	O3	-2.368355429	0.000620492	2.435494836
4	O4	0.036792766	-0.000685731	6.923747049
5	O5	-2.794573574	0.000972787	-0.142125889
6	O6	-1.834348546	0.000130888	5.000280842
7	C7	-1.768125266	0.000855002	-2.771917422
8	C8	-1.201223683	0.000662599	-3.976900987
9	C9	1.765298225	-0.000924832	5.284157511
10	C10	2.177104863	-0.000856532	3.953397288
11	C11	-0.939707883	0.000547563	-1.574290838
12	O12	1.036233814	0.000140192	-2.937472422
13	O13	1.659414754	-0.000415149	1.663395530
14	C14	0.417705603	-0.000603670	5.622684040
15	C15	-1.467309050	0.000613474	-0.285764051
16	C16	0.458109043	0.000179479	-1.729765953
17	C17	-0.546100429	-0.000180691	4.610065336
18	C18	1.325768959	-0.000125826	-0.638180175
19	C19	-1.134354151	0.000301724	2.189716502
20	C20	1.218418999	-0.000455774	2.953785241
21	C21	0.781986296	-0.000078335	0.633534248
22	C22	-0.611577166	0.000297074	0.850191573
23	C23	-0.152409441	-0.000102424	3.260062093
24	C24	0.290330902	0.000138353	-4.185722403
25	H25	0.439638163	-2.153081442	-4.363162378
26	H26	1.814904781	-1.265112873	-5.061975214
27	H27	0.254830369	-1.307503920	-5.915157118
28	H28	1.815692530	1.264158108	-5.062336623
29	H29	0.255688183	1.307233964	-5.915598598
30	H30	0.440950620	2.153200346	-4.363870063
31	H31	-0.931877418	-0.000440303	6.941110809
32	H32	-2.987600740	0.000942621	0.825187291
33	H33	-2.384400018	0.000396659	4.181576973
34	H34	-2.845471905	0.001302552	-2.651161123
35	H35	-1.800665305	0.000888019	-4.882985441
36	H36	2.496034560	-0.001247299	6.085882999
37	H37	3.227338098	-0.001109041	3.687760107
38	H38	2.397926835	-0.000419028	-0.784968097

1 lowest-energy conformers used for Boltzmann-averaged ^{13}C NMR data.
Avg. Energy: -1145.349250 au

VIII.25. inophyllin A (R25–D31)[87] and its revised structure inoxanthone also call blancoxanthone (B31-9).[2]

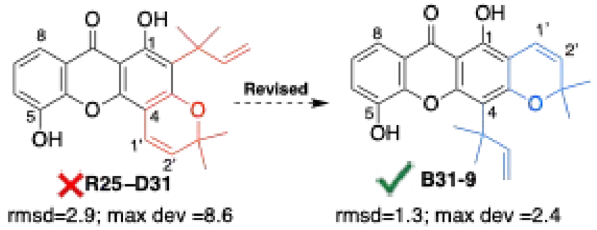

VIII.25a. Inophyllin A (R25–D31)[87]

No type δcal δexp diff

C1	C	165.20	156.60	8.60
C2	C	117.40	113.20	4.20
C3	C	161.30	159.40	1.90
C4	C	101.70	105.50	-3.80
C4a	C	151.10	153.90	-2.80
C5	C	144.90	145.30	-0.40
C6	CH	119.20	119.60	-0.40
C7	CH	124.20	124.20	0.00
C8	CH	118.60	116.00	2.60
C8a	C	122.70	120.40	2.30
C9	C	183.30	181.30	2.00
C9a	C	105.00	103.80	1.20
C10a	C	143.10	144.10	-1.00
C1'	CH	118.40	116.00	2.40
C2'	CH	125.70	127.30	-1.60
C3'	C	78.20	78.60	-0.40
C4'[2]	CH3	27.40	27.90	-0.50
C1''	C	42.00	41.30	0.70
C2''	CH	152.10	155.80	-3.70
C3''	CH2	109.10	104.00	5.10
C4''[2]	CH3	29.30	28.20	1.10
RMSD		2.86		
Max abs		8.61		

mol2 coordinates for lowest energy conformer

1	C1	-0.311626631	0.425509513	-4.560807953
2	C2	-1.712762485	-2.286937370	-2.196518659
3	C3	0.476911422	-3.243897146	-3.007150820
4	C4	0.476705958	3.017169485	-2.600363371
5	C5	2.041539234	1.100106417	-2.880064126
6	O6	0.175561456	3.292539886	2.602664356
7	O7	-0.725798865	-2.075664856	5.077668636
8	O8	0.517751733	3.090893109	0.079433011
9	C9	-0.462308348	0.910092501	-3.332084648
10	C10	0.038636289	-2.470406398	0.563778440
11	C11	-0.059824153	-3.195921521	-0.550406676
12	C12	-0.536811681	1.363948556	6.297436741
13	C13	-0.301653079	2.062480890	5.127257556
14	O14	0.424178076	-1.213580735	-1.842798428
15	O15	-0.257778553	-0.771513151	2.781335289
16	C16	-0.679756245	-0.027875205	6.284279727
17	C17	-0.585142255	-0.729455495	5.093447862
18	C18	0.349492636	1.773444321	0.166792068
19	C19	0.056038971	-1.014496292	0.481201690
20	C20	0.269788143	-0.413902398	-0.773816208
21	C21	0.397090548	0.972947989	-0.986780217
22	C22	0.040918169	2.059921264	2.641033802
23	C23	-0.203032762	1.367535403	3.914997219
24	C24	-0.341161284	-0.014816277	3.912250945
25	C25	-0.033944211	-0.174380366	1.578722328
26	C26	0.110480304	1.214450959	1.461430835
27	C27	-0.230006475	-2.502832524	-1.879797129
28	C28	0.621325638	1.485701406	-2.428214354
29	H29	-1.172153506	0.123505759	-5.151309634
30	H30	0.662920746	0.305450979	-5.025393514
31	H31	-2.185527369	-1.683527090	-1.414805516
32	H32	-2.230204045	-3.250729004	-2.242690661
33	H33	-1.821634788	-1.773920245	-3.156427614
34	H34	0.393011596	-2.669972342	-3.934541911
35	H35	1.535857151	-3.378957811	-2.769638124

36	H36	0.020726562	-4.227566150	-3.157087196
37	H37	-0.488855139	3.383138402	-2.241857876
38	H38	0.550210240	3.236762921	-3.670114593
39	H39	1.260469458	3.561897187	-2.073871169
40	H40	2.175691314	0.016012652	-2.905226662
41	H41	2.774041156	1.529338983	-2.188805340
42	H42	2.250878494	1.502707205	-3.877512410
43	H43	-0.657621297	-2.376263983	4.160909010
44	H44	0.427213735	3.467932335	0.993932903
45	H45	-1.469981044	1.001121964	-2.923560725
46	H46	0.143587419	-2.938284647	1.536921999
47	H47	-0.049523498	-4.281719448	-0.534472191
48	H48	-0.614922822	1.894670414	7.240552611
49	H49	-0.190686647	3.140651639	5.113246481
50	H50	-0.868309266	-0.583311687	7.196824219

4 lowest-energy conformers used for Boltzmann-averaged ^{13}C NMR data.
Avg. Energy: -1265.861227 au

VIII.25b. Inoxanthone also call blancoxanthone (B31-9).[2]

No	type	δcal	δexp	diff
C1	C	159.70 (-2.9)	156.60	3.10 (-2.9)
C2	C	106.70	105.50	1.20
C3	C	159.70	159.40	0.30
C4	C	111.60	113.20	-1.60
C4a	C	155.90	153.90	2.00
C5	C	146.40	145.30	1.10
C6	CH	119.70	119.60	0.10
C7	CH	124.30	124.20	0.10
C8	CH	117.30 (-2.9)	116.00	1.30 (-2.9)
C8a	C	122.20	120.40	1.80
C9	C	183.70	181.30	2.40
C9a	C	105.70	103.80	1.90
C10	C	143.80	144.10	-0.30
C1'	CH	121.20 (-3.7)	116.00	5.20 (-3.7)
C2'	CH	123.90 (+2.8)	127.30	-3.40 (+2.8)
C3'	C	77.90	78.60	-0.70
C4'[2]	CH3	27.60	27.90	-0.30
C1''	C	42.10	41.30	0.80
C2''	CH	157.10	155.80	1.30
C3''	CH2	106.00	104.00	2.00
C4''[2]	CH3	29.70	28.20	1.50
RMSD		1.90		
Max abs		5.18		
RMSD+CFx		1.29		
Max abs+CFx		2.42		

mol2 coordinates for lowest energy conformer in **SI: IV.27**
4 lowest-energy conformers used for Boltzmann-averaged ^{13}C NMR data.
Avg. Energy: -1265.866326 au

VIII.26. Buxixanthone (R26–D31)[88] and its revised structure calabaxanthone (B31-12).[17]

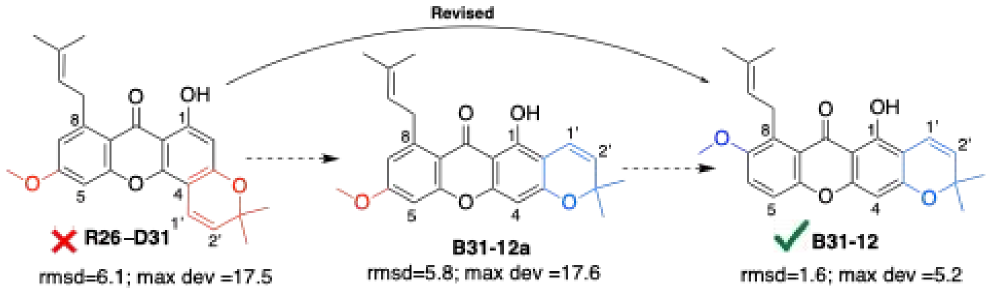

VIII.26a. Buxixanthone (R26–D31)[88]

No	type	δcal	δexp	diff
C1	C	165.7 (-2.9)	158.2	7.5 (-2.9)
C2	CH	99.60	94.1	5.5
C3	C	160.9	160.4	0.5
C4	C	100.4	104.3	-3.9
C4a	C	152.6	155.7	-3.1
C5	CH	98.50	116.0	-17.5
C6	C	162.2	153.6	8.6
C7	CH	114.3	118.7	-4.4
C8	C	149.9 (-2.9)	132.0	17.9 (-2.9)
C8a	C	114.2	115.9	-1.7
C9	C	183.8	183.4	0.4
C9a	C	105.3	104.3	1.0
C10a	C	159.3	151.7	7.6
C1'	CH	119.5 (-3.7)	115.8	3.7 (-3.7)
C2'	CH	124.0 (+2.8)	127.2	-3.2 (+2.8)
C3'	C	77.8	78.1	-0.3
C4'[2]	CH3	27.9	28.4	-0.5
C1''	CH2	36.5	25.7	10.8
C2''	CH	124.9	122.8	2.1
C3''	C	133.0	132.1	0.9
C4''	CH3	25.7	26.1	-0.4
C5''	CH3	19.1	18.2	0.9
C1'''	CH3	54.4	56.8	-2.4
RMSD		6.60		
Max abs		17.88		
RMSD+CFx		6.09		
Max abs+CFx		17.49		

mol2 coordinates for lowest energy conformer

1	O1	1.843238571	-1.989998714	0.160357374
2	C2	2.691179557	-1.330323361	2.784753629
3	C3	-0.333910129	0.632691967	3.968205426
4	C4	-0.721768598	-1.315464106	-3.123319802
5	C5	-1.124764995	0.730592859	2.834733001
6	C6	0.161196677	-1.573586748	-2.081352148
7	O7	-1.522951482	0.272583118	0.611282043
8	C8	-1.841942722	-0.524527743	-2.892255264
9	C9	0.881326854	-0.070149707	3.916418403
10	C10	-2.131904446	0.014913451	-1.625350327
11	C11	1.320033690	-0.682687013	2.759938537
12	C12	0.847718015	-1.262477838	0.312664824
13	C13	-0.683243527	0.109761783	1.666951175
14	C14	-0.072604932	-1.023411102	-0.788156412
15	C15	-1.225383436	-0.251525676	-0.603997506
16	C16	0.516106842	-0.610554763	1.586158940
17	O17	1.232349219	-2.326717439	-2.323294922
18	C18	3.722841322	-0.390187795	2.209306707
19	C19	4.912212310	-2.003462620	0.675381839

20	C20	4.673548991	-0.659996415	1.309691876
21	C21	5.618303457	0.422078109	0.849796312
22	C22	-3.389095889	0.730110373	-1.451601148
23	C23	-4.094390427	1.080015233	-2.528549856
24	C24	-3.549965333	0.829255821	-3.915105154
25	O25	-2.687196209	-0.335579324	-3.923057161
26	C26	-1.846470349	1.907524636	5.270732431
27	O27	-0.639031996	1.181771897	5.160288393
28	C28	-4.661716082	0.489103744	-4.899869103
29	C29	-2.726177854	2.028957980	-4.392879060
30	H30	2.939721823	-1.539974074	3.832979101
31	H31	2.677456245	-2.275988926	2.248062268
32	H32	-0.533993500	-1.724314129	-4.108125728
33	H33	-2.064770982	1.266177461	2.807391003
34	H34	1.480680232	-0.110250827	4.820266946
35	H35	1.727033421	-2.397700005	-1.468063042
36	H36	3.662143703	0.628962954	2.596454403
37	H37	5.941229539	-2.335503596	0.867730818
38	H38	4.226524886	-2.774592404	1.025071328
39	H39	4.796873135	-1.931016703	-0.412982443
40	H40	5.405194486	1.382840602	1.327801060
41	H41	6.659986631	0.153908790	1.069467380
42	H42	5.550749853	0.558835715	-0.237562642
43	H43	-3.741189480	0.932959818	-0.445887887
44	H44	-5.050996851	1.587714229	-2.447521629
45	H45	-1.893967156	2.253245576	6.303303327
46	H46	-1.854337153	2.772618759	4.596423163
47	H47	-2.714690766	1.271488575	5.058934903
48	H48	-4.240059726	0.268060083	-5.884408693
49	H49	-5.222407992	-0.383095379	-4.554319179
50	H50	-5.349355487	1.334681806	-4.996706559
51	H51	-1.896562366	2.217157088	-3.704583460
52	H52	-3.350771712	2.927244899	-4.429486964
53	H53	-2.322027885	1.837829085	-5.392110523

4 lowest-energy conformers used for Boltzmann-averaged ^{13}C NMR data.
Avg. Energy: -1305.179287 au

VIII.26b. B31-12a

C	Type	Theory	Expt.	diff.
C1	C	160.9 (-2.9)	158.2	2.7 (-2.9)
C2	C	105.5	104.3	1.2
C3	C	160.8	160.4	0.4
C4	CH	94.3	94.3	0.0
C4a	C	156.9	155.7	1.2
C5	CH	98.4	116.0	-17.6
C6	C	162.3	153.6	8.7
C7	CH	114.3	118.7	-4.4
C8	C	149.3 (-2.9)	132.0	17.3 (-2.9)
C8a	C	113.9	115.9	-2.0
C9	C	184.0	183.4	0.6
C9a	C	105.2	104.3	0.9
C10a	C	159.6	151.7	7.9
C1'	CH	120.9 (-3.7)	115.8	5.1 (-3.7)
C2'	CH	123.6 (+2.8)	127.2	-3.6 (+2.8)
C3'	C	78.1	78.1	0.0
C4'[2]	CH3	27.9	28.4	-0.5
C1''	CH2	36.4	25.7	10.7
C2''	CH	124.7	122.8	1.9
C3''	C	132.9	132.1	0.8
C4''	CH3	25.6	26.1	-0.5

C5''	CH3	18.9	18.2	0.7
C1'''	CH3	54.5	56.8	-2.3
RMSD		6.28		
Max abs		17.65		
RMSD+CFx		5.81		
Max abs+CFx		17.65		

mol2 coordinates for lowest energy conformer

1	O1	1.042598462	-0.095749173	-1.967355314
2	C2	0.978546711	2.538519070	-2.995328390
3	C3	0.610570324	4.494216207	0.237474101
4	C4	0.405226589	-2.807440114	1.205537910
5	C5	0.466966835	3.509747832	1.201679528
6	C6	0.607260459	-1.956110216	0.117476806
7	O7	0.371979556	1.290366143	1.816066944
8	C8	0.190380815	-2.241123278	2.474451553
9	C9	0.798608123	4.137012744	-1.108339077
10	C10	0.186395162	-0.864455141	2.680588325
11	C11	0.851165222	2.817922341	-1.510507438
12	C12	0.799892513	0.353035996	-0.833555291
13	C13	0.523086174	2.176328304	0.797303538
14	C14	0.590261781	-0.545814231	0.289639615
15	C15	0.386585986	-0.045436515	1.582832633
16	C16	0.718662969	1.787572615	-0.535685562
17	O17	0.820616423	-2.497915840	-1.081851703
18	C18	-0.370965063	2.233567770	-3.600142767
19	C19	0.266321753	0.217105283	-4.978639311
20	C20	-0.690781112	1.248046907	-4.443893748
21	C21	-2.102452137	1.106836259	-4.954727711
22	C22	0.496704734	-4.258812744	1.108772743
23	C23	0.130142655	-5.009942374	2.148131046
24	C24	-0.465485373	-4.375114802	3.383275217
25	O25	0.033842612	-3.023333756	3.558274404
26	C26	0.390552922	6.239373984	1.824677576
27	O27	0.579995886	5.817818860	0.488832369
28	C28	-1.990874033	-4.296080933	3.266785757
29	C29	-0.040319935	-5.107524678	4.650518402
30	H30	1.384915898	3.442696301	-3.466823760
31	H31	1.682214020	1.727340583	-3.168972598
32	H32	0.315079671	3.719863673	2.252246850
33	H33	0.897298985	4.937411777	-1.834585384
34	H34	0.965497008	-1.741894340	-1.708702085
35	H35	-1.159894847	2.925049275	-3.301260821
36	H36	0.303694743	0.270800286	-6.074332063
37	H37	1.280208973	0.326841626	-4.592889416
38	H38	-0.079988081	-0.791087836	-4.720040522
39	H39	-2.764894138	1.878256001	-4.550806767
40	H40	-2.130771340	1.169941445	-6.050026096
41	H41	-2.515856720	0.126087847	-4.685281272
42	H42	0.207110291	-6.093305423	2.128861091
43	H43	0.397966141	7.328908549	1.797986660
44	H44	-0.571868360	5.891581178	2.219669345
45	H45	1.201398004	5.884837423	2.472937873
46	H46	-2.417011964	-3.820858491	4.157333312
47	H47	-2.273747708	-3.714349117	2.382677455
48	H48	-2.414638970	-5.300598167	3.163395863
49	H49	1.050141415	-5.134292090	4.725947798
50	H50	-0.417229219	-6.135254692	4.635000742
51	H51	-0.442219941	-4.601473085	5.533414252
52	H52	0.026241885	-0.449498276	3.667192682

53 H53 0.890867241 -4.689620966 0.194764704

4 lowest-energy conformers used for Boltzmann-averaged ^{13}C NMR data.
Avg. Energy: -1305.180535 au

VIII.26c. Calabaxanthone (B31-12).[17]

No	type	δcal	δexp	diff
C1	C	161.10 (-2.9)	158.2	2.90 (-2.9)
C2	C	103.90	104.3	-0.40
C3	C	161.20	160.4	0.80
C4	CH	93.70	94.1	-0.40
C4a	C	157.30	155.7	1.60
C5	CH	115.60	116.0	-0.40
C6	CH	116.50	118.7	-2.20
C7	C	151.60	153.6	-2.00
C8	C	135.80 (-2.9)	132.0	3.80 (-2.9)
C8a	C	121.20	115.9	5.30
C9	C	185.50	183.4	2.10
C9a	C	105.10	104.3	0.80
C10a	C	150.90	151.7	-0.80
C1'	CH	120.10 (-3.7)	115.8	4.30 (-3.7)
C2'	CH	122.90 (+2.8)	127.2	-4.30 (+2.8)
C3'	C	78.80	78.1	0.70
C4'[2]	CH3	28.90	28.4	0.50
C1''	CH2	26.80	25.7	1.10
C2''	CH	124.90	122.8	2.10
C3''	C	131.20	132.1	-0.90
C4''	CH3	25.70	26.10	-0.40
C5''	CH3	18.80	18.20	0.60
C1'''	CH3	54.80	56.80	-2.00
RMSD		2.22		
Max abs		5.25		
RMSD+CFx		1.60		
Max abs+CFx		5.25		

mol2 coordinates for lowest energy conformer

1	O1	1.066001466	-0.062728331	-1.599999674
2	C2	1.158782521	2.635128849	-2.456622473
3	C3	0.355494022	4.376921931	0.823227759
4	C4	0.344080665	-2.975237947	1.359293355
5	C5	0.185268486	3.329890657	1.709927514
6	C6	0.581568924	-2.051911825	0.340511785
7	O7	0.135025643	1.070798219	2.214643313
8	C8	0.041878474	-2.496151349	2.646308064
9	C9	0.671964641	4.119394632	-0.512507203
10	C10	-0.019715180	-1.136363624	2.934861765
11	C11	0.837271607	2.808269007	-0.983871093
12	C12	0.767824288	0.313161373	-0.454764088
13	C13	0.328985407	2.025963438	1.260501188
14	C14	0.515381523	-0.653675674	0.596740625
15	C15	0.216060228	-0.241596777	1.904483044
16	C16	0.649365668	1.738742233	-0.078101641
17	O17	0.877369447	-2.514087338	-0.874286696
18	C18	-0.110880527	2.603982088	-3.272266210
19	C19	0.324214942	0.506387021	-4.612968913
20	C20	-0.483254218	1.704740275	-4.189013694
21	C21	-1.808955676	1.839151227	-4.897619277
22	C22	0.478509362	-4.414422270	1.176558357
23	C23	0.084712113	-5.239835193	2.147499645
24	C24	-0.578974835	-4.705865673	3.394482345
25	O25	-0.149629984	-3.346855777	3.669935209

26	C26	0.703847540	6.444573475	-1.027891890
27	O27	0.834859338	5.104824908	-1.438505210
28	C28	-2.101746447	-4.684541139	3.228169406
29	C29	-0.166337097	-5.495051834	4.631251804
30	H30	1.752861839	3.500806586	-2.765397975
31	H31	1.754696175	1.739917721	-2.607905983
32	H32	-0.062942189	3.504317110	2.750688041
33	H33	1.027382184	-1.718381482	-1.448156087
34	H34	-0.787299917	3.433855554	-3.069595358
35	H35	0.472413313	0.517014910	-5.699690448
36	H36	1.300627143	0.453327306	-4.131685318
37	H37	-0.210809059	-0.420550145	-4.373179634
38	H38	-2.367010613	2.718840720	-4.564438725
39	H39	-1.667736587	1.912212694	-5.983869871
40	H40	-2.433052956	0.952533404	-4.721848556
41	H41	0.189704789	-6.316937419	2.060974228
42	H42	0.891926737	7.046868986	-1.916559002
43	H43	-0.306683000	6.657755594	-0.657102042
44	H44	1.437112660	6.704605802	-0.252361966
45	H45	-2.575939135	-4.282507827	4.128858014
46	H46	-2.380019280	-4.062979851	2.372422181
47	H47	-2.479704100	-5.696257008	3.052368563
48	H48	0.921291113	-5.479936882	4.747166700
49	H49	-0.497084234	-6.534383258	4.542621784
50	H50	-0.621674191	-5.058948172	5.525509671
51	H51	-0.252729252	-0.789011844	3.933337266
52	H52	0.921593132	-4.777682880	0.255642449
53	H53	0.234103087	5.391915801	1.182224952

3 lowest-energy conformers used for Boltzmann-averaged ^{13}C NMR data.
Avg. Energy: -1305.173209 au

VIII.27. Musaxanthone (R27–D31)[89] and its revised structure garciosone E (B13-2).[33]

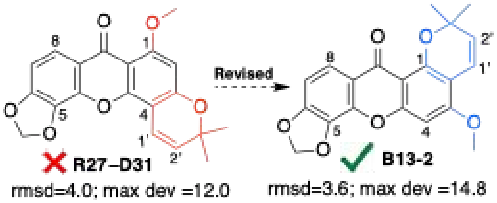

VIII.27a. Musaxanthone (R27–D31)[89]

No	type	δcal	δexp	diff
C1	C	162.9	159.4	3.5
C2	CH	96.4	91.4	5.0
C3	C	158.5	158.1	0.4
C4	C	100.8	107.2	-6.4
C4a	C	155.0	155.4	-0.4
C5	C	134.0	133.6	0.4
C6	C	151.3	155.4	-4.1
C7	CH	104.8	107.2	-2.4
C8	CH	124.2	121.4	2.8
C8a	C	120.7	119.3	1.4
C9	C	174.1	174.0	0.1
C9a	C	108.9	112.8	-3.9
C10a	C	140.2	152.3	-12.1
C1'	CH	118.2	115.8	2.4
C2'	CH	122.8	127.7	-4.9
C3'	C	79.6	79.1	0.5
C4'[2]	CH3	30.7	27.8	2.9
C1''	CH3	56.0	55.9	0.1

C1'''	CH2	105.4	102.9	2.5
RMSD		4.02		
Max abs		12.05		

mol2 coordinates for lowest energy conformer

1	O1	3.416061466	0.000000003	2.023224230
2	C2	0.330437287	-0.000000213	-2.050945757
3	C3	-0.535218691	0.000000146	5.021849840
4	C4	1.724359272	-0.000000167	-1.981134637
5	C5	0.815797505	0.000000208	5.329326409
6	C6	-0.459402040	-0.000000324	-0.902089992
7	C7	-1.007726254	-0.000000050	3.723824052
8	C8	2.355566169	-0.000000206	-0.743187926
9	C9	1.696052013	0.000000080	4.251235597
10	O10	-0.638576800	-0.000000290	1.401209506
11	C11	2.215231239	-0.000000175	1.805933555
12	C12	0.200062733	-0.000000345	0.329682963
13	C13	-0.130549439	-0.000000173	2.656285546
14	C14	1.597544016	-0.000000259	0.463508515
15	C15	1.243833727	-0.000000093	2.928290091
16	C16	-1.908038190	-0.000000334	-1.053830687
17	C17	-2.447111348	-0.000000194	-2.271933072
18	C18	-1.628102477	0.000000023	-3.537375281
19	O19	-0.197477096	-0.000000046	-3.286067390
20	C20	-1.907184945	1.262781592	-4.354853553
21	C21	-1.907184940	-1.262781270	-4.354853950
22	O22	-1.590959361	0.000000266	5.883285001
23	C23	-2.767304508	0.000000150	5.078258248
24	O24	-2.372746248	-0.000000033	3.709141918
25	C25	4.486342811	0.000000085	-1.777147116
26	O26	3.688921055	-0.000000068	-0.613208509
27	H27	2.275470981	-0.000000048	-2.911582057
28	H28	1.159252517	0.000000354	6.356645539
29	H29	2.769472410	0.000000123	4.405367477
30	H30	-2.524995017	-0.000000410	-0.162431634
31	H31	-3.524675277	-0.000000177	-2.411284266
32	H32	-1.298618920	1.265924790	-5.264691066
33	H33	-1.667549105	2.152940646	-3.766308869
34	H34	-2.963590131	1.306552445	-4.639700728
35	H35	-1.298618846	-1.265924254	-5.264691422
36	H36	-2.963590131	-1.306552107	-4.639701141
37	H37	-1.667549044	-2.152940458	-3.766309514
38	H38	-3.350710887	-0.902394196	5.289732815
39	H39	-3.350710830	0.902394585	5.289732546
40	H40	5.517288878	0.000000209	-1.423360261
41	H41	4.308248122	0.895600547	-2.384922465
42	H42	4.308248324	-0.895600356	-2.384922556

3 lowest-energy conformers used for Boltzmann-averaged ^{13}C NMR data.
Avg. Energy: -1223.115995 au

VIII.27b. Garciosone E (B13-2).[33]

C	Type	Theory	Expt.	diff.
C1	C	158.2(-2.9)	155.3	2.9(-2.9)
C2	C	107.7	107.2	0.5
C3	C	158.2	158.1	0.1
C4	CH	91.4	91.4	0
C4a	C	159.2	159.4	-0.2
C5	C	134.1	133.6	0.5
C6	C	151.3	152.3	-1
C7	CH	104.8	107.2	-2.4

C8	CH	124.3(-2.9)	121.4	2.9 (-2.9)
C8a	C	120.8	119.3	1.5
C9	C	174.1	174	0.1
C9a	C	109.7	112.8	-3.1
C10a	C	140.6	155.4	-14.8
C1'	CH	119.8(-3.7)	115.8	4 (-3.7)
C2'	CH	125.4(+2.8)	127.7	-2.3 (+2.8)
C3'	C	77.6	79.1	-1.5
C4'^{2}	CH3	27.3	27.8	-0.5
C1''	CH3	54.6	55.9	-1.3
C1'''	CH2	105.3	102.9	2.4
RMSD		3.77		
Max abs		14.80		
RMSD+CFx		3.56		
Max abs+CFx		14.80		

The signal assigned to C10a must be a mistake. If excluded:

RMSD+CFx	1.36
Max abs+CFx	3.0

mol2 coordinates for lowest energy conformer in **SI: IV.20**
3 lowest-energy conformers used for Boltzmann-averaged ^{13}C NMR data.
Avg. Energy: -1222.685511 au

VIII.28. Asmaxanthone (R28–D31)[89] and its revised structure (B13-3) (unreported natural product).

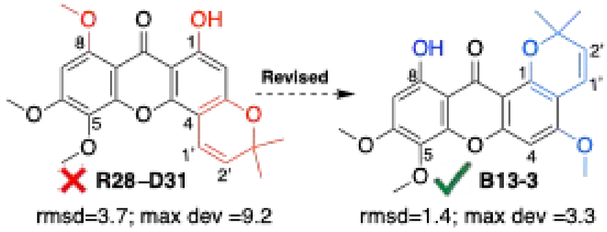

VIII.28a. Asmaxanthone (R28–D31).[89]

No	type	δcal	δexp	diff
C1	C	165.60	159.90	5.70
C2	CH	99.60	91.60	8.00
C3	C	160.60	151.40	9.20
C4	C	100.90	105.20	-4.30
C4a	C	152.40	158.00	-5.60
C5	C	131.40	130.10	1.30
C6	C	158.20	157.70	0.50
C7	CH	95.50	94.50	1.00
C8	C	158.20	157.30	0.90
C8a	C	109.20	104.70	4.50
C9	C	181.90	181.10	0.80
C9a	C	104.80	103.30	1.50
C10a	C	154.00	155.90	-1.90
C1'	CH	119.80	115.50	4.30
C2'	CH	123.90	127.10	-3.20
C3'	C	77.90	77.90	0.00
C4'^{2}	CH3	27.90	28.20	-0.30
C1''	CH3	60.20	61.50	-1.30
C1'''	CH3	55.70	56.40	-0.70
C1''''	CH3	56.50	56.20	0.30
RMSD		3.74		
Max abs		9.20		

mol2 coordinates for lowest energy conformer
1 O1 0.032824006 -0.473696476 -3.608953619

2	C2	-3.264089871	0.848774710	-0.072031323
3	C3	3.748467901	-0.406817375	-0.091942552
4	C4	-3.425784958	0.616754952	-1.433572176
5	C5	3.803170649	-0.682635401	-1.463343182
6	C6	-2.012744793	0.744152076	0.561190359
7	C7	2.538456848	-0.063806440	0.513797862
8	C8	-2.324526406	0.258497739	-2.204392526
9	C9	2.646072056	-0.633674945	-2.238626293
10	O10	0.263638897	0.290882294	0.401529330
11	C11	0.129355804	-0.240000006	-2.396204281
12	C12	-0.931575404	0.382877366	-0.234766464
13	C13	1.390366092	-0.029307567	-0.276140207
14	C14	-1.039835466	0.128612547	-1.605729603
15	C15	1.396576294	-0.305485867	-1.654162786
16	C16	-1.911484439	1.108349947	1.967700422
17	C17	-3.027786143	1.289522690	2.674946422
18	C18	-4.382589142	1.031007291	2.058063554
19	O19	-4.349415052	1.239298002	0.622903403
20	C20	-4.819562871	-0.415822007	2.310479951
21	C21	-5.422898791	2.023386797	2.564655632
22	O22	-2.500384323	0.030312161	-3.504551216
23	O23	2.649151674	-0.890412722	-3.555969009
24	C24	3.866004859	-1.213820771	-4.189020836
25	C25	6.082698792	-0.741245894	0.178017679
26	O26	4.815943600	-0.447732882	0.728489721
27	C27	2.247355098	-0.855132427	2.684808956
28	O28	2.478010284	0.260217359	1.837649686
29	H29	-4.401985571	0.709948458	-1.892885072
30	H30	4.748241339	-0.938425401	-1.920410535
31	H31	-0.926206519	1.255585540	2.397632156
32	H32	-3.002956039	1.590749964	3.718181201
33	H33	-5.796626914	-0.602235445	1.853147373
34	H34	-4.089844187	-1.111486477	1.884157359
35	H35	-4.889127220	-0.608917880	3.386138746
36	H36	-5.101769865	3.048307047	2.359190419
37	H37	-5.560092832	1.906595898	3.644533471
38	H38	-6.383010297	1.849807134	2.070047366
39	H39	-1.612613077	-0.213975779	-3.871669233
40	H40	4.592902881	-0.395892508	-4.105566011
41	H41	3.616340164	-1.369432548	-5.238660148
42	H42	4.300710560	-2.134312992	-3.779137022
43	H43	6.785025061	-0.703160792	1.011760172
44	H44	6.373128437	0.001877597	-0.573029327
45	H45	6.105322782	-1.743225302	-0.269583499
46	H46	2.236742729	-0.472422347	3.706695553
47	H47	3.048567383	-1.596480038	2.584255658
48	H48	1.281835990	-1.325959278	2.460374467

7 lowest-energy conformers used for Boltzmann-averaged ^{13}C NMR data.
Avg. Energy: -1338.868215 au

VIII.28b. B13-3 (Unreported natural product).

No	type	δcal	δexp	diff
C1	C	157.90 (-2.9)	157.30	0.60 (-2.9)
C2	C	107.50	105.20	2.30
C3	C	158.80	157.70	1.10
C4	CH	91.60	91.60	0.00
C4a	C	159.40	159.90*	1.40
C5	C	127.80	130.10	-2.30
C6	C	159.50	157.70	1.80
C7	CH	94.90	94.50	0.40

C8	C	161.40 (-2.9)	158.00 *	3.40 (-2.9)
C8a	C	104.90	103.30	1.60
C9	C	181.80	181.10	0.70
C9a	C	108.00	104.70	3.30
C10a	C	151.00	151.40	-0.40
C1'	CH	119.70 (-3.7)	115.50	4.20 (-3.7)
C2'	CH	125.10 (+2.8)	127.10	-2.00 (+2.8)
C3'	C	78.00	77.90	0.10
C4'[2]	CH3	27.40	28.20	-0.80
C1''	CH3	54.70	56.20	-1.50
C1'''	CH3	60.00	61.50	-1.50
C1''''	CH3	54.80	56.40	-1.60
RMSD		1.83		
Max abs		4.16		
RMSD+CFx		1.41		
Max abs+CFx		3.26		

mol2 coordinates for lowest energy conformer

1	O1	-1.350754280	1.810581287	-1.144150931
2	C2	-0.870759781	-2.143235574	2.061545743
3	C3	3.545364406	1.467849778	-2.217306457
4	C4	-1.997993229	-1.352172131	1.767401724
5	C5	2.554491755	2.335900269	-2.674242852
6	C6	0.359285125	-1.883085831	1.473585604
7	C7	3.245765568	0.448963029	-1.293928270
8	C8	-1.865943461	-0.299318716	0.860407357
9	C9	1.246743533	2.206658022	-2.208187597
10	O10	1.701427252	-0.642372433	0.065523717
11	C11	-0.441149391	1.065540959	-0.752087565
12	C12	0.460252195	-0.818132529	0.581965710
13	C13	1.940828587	0.345750731	-0.834280509
14	C14	-0.626988318	-0.001211731	0.238463981
15	C15	0.916496580	1.200770282	-1.269414882
16	C16	-3.283438072	-1.524284333	2.435951833
17	C17	-4.351858354	-0.863186887	1.989977909
18	C18	-4.253047269	-0.004165452	0.753374077
19	O19	-1.079784141	-3.141129955	2.946182948
20	C20	-4.596540492	-0.809266394	-0.502405409
21	C21	-5.114252884	1.247981657	0.864986226
22	O22	-2.905686573	0.502523132	0.600036280
23	C23	0.014022786	-3.961097470	3.301825717
24	C24	5.212541988	2.510404320	-3.537118931
25	O25	4.836159050	1.522118503	-2.601318144
26	C26	4.976549655	0.032754925	0.208709557
27	O27	4.201116381	-0.436542703	-0.883324893
28	H28	2.758818785	3.123229477	-3.387886507
29	H29	-3.331248591	-2.172064027	3.304232977
30	H30	-5.321076818	-0.945533651	2.473503828
31	H31	-4.487950124	-0.180814711	-1.390887548
32	H32	-3.929895797	-1.672178530	-0.595546285
33	H33	-5.626312136	-1.176148904	-0.446373964
34	H34	-4.848326086	1.812152750	1.763404766
35	H35	-6.172672650	0.974207665	0.920139397
36	H36	-4.957347958	1.886095850	-0.008638706
37	H37	0.813514373	-3.375486114	3.771655273
38	H38	0.412393017	-4.492813752	2.430456208
39	H39	-0.377270198	-4.681978553	4.020120602
40	H40	5.036774341	3.519565800	-3.146088755
41	H41	4.674662925	2.387544355	-4.484208231
42	H42	6.280065855	2.366980944	-3.705261969

43	H43	5.681024587	-0.765446918	0.453035073
44	H44	4.343050579	0.239918467	1.080716968
45	H45	5.533379565	0.937883491	-0.061825181
46	H46	1.246776234	-2.467772884	1.672520823
47	O47	0.325654183	3.055407980	-2.668623512
48	H48	-0.526862706	2.808656512	-2.226617201

6 lowest-energy conformers used for Boltzmann-averaged ^{13}C NMR data.
Avg. Energy: -1338.870779 au

VIII.29. Nigrolineaxanthone X (R29–D31)[90] and its revised structure morusignin H (D31-17).[91]

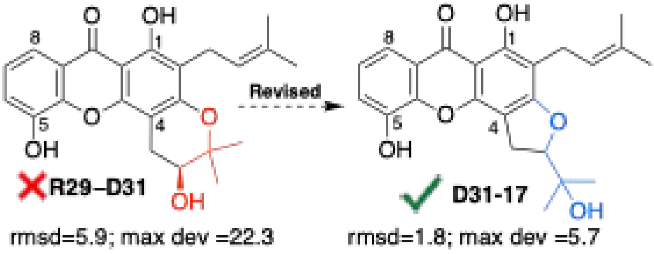

VIII. 29a. Nigrolineaxanthone X (R29–D31)[90]

No	type	δcal	δexp	diff
C1	C	160.80	160.50	0.30
C2	C	113.10	105.70	7.40
C3	C	158.60	165.40	-6.80
C4	C	96.90	103.20	-6.30
C4a	C	153.00	149.80	3.20
C5	C	145.10	145.50	-0.40
C6	CH	119.10	120.20	-1.10
C7	CH	124.20	123.40	0.80
C8	CH	118.40	115.20	3.20
C8a	C	122.80	121.30	1.50
C9	C	183.10	180.10	3.00
C9a	C	105.60	102.70	2.90
C10a	C	143.60	144.50	-0.90
C1'	CH2	22.90	21.00	1.90
C2'	CH	125.60	121.30	4.30
C3'	C	130.10	130.70	-0.60
C4'	CH3	18.90	16.50	2.40
C5'	CH3	25.50	25.00	0.50
C1''	CH2	26.50	26.50	0.00
C2''	CH	68.90	91.20	-22.30
C3''	C	79.80	70.20	9.60
C4''	CH3	23.10	24.50	-1.40
C5''	CH3	23.90	23.70	0.20
RMSD		5.91		
Max abs		22.31		

mol2 coordinates for lowest energy conformer

1	O1	0.927566989	3.118893334	-3.169229102
2	C2	0.520228579	-0.741245746	0.015617872
3	C3	1.316383378	6.302357694	0.721174389
4	C4	0.524339418	-0.722926430	-1.385171118
5	C5	1.327364405	6.517561333	-0.661487488
6	C6	0.660654758	0.411151052	0.802619919
7	C7	1.177453934	5.020933269	1.228529395
8	C8	0.654442234	0.508023480	-2.021441201
9	C9	1.201408131	5.459572376	-1.542997007
10	O10	0.912852987	2.724879210	0.906529691
11	C11	0.927034064	2.995294212	-1.936712151
12	C12	0.791063462	1.614836441	0.124545036
13	C13	1.049037856	3.953040468	0.329011152

14	C14	0.796927534	1.708546971	-1.274406821
15	C15	1.060494818	4.157487837	-1.046069482
16	C16	-1.195369281	-2.221428908	-2.352755070
17	C17	0.284310088	-1.986490867	-2.178402036
18	C18	-3.420549628	-3.278953681	-2.135791751
19	C19	-1.938390728	-3.220022848	-1.865250032
20	C20	-1.413849959	-4.364271927	-1.038404202
21	O21	1.165562014	4.808917230	2.565436869
22	O22	0.623430491	0.540311339	-3.357474188
23	C23	0.654157669	0.347034124	2.309089807
24	C24	0.824878432	-1.094918821	2.790723532
25	C25	-0.052878606	-2.045462706	1.955084836
26	O26	0.410152471	-1.968260284	0.585525309
27	O27	2.179867704	-1.491581661	2.744194543
28	C28	-1.535218925	-1.679779582	1.996339866
29	C29	0.161828744	-3.494969222	2.364090936
30	H30	1.416084799	7.125888731	1.420080127
31	H31	1.437073633	7.528870747	-1.038712048
32	H32	1.208144733	5.602123266	-2.617532562
33	H33	-1.695738330	-1.449359864	-2.938202480
34	H34	0.746006418	-1.870002010	-3.163799352
35	H35	0.771924645	-2.829615362	-1.685591438
36	H36	-3.991993960	-3.257322714	-1.198397982
37	H37	-3.754752113	-2.443731025	-2.757902385
38	H38	-3.689302108	-4.213483408	-2.644953647
39	H39	-0.366585928	-4.245366631	-0.753685873
40	H40	-1.521286205	-5.312494134	-1.580542026
41	H41	-2.001183612	-4.465250701	-0.116234523
42	H42	1.067862727	3.858775995	2.720015971
43	H43	0.729989323	1.484732525	-3.629668404
44	H44	-0.277937844	0.771197214	2.704719124
45	H45	1.474783987	0.939506511	2.725726377
46	H46	0.532155805	-1.171301380	3.841498013
47	H47	2.421336619	-1.582826223	1.811688476
48	H48	-1.904871291	-1.704771031	3.026226724
49	H49	-2.104674585	-2.399935933	1.402509995
50	H50	-1.723819577	-0.684900831	1.584379646
51	H51	-0.397363631	-4.160491165	1.701961075
52	H52	-0.192235132	-3.644469999	3.388727571
53	H53	1.221198594	-3.754300267	2.324768122

16 lowest-energy conformers used for Boltzmann-averaged ^{13}C NMR data.
Avg. Energy: -1342.334608 au

VIII.29b. Morusignin H (D31-17).[91]

No	type	δcal	δexp	diff
C1	C	163.1 (-2.9)	160.5	2.6 (-2.9)
C2	C	108.1	105.7	2.4
C3	C	165.9	165.4	0.5
C4	C	102.5	103.2	-0.7
C4a	C	150.5	149.8	0.7
C5	C	144.9	145.5	-0.6
C6	CH	119.1	120.2	-1.1
C7	CH	124.1	123.4	0.7
C8	CH	118.6 (-2.9)	115.2	3.4 (-2.9)
C8a	C	122.8	121.3	1.5
C9	C	182.8	180.1	2.7
C9a	C	104.9	102.7	2.2

C10a	C	143.4	144.5	-1.1
C1'	CH2	22.2	21.0	1.2
C2'	CH	127.0	121.3	5.7
C3'	C	132.9	130.7	2.2
C4'	CH3	25.6	25.0	0.6
C5'	CH3	17.9	16.5	1.4
C1''	CH2	27.9	26.5	1.4
C2''	CH	93.1	91.2	1.9
C3''	C	72.5	70.2	2.3
C4''	CH3	25.1	24.5	0.6
C5''	CH3	22.3	23.7	-1.4
RMSD		2.05		
Max abs		5.73		
RMSD+CFx		1.85		
Max abs+CFx		5.73		

mol2 coordinates for lowest energy conformer

1	O1	2.894530522	-1.952348723	2.835320446
2	C2	-0.084473557	-0.974706658	-1.034324929
3	C3	-1.028353511	-1.660902285	5.984664006
4	C4	1.289509849	-1.182864290	-1.032988875
5	C5	0.326156081	-1.934755160	6.204626268
6	C6	-0.896416221	-0.957089329	0.093436852
7	C7	-1.492768718	-1.414902912	4.702765978
8	C8	1.856012920	-1.453962949	0.216444676
9	C9	1.221722224	-1.964136596	5.151122564
10	O10	-1.116369801	-1.195934130	2.409904692
11	C11	1.681202448	-1.737291951	2.693607156
12	C12	-0.314203422	-1.218378876	1.309752220
13	C13	-0.579622450	-1.447806420	3.639549472
14	C14	1.068704806	-1.485818933	1.403981310
15	C15	0.768430906	-1.719029082	3.848551566
16	C16	1.880017055	0.344964382	-2.946399538
17	C17	2.114208658	-1.000897486	-2.285973447
18	C18	1.806613350	2.818335333	-3.143526771
19	C19	2.135343295	1.544041004	-2.406458675
20	C20	2.732832657	1.731239338	-1.034770499
21	O21	-2.802251320	-1.149188312	4.485450304
22	O22	3.176032617	-1.648228511	0.282527404
23	C23	-2.280193799	-0.541572448	-0.308117676
24	C24	-2.084280589	-0.203280217	-1.809133317
25	C25	-2.082318921	1.302967207	-2.157721166
26	O26	-0.771172745	-0.708861047	-2.164351866
27	C27	-3.400992885	1.963215183	-1.771225974
28	C28	-1.823711705	1.483645362	-3.657696904
29	H29	-1.738196047	-1.635312386	6.804550819
30	H30	0.670109376	-2.124849463	7.215947281
31	H31	2.275269044	-2.172498125	5.297581665
32	H32	1.445134703	0.314726642	-3.944310741
33	H33	3.169050154	-1.125087987	-2.024914929
34	H34	1.874840617	-1.792706573	-3.002859551
35	H35	2.705659809	3.429191855	-3.289261916
36	H36	1.364128952	2.617711860	-4.124719233
37	H37	1.095301107	3.427434487	-2.569613842
38	H38	3.201569139	0.823685387	-0.649284818
39	H39	1.961177485	2.047462850	-0.318620962
40	H40	3.486583187	2.526246550	-1.054712034
41	H41	-2.926263663	-1.004790619	3.536817610
42	H42	3.401310146	-1.806781308	1.234954078
43	H43	-2.613331776	0.329953160	0.260483911

44	H44	-3.018649194	-1.338350373	-0.170656569
45	H45	-2.800432778	-0.730007860	-2.445378107
46	H46	-4.246510612	1.460843262	-2.252758330
47	H47	-3.388715527	3.007286467	-2.094701505
48	H48	-3.554979582	1.953770498	-0.689253637
49	H49	-1.760937564	2.551595407	-3.887771777
50	H50	-2.629274192	1.044655565	-4.257344397
51	H51	-0.882787725	1.007607879	-3.946014464
52	O52	-1.066152696	1.945810184	-1.404836937
53	H53	-0.218090107	1.605951144	-1.732336895

6 lowest-energy conformers used for Boltzmann-averaged ^{13}C NMR data.
Avg. Energy: -1342.333007 au

VIII.30. Neriifolone C (R30–D31)[92] and its revised structure pruniflorone N (D31-18).[93,100]

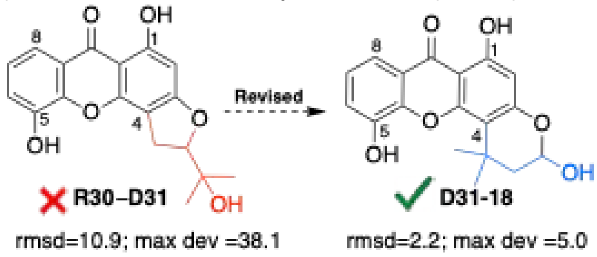

VIII.30a. Neriifolone C (R30–D31)[92]

No	type	δcal	δexp	diff
C1	C	167.1	162.1	5.0
C2	CH	93.8	100.0	-6.2
C3	C	167.2	161.6	5.6
C4	C	102.1	110.6	-8.5
C4a	C	152.4	156.7	-4.3
C5	C	144.9	147.4	-2.5
C6	CH	119.3	120.9	-1.6
C7	CH	124.3	124.8	-0.5
C8	CH	118.6	116.1	2.5
C8a	C	122.9	122.3	0.6
C9	C	182.5	182.1	0.4
C9a	C	105.3	105.1	0.2
C10a	C	143.4	146.3	-2.9
C1'	CH2	27.9	46.8	-18.9
C2'	CH	93.5	94.0	-0.5
C3'	C	71.1	109.2	-38.1
C4'	CH3	25.5	32.8	-7.3
C5'	CH3	23.7	32.8	-9.1
RMSD		10.94		
Max abs		38.09		

mol2 coordinates for lowest energy conformer

1	O1	-0.178736370	-0.718177446	-4.546158605
2	O2	2.202968378	-0.923532624	-3.521846984
3	O3	-4.246857974	0.388702964	-0.295843043
4	O4	2.182646943	1.929330591	2.681254406
5	C5	-0.320129999	-0.275412453	-0.947132128
6	C6	0.471834213	-0.268735253	0.177745545
7	C7	0.231653215	-0.504089524	-2.226536717
8	O8	-1.651212878	-0.052959523	-0.774025218
9	C9	1.840381384	-0.474926177	0.016193108
10	C10	0.189492738	0.015562635	1.623504302
11	C11	-0.616447881	-0.522107005	-3.403627793
12	C12	1.638373353	-0.708955801	-2.335653485
13	C13	-2.484403006	-0.064403876	-1.856833543

14	C14	2.455628016	-0.695658257	-1.204723857
15	O15	2.507527743	-0.427810105	1.188617593
16	C16	1.541436189	-0.354296208	2.273543202
17	C17	-2.049636060	-0.288705828	-3.158941395
18	C18	-3.835478335	0.169584323	-1.566637936
19	C19	2.077511687	0.656250692	3.299811608
20	C20	-2.984801083	-0.283635310	-4.201528969
21	C21	-4.747983078	0.170280833	-2.609653669
22	C22	-4.320429029	-0.056472280	-3.922194279
23	H23	1.482059372	-0.904035814	-4.199217086
24	H24	-3.471367462	0.360299301	0.281773178
25	H25	2.787545980	1.824115323	1.933701101
26	H26	3.521844507	-0.858438750	-1.295992520
27	H27	1.498610868	-1.354644684	2.721290353
28	H28	-2.629882660	-0.459374799	-5.210628974
29	H29	-5.792876600	0.350723112	-2.380490723
30	H30	-5.050154564	-0.051701601	-4.725094309
31	H31	-0.035891220	1.076721096	1.781170530
32	H32	-0.628911729	-0.579418923	2.038085182
33	C33	1.085481237	0.813767628	4.447411384
34	H34	1.480024358	1.531018349	5.172609032
35	H35	0.923460149	-0.141907931	4.956467329
36	H36	0.123609939	1.191351101	4.089473798
37	C37	3.444051109	0.204507832	3.825121347
38	H38	3.830960812	0.954437709	4.520652454
39	H39	4.157829513	0.086722614	3.004580600
40	H40	3.370268225	-0.753975930	4.349755179

4 lowest-energy conformers used for Boltzmann-averaged ^{13}C NMR data.
Avg. Energy: -1146.977687 au

VIII.30b. Pruniflorone N (D31-18)[93,100]

No	type	δcal	δexp	diff
C1	C	163.90 (-2.9)	161.60*	2.30 (-2.9)
C2	CH	100.60	100.00	0.60
C3	C	159.20	162.10*	-2.90
C4	C	106.40	110.60	-4.20
C4a	C	156.00	156.70	-0.70
C5	C	144.80	147.40	-2.60
C6	CH	119.20	120.90	-1.70
C7	CH	124.10	124.80	-0.70
C8	CH	118.50 (-2.9)	116.10	2.40 (-2.9)
C8a	C	122.60	122.30	0.30
C9	C	183.30	182.10	1.20
C9a	C	106.50	105.10	1.40
C10a	C	143.50	146.30	-2.80
C1'	C	30.40	32.80	-2.40
C2'	CH2	41.80	46.80	-5.00
C3'	CH	93.20	94.00	-0.80
RMSD		2.37		
Max abs		5.01		
RMSD+CFx		2.24		
Max abs+CFx		5.01		

mol2 coordinates for lowest energy conformer

1	C1	0.382596687	-1.102815220	2.371267582
2	C2	0.352846360	1.414578689	2.212044460
3	O3	-0.412409485	-0.619457723	-4.108461212
4	O4	-2.817585363	-0.353094889	-3.156375868
5	O5	3.661102201	-0.167286629	0.244932805
6	O6	-2.576213345	2.234010989	2.507580788

7	C7	-0.304921170	-0.129590279	-0.509846391
8	C8	-1.081332840	0.045141267	0.638444607
9	C9	-0.833321050	-0.274644165	-1.806193007
10	O10	1.045618822	-0.157140261	-0.324511327
11	C11	-2.464727353	0.141103086	0.407469529
12	C12	-0.484430139	0.131283896	2.046068407
13	C13	0.028865969	-0.479212290	-2.959269716
14	C14	-2.244268524	-0.216975887	-1.961828769
15	C15	1.895272147	-0.340328106	-1.371490380
16	C16	-3.042421837	0.003034268	-0.852936172
17	O17	-3.362661074	0.353608882	1.405247384
18	C18	-1.639127725	0.117710843	3.071950553
19	C19	1.471011567	-0.510533153	-2.682902933
20	C20	3.256811645	-0.342676158	-1.035668003
21	C21	-2.869509163	0.876406250	2.629159417
22	C22	2.423464927	-0.700038018	-3.692601503
23	C23	4.186654818	-0.532782438	-2.044354022
24	C24	3.767647104	-0.712895504	-3.368039501
25	H25	-0.172700755	-2.028802951	2.188940652
26	H26	1.295593989	-1.142349390	1.775106465
27	H27	0.670717022	-1.079859882	3.428682542
28	H28	0.771012505	1.459216567	3.224909286
29	H29	1.182970052	1.442339436	1.500419077
30	H30	-0.263057597	2.301339118	2.055357546
31	H31	-2.091586036	-0.488079206	-3.813415388
32	H32	2.884157689	0.015354684	0.791050502
33	H33	-3.371528247	2.677826037	2.185631056
34	H34	-4.117476817	0.076327747	-0.960629370
35	H35	-1.961628408	-0.915090092	3.246569393
36	H36	-1.290943329	0.518394746	4.029219132
37	H37	-3.701639718	0.713531833	3.323992377
38	H38	2.075237600	-0.831806147	-4.710643135
39	H39	5.239212490	-0.535965354	-1.781877514
40	H40	4.512696380	-0.859784594	-4.142999347

4 lowest-energy conformers used for Boltzmann-averaged ^{13}C NMR data.
Avg. Energy: -1146.979755 au

VIII.31. 1,3,7-Trihydroxy-4-(1,1-dimethyl-2-propenyl)-5,6-(2,2-dimethylchromeno)-xanthone (R31–D32)[94] and its revised structure cudraxanthone B (A23-3).[17]

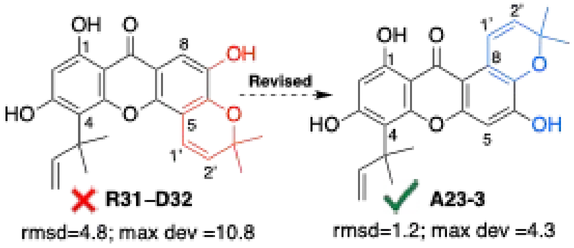

VIII.31a. 1,3,7-Trihydroxy-4-(1,1-dimethyl-2-propenyl)-5,6-(2,2-dimethylchromeno)-xanthone (R31–D32)[94]

No	type	δcal	δexp	diff
C1	C	164.6	162.2	2.4
C2	CH	100.7	100.6	0.1
C3	C	163.2	162.4	0.8

C4	C	106.0	109.4	-3.4
C4a	C	157.0	155.6	1.4
C5	C	111.1	120.0	-8.9
C6	C	145.3	151.4	-6.1
C7	C	142.7	153.0	-10.3
C8	CH	113.1	102.3	10.8
C8a	C	116.9	108.5	8.4
C9	C	182.4	183.2	-0.8
C10a	C	146.0	137.4	8.6
C16	C	105.6	105.0	0.6
C1'	CH	121.6	121.3	0.3
C2'	CH	127.7	132.8	-5.1
C3'	C	78.4	77.5	0.9
C4'[2]	CH3	27.0	27.7	-0.7
C1''	C	42.4	41.3	1.1
C2''	CH	149.1	149.7	-0.6
C3''	CH2	113.9	113.7	0.2
C4''[2]	CH3	30.4	28.4	2.0
RMSD		4.85		
Max abs		10.78		

mol2 coordinates for lowest energy conformer

1	C1	4.645825957	2.012281652	2.185420437
2	C2	-4.697461135	1.386607639	0.847587460
3	C3	-5.467835375	-0.685774318	2.073106187
4	C4	1.624762974	0.468685576	1.983884280
5	C5	3.749619123	-0.773926898	1.733435752
6	O6	0.271184637	-1.025710931	-4.317442189
7	O7	5.153471584	0.612531804	-0.679557617
8	O8	-4.464528444	-1.398837911	-2.538048357
9	O9	2.804655442	-0.601695882	-4.482698884
10	C10	3.586192258	1.757575545	1.414149838
11	C11	-1.878183493	-0.261904863	1.361355692
12	C12	-3.033303369	-0.081050350	2.004541465
13	C13	3.942900887	0.012230510	-2.536153799
14	C14	-2.120308019	-1.097777423	-2.822745510
15	O15	-4.255600913	-0.857719686	0.062371330
16	O16	0.444292514	-0.214267517	-0.327101412
17	C17	3.938901319	0.284810629	-1.172028276
18	C18	-3.244771700	-1.105977243	-2.029226670
19	C19	2.773017663	-0.346180819	-3.176818897
20	C20	-1.882221586	-0.502858445	-0.079198053
21	C21	-3.110788727	-0.795067260	-0.663281085
22	C22	2.775554142	0.205091303	-0.359798274
23	C23	0.328264952	-0.767022291	-3.104088602
24	C24	-0.868111051	-0.793650359	-2.267037029
25	C25	-0.750123078	-0.505475368	-0.911346081
26	C26	1.601470211	-0.141166688	-1.047503321
27	C27	1.565666412	-0.415477055	-2.432631409
28	C28	-4.348574507	-0.045759059	1.261386021
29	C29	2.931329528	0.403921086	1.166431804
30	H30	5.006065563	3.026750029	2.322986032
31	H31	5.187727220	1.229106824	2.706733431
32	H32	-4.785355925	2.025252717	1.732450143
33	H33	-3.913584471	1.798869270	0.204521419
34	H34	-5.648255999	1.405313326	0.304905655
35	H35	-5.208146806	-1.714522589	2.336945315
36	H36	-6.396983917	-0.693703925	1.495617722
37	H37	-5.637543378	-0.117941938	2.993104387
38	H38	0.937233170	1.233337970	1.615462627
39	H39	1.887228220	0.708934039	3.019405272

40	H40	1.115886985	-0.496126710	1.974045456
41	H41	3.878298610	-0.676237935	2.817248278
42	H42	3.210542679	-1.706104281	1.541742085
43	H43	4.737034673	-0.860850067	1.271847122
44	H44	5.064750661	1.071178756	0.171913837
45	H45	-5.100495626	-1.328666442	-1.810727330
46	H46	1.879734822	-0.826711372	-4.755310984
47	H47	3.085155274	2.588234198	0.914701453
48	H48	-0.934739319	-0.254752174	1.890894865
49	H49	-3.063798336	0.084448002	3.077612148
50	H50	4.869432013	0.093227696	-3.090564836
51	H51	-2.185484319	-1.315470768	-3.882498898

5 lowest-energy conformers used for Boltzmann-averaged ^{13}C NMR data.
Avg. Energy: -1341.092962 au

VIII.31b. Cudraxanthone B (A23-3).[17]

No	type	δcal	δexp	diff
C1	C	164.60 (-2.9)	162.20	2.40 (-2.9)
C2	CH	101.00	100.60	0.40
C3	C	163.80	162.40	1.40
C4	C	105.10	109.40	-4.30
C4a	C	155.80	155.60	0.20
C5	CH	102.00	102.30	-0.30
C6	C	152.10	151.40	0.70
C7	C	136.00	137.40	-1.40
C8	C	124.00 (-2.9)	120.00	4.00 (-2.9)
C8a	C	109.50	108.50	1.00
C9	C	184.80	183.20	1.60
C9a	C	105.50	105.00	0.50
C10a	C	153.10	153.00	0.10
C1'	CH	125.10 (-3.7)	121.30	3.80 (-3.7)
C2'	CH	131.30 (+2.8)	132.80	-1.50 (+2.8)
C3'	C	76.90	77.50	-0.60
C4'[2]	CH3	26.50	27.70	-1.20
C1''	C	42.00	41.30	0.70
C2''	CH	149.50	149.70	-0.20
C3''	CH2	114.30	113.70	0.60
C4''[2]	CH3	29.10	28.40	0.70
RMSD		1.75		
Max abs		4.30		
RMSD+CFx		1.23		
Max abs+CFx		4.30		

mol2 coordinates for lowest energy conformer in **SI: IV.9**
4 lowest-energy conformers used for Boltzmann-averaged ^{13}C NMR data.
Avg. Energy: -1341.093935 au

Bibliography

(1) Yimdjo, M. C.; Azebaze, A. G.; Nkengfack, A. E.; Meyer, A. M.; Bodo, B.; Fomum, Z. T. Antimicrobial and cytotoxic agents from *Calophyllum inophyllum*. *Phytochemistry* **2004**, *65* (20), 2789-2795. DOI: 10.1016/j.phytochem.2004.08.024.

(2) Shen, Y. C.; Wang, L. T.; Khalil, A. T.; Chiang, L. C.; Cheng, P. W. Bioactive pyranoxanthones from the roots of *Calophyllum blancoi*. *Chem Pharm Bull* **2005**, *53* (2), 244-247. DOI: 10.1248/cpb.53.244.

(3) Iinuma, M.; Tosa, H.; Tanaka, T.; Yonemori, S. Two New Xanthones in the Underground Part of *Calophyllum inophyllum*. *Heterocycles* **1994**, *37*, 833-838. DOI: 10.3987/com-93-s70.

(4) Mah, S. H.; Ee, G. C. L.; Rahmani, M.; Taufiq-Yap, Y. H.; Sukari, M. A.; Teh, S. S. A New Pyranoxanthone from *Calophyllum soulattri*. *Molecules* **2011**, *16* (5), 3999-4004. DOI: 10.3390/molecules16053999.

(5) Goh, S. H.; Jantan, I.; Gray, A. I.; Waterman, P. G. Prenylated xanthones from *Garcinia opaca*. *Phytochemistry* **1992**, *31* (4), 1383-1386. DOI: 10.1016/0031-9422(92)80296-Q.

(6) Teh, S. S.; Ee, G. C. L.; Rahmani, M.; Taufiq-Yap, Y. H.; Go, R.; Mah, S. H. Pyranoxanthones from *Mesua ferrea*. *Molecules* **2011**, *16* (7), 5647-5654. DOI: 10.3390/molecules16075647.

(7) Ee, G. C. L.; Kua, A. S. M.; Cheow, Y. L.; Lim, C. K.; Jong, V.; Rahmani, M. A New Pyranoxanthone Inophyllin B from *Calophyllum inophyllum*. *Nat Prod Sci* **2004**, *10*, 220-222. DOI: https://koreascience.kr/article/JAKO200403041150920.page.

(8) Ee, G. C. L.; Lim, C. K.; Rahmat, A. Structure-activity relationship of xanthones from *Mesua daphnifolia* and *Garcinia nitida* towards human estrogen receptor negative breast cancer cell line. *Nat Prod Sci* **2005**, *11*, 220-224. DOI: https://koreascience.kr/article/JAKO200503041157684.pdf.

(9) Iinuma, M.; Tosa, H.; Tanaka, T.; Yonemori, S. Two Xanthones from root bark of *Calophyllum-inophyllum*. *Phytochemistry* **1994**, *35* (2), 527-532. DOI: 10.1016/S0031-9422(00)94795-2.

(10) Ngoupayo, J.; Tabopda, T. K.; Ali, M. S. Antimicrobial and immunomodulatory properties of prenylated xanthones from twigs of *Garcinia staudtii*. *Bioorg Med Chem* **2009**, *17* (15), 5688-5695. DOI: 10.1016/j.bmc.2009.06.009.

(11) Kapingu, M. C.; Magadula, J. J. Prenylated Xanthones and a Benzophenone from *Baphia Kirkii*. *Nat Prod Commun* **2008**, *3* (9), 1501-1504. DOI: 10.1177/1934578X0800300921.

(12) Azevedo, C. M. G.; Afonso, C. M. M.; Soares, J. X.; Reis, S.; Sousa, D.; Lima, R. T.; Vasconcelos, M. H.; Pedro, M.; Barbosa, J.; Gales, L.; Pinto, M. M. M. Pyranoxanthones: Synthesis, growth inhibitory activity on human tumor cell lines and determination of their lipophilicity in two membrane models. *Eur J Med Chem* **2013**, *69*, 798-816. DOI: 10.1016/j.ejmech.2013.09.012.

(13) Cao, T.-T.; Huang, R.-Y.; Li, X.; Yang, T.-Y.; Xie, H.-D.; Shen, Y.-H.; Li, F.; Li, X. Xanthones from *Calophyllum polyanthum* Wallich ex Choisy with CYP1 Enzymes Inhibitory Activity. *Chem Biodivers* **2022**, *19* (6), e202200268. DOI: 10.1002/cbdv.202200268.

(14) Suksamrarn, S.; Komutiban, O.; Ratananukul, P.; Chimnoi, N.; Lartpornmatulee, N.; Suksamrarn, A. Cytotoxic prenylated xanthones from the young fruit of *Garcinia mangostana*. *Chem Pharm Bull* **2006**, *54* (3), 301-305. DOI: 10.1248/cpb.54.301.

(15) Hay, A. E.; Guilet, D.; Morel, C.; Larcher, G.; Macherel, D.; Le Ray, A. M.; Litaudon, M.; Richomme, P. Antifungal Chromans Inhibiting the Mitochondrial Respiratory Chain of Pea Seeds and New Xanthones from *Calophyllum caledonicum*. *Planta Med* **2003**, *69* (12), 1130-1135. DOI: 10.1055/s-2003-818004.

(16) Chang, C. H.; Lin, C. C.; Hattori, M.; Namba, T. Four Prenylated Xanthones from *Cudrania cochinchinensis*. *Phytochemistry* **1989**, *28* (2), 595-598. DOI: 10.1016/0031-9422(89)80058-5.

(17) Hano, Y.; Matsumoto, Y.; Sun, J. Y.; Nomura, T. Structures of Four New Isoprenylated Xanthones, Cudraxanthones H,I,J and K1,2. *Planta Med* **1990**, *56* (5), 478-481. DOI: 10.1055/s-2006-961016.

(18) Dharmaratne, H. R. W.; Wanigasekera, W. M. A. P.; Amarasekara, A. S. Batukinaxanthone, a New Trioxygenated Diprenylated Chromenxanthone from *Calophyllum Thwaitesii*. *Nat Prod Lett* **1996**, *8* (4), 241-243. DOI: 10.1080/10575639608044901.

(19) Tanaka, N.; Takaishi, Y.; Shikishima, Y.; Nakanishi, Y.; Bastow, K.; Lee, K. H.; Honda, G.; Ito, M.; Takeda, Y.; Kodzhimatov, O. K.; Ashurmetov, O. Prenylated benzophenones and xanthones from *Hypericum scabrum*. *J Nat Prod* **2004**, *67* (11), 1870-1875. DOI: 10.1021/np040024+.

(20) Ishiguro, K.; Fukumoto, H.; Nakajima, M.; Isoi, K. Xanthones in Cell-Suspension Cultures of *Hypericum paturum*. *Phytochemistry* **1993**, *33* (4), 839-840. DOI: 10.1016/0031-9422(93)85286-Z.

(21) Manning, K.; Petrunak, E.; Lebo, M.; Gonzalez-Sarrias, A.; Seeram, N. P.; Henry, G. E. Acylphloroglucinol and xanthones from *Hypericum ellipticum*. *Phytochemistry* **2011**, *72* (7), 662-667. DOI: 10.1016/j.phytochem.2011.01.032.

(22) Jo, Y. H.; Kim, S. B.; Ahn, J. H.; Turk, A.; Kwon, E.-B.; Kim, M.-O.; Hwang, B. Y.; Lee, M. K. Xanthones from the stems of *Cudrania tricuspidata* and their inhibitory effects on pancreatic lipase and fat accumulation. *Bioorg Chem* **2019**, *92*, 103234. DOI: 10.1016/j.bioorg.2019.103234.

(23) Sen, A. K.; Sarkar, K. K.; Mazumder, P. C.; Banerji, N.; Uusvuori, R.; Hase, T. A. The structures of garcinones A, B and C - 3 new xanthones from *Garcinia mangostana*. *Phytochemistry* **1982**, *21* (7), 1747-1750. DOI: 10.1016/S0031-9422(82)85052-8.

(24) Azebaze, A. G.; Meyer, M.; Bodo, B.; Nkengfack, A. E. Allanxanthone B, a polyisoprenylated xanthone from the stem bark of *Allanblackia monticola* Staner L.C. *Phytochemistry* **2004**, *65* (18), 2561-2564. DOI: 10.1016/j.phytochem.2004.07.027.

(25) Marques, V. L.; De Oliveira, F. M.; Conserva, L. M.; Brito, R. G.; Guilhon, G. M. Dichromenoxanthones from T*ovomita brasiliensis*. *Phytochemistry* **2000**, *55* (7), 815-818. DOI: 10.1016/s0031-9422(00)00296-x.

(26) Cheng, H.-C.; Wang, L.-T.; Khalil, A. T.; Chang, Y.-T.; Lin, Y.-C.; Shen, Y.-C. Pyranoxanthones from *Calophyllum Inophyllum*. *J Chin Chem Soc* **2004**, *51* (2), 431-435. DOI: 10.1002/jccs.200400066.
(27) Rukachaisirikul, V.; Tadpetch, K.; Watthanaphanit, A.; Saengsanae, N.; Phongpaichit, S. Benzopyran, biphenyl, and tetraoxygenated xanthone derivatives from the twigs of *Garcinia nigrolineata*. *J Nat Prod* **2005**, *68* (8), 1218-1221. DOI: 10.1021/np058050a.
(28) Suksamrarn, S.; Suwannapoch, N.; Ratananukul, P.; Aroonlerk, N.; Suksamrarn, A. Xanthones from the green fruit hulls of *Garcinia mangostana*. *J Nat Prod* **2002**, *65* (5), 761-763. DOI: 10.1021/np010566g.
(29) Deachathai, S.; Mahabusarakam, W.; Phongpaichit, S.; Taylor, W. C.; Zhang, Y. J.; Yang, C. R. Phenolic compounds from the flowers of *Garcinia dulcis*. *Phytochemistry* **2006**, *67* (5), 464-469. DOI: 10.1016/j.phytochem.2005.10.016.
(30) Bennett, G. J.; Harrison, L. J.; Sia, G.-L.; Sim, K.-Y. Triterpenoids, tocotrienols and xanthones from the bark of *Cratoxylum Cochinchinense*. *Phytochemistry* **1993**, *32* (5), 1245-1251. DOI: 10.1016/S0031-9422(00)95100-8.
(31) Kawahara, N.; Nozawa, K.; Nakajima, S.; Kawai, K.-i. Studies on fungal products. Part 15. Isolation and structure determination of arugosin E from *Aspergillus silvaticus* and cycloisoemericellin from *Emericella striata*. *J Chem Soc, Perkin Trans 1* **1988**, (4), 907-911. DOI: 10.1039/P19880000907.
(32) Xu, Y.-Z.; Tian, J.-W.; Sha, F.; Li, Q.; Wu, X.-Y. Concise Synthesis of Chromene/Chromane-Type Aryne Precursors and Their Applications. *J Org Chem* **2021**, *86* (9), 6765-6779. DOI: 10.1021/acs.joc.1c00493.
(33) Pailee, P.; Kuhakarn, C.; Sangsuwan, C.; Hongthong, S.; Piyachaturawat, P.; Suksen, K.; Jariyawat, S.; Akkarawongsapat, R.; Limthongkul, J.; Napaswad, C.; Kongsaeree, P.; Prabpai, S.; Jaipetch, T.; Pohmakotr, M.; Tuchinda, P.; Reutrakul, V. Anti-HIV and cytotoxic biphenyls, benzophenones and xanthones from stems, leaves and twigs of *Garcinia speciosa*. *Phytochemistry* **2018**, *147*, 68-79. DOI: 10.1016/j.phytochem.2017.12.013.
(34) Mondal, M.; Puranik, V. G.; Argade, N. P. Facile Synthesis of 1,3,7-Trihydroxyxanthone and Its Regioselective Coupling Reactions with Prenal: Simple and Efficient Access to Osajaxanthone and Nigrolineaxanthone F. *J Org Chem* **2006**, *71* (13), 4992-4995. DOI: 10.1021/jo0606655.
(35) Rocha, L.; Marston, A.; Kaplan, M. A.; Stoeckli-Evans, H.; Thull, U.; Testa, B.; Hostettmann, K. An antifungal gamma-pyrone and xanthones with monoamine oxidase inhibitory activity from *Hypericum brasiliense*. *Phytochemistry* **1994**, *36* (6), 1381-1385. DOI: 10.1016/s0031-9422(00)89727-7 From NLM.
(36) Westerman, P. W.; Gunasekera, S. P.; Uvais, M.; Sultanbawa, S.; Kazlauskas, R. Carbon-13 n.m.r. study of naturally occurring xanthones. *Org Mag Reson* **1977**, *9* (11), 631-636. DOI: 10.1002/mrc.1270091106.
(37) Rukachaisirikul, V.; Kamkaew, M.; Sukavisit, D.; Phongpaichit, S.; Sawangchote, P.; Taylor, W. C. Antibacterial xanthones from the leaves of *Garcinia nigrolineata*. *J Nat Prod* **2003**, *66* (12), 1531-1535. DOI: 10.1021/np0303254.
(38) Zhou, X.; Huang, R.; Hao, J.; Huang, H.; Fu, M.; Xu, Z.; Zhou, Y.; Li, X.-E.; Qiu, S. X.; Wang, B. Two New Prenylated Xanthones from the Pericarp of *Garcinia mangostana* (Mangosteen). *Hel Chim Acta* **2011**, *94* (11), 2092-2098. DOI: 10.1002/hlca.201100157.
(39) Tanjung, M.; Rachmadiarti, F.; Prameswari, A.; Ultha Wustha Agyani, V.; Dewi Saputri, R.; Srie Tjahjandarie, T.; Maolana Syah, Y. Airlanggins A-B, two new isoprenylated benzofuran-3-ones from the stem bark of *Calophyllum soulattri*. *Nat Prod Res* **2018**, *32* (13), 1493-1498. DOI: 10.1080/14786419.2017.1380027.
(40) Monache, F. D.; Botta, B.; Nicoletti, M.; de Barros Coêlho, J. S.; de Andrade Lyra, F. D. Three new xanthones and macluraxanthone from *Rheedia benthamiana* Pl. Triana (guttiferae). *J Chem Soc, Perkin Trans 1* **1981**, (0), 484-488. DOI: 10.1039/P19810000484.
(41) Kosela, S.; Hu, L. H.; Yip, S. C.; Rachmatia, T.; Sukri, T.; Daulay, T. S.; Tan, G. K.; Vittal, J. J.; Sim, K. Y. Dulxanthone E: a pyranoxanthone from the leaves of *Garcinia dulcis*. *Phytochemistry* **1999**, *52* (7), 1375-1377. DOI: 10.1016/S0031-9422(99)00387-8.
(42) Rukachaisirikul, V.; Ritthiwigrom, T.; Pinsa, A.; Sawangchote, P.; Taylor, W. C. Xanthones from the stem bark of *Garcinia nigrolineata*. *Phytochemistry* **2003**, *64* (6), 1149-1156. DOI: 10.1016/s0031-9422(03)00502-8.
(43) Shen, J.; Yang, J. S. Two new xanthones from the stems of *Garcinia cowa*. *Chem Pharm Bull* **2006**, *54* (1), 126-128. DOI: 10.1248/cpb.54.126.
(44) Zhong, F.; Chen, Y.; Wang, P.; Feng, H.; Yang, G. Xanthones from the Bark of *Garcinia xanthochymus* and Their 1,1-Diphenyl-2-picrylhydrazyl Radical-Scavenging Activity. *Chin J Chem* **2009**, *27* (1), 74-80. DOI: 10.1002/cjoc.200990029.
(45) Iinuma, M.; Tosa, H.; Tanaka, T.; Asai, F.; Shimano, R. 2 Xanthones with a 1,1-dimethylallyl group in root bark of *Garcinia subelliptica*. *Phytochemistry* **1995**, *39* (4), 945-947. DOI: 10.1016/0031-9422(95)00041-5.
(46) Vo, H. T.; Nguyen, N.-T. T.; Maas, G.; Werz, U. R.; Pham, H. D.; Nguyen, L.-H. D. Xanthones from the bark of *Garcinia pedunculata*. *Phytochem Lett* **2012**, *5* (4), 766-769. DOI: 10.1016/j.phytol.2012.08.009.
(47) Nguyen, L. H.; Vo, H. T.; Pham, H. D.; Connolly, J. D.; Harrison, L. J. Xanthones from the bark of *Garcinia merguensis*. *Phytochemistry* **2003**, *63* (4), 467-470. DOI: 10.1016/s0031-9422(02)00433-8.
(48) Teh, S. S.; Ee, G. C. L.; Rahmani, M.; Sim, W. C.; Mah, S. H.; Teo, S. H. Two New Pyranoxanthones from *Mesua beccariana* (Guttiferae). *Molecules* **2010**, *15* (10), 6733-6742. DOI: 10.3390/molecules15106733.
(49) Azevedo, C. M. G.; Afonso, C. M. M.; Sousa, D.; Lima, R. T.; Helena Vasconcelos, M.; Pedro, M.; Barbosa, J.; Corrêa, A. G.; Reis, S.; Pinto, M. M. M. Multidimensional optimization of promising antitumor xanthone derivatives. *Bioorg Med Chem* **2013**, *21* (11), 2941-2959. DOI: 10.1016/j.bmc.2013.03.079.

(50) Morel, C.; Seraphin, D.; Oger, J. M.; Litaudon, M.; Sevenet, T.; Richomme, P.; Bruneton, J. New xanthones from *Calophyllum caledonicum*. *J Nat Prod* **2000**, *63* (11), 1471-1474. DOI: 10.1021/np000215m.
(51) França, F.; Silva, P. M. A.; Soares, J. X.; Henriques, A. C.; Loureiro, D. R. P.; Azevedo, C. M. G.; Afonso, C. M. M.; Bousbaa, H. A Pyranoxanthone as a Potent Antimitotic and Sensitizer of Cancer Cells to Low Doses of Paclitaxel. *Molecules* **2020**, *25* (24), 5845. DOI: 10.3390/molecules25245845.
(52) Chen, Y.; Fan, H.; Yang, G.-z.; Jiang, Y.; Zhong, F.-f.; He, H.-w. Prenylated Xanthones from the Bark of Garcinia xanthochymus and Their 1,1-Diphenyl-2-picrylhydrazyl (DPPH) Radical Scavenging Activities. *Molecules* **2010**, *15* (10), 7438-7449. DOI: 10.3390/molecules15107438.
(53) Minami, H.; Takahashi, E.; Fukuyama, Y.; Kodama, M.; Yoshizawa, T.; Nakagawa, K. Novel xanthones with superoxide scavenging activity from *Garcinia subelliptica*. *Chem Pharm Bull* **1995**, *43* (2), 347-349. DOI: 10.1248/cpb.43.347.
(54) Fukuyama, Y.; Kamiyama, A.; Mima, Y.; Kodama, M. Prenylated Xanthones From *Garcinia-subelliptica*. *Phytochemistry* **1991**, *30* (10), 3433-3436. DOI: 10.1016/0031-9422(91)83223-8.
(55) Mahabusarakam, W.; Mecawun, P.; Phongpaichit, S. Xanthones from the green branch of *Garcinia dulcis*. *Nat Prod Res* **2016**, *30* (20), 2323-2328. DOI: 10.1080/14786419.2016.1169417.
(56) Gobbi, S.; Rampa, A.; Bisi, A.; Belluti, F.; Valenti, P.; Caputo, A.; Zampiron, A.; Carrara, M. Synthesis and Antitumor Activity of New Derivatives of Xanthen-9-one-4-acetic Acid. *J Med Chem* **2002**, *45* (22), 4931-4939. DOI: 10.1021/jm020929p.
(57) Nale, S. D.; Maiti, D.; Lee, Y. R. Construction of Highly Functionalized Xanthones via Rh-Catalyzed Cascade C–H Activation/O-Annulation. *Org Lett* **2021**, *23* (7), 2465-2470. DOI: 10.1021/acs.orglett.1c00391.
(58) Castanheiro, R. A. P.; Silva, A. M. S.; Campos, N. A. N.; Nascimento, M. S. J.; Pinto, M. M. M. Antitumor Activity of Some Prenylated Xanthones. *Pharmaceuticals* **2009**, *2* (2), 33-43. DOI: 10.3390/ph2020033.
(59) Ishiguro, K.; Nagata, S.; Fukumoto, H.; Yamaki, M.; Isoi, K.; Oyama, Y. An isopentenylated flavonol from *Hypericum japonicum*. *Phytochemistry* **1993**, *32* (6), 1583-1585. DOI: 10.1016/0031-9422(93)85184-S.
(60) Zou, Y. S.; Hou, A. J.; Zhu, G. F.; Chen, Y. F.; Sun, H. D.; Zhao, Q. S. Cytotoxic isoprenylated xanthones from *Cudrania tricuspidata*. *Bioorg Med Chem* **2004**, *12* (8), 1947-1953. DOI: 10.1016/j.bmc.2004.01.030.
(61) Huang, S. X.; Feng, C.; Zhou, Y.; Xu, G.; Han, Q. B.; Qiao, C. F.; Chang, D. C.; Luo, K. Q.; Xu, H. X. Bioassay-guided isolation of xanthones and polycyclic prenylated acylphloroglucinols from *Garcinia oblongifolia*. *J Nat Prod* **2009**, *72* (1), 130-135. DOI: 10.1021/np800496c.
(62) An, R.-B.; Sohn, D.-H.; Kim, Y.-C. Hepatoprotective Compounds of the Roots of *Cudrania tricuspidata* on Tacrine-Induced Cytotoxicity in Hep G2 Cells. *Biol Pharm Bull* **2006**, *29* (4), 838-840. DOI: 10.1248/bpb.29.838.
(63) Jo, Y. H.; Shin, B.; Liu, Q.; Lee, K. Y.; Oh, D. C.; Hwang, B. Y.; Lee, M. K. Antiproliferative prenylated xanthones and benzophenones from the roots of *Cudrania tricuspidata* in HSC-T6 cells. *J Nat Prod* **2014**, *77* (11), 2361-2366. DOI: 10.1021/np5002797.
(64) Kwon, J.; Hiep, N. T.; Kim, D. W.; Hwang, B. Y.; Lee, H. J.; Mar, W.; Lee, D. Neuroprotective Xanthones from the Root Bark of *Cudrania tricuspidata*. *J Nat Prod* **2014**, *77* (8), 1893-1901. DOI: 10.1021/np500364x.
(65) Morel, C.; Seraphin, D.; Teyrouz, A.; Larcher, G.; Bouchara, J. P.; Litaudon, M.; Richomme, P.; Bruneton, J. New and antifungal xanthones from *Calophyllum caledonicum*. *Planta Med* **2002**, *68* (1), 41-44. DOI: 10.1055/s-2002-19867.
(66) *SIMFIT Statistical Package 2024* 2025. https://simfit.org.uk/ (accessed.
(67) Ito, C.; Itoigawa, M.; Mishina, Y.; Filho, V. C.; Mukainaka, T.; Tokuda, H.; Nishino, H.; Furukawa, H. Chemical constituents of *Calophyllum brasiliensis*: structure elucidation of seven new xanthones and their cancer chemopreventive activity. *J Nat Prod* **2002**, *65* (3), 267-272. DOI: 10.1021/np010398s.
(68) Deachathai, S.; Mahabusarakam, W.; Phongpaichit, S.; Taylor, W. C. Phenolic compounds from the fruit of *Garcinia dulcis*. *Phytochemistry* **2005**, *66* (19), 2368-2375. DOI: 10.1016/j.phytochem.2005.06.025.
(69) Mah, S. H.; Gwendoline, C. L. E.; Rahmani, M.; Taufiq-Yap, Y. H.; Go, R.; Teh, S. S. A New Pyranoxanthone from the Stem Bark of *Calophyllum inophyllum*. *Lett Org Chem* **2011**, *8* (6), 447-449. DOI: 10.2174/157017811796064430.
(70) Tjahjandarie, T. S.; Saputri, R. D.; Tanjung, M. 5,9,11-Trihydroxy-2,2-dimethyl-3-(2-methylbut-3-en-2-yl)pyrano[2,3-a]xanthen-12(2H)-one from the Stem Bark of *Calophyllum tetrapterum* Miq. *Molbank* **2017**, *2017* (1), M936. DOI: 10.3390/M936.
(71) Tanjung, M.; Tjahjandarie, T. S.; Saputri, R. D.; Aldin, M. F.; Purnobasuki, H. Two new pyranoxanthones from the stem bark of *Calophyllum pseudomolle* P.F. Stevens. *Nat Prod Res* **2022**, *36* (3), 822-827. DOI: 10.1080/14786419.2020.1808638.
(72) Tanjung, M.; Saputri, R. D.; Tjahjandarie, T. S. 5,9,11-Trihydroxy-2,2-dimethyl-10-(3′-methyl-2′-butenyl)-3-(2″-methyl-3″-butenyl)pyrano[2,3-a]xanthen-12(2H)-one from the Stem Bark of *Calophyllum pseudomole*. *Molbank* **2016**, *2016* (3), M906. DOI: 10.3390/M906.
(73) Tanjung, M.; Tjahjandarie, T. S.; Saputri, R. D.; Kurnia, B. D.; Rachman, M. F.; Syah, Y. M. Calotetrapterins A-C, three new pyranoxanthones and their cytotoxicity from the stem bark of *Calophyllum tetrapterum* Miq. *Nat Prod Res* **2021**, *35* (3), 407-412. DOI: 10.1080/14786419.2019.1634714.
(74) Tanjung, M.; Saputri, R. D.; Tjahjandarie, T. S. 5,9,11-Trihydroxy-10-(2″-hydroxy-3″-methylbut-3″-en-1-yl)-2,2-dimethyl-3-(2′-methylbut-3′-en-2′-yl)-2H,12H-pyrano[2,3-a]xanthen-12-one from *Calophyllum pseudomole*. *Molbank* **2017**, *2017* (4), M961. DOI: 10.3390/M961.

(75) Chi, X.-Q.; Zi, C.-T.; Li, H.-M.; Yang, L.; Lv, Y.-F.; Li, J.-Y.; Hou, B.; Ren, F.-C.; Hu, J.-M.; Zhou, J. Design, synthesis and structure–activity relationships of mangostin analogs as cytotoxic agents. *RSC Advances* **2018**, *8* (72), 41377-41388. DOI: 10.1039/C8RA08409B.
(76) Morelli, C. F.; Biagiotti, M.; Pappalardo, V. M.; Rabuffetti, M.; Speranza, G. Chemistry of α-mangostin. Studies on the semisynthesis of minor xanthones from *Garcinia mangostana*. *Nat Prod Res* **2015**, *29* (8), 750-755. DOI: 10.1080/14786419.2014.986729.
(77) Boonnak, N.; Chantrapromma, S.; Sathirakul, K.; Kaewpiboon, C. Modified tetra-oxygenated xanthones analogues as anti-MRSA and *P. aeruginosa* agent and their synergism with vancomycin. *Bioorg Med Chem Lett* **2020**, *30* (20), 127494. DOI: 10.1016/j.bmcl.2020.127494.
(78) Sana, T.; Khan, M.; Jabeen, A.; Shams, S.; Hadda, T. B.; Begum, S.; Siddiqui, B. S. Urease and Carbonic Anhydrase Inhibitory Effect of Xanthones from *Aspergillus nidulans*, an Endophytic Fungus of Nyctanthes arbor-tristis. *Planta Med* **2023**, *89* (4), 377-384. DOI: 10.1055/a-1908-0935.
(79) Zhang, D.; Yi, W.; Ge, H.; Zhang, Z.; Wu, B. A new antimicrobial indoloditerpene from a marine-sourced fungus *Aspergillus versicolor* ZZ761. *Nat Prod Res* **2021**, *35* (18), 3114-3119. DOI: 10.1080/14786419.2019.1684281.
(80) Huang, Y. L.; Chen, C. C.; Chen, Y. J.; Huang, R. L.; Shieh, B. J. Three xanthones and a benzophenone from *Garcinia mangostana*. *J Nat Prod* **2001**, *64* (7), 903-906. DOI: 10.1021/np000583q.
(81) bin Ismail, A. A. F.; Ee, G. C. L.; bin Daud, S.; Teh, S. S.; Hashim, N. M.; Awang, K. Venuloxanthone, a new pyranoxanthone from the stem bark of *Calophyllum venulosum*. *J Asian Nat Prod Res* **2015**, *17* (11), 1104-1108. DOI: 10.1080/10286020.2015.1047353.
(82) Hartati, S.; Kadono, L. B. S.; Kosela, S.; Harrison, L. J. A New Pyrano Xanthone from the Stem Barks of *Garcinia tetrandra* Pierre *J Bio Sci* **2008**, (6), 137-142. DOI: 10.3923/jbs.2008.137.142.
(83) Han, Y.; Li, X.; Yuan, C.; Gu, R.; Kennelly, E. J.; Long, C. Chemical Constituents From the Bark of *Garcinia oblongifolia*. *Nat Prod Commun* **2020**, *15* (8), 1934578X20944660. DOI: 10.1177/1934578X20944660.
(84) Yong Rok, L.; Gala Sri, H. Facile Synthesis of Pyranoxanthones, Dihydropyranoxanthones, and Their Analogues. *Bull Korean Chem Soc* **2011**, *32* (8), 2949-2954. DOI: 10.5012/bkcs.2011.32.8.2949.
(85) Ghirtis, K.; Pouli, N.; Marakos, P.; Skaltsounis, A.-L.; Leonce, S.; Daniel, H. C.; Atassi, G. Synthesis and Conformational Analysis of Some New Pyrano[2,3-c]xanthen-7-one and Pyrano[3,2-b]xanthen-6-one Derivatives with Cytotoxic Activity. *Heterocycles* **2000**, *53* (1), 93-106. DOI: 10.3987/COM-99-8727.
(86) Xian, L.; Tingting, C.; Fei, L.; Weimin, Y.; Chen, C.; Ruoyue, H.; Xu, L.; Jia, L.; Huiding, X.; Yong, L.; et al. Preparation of isopentenyl substituted dibenzopyrone compounds and its application *Spacenet* **2022**, (CN114751912 A). DOI: https://worldwide.espacenet.com/patent/search/family/082325386/publication/CN114751912A?q=CN114751912A.
(87) Ee, G. C.; Kua, A. S.; Lim, C. K.; Jong, V.; Lee, H. L. Inophyllin A, a new pyranoxanthone from *Calophyllum inophyllum* (Guttiferae). *Nat Prod Res* **2006**, *20* (5), 485-491. DOI: 10.1080/14786410500513207.
(88) bin Daud, S.; Ee, G. C. L.; Malek, E. A.; Ahmad, Z.; Hashim, N. M.; See, I.; Teh, S. S.; Ismail, A. A. F. A New Pyranoxanthone from the Stem Bark of *Calophyllum buxifolium*. *Chem Nat Comp* **2016**, *52* (5), 807-809. DOI: 10.1007/s10600-016-1783-4.
(89) Elya, B.; He, H. P.; Kosela, S.; Hanafi, M.; Hao, X. J. Two new xanthons from *Garcinia rigida* leaves. *Nat Prod Res* **2006**, *20* (9), 788-791. DOI: 10.1080/14786410500049434.
(90) Raksat, A.; Maneerat, W.; Andersen, R. J.; Pyne, S. G.; Laphookhieo, S. A tocotrienol quinone dimer and xanthones from the leaf extract of *Garcinia nigrolineata*. *Fitoterapia* **2019**, *136*, 104175. DOI: 10.1016/j.fitote.2019.104175.
(91) Nomura, T.; Hano, Y.; Okamoto, T. Components of the Root Bark of *Morus insignis Bur.* 2. Structures of Four New Isoprenylated Xanthones, Morusignins E, F, G. and H. *Heterocycles* **1991**, *32* (7), 1357-1364. DOI: 10.3987/COM-91-5754.
(92) Nuangnaowarat, W.; Phupong, W.; Isaka, M. New Xanthones from the Barks of *Cratoxylum Sumatranum* Ssp. Neriifolium. *Heterocycles* **2010**, *81*, 2335-2341. DOI: 10.3987/COM-10-11994.
(93) Boonnak, N.; Khamthip, A.; Karalai, C.; Chantrapromma, S.; Ponglimanont, C.; Kanjana-Opas, A.; Tewtrakul, S.; Chantrapromma, K.; Fun, H.-K.; Kato, S. Nitric Oxide Inhibitory Activity of Xanthones from the Green Fruits of *Cratoxylum formosum* ssp. pruniflorum. *Aus J Chem* **2010**, *63* (11), 1550-1556. DOI: 10.1071/CH10193.
(94) Lee, B. W.; Lee, J. H.; Lee, S.-T.; Lee, H. S.; Lee, W. S.; Jeong, T.-S.; Park, K. H. Antioxidant and cytotoxic activities of xanthones from *Cudrania tricuspidata*. *Bioorg Med Chem Lett* **2005**, *15* (24), 5548-5552. DOI: 10.1016/j.bmcl.2005.08.099.
(95) Nkengfack, A. E.; Mkounga, P.; Fomum, Z. T.; Meyer, M.; Bodo, B. Globulixanthones A and B, two new cytotoxic xanthones with isoprenoid groups from the root bark of *Symphonia globulifera*. *J Nat Prod* **2002**, *65* (5), 734-736. DOI: 10.1021/np010478w.
(96) Jo, Y. H.; Kim, S. B.; Liu, Q.; Hwang, B. Y.; Lee, M. K. Prenylated Xanthones from the Roots of *Cudrania tricuspidata* as Inhibitors of Lipopolysaccharide-Stimulated Nitric Oxide Production. *Arch Pharm Chem Life Sci* **2017**, *350* (1), e1600263. DOI: 10.1002/ardp.201600263.
(97) Zhu, W.; Qiu, J.; Zeng, Y.-R.; Yi, P.; Lou, H.-Y.; Jian, J.-Y.; Zuo, M.-X.; Duan, L.; Gu, W.; Huang, L.-J.; Li, Y.-M.; Yuan, C.-M.; Hao, X. Cytotoxic phenolic constituents from *Hypericum japonicum*. *Phytochemistry* **2019**, *164*, 33-40. DOI: 10.1016/j.phytochem.2019.04.012.
(98) Krishna Chaithanya, K.; Gopalakrishnan, V. K.; Hagos, Z.; Kamalakararao, K.; Noyola, P. P.; John Dogulas, P.; Govinda Rao, D. Isolation and Structural Characterization of Bioactive Anti-Inflammatory Compound Mesuaferrin-A from *M. Ferrea*. *Anal Chem Lett* **2019**, *9* (1), 74-85. DOI: 10.1080/22297928.2019.1573702.

(99) Nkengfack, A. E.; Mkounga, P.; Meyer, M.; Fomum, Z. T.; Bodo, B. Globulixanthones C, D and E: three prenylated xanthones with antimicrobial properties from the root bark of *Symphonia globulifera*. *Phytochemistry* **2002**, *61* (2), 181-187. DOI: 10.1016/s0031-9422(02)00222-4.
(100) Thu, Z. M.; Aung, H. T.; Sein, M. M.; Maggiolini, M.; Lappano, R.; Vidari, G. Highly Cytotoxic Xanthones from *Cratoxylum cochinchinense* Collected in Myanmar. *Nat Prod Commun* **2017**, *12* (11), 1759-1762. DOI: 10.1177/1934578X1701201127.
